# Electronic cigarettes and subsequent cigarette smoking in young people: A systematic review

**DOI:** 10.1111/add.16773

**Published:** 2025-01-30

**Authors:** Rachna Begh, Monserrat Conde, Thomas R. Fanshawe, Dylan Kneale, Lion Shahab, Sufen Zhu, Michael Pesko, Jonathan Livingstone‐Banks, Nicola Lindson, Nancy A. Rigotti, Kate Tudor, Dimitra Kale, Sarah E. Jackson, Karen Rees, Jamie Hartmann‐Boyce

**Affiliations:** ^1^ Nuffield Department of Primary Care Health Sciences University of Oxford Oxford UK; ^2^ EPPI Centre, Social Research Institute, UCL Institute of Education University College London London UK; ^3^ Department of Behavioural Science and Health University College London London UK; ^4^ Department of Economics University of Missouri Columbia MO USA; ^5^ Tobacco Research and Treatment Center, Department of Medicine Massachusetts General Hospital and Harvard Medical School Boston MA USA; ^6^ National Endowment for Science, Technology and the Arts (NESTA) London UK; ^7^ Clinical Trials Unit, Warwick Medical School University of Warwick Coventry UK; ^8^ Department of Health Promotion and Policy University of Massachusetts Amherst Amherst MA USA

**Keywords:** e‐cigarettes, gateway hypothesis, smoking, systematic review, young adults, youth

## Abstract

**Aims:**

To assess the evidence for a relationship between the use of e‐cigarettes and subsequent smoking in young people (≤29 years), and whether this differs by demographic characteristics.

**Methods:**

Systematic review with association direction plots (searches to April 2023). Screening, data extraction and critical appraisal followed Cochrane methods. Our primary outcome was the association between e‐cigarette use, availability or both, and change in population rate of smoking in young people. The secondary outcomes were initiation, progression and cessation of smoking at individual level. We assessed certainty using Grading of Recommendations, Assessment, Development and Evaluations (GRADE).

**Results:**

We included 126 studies. For our primary outcome, there was very low certainty evidence (limited by risk of bias and inconsistency) suggesting that e‐cigarette use and availability were inversely associated with smoking in young people (i.e. as e‐cigarettes became more available and/or used more widely, youth smoking rates went down or, conversely, as e‐cigarettes were restricted, youth smoking rates went up). All secondary outcomes were judged to be very low certainty due to very serious risk of bias. Data consistently showed direct associations between vaping at baseline and smoking initiation (28 studies) and smoking progression (5 studies). The four studies contributing data on smoking cessation had mixed results, precluding drawing any conclusion on the direction of association. There was limited information to determine whether relationships varied by sociodemographic characteristics.

**Conclusion:**

At an individual level, people who vape appear to be more likely to go on to smoke than people who do not vape; however, it is unclear if these behaviours are causally linked. Very low certainty evidence suggests that youth vaping and smoking could be inversely related.

## INTRODUCTION

Combustible tobacco use kills more than 8 million people globally each year [1]. Electronic cigarettes (ECs) are electronic vaping devices that are handheld and produce an aerosol formed by heating an e‐liquid, designed for inhalation by the user [2]. Regulatory approaches for ECs vary widely, from no regulation to partial and complete bans [3]. Where ECs are permitted, sales are often restricted to adults [4].

There is considerable debate over the role of EC in influencing combustible tobacco use in young people. Multiple routes have been theorized. Stances such as the ‘gateway’ [5], ‘common liability’ [6] and ‘diversion’ hypotheses, or even a combination of such factors, have been postulated as possible explanatory mechanisms, either causally linking vaping with increased risk of smoking in young people (‘gateway’), causally linking vaping with a reduction in smoking in young people (‘diversion’) or suggesting no causal relationship (‘common liability’). A lack of empirical support for these hypotheses persists [7].

Previous systematic reviews and meta‐analyses in this area have focused on longitudinal, individual‐level studies; although consistently detecting a positive association between smoking and vaping, conclusions differ as to whether this is causal [8, 9]. Given the complexity of the topic, a robust and thorough review of the available individual‐ and population‐level evidence is needed to inform policy, interventions and future research.

## METHODS

We published the protocol for this systematic review [10]. Post‐protocol changes were pre‐registered on https://osf.io/4wycq/.

### Search strategy

We searched the Cochrane Tobacco Addiction Group Specialized Register (CRS‐Web), MEDLINE (OVID SP), Embase (OVID SP), PsycINFO (OVID SP) from 2004 to 3 April 2023. See Data S1 for full search strategy.

A call for studies circulated on our networks did not result in additional studies. One author later identified two studies [11, 12] that our searches had missed (see Discussion).

### Eligibility criteria

- Individual‐level: cohort studies that prospectively collected data on e‐cigarettes and combustible tobacco smoking from the same individuals at a minimum of two timepoints.
- Population‐level: studies with repeated cross‐sectional measures that evaluated combustible tobacco use in young people in relation to e‐cigarette use or availability (or both) (aggregate‐level data analyses).

We included studies with participants age ≤29 years or with extractable data on this subgroup [10]. There was discussion among the author team as to whether to create a cut‐off for the number of participants in an individual‐level study, because of limitations with small observational studies. The decision was taken to include studies regardless of sample size, but to further sub‐divide individual‐level studies into two categories: Tier 1 studies comprised 5000 or more participants; Tier 2 studies comprised fewer than 5000 participants. We focus on Tier 1 studies in the analysis and assessed their risk of bias. Tier 2 studies are reported for descriptive purposes.

### Outcomes

The primary outcome was the association between EC use, availability or both and change in population rate of tobacco use in young people, assessed through the proportion reporting current cigarette use (using definitions provided by study authors). Where studies only reported combustible tobacco use, but did not provide a breakdown by type (e.g. cigarettes, cigars), we included these data as a proxy measure for cigarette smoking.

The secondary outcomes were the association between EC use, availability or both and initiation (defined as the rate at which young people begin smoking in a specified time frame), progression (to include progression from never‐smoking to ever smoking, occasional use or regular use) and cessation (as defined by study authors) of cigarette smoking.

### Data collection and analysis

Screening was conducted independently by two review authors in Covidence, with discrepancies resolved via discussion or through referral to a third author.

Two reviewers extracted outcome data and assessed risk of bias independently using a pre‐specified and piloted data extraction form, with discrepancies resolved via discussion or through referral to a third reviewer. Extraction of study characteristics was carried out by one review author and checked by another. Data extracted included characteristics of studies, outcomes, covariates/confounders (PROGRESS‐Plus indicators), funding and conflicts of interest. See Data S1 for full list.

### Risk of bias assessment

When we started this review, there was no specific tool available for assessing risk of bias in our eligible study designs [13, 14]. We adapted the risk of bias instrument for non‐randomized studies (NRS) of exposures, developed by Morgan *et al*. [15]. This process is described in Data S1, and our tool is available at https://osf.io/svgud. Domains could be judged to be at low, moderate, serious or critical risk of bias because of:

- confounding
- selection of participants
- classification of interventions
- deviations from intended interventions
- missing data
- measurement of outcome
- selection of the reported result

The overall risk of bias for each study was judged based on the highest risk of bias allocated for any domain of the tool.

### Data synthesis

Clinical heterogeneity precluded meta‐analysis. We conducted qualitative comparative analysis (QCA) [16] to investigate patterns in our findings; this was inconclusive and is reported in Data S1, 1.4.1 and Data S2, 4. Our main findings are synthesized in association direction plots grouped by outcome, by study type (population‐level; individual‐level Tier 1; individual‐level Tier 2), and by exposure (where relevant). Exposures were classified as EC availability and EC use, and within the latter, as current use (past 30 day use or more frequent) or ever use (using definitions of ever use as provided by authors, but also including studies where use could be less recent than past 30 day or where frequency or timing of use was not specified). Full details can be found in Data S1, 1.4.2. Associations were categorized as inverse, where the association was negative (e.g. as vaping went up, smoking went down), direct, where the association was positive (e.g. as vaping went up, smoking went up) and as no evidence of an association.

We used PROGRESS‐Plus indicators [17] where reported in studies to assess whether the effects differed across subgroups. [17] We explored the degree to which findings were sensitive to definitions used for both EC and conventional cigarette use via QCA and in association direction plots.

We created a summary of findings table for all primary and secondary outcomes. Certainty was assessed using Grading of Recommendations, Assessment, Development, and Evaluations (GRADE) [18] for observational data [19]. Judgement did not reflect whether an association existed, but whether the association was causal.

## RESULTS

### Search results

After duplicates were removed, we screened 9057 titles and abstracts and retrieved the full‐text of 1219 potentially relevant articles [Preferred Reporting Items for Systematic Reviews and Meta‐analysis (PRISMA) flowdiagram in Data S2, 1; characteristics of excluded studies in Data S4. A total of 126 papers were included in this review.

### Study characteristics

In total, we included 126 studies, with 27 population‐level studies, 40 Tier 1 studies and 59 Tier 2 studies.

The included population‐level studies represented approximately 4 015 664 participants, and our best estimate is that individual‐level studies represented approximately 500 000 participants (an exact number is not possible, given that five population‐level studies did not report the total number of participants and cohorts overlapped).

Ninety‐eight studies were conducted in the United States (US), eight in the United Kingdom (UK), five in Canada, three in Taiwan, one each in Australia, Mexico, Thailand, the Netherlands, Germany, Finland, Romania, Indonesia, Hong Kong and three across multiple countries. Participants' ages ranged between 9 and 29 years.

Most studies used state or national surveys; 29 used the Population Assessment of Tobacco and Health (PATH) study, 10 used National Youth Tobacco Surveys (NYTS) and the rest used other longitudinal datasets or collected original data. Details of each included study can be found in the Data S3.

### Risk of bias

No studies were judged to be at low overall risk of bias. We judged 11 population‐level studies to be at moderate risk of bias, nine at serious risk and seven at critical risk. Twenty‐seven Tier 1 individual‐level studies were deemed to be at serious and 13 at critical risk of bias. Details of risk of bias judgements for each included study can be found in Data S2, 3.

### Change in population rate of combusted tobacco use

Only population‐level studies contributed data to this outcome. We synthesized data using both QCA and association direction plots. The results of the QCA were inconclusive and are reported in full in Data S2, 4.

Twenty‐one studies contributed data to our association direction plots. All analysed data at time periods exceeding 3 months, except for Kowitt *et al*. [20], which analysed data from a 3‐month period. Fourteen studies overlapped with other studies in this set: NYTS [12, 21, 22, 23, 24, 25, 26, 27], Monitoring the future (MTF) [27, 28] and Youth Risk Behavior Surveillance System (YRBSS) [28, 29, 30, 31].

### Exposure: EC availability

Twenty‐one studies evaluated EC availability as the exposure of interest and provided appropriate data for categorization (Table 1). Of these, 12 found a statistically significant inverse association between EC availability or accessibility and subsequent smoking, and nine of these were judged to be at moderate risk of bias and three at serious risk of bias. A further three studies (one moderate risk of bias, one serious, one critical) also found an inverse association, but the finding was not statistically significant. Two studies (one serious risk of bias, one critical) found a direct association, but this was not statistically significant. Two, both judged to be at critical risk of bias, found a statistically significant direct association between EC availability and subsequent smoking. The final two studies, judged to be at critical and moderate risk of bias showed ‘no evidence of an association’.

**TABLE 1:** Association direction plot.

Study ID	Country (dataset)	Risk of bias overall	Exposure	Association direction	Brief summary of contributing data
Abouk *et al*. [32] 2017 MTF	USA	Critical	E‐cigarette sales bans	=*	Source: 2007–2014 waves of the MTF to examine the effect of prohibiting e‐cigarette sales on smoking. Findings: past 30‐day smoking rates declined by 2.01 percentage points (*P* < 0.05) after the e‐cigarette sales ban among high school seniors. The authors quoted a 16.2% mean smoking rate among the 12th graders and estimated that a 2.01 percentage point decline in smoking translated as a 12.4% relative decline in smoking as a result of the ban. Breakdowns for 8th and 10th graders suggested an inverse association, but were not statistically significant.
Abouk *et al*. [31] 2023a MTF and YRBSS	USA	Moderate	E‐cigarette taxes	X* (MTF sample) X (YRBSS sample)	Source: Policy data matched by quarter for the annual MTF (2014–2019) and by year for the YRBSS (2015–2019) to estimate the impact of ENDS taxes on youth tobacco use. Findings: among the MTF sample, higher ENDS taxes significantly increased current cigarette use; a $1.00 increase in standardized ENDS tax increased cigarette use by 1.3 percentage points (95% CI = 0.1, 2.6; *P* < 0.05). Among the YRBSS sample, estimates also suggested that higher ENDS taxes increased cigarette use (0.007, 95% CI = −0.024, 0.0385, *P* = 0.66) ‘with coefficient estimates largely similar to the MTF estimates although imprecise because of inflated standard errors.’
Abouk *et al*. [11] 2023b (Pregnancy)	USA	Moderate	E‐cigarette taxes	X*	Source: administrative birth records from the National Center for Health Statistics, 2013–2019 Findings: overall (all ages): ‘coefficient estimate suggests a $1.00 increase in the e‐cigarette tax increases the probability of pre‐pregnancy smoking by 0.5 percentage points’ (*P* < 0.05). Table 6 reports results for our populations of interest. For the subgroup of women age <18, authors report 2.06 percentage point change; for those 18–30, 4.8 percentage point change.
Cantrell *et al*. [35] 2020 TLC	USA	Critical	E‐cigarette prices	X	Source: 2014–2016 waves of the TLC to examine the impact of e‐cigarette prices on cigarette use among 15‐ to 21‐year‐olds. Findings: The price of rechargeable e‐cigarettes (per $) was positively associated with past 30‐day cigarette use (OR = 1.02; 95% CI = 1.00, 1.04; *P* > 0.05).
Creamer *et al*. [21] 2021 NYTS	USA	Serious	Market introduction of e‐cigarettes	X*	Source: Data from 2004–2018 waves of the NYTS to examine whether youth cigarette smoking changed after the introduction of e‐cigarettes. Finding: there was a continuous decline in ever and current cigarette smoking from 2004–2018, with a notable breakpoint in 2012 for ever smoking and 2014 for current smoking. Before 2012, prevalence for ever smoking declined at a rate of 1.45 percentage points per year (95% CI = −1.59, −1.31) whereas in 2012, it dropped 1.83 percentage points (95% CI = −2.52, −1.14), and after 2012, it declined at a rate of 1.71 percentage points per year (95% CI = −1.75, −1.66) ‘indicating that the decline in ever cigarette smoking was decreasing faster (−0.26 95% CI = −0.39, −0.12) after the breakpoint in 2012’. For current smoking, before 2014, prevalence declined at a rate of 0.75 percentage points per year (95% CI = −0.81, −0.68) whereas during 2014, prevalence for current smoking dropped 1.89 percentage points (95% CI = −2.36, −1.41); thereafter, the rate of decline then slowed significantly from 0.49 percentage points (95% CI = −0.35, −0.63) to 0.26 (95% CI = −0.40, −0.12).
Dave *et al*. [28] 2019 YRBSS	USA	Moderate	E‐cigarette minimum legal sale age laws	X*	Source: 2005–2015 waves of the YRBSS to examine the effects of e‐cigarette MLSA laws on youth cigarette smoking. Findings: e‐cigarette MLSA laws increased the probability of initiating smoking in underage youth (below the age of 18) by 0.7 percentage points (SE = 0.3; *P* < 0.05), although no evidence that the increase in smoking persisted when youth aged out of the e‐cigarette MLSA restriction.
Dutra *et al*. [36] 2017	USA	Serious	Market introduction of e‐cigarettes	No association	Source: data from 2004–2014 waves of the NYTS to examine whether youth cigarette smoking changed after the introduction of e‐cigarettes. Findings: ‘Ever cigarette smoking, including dual use after 2011, showed a continuous linear decline over the study period (2004–2014; *P* = 0.009), with no significant slope change after the introduction of e‐cigarettes in 2009 (*P* = 0.57). Similarly, there was a decline in current smoking (*P* = 0.05) that did not change after the introduction of e‐cigarettes (*P* = 0.23).’
Dutra *et al*. [22] 2018 NYTS	USA	Critical	E‐cigarette minimum legal sale age laws	=	Source: 2009–2014 waves of the NYTS to assess the relationship between e‐cigarette MLSA laws and youth cigarette smoking. Findings: cigarette smoking was not significantly associated with lagged MLSA laws after adjusting for year (OR = 0.87, 95% CI = 0.73–1.03; *P* = 0.10) and covariates (OR = 0.85, 0.69–1.03; *P* = 0.10). Unlagged laws were significantly and negatively associated with cigarette smoking (OR = 0.84, 0.71–0.98, *P* = 0.02), but not after adjusting for covariates (OR = 0.84, 0.70–1.01, *P* = 0.07). The authors also reported that ‘e‐cigarette and other tobacco use, sex, race/ethnicity, age, and smoke‐free laws were associated with cigarette smoking (*P* < 0.05). Results unadjusted for e‐cigarette use and other tobacco use yielded a significant negative association between e‐cigarette MLSA laws and cigarette smoking (lagged: OR = 0.78, 0.64–0.93, *P* = 0.01; unlagged: OR = 0.80, 0.68–0.95, *P* = 0.01).’
Friedman [37] 2015a NSDUH	USA	Moderate	E‐cigarette sales bans to minors	X*	Source: 2002–2013 waves of the NSDUH to examine how state‐level bans on e‐cigarette sales bans to minors influence smoking rates among 12‐ to 17‐year‐olds. Findings: state bans on e‐cigarette sales to minors yielded a statistically significant 0.9 percentage point increase in combustible tobacco use, relative to states without such bans (*P* < 0.01) ‘with the impact only evident once the ban goes into effect, and only among those subject to the ban (i.e. under age 18).’
Friedman [38] 2015b (Dissertation) NYTS	USA	Serious	E‐cigarette sales	X*	Source: 2004–2012 waves of the NYTS to examine the impact of changes in e‐cigarette availability on smoking rates among 14‐ to 18‐year‐olds with different propensities to smoke (low, medium, high). Findings: in analyses that included e‐cigarette sales as an interaction term, there was a statistically significant 2.0 and 2.4 percentage point reduction in current smoking in middle and high propensity to smoke groups respectively, for every $100 million increase in e‐cigarette sales (*P* < 0.01). There was a non‐statistically significant 0.04 percentage point increase in smoking in the low propensity to smoke group, although ‘overall, the e‐cigarette sales coefficients are indicative of harm reduction in the middle and high propensity to smoke groups, in response to increased e‐cigarette availability.’
Friedman and Pesko [39] 2022 CPS‐TUS	USA	Moderate	E‐cigarette taxes	X*	Source: 2010–2019 waves of the CPS‐TUS to measure the relationship between ENDS and cigarette tax rates and ENDS use in 18‐ to 25‐year‐olds. Findings: ENDS taxes yielded statistically significant increases in recent cigarette use; there was a 3.7 percentage point increase in recent smoking for every $1 increase in ENDS taxes (95% CI = 1.30, 6.12). There was a marginal non‐significant increase of 2.5 percentage points in daily cigarette use (*P* = 0.054). Recent smoking and daily smoking were more common in states without ENDS taxes than states with taxes (15.6% vs. 12.8% and 11.2% vs. 8.8% respectively; *P* < 0.001 for both).
Gao *et al*. [40] 2021 TGYTS, Taiwan Adult Smoking Behaviour Survey	Taiwan	Critical	E‐cigarette popularity	X*	Source: data from 2005 to 2017 waves of the TGYTS and Taiwan Adult Smoking Behaviour Survey. Findings: ‘The long‐term annual relative decline in the smoking rate among senior high school students was −2%, while after e‐cigarettes started to gain popularity, the annual relative decline was −10%. In other words, smoking prevalence decreased five times faster once e‐cigarettes became popular.’
Hallingberg *et al*. [41] 2020 SDDU, SALSUS, HBSC	England, Wales and Scotland	Critical	Market introduction of e‐cigarettes	=*	Source: 1998–2015 data from the SDDU, the biennial SALSUS, and for Wales, HBSC survey (from 1998–2013) and the SHRN survey (2015) to examine the association between the introduction of e‐cigarettes (2010–2015) and smoking prevalence. Findings: no significant change was observed in the rate of decline for ever smoking post‐2010 (OR = 1.01, 95% CI = 0.99, 1.03; *P* = 0.23). There was a marginally significant slowing in the rate of decline for regular smoking post‐2010 (OR = 1.04, 95% CI = 1.00, 1.08; *P* = 0.03).
Harrell *et al*. [25] 2022 NYTS	USA	Moderate	Market introduction of e‐cigarettes	X*	Source: 2002–2019 waves of the NYTS to compare trends in past 30‐day smoking among adolescents before and after the introduction of e‐cigarettes in 2014. Findings: past 30‐day smoking prevalence decreased significantly by 0.75 percentage points (95% CI = −0.82, −0.68; *P* < 0.001) per year, from 2002–2013. From 2013–2014, smoking prevalence dropped significantly by 1.64 percentage points (95% CI = −2.33, −0.95) *P* < 0.001). Past 30‐day smoking prevalence continued to decrease from 2015–2019, but the decrease in prevalence was significantly less (0.37 percentage points) compared to the observed decrease from 2002–2013 (difference in decrease in prevalence, β = 0.38 percentage points; 95% CI = 0.21, 0.55; *P* = 0.001).
Hawkins *et al*. [42] 2022 YHS	USA	Serious	E‐cigarette bans	=	Source: 2011 to 2017 waves of the Massachusetts YHS to examine associations between county‐level flavoured tobacco product restrictions, tobacco 21 policies and smoke‐free laws prohibiting e‐cigarettes with adolescent e‐cigarette use. Findings: no evidence of a significant association between smoke‐free laws prohibiting e‐cigarettes and cigarette use; in Table 1, the coefficients given indicate a direct but not statistically significant association between these laws and cigarette smoking (inflation model coefficient −0.90, 95% CI = −1.88, 0.07; negative binomial model coefficient −0.21, −1.04 to 0.63).
Kowitt *et al*. [20] 2022	Indonesia	Critical	E‐cigarette taxes	No association	Source: data from a pre‐post on‐line survey conducted in Indonesia in a cohort of adults between September 2018 (wave 1) and November–December 2018 (wave 2) to investigate the impact of e‐liquid tax on e‐cigarette and cigarette use. Findings: in a subgroup analysis of 18‐ to 24‐year‐olds, no evidence of a significant association was found between e‐liquid tax and increased use vs no change in use of cigarettes (OR = 1.05, 95% CI = 0.66, 1.67) or decreased use of cigarettes (OR = 1.04, 95% CI = 0.70, 1.55). Note: we class this as no association given the contradictory directions of association between the two measures.
Nguyen and Bornstein [43] 2021 CTADS, CTUMS	Canada	Serious	E‐cigarette bans	X	Source: 2004–2017 waves of the CTADS and its predecessor, the CTUMS to investigate the association between banning e‐cigarette use in public places and workplaces and cigarette use. 2015–2017 was the policy implementation period. No evidence of an association was found between the e‐cigarette bans and current combustible cigarette use (β = 0.9 percentage points, 95% CI = −1.9, 3.7; *P* = 0.488). Coefficients were also small and statistically non‐significant for ever cigarette use (β = 3.1 percentage points; 95% CI = −2.2, 8.3; *P* = 0.218).
Pesko *et al*. [29] 2016 YRBSS	USA	Moderate	E‐cigarette minimum legal sales age laws	X*	Source: state‐level aggregated data from the 2007, 2009, 2011 and 2013 waves of the YRBSS to examine the impact of ENDS age purchasing restrictions on cigarette use. Findings: ‘ENDS age purchasing restrictions are associated with a 3.1 percentage point (17.9% of the mean) increase in adolescent cigarette use (95% CI = 0.20, 5.95; *P* < 0.05) in the period of implementation.’
Pesko and Currie [44] 2019	USA	Serious	E‐cigarette minimum legal sales age laws	X*	Source: Administrative birth records with geocoded information provided by the National Center for Health Statistics from 2010–2016 in 32 USA states; the study investigated the effects of e‐cigarette minimum legal sale age laws on prenatal cigarette smoking and birth outcomes for underage rural teenagers. Findings: MLSA laws increased prenatal smoking by 0.2 percentage points in all pregnant teens (SE = 0.1; *P* < 0.05, 3.2% of the mean). The effects were greater among rural pregnant teens, where the laws yielded a 0.6 percentage point increase in smoking (SE = 0.3; *P* < 0.05, 4.8% of the mean).
Pesko and Warman [27] 2021 NYTS	USA	Moderate	E‐cigarette prices	X	Source: 2011–2015 waves of the NYTS to investigate the relationship between e‐cigarette and cigarette price and tax changes on cigarette use. Findings: Authors report e‐cigarette prices had no statistically significant effect on youth past‐30 day smoking, neither for cartridge state prices (−1.16 percentage points; SE = 1.82; *P* > 0.05) nor disposable state prices (−0.21 percentage points; SE = 0.68; *P* > 0.05); both associations inverse, but not statistically significant. Authors report statistically significant inverse association with number of cigarettes smoked.
Pesko [12] 2023 NYTS	USA	Moderate	E‐cigarette minimum legal sales age laws	X*	Source: 2000–2017 waves of the NYTS Findings: ‘Using an estimator designed to correct for dynamic heterogeneity in treatment effects, e‐cigarette MLSAs are estimated to reduce lifetime e‐cigarette use by approximately 25% and increase daily cigarette use and daily cigar use by approximately 35%.’ (*P* < 0.05)

*Notes*: Exposure: e‐cigarette availability and accessibility; outcome: population smoking rates in young people at 3 months or longer following the exposure of interest (=, direct association, not statistically significant; =*, statistically significant direct association; X, inverse association, not statistically significant; X*, inverse association, statistically significant).

Abbreviations: CTADS, Canadian Tobacco, Alcohol and Drugs Survey; CPS‐TUS, Current Population Survey's Tobacco Use Supplement; CTUMS, Canadian Tobacco Use Monitoring Survey; HBSC, Health Behaviour in School‐Aged Children; MLSA, minimum legal sale age; MTF, Monitoring the Future surveys; NSDUH, National Survey on Drug Use and Health; NYTS, National Youth Tobacco Surveys; SALSUS; Scottish Adolescent Lifestyle and Substance Use Survey; SDDU, Smoking Drinking and Drug Use Among Young People in England Survey; SHRN, School Health Research Network; TGYTS, Taiwan Global Youth Tobacco Survey; TLC, Truth Longitudinal Cohort; USA, United States of America; YHS, Youth Health Survey; YRBSS, Youth Risk Behaviour Surveillance System.

A further two studies contributed data but could not be categorized based on direction of association. Schneller *et al*. [45] described tobacco and nicotine use patterns in young people who used ECs in the past 30 days. Following a state‐wide vaping flavor restriction policy, they found patterns in smoking varied over time. Wu *et al*. [46] analysed data from Canada, the United Kingdom and Australia and found different trends following the market introduction of EC depending on country and gender. They concluded that in contexts supportive of substitution of smoking with vaping, ECs were associated with reduced smoking, but did not provide statistical analyses of associations as a whole.

### Exposure: Rates of EC use

Of the four studies that evaluated prevalence of EC use as the exposure of interest, we were able to categorise association direction for three (Table 2). One (serious risk of bias) did not find any evidence of an association, the other two (one critical risk of bias, one serious) found evidence of a statistically significant inverse association.

**TABLE 2:** Association direction plot.

Study ID	Country (dataset)	Risk of bias overall	Exposure	Association direction	Brief summary of contributing data
Beard *et al*. [47] 2022 STS	UK	Serious	EC use	No association	Source: 2007–2018 waves of the STS to examine how changes in the prevalence of ECs among young adults is associated with changes in uptake of smoking. Findings: ‘There was no evidence of an association between the prevalence of e‐cigarettes and ever regular smoking among 16‐ to 24‐year‐olds in the unadjusted (B = −0.013, 95% CI = –0.046, 0.021, *P* = 0.461 and adjusted models (B = −0.015, 95% CI = –0.046, 0.016, *P* = 0.341). Similar results were found in unadjusted models for 16‐ to 17‐year‐olds (B = 0.077, 95% CI = –0.006, 0.159, *P* = 0.068) and 18‐ to 24‐year‐olds (B = −0.018, 95% CI = –0.052, 0.016, *P* = 0.301).’ ‘… the null hypothesis was more likely than, on average, a 1% increase in the prevalence of EC use being associated with more than a 0.310 percentage point increase in ever regular smoking among 16‐ to 17‐year‐olds and a 0.013 percentage point increase in ever regular smoking among 18‐ to 24‐year‐olds. Conversely, respective negative associations less than −0.028 and −0.080 percentage points could also be ruled out.’
Foxon and Selya [24] 2020 NYTS	USA	Critical	EC use	X*	Source: 1999–2018 waves of the NYTS to examine prevalence trends of exclusive EC use, cigarette use and dual use over time, ages of initiation and established use. Findings: ‘Exclusive cigarette use prevalence declined from 1999–2018, while exclusive EC use and dual use prevalences increased since their introduction in 2009. The age of cigarette initiation began a slight increase after 2014, whereas the age for EC use remained approximately constant and was higher than that of cigarettes. The counterfactual comparison results were consistent with ECs not increasing the number of US adolescent nicotine users, and in fact diverting adolescents from cigarettes.’
Levy *et al*. [30] 2019 MTF, NYTS, YRBSS, NSDUH, NHIS	USA	Serious	EC use	X*	Source: Data from 2011–2017 waves of the MTF survey; NYTS; YRBS; NSDUH; and NHIS for young adults to examine the temporal relationship between vaping and youth smoking using multiple data sets to explore the question of whether vaping promotes smoking initiation. Findings: ‘There was a substantial increase in youth vaping prevalence beginning in about 2014. Time trend analyses showed that the decline in the past 30‐day smoking prevalence accelerated by two to four times after 2014. Indicators of more established smoking rates, including the proportion of daily smokers among past 30‐day smokers, also decreased more rapidly as vaping became more prevalent.’

*Notes*: Exposure: EC use in young people; outcome: population smoking rates in young people at 3 months or longer following the exposure of interest (=*,statistically significant direct association).

Abbreviations: EC, e‐cigarettes; MTF, Monitoring the Future survey; NHIS, National Health Interview Survey; NSDUH, National Survey of Drug Use and Health; NYTS National Youth Tobacco Survey; STS; Smoking Toolkit Study; UK, United Kingdom; US, United States; USA, United States of America; YRBS, Youth Risk Behavior Survey.

A third study, Shahab *et al*. [26], provided relevant data, but we did not judge the association direction based on the retrospective study design. Adolescents who tried EC first were less likely to have ever smoked cigarettes than those who first used non‐cigarette combustible tobacco or other non‐combustible tobacco. They were also less likely to be past 30‐day or established cigarette smokers compared with those that had first used a cigarette, other combustible tobacco or other non‐combustible tobacco.

### Initiation

For this and all other secondary outcomes, we rely on data from individual level studies—all at critical or serious risk of bias—unless otherwise indicated. Studies contributing to this outcome consistently showed direct associations between vaping and subsequent smoking initiation. All studies focused on EC use as the exposure.

Nine Tier 1 studies contributed data relating to current EC use. As indicated in Table 3, many relied on the same datasets. Eight of these reported statistically significant direct associations between current vaping and subsequent smoking (in one this was not statistically significant in Hispanic white participants), and all were judged at serious or critical risk of bias.

**TABLE 3:** Association direction plot.

Study ID	Country (dataset)	Risk of bias overall	Exposure	Association direction	Brief summary of contributing data
Aleyan *et al*. [48] 2021	Canada (COMPASS)	Serious	Past 30‐day use	=*	Source: Three waves (2015–2016, 2016–2017, 2017–2018) of the COMPASS study to investigate potential mediating factors between e‐cigarette use and smoking uptake among Canadian youth. Findings: In adjusted logistic regression models, past 30‐day e‐cigarette use at wave 1 significantly predicted past 30‐day cigarette smoking at wave 3 (β = 1.06, 95% CI = 0.52, 1.60; *P* < 0.001).
Barrington‐Trimis *et al*. [49] 2019	USA CHS, H&H and YASS	Critical	Past 30‐day use	=* (non‐Hispanic white) = (Hispanic White)	Source: Baseline 2013–2014 and follow‐up data 2014–2015 from 3 cohort studies: Southern CHS, H&H and YASS. Findings: In the non‐Hispanic White sample, exclusive use of e‐cigarettes at baseline was significantly associated with exclusive cigarette use at follow‐up vs. no use (OR = 4.20, 95% CI = 1.87, 9.44). In the Hispanic White sample, exclusive use of e‐cigarettes at baseline was not significantly associated with exclusive cigarette use at follow‐up vs. no use (OR = 1.27, 95% CI = 0.47, 3.46). ‘Exclusive e‐cigarette users at baseline had higher odds of reporting e‐cigarette or dual product use at follow‐up than of reporting exclusive cigarette use at follow‐up (although differences in ORs were not significant)’.
Duan *et al*. [50] 2021	USA (PATH)	Serious	Past 30‐day use	=*	Source: Waves 1–4 (2013–2018) of the PATH study to estimate associations between baseline e‐cigarette use and subsequent smoking in US adolescents. Findings: Among never smokers at baseline, past 30‐day e‐cigarette use at baseline was significantly associated with cigarette smoking at the follow‐up waves (adjusted OR = 3.90, 95% CI = 2.51, 6.08; *P* < 0.001).
Hair *et al*. [51] 2021b	USA (TLC)	Serious	Past 30‐day use	=*	Source: TLC, January – April 2017; late 2019 (September – December) Findings: ‘Those who were current e‐cigarette users (but never JUUL users) by 2018 had 3.57 times higher odds (95% CI = 1.71, 7.47) of reporting flavored CLCC [cigar, little cigar, or cigarillo] use between 2018 and 2019 compared to never users’.
Hammond *et al*. [52] 2017	Canada COMPASS	Serious	Past 30‐day use	=*	Source: Baseline (2013–2014) to 1 year follow‐up (2014–2015) of the COMPASS study. Findings: ‘Students who reported past 30‐day e‐cigarette use at baseline were significantly more likely to initiate smoking at follow‐up’ (adjusted OR = 2.12, 95% CI = 1.68, 2.66). Past 30‐day e‐cigarette use was also associated with initiation of daily smoking (adjusted OR = 1.79, 95% CI = 1.41, 2.28).
Harlow *et al*. [53] 2022	USA (PATH)	Serious	Past 30‐day use	=*	Source: Wave 1 (2013–2014), wave 2 (baseline; 2014–2015), wave 3 (2015–2016), wave 4 (2016–2018), and wave 5 (2018–2019) of the PATH study. Findings: Baseline‐adjusted RR for the association between e‐cigarette use and current cigarette smoking initiation was 3.5 (95% CI = 2.9, 4.1), and after accounting for time‐dependent confounding and selection bias using MSMs, the RR was 3.1 (95% CI = 2.6, 3.7). The baseline‐adjusted RR was 3.8 (95% CI = 3.1, 4.6) for current e‐cigarette use relative to never e‐cigarette use. In weighted MSMs, the RR attenuated to 3.4 (95% CI = 2.8, 4.2).
Loukas *et al*. [33] 2022	USA (PROJECT M‐PACT)	Serious	Past 30‐day use	=*	Source: Project M‐PACT, a longitudinal study spanning a 4.5‐year period from 2014–2019. Findings: After adjusting for covariates, current ENDS use increased the probability of transitioning from never to current cigarette use (initiation) by 2.69 times (95% CI = 1.95, 3.72).
Stanton *et al*. [54] 2019	USA (PATH)	Serious	Past 30‐day use	No association	Source: 2013–2014 (wave 1) and 2014–2015 (wave 2) waves of the PATH study to examine bidirectional associations between ENDS use and cigarette use among 12‐ to 17‐year‐olds. Findings: Among wave 1 never smokers, 1–5 days ENDS use in the past 30 days was not associated with higher odds of new cigarette smoking between wave 1 and wave 2 [OR = 1.02 (95%CI = 0.37, 2.80), *P* = 0.97]. Similarly, there was no association between 6+ days ENDS use in the past 30 days and new cigarette smoking.
Watkins *et al*. [55] 2018	USA (PATH)	Serious	Past 30‐day use	=* (ever cigarette use) = (past 30‐day cigarette use)	Source: Wave 1 (2013–2014) and wave 2 (2014–2015) of the PATH study to assess the longitudinal association between e‐cigarette use (and other non‐cigarette tobacco use) and cigarette smoking initiation in US youth. Past 30‐day use of e‐cigarettes at wave 1 was significantly associated with higher odds of ever cigarette use at wave 2 compared with never e‐cigarette users (OR = 2.65, 95% CI = 1.38, 5.10), and higher odds of past 30‐day cigarette use at wave 2 compared with never e‐cigarette users (OR = 2.08, 95% CI = 0.81, 5.40), although the latter was not statistically significant.

*Notes*: Exposure: current e‐cigarette use in young people; outcome: smoking initiation at follow‐up (=* statistically significant direct association; = direct association, not statistically significant). Tier 1 studies (each study had 5000 + participants).

Abbreviations: CHS, California Children's Health Study; H&H, Happiness and Health Project; Project M‐PACT, Marketing and Promotions across Colleges in Texas project; PATH, Population Assessment of Tobacco and Health; RR relative risk; TLC, Truth Longitudinal Cohort: US, United States; USA, United States of America; YASS, Yale Adolescent Survey Study.

Nineteen Tier 1 studies looked at associations between ever vaping and cigarette initiation (Table 4). All but one found statistically significant direct associations; the other controlled for ‘general liability to use tobacco products’ and reported no association after controlling for this variable [58].

**TABLE 4:** Association direction plot.

Study ID	Country (dataset)	Risk of bias overall	Exposure	Association direction	Brief summary of contributing data
Barrington‐Trimis *et al*. [56] 2018b	USA (CHS)	Critical	Ever use	=*	Source: Southern CHS; H&H Study; YASS (baseline: 2013–2014; follow‐up: 2014–2016) Findings: Among baseline never smokers, ‘Elevated ORs were observed for baseline e‐cigarette use (vs. no use) for cigarette experimentation at follow‐up (OR = 4.57; 95% CI = 3.56–5.87), infrequent smoking (OR = 4.27; 95% CI =2.75–6.62), and frequent smoking (OR = 3.51; 95% CI = 1.97–6.24) versus maintaining never use of cigarettes by follow‐up’
Berry *et al*. [57] 2019	USA (PATH)	Serious	Ever use	=*	Source: Wave 1 (2013–2014), wave 2 (2014–2015) and wave 3 (2015–2016) of PATH. Findings: ‘Prior e‐cigarette users had 4.09 (95% CI = 2.97, 5.63, *P* < 0.001) times the odds of ever cigarette use compared with youths with no prior tobacco use, while prior other product users had 3.84 (95% CI = 2.63, 5.63, *P* < 0.001) times the odds of ever cigarette use. Additionally, the odds of current cigarette use at wave 3 were higher among prior e‐cigarette users (OR = 2.75; 95% CI = 1.60–4.73, *P* < 0.001) and prior other product users (OR = 3.43; 95% CI = 1.88–6.26, *P* < 0.001) compared with youths with no prior tobacco use.’
Cheng *et al*. [58] 2019	USA (PATH)	Serious	Ever use	No association	Source: Wave 1 (2013–2014), wave 2 (2014–2015) and wave 3 (2015–2016) of the PATH study to investigate the relationship between ever e‐cigarette use and cigarette smoking onset in US youth. Findings: Among never smokers at wave 1, e‐cigarette ever use at wave 1 was not associated with cigarette onset at wave 2 ‘after accounting for the general liability to use tobacco products’ (β from structural equation model 0.13; 95% CI = −0.07, 0.32; *P* = 0.204). E‐cigarette ever use at wave 2 was not associated with cigarette onset at wave 3 (β = 0.15; 95% CI = −0.06, 0.35; *P* = 0.157). ‘The latent “common liability to use tobacco products” was a robust predictor for the onset of cigarette smoking (β = 0.38; 95% CI = 0.07, 0.69; *P* = 0.015).’
Chien *et al*. [59] 2019	Taiwan (TAALS)	Serious	Ever use	=*	Source: Wave 1 (2014) to wave 2 (2016) of the TAALS. Findings: ‘Students who had already tried e‐cigarettes at baseline exhibited significantly higher odds of starting smoking in the following 2 years than those who never tried e‐cigarettes; the ORs being 2.44 (95% CI = 1.94, 3.09; *P* < 0.001) with the unadjusted model and 2.14 (95% CI = 1.66, 2.75; *P* < 0.001) with the adjusted model.’
Friedman and Xu [60] 2020	USA (PATH)	Serious	Ever use	=*	Source: Wave 1–4 (2013–2018) of the PATH study. Findings: Among never smokers at baseline, ‘new vaping was positively associated with smoking initiation by wave 3 for youths (aOR = 6.75; 95% CI = 3.93–11.57; *P* < 0.001) and emerging adults (aOR = 3.20; 95% CI = 1.70–6.02; *P* < 0.001). This association held for smoking initiation by wave 4 as well, with aORs of 5.62 for both youths (95% CI = 3.17–9.96; *P* < 0.001) and emerging adults (95% CI = 2.99–10.56; *P* < 0.001)’.
Hair *et al*. [51] 2021b	USA (TLC)	Serious	Ever use	=* (ever used JUUL) = (ever used e‐cigarettes, but never used JUUL)	Source: TLC, January–April 2017; late 2019 (September–December) Findings: ‘Compared to those who had not used e‐cigarettes by 2018, those who had ever used JUUL had 3.30 times higher odds (95% CI = 2.03, 5.36) of initiating CLCC use. A significant association was not observed for those who reported ever using some other e‐cigarette brand rather than JUUL compared to those who had not used any e‐cigarette product by 2018.’ Compared to those who had not used e‐cigarettes by 2018, those who had ever used e‐cigarettes, but never JUUL, had 1.33 times higher odds of initiating CLCC use, however this was not statistically significant (95% CI = 0.82, 2.14).
Harlow *et al*. [53] 2022	USA (PATH)	Serious	Ever use	=*	Source: Wave 1 (2013–2014), wave 2 (baseline; 2014–2015), wave 3 (2015–2016), wave 4 (2016–2018), and wave 5 (2018–2019) of the PATH study. Findings: ‘Before accounting for time‐dependent confounding, baseline‐adjusted RRs for the association between e‐cigarette use and ever cigarette smoking initiation were 2.7 (95% CI = 2.4, 3.0) for ever e‐cigarette use…and 2.5 (95% CI = 2.2, 2.9) for former e‐cigarette use relative to never e‐cigarette use. After accounting for time‐dependent confounding and selection bias using MSMs, RRs for ever cigarette smoking initiation attenuated to 2.4 (95% CI = 2.1, 2.7) for ever e‐cigarette use…and 2.2 (95% CI = 2.0, 2.5) for former e‐cigarette use.’ ‘Among youth who ever initiated e‐cigarettes, MSM–adjusted RRs for ever smoking initiation were 1.8 (95% CI = 1.4, 2.2) for vaping ≥3 days and 1.2 (95% CI = 0.93, 1.6) for vaping 1–2 days compared with vaping 0 days in the past 30 days.’ For current cigarette smoking initiation at follow up waves, ‘baseline‐adjusted RRs were 2.9 (95% CI = 2.5, 3.3) for ever e‐cigarette use…and 2.6 (95% CI = 2.2, 3.1) for former e‐cigarette use relative to never e‐cigarette use. In weighted MSMs, RRs attenuated to 2.5 (95% CI = 2.2, 2.9) for ever e‐cigarette use…and 2.3 (95% CI = 1.9, 2.7) for former e‐cigarette use.’ ‘Among youth who ever initiated e‐cigarettes, MSM–adjusted RRs for current smoking initiation were 1.9 (95% CI = 1.5, 2.6) for vaping ≥3 days and 1.3 (95% CI = 0.92, 1.8) for vaping 1–2 days compared with vaping 0 days in the past 30 days.’
Kasza *et al*. [61] 2020	USA (PATH)	Critical	Ever use	=*	Source: First three waves (2013–2016) of the PATH. Findings: Ever use of ENDS at baseline was significantly associated with higher odds of initiating past 30 day use of cigarettes in 12‐ to 17‐year‐olds compared with never use (adjusted OR = 3.4, 95% CI = 2.4, 4.7; *P* < 0.001).
Lee and Fry [62] 2019	USA (PATH)	Critical	Ever use	=*	Source: Wave 1 (2013–2014 and wave 2 (2014–1015) of the PATH. Finding: In never smokers, ‘the unadjusted OR for the association of vaping by wave 1 with cigarette smoking initiation by wave 2 was 5.702 (95% CI = 4.334–7.502). The OR was markedly reduced by adjustment for the propensity score, whether as quintiles (2.476, 1.852–3.310), as a continuous variable (2.474, 1.791–3.419), or for the 12 variables making up the score (1.847, 1.347–2.533).’
Loukas *et al*. [33] 2022	USA (PROJECT M‐PACT)	Serious	Ever use	=*	Source: Project M‐PACT, a longitudinal study spanning a 4.5‐year period from 2014–2019. Findings: After adjusting for the covariates, ever ENDS use increased the probability of transitioning from never to current cigarette use by 2.16 times (95% CI = 1.79–2.62).
Lozano *et al*. [63] 2017	Mexico (Original dataset)	Serious	Ever use	=*	Source: A school‐based, longitudinal survey of 60 public middle schools from 2015 (baseline) to 2016 (follow‐up). Findings: ‘Non‐smoking participants who had tried e‐cigarettes at baseline were more likely than those who had not to try conventional cigarettes (43% vs. 24%, respectively; RR = 1.41, 95% CI = 1.18–1.70) at follow‐up.’
Melka *et al*. [64] 2021	Australia (Australian Longitudinal Study on Women's Health)	Serious	Ever use	=*	Source: Wave 3 (2015) and wave 4 (2016) of the Australian Longitudinal Study on Women's Health project. Findings: ‘The odds of subsequent smoking initiation were 3.7 times higher among baseline survey ever e‐cigarette users compared to never e‐cigarette users (aOR = 3.71, 95% CI = 2.33, 5.93).’
Owotomo *et al*. [65] 2020	USA (PATH)	Serious	Ever use	=*	Source: Wave 2 (2014–2015) and wave 3 (2015–2016) of the PATH. Findings: Ever e‐cigarette use at wave 2 was positively associated with ever smoking at wave 3 (aOR = 2.58; 95% CI = 1.73–3.85; *P* < 0.001). ‘The interaction of smoking intention and ever using e‐cigarettes was significant (aOR = 0.34; 95% CI = 0.18–0.64; *P* = 0.01), suggesting the association between e‐cigarette use and ever smoking was dependent on previous smoking intention status. Among adolescents who intended to smoke conventional cigarettes at wave 2, e‐cigarette use was not significantly associated with ever smoking at wave 3 (aOR = 1.57; 95% CI = 0.94–2.63; *P* = 0.08). Among those without intention to smoke at wave 2, e‐cigarette users had 4 times higher odds of smoking at wave 3 than never e‐cigarette users (aOR = 4.62; 95% CI = 2.87–7.42; *P* = 0.0001).’
Staff *et al*. [66] 2022	UK (MCS)	Serious	Ever use	=*	Source: Data collected in infancy from 2002 at ages 3, 5, 7 and in youth at ages 11 (2012–2013), 14 (2015–2016) and 17 (2018–2019) from the MCS. Findings: ‘Odds of ever smoking by age 17 were more than five times higher among youth who had used e‐cigarettes by age 14 compared to teens who had not (OR = 5.31; 95% CI = 3.27–8.62). After adjustment for confounders and demographics, the odds of smoking by age 17 remained more than five times higher among early e‐cigarette users (OR = 5.25; 95% CI = 3.28–8.38).’ ‘Similarly, the odds of transitioning from being a never smoker at 14 to a frequent tobacco user at age 17 were more than three times higher (OR = 3.59; 95% CI = 2.04–6.33) among youth who had used e‐cigarettes by age 14. The odds reduced in magnitude after adjusting for the measured confounders, but e‐cigarette users by 14 still had nearly three times higher odds (OR = 2.91; 95% CI = 1.56–5.41).’
Stanton *et al*. [54] 2019	USA (PATH)	Serious	Ever use	=*	Source: 2013–2014 (wave 1) and 2014–2015 (wave 2) waves of the PATH study to examine bidirectional associations between ENDS use and cigarette use among 12‐ to 17‐year‐olds. Findings: The authors reported that ‘cigarette‐naive ever‐ENDS users at wave 1 were more than 4 times more likely to exhibit new ever‐cigarette smoking at wave 2 compared with ENDS‐naive youth at wave 1 (*n* = 78, 19.2%, 95% CI = 15.0,24.1 vs. *n* = 390, 4.0%, 95% CI = 3.5, 4.4)’. Compared with never ENDS use, ever ENDS use among cigarette‐naïve youth at wave 1 was significantly associated with ever‐cigarette smoking at wave 2 (OR = 3.21, 95% CI = 1.95, 5.45, *P* < 0.001) and for ever, but no past 30‐day ENDS use compared with never use (OR = 3.67, 95% CI = 2.03, 6.98, *P* < 0.001).
Stokes *et al*. [67] 2021	USA (PATH)	Serious	Ever use	=*	Source: PATH (2013–2018) Findings: ‘Ever e‐cigarette use (OR = 2.76; 95% CI = 2.21–3.45) [was] significantly associated with increased odds of cigarette initiation over 1‐year of follow‐up compared with never users. Additionally, the odds of past‐30‐day use were higher among youth with prior e‐cigarette use (OR = 2.72; 95% CI = 2.00–3.68)…compared with never users.’
Sun *et al*. [68] 2022	USA	Serious	Ever use	=*	Source: Waves 1–2 (2013–2015), 2–3 (2014–2016), 3–4 (2015–2017), 4–4.5 (2016–2018), and 4.5–5 (2017–2019) of the PATH Study. Findings: ‘Across all four models, ever e‐cigarette use is positively associated with subsequent cigarette smoking, but as we move from model 1 (includes socio‐demographic factors) to model 4 (includes socio‐demographic factors, exposure to tobacco users, cigarette susceptibility and behavioural risk factors) the aOR becomes successively smaller across all waves and is non‐significant (at the 5% level) in model 4 for the two most recent wave comparisons, waves 4–4.5 and waves 4.5–5. For waves 1–2, the aOR decreases from 5.55 (95% CI = 3.87–7.97) in model 1 to 2.09 (95% CI = 1.26–3.48) in model 4; for waves 2–3, 5.93 (95% CI = 4.07–8.63) to 2.10 (95% CI = 1.33–3.30); for waves 3–4, 5.53 (95% CI = 4.11–7.44) to 2.25 (95% CI = 1.55–3.27); for waves 4–4.5, 4.96 (95% CI = 3.66–6.72) to 1.40 (95% CI = 0.91–2.14); and for waves 4.5–5, 4.07 (95% CI = 2.86–5.81) to 1.35 (95% CI = 0.84–2.16). In models comparing the association between ever e‐cigarette use and past 30‐day cigarette smoking the aOR ‘declines steadily and substantially from model 1 to model 4. Except for waves 3–4, in which the aOR is significant at 2.16 (95% CI = 1.18–3.97), the model 4 aORs are all non‐significant. Specifically, the non‐significant aORs in model 4 for the other wave comparisons are: 1.41 (95% CI = 0.64–3.09) in waves 1–2, 1.41 (95% CI = 0.67–2.98) in waves 2–3, 1.11 (95% CI = 0.57–2.16) in waves 4–4.5, and 1.21 (95% CI = 0.59–2.48) in waves 4.5–5.’
Watkins *et al*. [55] 2018	USA (PATH)	Serious	Ever use	=*	Source: Wave 1 (2013–2014) and wave 2 (2014–2015) of the PATH study to assess the longitudinal association between e‐cigarette use (and other non‐cigarette tobacco use) and cigarette smoking initiation in US youth. Findings: Among never smokers at baseline, the adjusted odds for ever cigarette use at wave 2 were higher in ever e‐cigarette users compared with never users (OR = 2.53; 95% CI = 1.80, 3.56). Similarly, the odds of past 30‐day use of cigarettes at wave 2 were higher in ever users of e‐cigarettes compared with never users at wave 1 (OR = 1.87; 95% CI = 1.15, 3.05).
Xu *et al*. [69] 2022	USA	Serious	Ever use	=*	Source: First three waves of the PATH study 2013–2014 to 2015–2016). Findings: ‘Results from weighted logistic regression indicated a positive association between prior e‐cigarette use and subsequent combustible cigarette initiation, OR = 3.42, 95% CI = (1.99, 5.93), and P30D combustible cigarette use, OR = 2.88, 95% CI = (1.22, 6.86), in the following year’

*Notes*: Exposure: ever e‐cigarette use; outcome: smoking initiation at follow‐up (=* statistically significant direct association; = direct association, not statistically significant). Tier 1 studies (each study had 5000 + participants).

Abbreviations; aOR, adjusted odds ratio; CHS, California Children's Health Study; CLCC, cigar, little cigar or cigarillo; H&H, Happiness and Health Study; MCS, Millennium Cohort Study; PATH, Population Assessment of Tobacco and Health; P30D, past 30‐day; Project M‐PACT, Marketing and Promotions across Colleges in Texas project; TAALS, Taiwan Adolescent to Adult Longitudinal Study; TLC, Truth Longitudinal Cohort; US, United States; USA, United States of America; YASS, Yale Adolescent Survey Study.

One further Tier 1 study, Sumbe *et al*. [70], looked at whether EC type was associated with smoking initiation. They reported that the odds of initiating combustible tobacco use after EC initiation were statistically significantly lower among those who reported using cartridges as their initial device type, compared with refillable devices. They reported no significant differences when comparing disposables to other device types. Tier 2 studies are reported in Data S2.

### Progression

All studies examining this outcome evaluated EC use as the exposure. Overall, studies suggested vaping was associated with subsequent smoking progression, although data were slightly more varied than for initiation measures.

Three Tier 1 studies (two using PATH data) reported statistically significant direct associations between current vaping and subsequent smoking progression, although in one of these the finding was no longer statistically significant at 2 years (Table 5). Two Tier 1 studies evaluated associations between ever use of e‐cigarettes and smoking progression. One found a non‐statistically significant direct association, the other a statistically significant direct association (Table 6). Tier 2 studies are reported in Data S2.

**TABLE 5:** Association direction plot.

Study ID	Country (dataset)	Risk of bias overall	Exposure	Association direction	Brief summary of contributing data
Loukas et al. [33] 2022	USA (PROJECT M‐PACT)	serious	Past 30‐day use	=*	Source: Project M‐PACT, a longitudinal study spanning a 4.5‐year period from 2014–2019. Findings: After adjusting for the covariates, current ENDS use increased the probability of transitioning from non‐current to current cigarette use (re‐uptake) by 1.92 times (95% CI = 1.50–2.45). Note: non‐current smokers were defined as ever smokers who did not use cigarettes in the past 30 days.
Osibogun et al. [71] 2020	USA (PATH)	serious	Past 30‐day use	=* (for 1‐year progression) = (for 2‐year progression)	Source: Waves 1–3 of the PATH (2013–2016) Findings: ‘5.3% (95% CI = 3.1%, 8.9%) of current e‐cigarette users at wave 1 and 2 reported regular cigarette smoking at the 1‐year progression compared with 0.3% (95% CI = 0.2%, 0.5%) among non‐current e‐cigarette users (*P* < 0.0001). In the 2‐year progression, 8.2% (95% CI = 3.3%, 19.1%) of current e‐cigarette users identified at wave 1 reported regular cigarette smoking 2 years later compared with 0.8% (95% CI = 0.6%, 1.1%) among non‐current e‐cigarette users (*P* < 0.0001).’ Current e‐cigarette users were at 5.0 (95% CI = 1.9, 12.8) times higher odds of regular cigarette smoking in the 1‐year progression model compared with non‐current e‐cigarette users. In the 2‐year progression model, current e‐cigarette users had 3.4 (95% CI = 1.0, 11.5) times the odds of regular cigarette use compared with non‐current e‐cigarette users, although not statistically significant.
Sun *et al*. [72] 2023	USA (PATH)	Serious	Past 30‐day use	=*	Source: Waves 3 (2015–2016), wave 4 (2016–2018) and wave 5 (2018–2019) of the PATH study. Finding: In the models that assessed any use of cigarettes in the past 30 days, among adolescents who had used e‐cigarettes in the past 30‐days in wave 3, 9.4% (95% CI = 5.1%–16.8%) initiated and continued smoking compared with 1.4% (95% CI = 1.2%–1.7%) among never e‐cigarette users (*P* < 0.0001). The aOR was 2.66 (95% CI = 1.07–6.63) for current use of e‐cigarettes. ‘Baseline current e‐cigarette use was associated with a 1.88 percentage points increase (95% CI = −0.66 to 4.41 percentage points) in continued smoking, from 1.30% (95% CI = 0.90%–1.70%) among non‐current e‐cigarette users to 3.18% (95% CI = 0.57%–5.79%) among current users.’ In models that assessed established use of cigarettes, 7.4% (95% CI = 3.6%–14.6%) of past 30 ‐day e‐cigarette users continued smoking, compared with 0.7% (95% CI = 0.5%–0.9%; *P* < 0.001) of never e‐cigarette users. The aOR was 4.59 (95% CI = 1.39–15.16) for current use of e‐cigarettes in this model.

*Notes*: =* statistically significant direct association; = direct association, not statistically significant. Tier 1 studies.

Abbreviations: aOR, adjusted odds ratio; PATH, Population Assessment of Tobacco and Health Study; Project M‐PACT, Marketing and Promotions across Colleges in Texas project.

**TABLE 6:** Association direction plot. Exposure: ever e‐cigarette use; Outcome: smoking progression at follow‐up.

Study ID	Country (dataset)	Risk of bias overall	Exposure	Association direction	Brief summary of contributing data
Loukas et al. 2022 [33]	USA (PROJECT M‐PACT)	Serious	Ever use	=	Source: Project M‐PACT, a longitudinal study spanning a 4.5‐year period from 2014–2019. Findings: After adjusting for the covariates, ever ENDS use ‘did not impact transitions from non‐current to current cigarette use (re‐uptake)’ (HR = 1.14, 95% CI = 0.92–1.40). Note: non‐current smokers were defined as ever smokers who did not use cigarettes in the past 30 days.
Pierce et al. 2021 [34]	USA (PATH)	Critical	Ever use	=*	Source: Waves 1–4 (2013–2017) of the PATH. Finding: ‘Ever use of an e‐cigarette (vs. never use) increased the risk of later daily cigarette smoking by threefold (3% vs 10%; adjusted risk difference [aRD] 7%; 95% CI = 6% to 9%), adjusted for confounders.’

*Notes*: =* statistically significant direct association; = direct association, not statistically significant. Tier 1 studies.

Abbreviations: HR, hazard ratio; PATH, Population Assessment of Tobacco and Health Study; Project M‐PACT Marketing and Promotions across Colleges in Texas project.

### Cessation

Overall, fewer studies looked at cessation than at initiation or progression, and of those that did, data were mixed with no clear patterns emerging. This may be in part because of some, but not all studies differentiating between people using EC to quit smoking and people who reported using EC, but not to quit smoking.

One study evaluated associations between EC availability and cessation of cigarette smoking. Nguyen and Bornstein [43] investigated the association between banning EC use in public places and workplaces and cigarette use. Coefficients were negative and statistically non‐significant in analyses of the impact of EC bans on EC use for smoking cessation in current smokers.

Two Tier 1 studies provided data on associations between current EC use and subsequent smoking cessation. Loukas *et al*. [33] was the only one for which an association could be categorized. In this study, authors report that current EC use ‘decreased the probability of transitioning from current to non‐current cigarette use (desistance) by 1.59 times’. One further Tier 1 study provided data on cessation, Glantz [73] used US data, focusing on youth who started smoking before starting EC use, and reported that using ECs to quit smoking was associated with a statistically significantly lower odds of having stopped smoking relative to youth who did not report use of ECs as a quit aid. Tier 2 studies are reported in Data S2.

Three Tier 1 studies evaluated associations between ever vaping and subsequent smoking cessation (Table 7). One found vaping was non‐statistically significantly associated with greater smoking cessation at follow up, and the other two found vaping was statistically significantly associated with less smoking cessation at follow‐up. Tier 2 studies are reported in Data S2. Results of associations for other measures of smoking behaviour can be found in Data S2.

**TABLE 7:** Association direction plot.

Study ID	Country (dataset)	Risk of bias overall	Exposure	Association direction	Brief summary of contributing data
Friedman and Xu [60] 2020	USA (PATH)	Serious	Ever use	X (vaping associated with more smoking cessation)	Source: Wave 1–4 (2013–2018) of the PATH study. Findings: ‘For individuals who smoked at baseline, vaping was associated with increased cessation among prime‐age adults (aOR = 1.40; 95% CI = 1.01–1.96; *P* = 0.046). Although the aOR was not statistically significant for emerging adults (aOR = 1.22; 95% CI = 0.80–1.86; *P* = 0.36), it was significant in the pooled analyses for those aged 18 to 54 years (aOR = 1.34; 95% CI = 1.02–1.75; *P* = 0.03). Both findings became insignificant when wave 4 cessation was considered, although unweighted regressions yielded prime‐age findings that were significant for cessation at both wave 3 (aOR = 1.49; 95% CI = 1.11–2.00; *P* = 0.01) and wave 4 (aOR = 1.38; 95% CI = 1.02–1.87; *P* = 0.04)’. Note: We focus here on ‘emerging adults’ who were age 18–24‐years rather than prime age adults (25–54 years) who do not fit the criteria for this review.
Glantz [73] 2023	USA (NYTS)	Critical	Ever use	=* (vaping associated with less smoking cessation)	Source: NYTS from 2015 to 2021 Findings: ‘Among ever‐smoking youth who started using e‐cigarettes after they started using cigarettes, the aOR of having stopped smoking cigarettes associated with using e‐cigarettes to quit was 0.62 (95% CI = 0.45,0.85; *P* = 0.003), controlling for level of dependence, year, age, gender, and race/ethnicity…The odds of stopping cigarettes associated with using e‐cigarettes to quit were stable over time: in an additional analysis (not shown) the interaction between using e‐cigarettes to quit and year (centered) was not significant (*P* = 0.382).’
Loukas *et al*. [33] 2022	USA (PROJECT M‐PACT)	Serious	Past 30‐day	=* (vaping associated with less smoking cessation)	Source: Project M‐PACT, a longitudinal study spanning a 4.5‐year period from 2014–2019. Findings: After adjusting for covariates, ever ENDS use ‘decreased the probability of transitioning from current to non‐current cigarette use (desistance) by 1.85 times.’ (HR = 0.54, 95% CI = 0.47, 0.)

*Notes*: Exposure: ever e‐cigarette use; outcome: smoking cessation at follow‐up (=* statistically significant direct association; = direct association, not statistically significant; X inverse association, not statistically significant). Tier 1 studies.

Abbreviations: aOR, adjusted odds ratio; HR, hazard ratio; PATH, Population Assessment of Tobacco and Health Project; M‐PACT Marketing and Promotions across Colleges in Texas project; NYTS, National Youth Tobacco Surveys.

### Differences based on socio‐demographic characteristics

Although many studies controlled for PROGRESS‐Plus characteristics in their analyses, few reported whether associations differed within subgroups. Of those that did, no clear patterns emerged for rurality, race/ethnicity, income, education or age. Although there was no evidence of a difference at the population level, individual‐level studies suggested vaping was more strongly associated with subsequent smoking in males than females. Seven of the nine individual‐level studies that examined associations based on measures related to susceptibility to smoking found that the associations were stronger in those with lowest susceptibility at baseline, whereas the other two found the opposite. No population‐level studies provided a breakdown by this category. No studies reported associations broken down by other PROGRESS‐Plus categories. More detail can be found in Data S2.

## DISCUSSION

Overall, the certainty of the evidence was judged to be very low (Table 8). The evidence‐base in this space has critical limitations and contradictions. The only thing that can be said with certainty is that the evidence is not certain. Population‐level studies had lower risk of bias than individual‐level studies, with several of these population‐level studies reaching the category of ‘moderate’ risk of bias, missing ‘low’ designation only on account of not using a pre‐approved study protocol. None of the individual‐level studies reached the ‘moderate’ designation. Previous systematic reviews of cohort studies were suggestive of a positive association between vaping and later smoking, but none were able to establish a causal relationship and they did not focus on evidence emerging from primary population‐level studies [8, 74, 75, 76, 77, 78].

**TABLE 8:** Electronic cigarettes and subsequent smoking in young people.

Population: people aged 29 and younger			
Setting: various			
Exposure: e‐cigarette use or availability			
Outcomes	Direction of association	No. of studies^a^ ^,^ ^b^	Certainty of the evidence^c,j^
Population rate of combusted tobacco use	Inverse association; e‐cigarette use/availability associated with less combustible tobacco use than would be otherwise expected	21	⊕⊝⊝⊝ VERY LOW^c^ ^,^ ^d^
Initiation of cigarette smoking	Direct association; e‐cigarette use was positively associated with subsequent initiation of combustible tobacco use	28	⊕⊝⊝⊝ VERY LOW^e^
Progression of cigarette smoking	Direct association; e‐cigarette use was positively associated with subsequent progression of combustible tobacco use	5	⊕⊝⊝⊝ VERY LOW^e^
Cessation of cigarette smoking	Inconclusive. One study using ‘current use’ as an exposure and two using ‘ever use’ as an exposure found a statistically significant decrease in smoking cessation in people vaping at baseline; one found a non‐statistically significant increase in cessation associated with ever use.^f^	4	⊕⊝⊝⊝ VERY LOW^e^ ^,^ ^g^

Notes: GRADE Working Group grades of evidence. High certainty: we are very confident that the true effect lies close to that of the estimate of the effect. Moderate certainty: we are moderately confident in the effect estimate: the true effect is likely to be close to the estimate of the effect, but there is a possibility that it is substantially different. Low certainty: our confidence in the effect estimate is limited: the true effect may be substantially different from the estimate of the effect. Very low certainty: we have very little confidence in the effect estimate: the true effect is likely to be substantially different from the estimate of effect.

^a^ We do not provide a number of participants as some of the included studies do not state the number of participants eligible for contributing analyses, and as some of the studies use overlapping datasets.

^b^ Conclusions for initiation, progression and cessation are based on studies with *n* >5000; this is the number of studies we report in this column (these are the studies, which we pre‐specified would be prioritized in our analyses, and for which we conducted risk of bias assessments; data from studies with <5000 participants were consistent with the findings reported here unless otherwise indicated).

^c^ Downgraded one level for risk of bias; all studies judged to be at moderate, serious, or critical risk of bias.

^d^ Downgraded one level for inconsistency; association directions varied across studies and we were unable to identify the underlying causes of variation (although risk of bias was one).

^e^ Downgraded two levels for risk of bias; all studies were judged to be at serious or critical risk of bias.

^f^ Data from studies in <5000 participants were also mixed: two found statistically significant associations between vaping and increased smoking cessation at follow‐up; two found statistically significant associations between vaping and decreased smoking cessation; two found no evidence of an association; one found a non‐statistically significant association with increased smoking cessation; and two found a non‐statistically significant association between vaping and decreased smoking cessation.

^g^ Downgraded two levels because of inconsistency; findings mixed across studies with no clear pattern.

^h^ By virtue of all studies being based on quasi‐experimental and observational analyses, the GRADE starts at low, and can be upgraded or downgraded accordingly.

^i^ GRADE guidance specifies that studies using observational data begin at low certainty, can be downgraded for the same reasons as apply to randomized controlled trials, and can be upgraded based on magnitude of effect, plausible confounding in the opposite direction of the demonstrated association, and evidence of dose–response gradients [18]. We did not upgrade any of our outcomes based on these—plausible unmeasured confounding (for example, measures related to propensity to smoke) is more likely to amplify the demonstrated effect; there was no consistent evidence of dose–response gradients; and magnitude of effect varied both across and within studies depending on which variables were controlled for—whereas some studies reported large unadjusted effect sizes, magnitudes often decreased as additional variables were controlled for. When grading, we were not considering whether an association existed, but whether such an association represented a true effect (i.e. was causal).

At a population level, evidence is mixed, but on balance suggests an inverse relationship between EC use or availability and smoking in young people—in other words, as ECs become more available and their use in young people increases, smoking rates decrease more than would have been expected otherwise. Similarly, as ECs become less available and their use in young people declines, smoking rates are higher than would have been expected otherwise. This evidence is consistent with more young people never starting to smoke to begin with, or quitting smoking, as a result of ECs rather than initiating via a gateway‐style relationship, at this point in time. However, findings are not consistent across all studies contributing data, and many are judged to have serious limitations.

In the population studies that did detect a relationship between EC use or availability and smoking, the effect size in terms of the change in population‐level smoking prevalence was typically of the order of one to two percentage points. As this constitutes only a small fraction of the overall prevalence of smoking among young people, the impact of EC policies on smoking rates appears not to have been transformational. Nevertheless, even apparently small percentage changes such as this may have a large societal and health impact as they may represent many thousands of individuals when applied across large population groups.

At an individual level, data demonstrates that vaping is positively associated with smoking initiation and progression. Our judgement of very low certainty for this outcome does not reflect whether an association exists, but whether this association is causal. The impact of vaping on smoking cessation in young people is also unclear, with fewer individual studies contributing data, and mixed results across those which do. Evidence from randomised controlled trials shows that e‐cigarettes with nicotine are an effective smoking cessation aid [79], but other effective smoking cessation aids (e.g. nicotine replacement therapy) appear to work less well in adolescents than adults [80]. There is limited evidence around the long‐term smoking patterns of individuals who progressed from vaping to smoking at a young age, with some studies suggesting frequent transitions between tobacco and nicotine products among young people. It is possible that some individual‐level studies in this review could be capturing one element of a larger trend of episodic behaviours. It is important to triangulate this information with population‐level data to capture effects on sustained population‐level use [81, 82]. Determinants at individual and population levels could also differ [83].

The certainty of our primary outcome, association between EC use or availability and population rate of combustible tobacco use, was downgraded one level because of risk of bias—all studies were judged to be at least at moderate risk of bias, with the main reason being that analyses were not pre‐registered or specified. Certainty was also downgraded because of inconsistency, and differences between the studies could not be fully explained by variables investigated in our QCA. There was, however, a noticeable pattern, whereby the lower risk of bias studies were those most likely to detect an inverse association, and the higher risk of bias studies were most likely to detect a direct association. Risk of bias ratings were done independently according to a detailed, pre‐specified tool and were not impacted by the findings of the studies.

For our secondary outcomes—initiation, progression and cessation—certainty was again very low. All of these outcomes were downgraded, as all studies contributing data were judged to be at serious or critical risk of bias. Certainty in our cessation outcome was further limited by unexplained heterogeneity in findings across the studies.

Given risk of bias is the main limitation of the evidence overall, it is worth noting that the tool we used, although strict, does not rule out the possibility of a low risk of bias study. The only risk of bias detected for some population‐level (quasi‐experimental) studies was a lack of pre‐registration, which can introduce bias because of selective reporting. These studies were judged to be at low risk of bias in all other domains because of their attempts to mimic randomization by using plausibly exogenous changes in e‐cigarette policies or introductions. Our individual‐level studies for the most part did not set out to do this, and confounding is and will continue to be difficult to rule out in these designs. However, methods such as Mendelian randomization, or use of other instrumental variables, conceivably could provide a path toward low risk of bias individual‐level studies using our tool.

Data overwhelmingly came from a small number of high‐income countries. This is a further critical limitation to the evidence base. Different countries have different regulations and enforcement processes in place for vaping and smoking, and different rates of smoking and vaping among youth. These may affect the relationship between vaping and smoking, and so conclusions cannot be extrapolated to lower‐income countries or even to other higher‐income countries that have different cultural or regulatory environments. More research is needed outside of the United States. We stratified individual‐level studies by sample size, but findings were consistent across both groups of studies, and both groups of studies predominantly represented data from the same countries.

Many studies did not report whether associations differed based on socially stratifying characteristics, so there is uncertainty as to whether the impact of EC availability on smoking applies equally in all population subgroups. The data we do have suggest that, at an individual level, direct associations between vaping and subsequent smoking may be more pronounced in males than females and in groups judged to have the lowest susceptibility to smoking at baseline.

Although relatively few studies investigated the effect of EC on smoking cessation or reduction, this is the one area in which randomization to ECs may be judged appropriate—there is now a strong body of evidence from randomized controlled trials showing that giving ECs to adult tobacco smokers can help them quit smoking [79]. Most of these studies to date have not been restricted to young people, therefore, are not eligible for our review, but conceivably such a study could be conducted in the future (one study testing e‐cigarettes for smoking reduction in young people is currently underway [84, 85]). Most other outcomes considered in this review would be unsuitable for a randomized trial design, because it would be unethical to randomize non‐smoking young people to start vaping.

Conducting this review posed many challenges. As it investigates an exposure as opposed to an intervention, in some cases we could not follow standard Cochrane methods, and the varied study designs included eventually precluded this review from being published as a Cochrane review, as was originally intended. This particularly applies to coding of outcome data, and to risk of bias assessment —there was no agreed tool for assessing risk of bias in reviews of exposures when we started this review, and hence, we adapted an external tool that was recommended by Cochrane. A different tool may have yielded different results, particularly for our population‐level studies. However, even if we had used a different risk of bias tool, our findings on effect direction would be unchanged and certainty would still be limited by the fact these were observational studies, and in some cases, unexplained heterogeneity was present.

The lack of pre‐registration as a norm for observational and quasi‐experimental studies also means we are unable to rule out publication bias. We tried to mitigate this by searching grey literature and contacting experts in the field. We may have also missed some relevant studies published in economics journals—we know of two missed because of indexing in economic journals not being as consistent as that for traditional medical journals (these two has been incorporated, but there may be others). Nonetheless, our search strategy followed Cochrane best practice for medical research.

Several studies used data that originated from the same surveys, so there may have been partial overlap in the individuals and time periods that made up the datasets used in these studies. It is possible that this may have made some study results more similar than would have been the case if they had all used fully independent data sources.

We had to adjust our analysis plan substantially between the protocol and the review, because of the quantity and quality of the available literature. We attempted to minimise bias in amending our methods through agreeing to all changes as an author team and pre‐registering these changes on Open Science Framework. Further methods development and consensus would undoubtedly help future systematic reviews in this area.

## CONCLUSIONS

Data are very low certainty for all outcomes. This means we think future studies are very likely to change our conclusions. At a population level, the balance of evidence suggests that overall, youth vaping and smoking are inversely related—that is, as more young people vape, fewer smoke, and vice versa. However, this could vary by context and was not consistent across all studies. There is insufficient information to say if this varies based on socio‐demographic characteristics of individuals.

Data showed a clear association between vaping and subsequent smoking initiation and progression in individuals, that is, young people who vape are more likely to progress to smoking. However, it is unclear whether these patterns in individuals reflect a causal relationship. On balance, population‐level data show smoking rates decline as vaping rates go up. Patterns in individuals may be driven by underlying factors, which are often not considered in analyses. Further research establishing causal relationships between vaping and later smoking in young people, at both individual and population‐level, is needed.

## AUTHOR CONTRIBUTIONS

**Rachna Begh:** Data curation; formal analysis; investigation; methodology; project administration; resources; validation; writing—original draft. **Monserrat Conde:** Data curation; formal analysis; investigation; methodology; project administration; resources; validation; writing—original draft. **Thomas R. Fanshawe:** Formal analysis; investigation; methodology; validation; writing—original draft. **Dylan Kneale:** Formal analysis; investigation; methodology; validation; writing—original draft. **Lion Shahab:** Methodology; validation; writing—original draft. **Sufen Zhu:** Data curation; formal analysis; investigation; validation; writing—original draft. **Michael Pesko:** Methodology; validation; writing—original draft. **Jonathan Livingstone‐Banks:** Data curation; validation; writing—original draft. **Nicola Lindson:** Funding acquisition; methodology; writing—original draft. **Nancy A. Rigotti:** Methodology; writing—original draft. **Kate Tudor:** Data curation; methodology; validation; writing—original draft. **Dimitra Kale:** Validation; writing—original draft. **Sarah E. Jackson:** Validation; writing—original draft. **Karen Rees:** Validation; writing—original draft. **Jamie Hartmann‐Boyce:** Conceptualization; data curation; formal analysis; funding acquisition; methodology; project administration; resources; supervision; validation; writing—original draft.

## DECLARATION OF INTERESTS

M.C.'s work is funded by Cancer Research UK under grant number PPRCTAGPJT\10 000. This was not deemed a conflict of interest. R.B., J.H., S.Z., R.N., J.L.B., K.R. and K.T. declare no competing interests. D.K. is an Associate Editor for Addiction and has no competing interests to declare. J.H.B. declares current funding from the Food and Drug Administration and the Truth Initiative on topics related to tobacco control and e‐cigarettes. C.N. is an Associate Editor at Addiction and the first author of an included study. C.N. was not involved in the screening, coding or quality appraisal of this study. N.L. is an Associate Editor for Addiction and has no competing interests to declare. S.C. is a Senior Editor at Addiction and the co‐author of an included study. S.C. was not involved in the screening, coding or quality appraisal of this study. S.J. is a Senior Editor at Addiction. L.S. is a HEFCE funded member of staff at University College London. He has received honoraria for talks, an unrestricted research grant and travel expenses to attend meetings and workshops from Pfizer and an honorarium to sit on advisory panel from Johnson and Johnson, both pharmaceutical companies that make smoking cessation products. He has acted as paid reviewer for grant awarding bodies and as a paid consultant for health care companies. Other research has been funded by the Department of Health, UKRI, a community‐interested company (National Centre for Smoking Cessation) and charitable sources (Cancer Research UK, Yorkshire Cancer Research). He has never received personal fees or research funding of any kind from alcohol, electronic cigarette or tobacco companies. L.S. is the first author of an included study and was not involved in screening, data extraction, or appraisal of this study. M.P.'s research is supported by the National Institute on Drug Abuse of the National Institutes of Health under award number R01DA045016. M.P. declares current funding from the Food and Drug Administration, the American Cancer Society, University of Kentucky Institute for the Study of Free Enterprise, and Health Canada. M.P. is the first author and co‐author of included studies and was not involved in the screening, data extraction, or appraisal of these studies. T.R.F. receives funding from the National Institute for Health and care Research (NIHR) HealthTech Research Centre for Community Healthcare at Oxford Health NHS Foundation Trust and the NIHR Applied Research Collaboration Oxford and Thames Valley at Oxford Health NHS Foundation Trust. The views expressed are those of the authors and not necessarily those of the NHS, the NIHR or the Department of Health and Social Care.

## Supporting information


**Data S1.** Supporting Information.

**Data S2.** Supporting Information.

**Data S3.** Supporting Information.

**Data S4.** Supporting Information.

## Data Availability

The data that support the findings of this study are available from the corresponding author upon reasonable request. Most data are in the public domain as they are from published literature.
