# Supplementary material for: Electronic cigarettes and subsequent cigarette smoking in young people: A systematic review
Source: Addiction. 2025 Jan 30;120(6):1090–111. doi: 10.1111/add.16773 (PMC12046492; doi:10.1111/add.16773)
Supplement: Supplementary file 1 — Data S1. Supporting Information. [file ADD-120-1090-s003.pdf]

Supplementary materials 1

1. **Methods**

1.1 Search strategy

We searched the following databases up to 3rd April 2023:

- Cochrane Tobacco Addiction Group Specialized Register (CRS-Web 1st January 2004 to 3rd April 2023)
- MEDLINE (OVID SP; 1st January 2004 to 3rd April 2023)
- Embase (OVID SP; 1st January 2004 to 3rd April 2023)
- PsycINFO (OVID SP; 1st January 2004 to 3rd April 2023)

We searched all databases from 2004 onwards, as ECs were not available before then, and searched using free text and controlled vocabulary relating to ECs and child and young adult populations. Note that the Cochrane Tobacco Addiction Group Specialized Register ceased to be maintained in December 2022. At the time of our search, the Register included the results of searches of CENTRAL Issue 11, 2022; MEDLINE (via OVID) to update 20221222; Embase (via OVID) to week 202251; PsycINFO (via OVID) to update 20221219; ClinicalTrials.gov (via CENTRAL Issue 11, 2022); WHO International Clinical Trials Registry Platform (ICTRP: www.who.int/ictrp/en/, via CENTRAL Issue 11, 2022). See the Tobacco Addiction Group website for full search strategies and a list of other resources searched.

Our search strategy is listed below.

OVID Databases (MEDLINE, Embase, PsycINFO)

1. randomized controlled trial.pt. OR controlled clinical trial.pt. OR clinical trials as topic.sh. OR trial.ti.

2. (teen* or young* or youth* or child* or paed* or college* or school* or university* or pupil* or student* or minor* or adolescent* or uptake or gateway or commenc* or prevalence).mp.

3. e-cig*.mp. OR ecig*.mp. OR electr* cigar*.mp. OR electronic nicotine.mp. OR (vape or vapes or vaporizer or vapourizer or vaporiser or vapouriser or vaper or vapers or vaping).ti,ab. OR Exp Electronic Nicotine Delivery Systems/

4. (smok* or cigar* or tobacco or combust*).mp.

5. 2 and 3 and 4

6. 5 not 1

7. exp animals/ not human/

8. 6 not 7

Cochrane Tobacco Addiction Group Specialised Register (via CRS-Web)

1. (trial): TI

2. (teen* or young* or youth* or child* or paed* or college* or school* or university* or pupil* or student* or minor* or adolescent* or uptake or gateway or commenc* or prevalence): TI,AB,MH,EMT,KY,XKY

3. (e-cig* OR ecig* OR electr* cigar* OR electronic nicotine): TI, AB,MH,EMT,KY,XKY

4. (vape or vapes or vaporizer or vapourizer or vaporiser or vapouriser or vaper or vapers or vaping): TI, AB, MH, EMT, KY, XKY

5. MESH DESCRIPTOR Electronic Nicotine Delivery Systems EXPLODE ALL

6. (smok* or cigar* or tobacco or combust*): TI, AB,MH,EMT,KY,XKY

7. 3 or 4 or 5

8. 2 and 6 and 7

9. 8 not 1

1.2 Data extraction

Data extraction was conducted using a prespecified and piloted data extraction form. We extracted data for the following fields:

- Country
- Economic level of the country or countries
- World Health Organization Region (e.g., region of the Americas)
- Setting
- Study design
- Study dates
- Number of data timepoints
- Participants (population, number, mean age, age range, ethnicity, gender, measures of socioeconomic status, PROGRESS-Plus indicators (1), cigarette and EC use at baseline)
- Study methods (including confounders and how these were controlled for)
- Exposure(s), including EC use (e.g. ever use, past 30-day use) and availability (e.g. restrictions such as bans on use, bans on flavours, price changes)
- Comparators
- Covariates/confounders (PROGRESS-Plus indicators)
- Outcomes, including population rate of tobacco use in young people, uptake, progression or cessation of cigarette smoking (e.g. ever use, use during past six months, past 30-day use, daily use)
- Analysis methods
- Information needed to assess risk of bias (as detailed below)

- Study funding
- Conflicts of interest

1.3 Risk of Bias assessment

We adapted the risk of bias instrument for non-randomized studies (NRS) of exposures, developed by Morgan et al. (2019).(2)Members of the core review team (MC, KT, JHB, NL) met to adapt the tool to specificities of this complex review, namely, considering known risks of bias in individual-level and population-level studies looking into e-cigarettes and combustible tobacco use. After developing the first draft guidance, there was a meeting with two other reviewers (LS, MP) for their expert input on risk of bias due to confounding associated with different population-level study designs. A version integrating their input was circulated to the whole author team for review. Any further suggested elements were integrated into subsequent versions of the tool, until a final draft version was achieved. This version was tested by the core review team using a sample of included studies, with any resulting modifications being integrated into the final version of the tool. After this stage, risk of bias judgements were conducted independently and in duplicate by two reviewers (MC, KT, SZ, RB in teams of two), with discrepancies resolved by discussion or referral to a third review author (JHB, RB) for population-level studies and tier 1 individual-level studies.

1.4 Data synthesis

Heterogeneity in study designs, outcome measures, and exposures precluded meta-analysis. If, in the future, studies have the same designs and use similar outcome measures and exposure measures, we will consider combining them in meta-analysis using standard Cochrane methods.

Our initial plans for synthesis needed to be changed due to the nature of the available data. We agreed to these changes as an author team and registered our plans before starting analyses: https://osf.io/4wycq/. In this version of the review, we follow Cochrane guidance on synthesis without meta-analysis.(3) We had initially planned on using qualitative comparative analysis (QCA) and framework synthesis.(4,5) However, given the amount of data returned and the methodological heterogeneity amongst it, we made the decision - after full text screening but before data extraction - to use QCA and association direction plots (formatted as per effect direction plots), following Cochrane guidance.

1.4.1 Qualitative comparative analysis (QCA)

QCA is a method originating in the political sciences (see (6)) and has been increasingly used to understand how intervention processes and components combine to produce variations in outcomes within systematic review (see (7)). QCA aims to identify conditions (analogous to variables or factors e.g. vaping regulations, socioeconomic status characteristics) present when an outcome (e.g. youth smoking prevalence, progression to smoking) occurs within a case (a study). QCA draws on the mathematical principles of set theory to identify the extent to which different configurations of intervention characteristics trigger a given outcome through exploring set relationships. The full procedures for conducting a QCA as part of a systematic review are laid out elsewhere (see (7)), although briefly involve: (i) identification of theory; (ii) development of a data table of studies, outcomes and conditions that support exploration of the theory; (iii) the development of a truth table

that summarises how many cases within the review have a given configuration of conditions; (iv) checking the quality of the truth table; (v) undertaking Boolean minimisation to develop and simplify the solution; (vi) incorporating assumptions about logical remainders (unobserved configurations) to simplify the solution further; and (vii) further checks and interpretation of the solution. We originally planned to conduct three QCAs : a population-level QCA, an analysis methods QCA, and a predictor QCA, all of which we pre-registered: https://osf.io/nd3y4. However, we only conducted the population-level QCA, as the individual-level studies did not meet the requirements to perfom the analysis methods QCA and the predictor QCA (due to minimal variation in outcomes for initiation and progression, and insufficient studies for cessation).

A fundamental principle of conducting QCA is to ensure that analytical decisions are based on theory. In our QCA, our underlying theory is not based around psychosocial theories of why young people choose to vape or theories around social policy that explain why jurisdictions develop different policies. Instead, we aim to explain why some studies find that policies that liberalising/increasing access to electronic cigarettes/vapes leads to an increase in combustible tobacco consumption among young people, and others find that such policies lead to a decrease in combustible tobacco use. No cohesive theory from the public health literature was identified that could explain population level changes (although several exist at an individual level). Instead, we drew on knowledge from the team to consider the characteristics of the population, the features of the study (methods) and the type of policy (in this case the intervention), the comparator used, and the way of measuring the outcomes that could explain how policies appear to have contradictory influences. These roughly map onto 'PICOS' as a conceptual framework or form of 'theory'(8). For population characteristics we had intended to draw on PROGRESS-Plus(1)as a framework (for organising features of the population) although were limited in our capacity to do so in practice due to the poor reporting of population (and contextual) features. For the features of the studies and their methods, we focussed on elements that were specific to smoking and not on more general elements associated with risk of bias. Finally, we also drew on elements of context specific to our question through examining the level of smoking of combustible tobacco and EC use within different contexts to understand the level at which patterns of smoking were embedded among young people.

1.4.2 Association direction plots

We categorized the studies as showing a direct association, an inverse association, or no association, and within direct and inverse associations, as statistically significant or as not statistically significant/statistical significance not reported.

We categorized the following relationships as direct associations:

- EC use increases, smoking rates are higher than predicted
- EC become more available/accessible, smoking rates are higher than predicted
- EC use decreases, smoking rates are lower than predicted
- EC become less available/accessible, smoking rates are lower than predicted
- EC use is positively correlated with smoking uptake and/or progression
- EC use is negatively correlated with smoking reduction or cessation

We categorized the following relationships as inverse associations:

- EC use increases, smoking rates are lower than predicted
- EC become more available/accessible, smoking rates are lower than predicted
- EC use decreases, smoking rates are higher than predicted
- EC become less available/accessible, smoking rates are higher than predicted
- EC use is negatively correlated with smoking uptake and/or progression
- EC use is positively correlated with smoking reduction or cessation

We categorized the following as no association:

- An OR or RR of 1
- Authors reported 'no association' or 'no statistically signficant association' and did not provide further information with which to judge direction of association
- Authors reported 'no association' and the data provided were inconsistent regarding direction of effect

We categorized the following as statistically significant results:

- Where reported, p values <0.05, or 95% CI for OR or RR excluded 1, or CIs did not overlap where different estimates were given for different subgroups
- Authors stated 'statistical significance' but gave no other information

We categorized the following as 'not statistically significant'/'statistical significance not stated':

- Where reported, p values greater than or equal to 0.05, or 95% CI for RR or OR included 1, or CIs overlapped where different estimates were given for different subgroups
- Authors stated 'not statistically significant' but gave no other information
- Authors gave no information on statistical significance and statistical signficance cannot be derived from data directly

In each association direction plot, we provide a short summary of the supporting data that led to our categorization, including the effect size (such as a percentage point difference or odds ratio (OR)) and its precision, as reported by the study authors, where available. We also list the survey used, where relevant, to highlight potential overlap between datasets.

We examined study characteristics to assess the presence of clinical and methodological heterogeneity. As anticipated, methodological heterogeneity precluded traditional meta-analysis. We also considered the consistency of results across studies synthesised together and took this into account when carrying out GRADE assessments.

1.4.3 Investigation of heterogeneity and subgroup analysis

We used PROGRESS-Plus indicators (1)where reported in studies (e.g., socioeconomic status, gender, and other demographic factors) to assess whether the effects of vaping on risk of smoking differed across subgroups. These were selected because they are thought to be the most relevant factors when considering inequalities in

health.(1) Where sufficient studies reported this, these were included in our QCA. We also present summaries of directions of association for all PROGRESS-Plus indicators, grouped by indicator and within that by study type (population-level, individual-level tiers 1 and 2). We had also planned to tabulate studies based on the relevant covariates included in the analyses, in order to identify any associations between covariates and the outcomes of those analyses. This included definitions of EC and conventional cigarette use (e.g., ever use, past 30-day use, daily use, etc.), however, instead we capture these in the association direction plots and in the QCA. As PROGRESS-Plus indicators were so infrequently reported, we do not include these in association direction plots but instead report them in the text.

1.3.3 Sensitivity analysis

We explored the degree to which findings were sensitive to definitions used for both EC and conventional cigarette use via QCA and in our association direction plots.

References:

1. O'Neill J, Tabish H, Welch V, Petticrew M, Pottie K, Clarke M, et al. Applying an equity lens to interventions: Using PROGRESS ensures consideration of socially stratifying factors to illuminate inequities in health. J Clin Epidemiol. 2014;67(1).

2. Morgan RL, Thayer KA, Santesso N, Holloway AC, Blain R, Eftim SE, et al. A risk of bias instrument for non-randomized studies of exposures: A users' guide to its application in the context of GRADE. Environ Int [Internet]. 2019;122:168–84. Available from: https://www.sciencedirect.com/science/article/pii/S0160412018320853

3. Higgins P, Thomas J, Chandler J, Cumpston M, Li T, Page M, et al. Cochrane Handbook for Systematic Reviews of Interventions [Internet]. version 6.4. Cochrane; 2023. Available from: www.training.cochrane.org/handbook

4. Booth A, Carroll C. How to build up the actionable knowledge base: the role of 'best fit' framework synthesis for studies of improvement in healthcare. BMJ Quality & Safety [Internet]. 2015 Nov 1;24(11):700. Available from: http://qualitysafety.bmj.com/content/24/11/700.abstract

5. Caren N, Panofsky A. TQCA: A Technique for Adding Temporality to Qualitative Comparative Analysis. Sociol Methods Res [Internet]. 2005 Nov 1;34(2):147–72. Available from: https://doi.org/10.1177/0049124105277197

6. Ragin CC. The Comparative Method: Moving Beyond Qualitative and Quantitative Strategies. University of California Press; 1987.

7. Thomas J, O'Mara-Eves A, Brunton G. Using qualitative comparative analysis (QCA) in systematic reviews of complex interventions: a worked example. Syst Rev [Internet]. 2014;3(1):67. Available from: https://doi.org/10.1186/2046-4053-3-67

8. Thomas J, KD, MJE, BSE and BS. Determining the scope of the review and the questions it will address. In: Cochrane handbook for systematic reviews of interventions. Wiley Blackwell; 2019. p. 13–31.
