# Supplementary material for: Electronic cigarettes and subsequent cigarette smoking in young people: A systematic review
Source: Addiction. 2025 Jan 30;120(6):1090–111. doi: 10.1111/add.16773 (PMC12046492; doi:10.1111/add.16773)
Supplement: Supplementary file 2 — Data S2. Supporting Information. [file ADD-120-1090-s004.pdf]

Supplementary materials 2

Results

1. Search yield

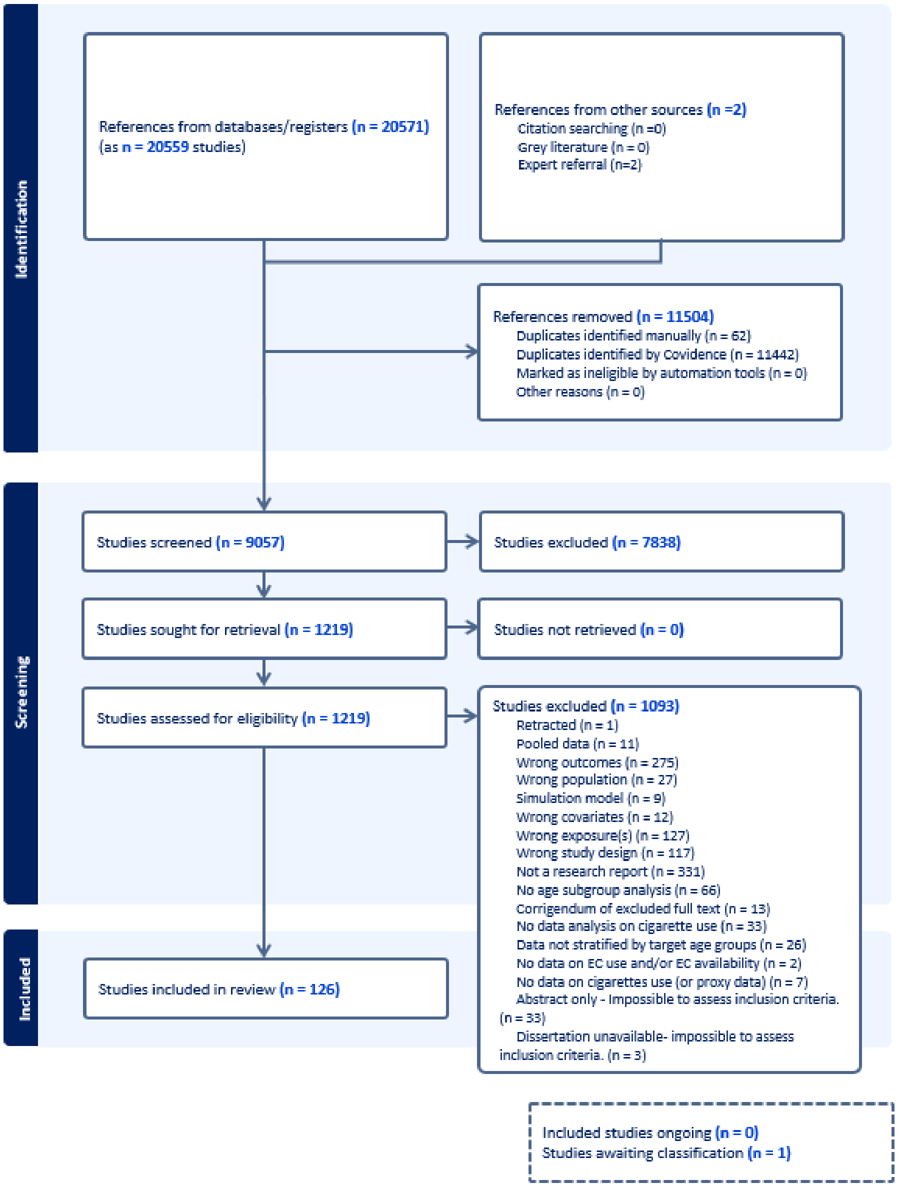

Figure 1. PRISMA flowchart

The study awaiting classification is Delnevo, C.D. and Villanti, A.C., 2023. Dramatic Reductions in Cigarette Smoking Prevalence among High School Youth from 1991 to 2022 Unlikely to Have Been Undermined by E-Cigarettes. International journal of environmental research and public health, 20(19), p.6866. It was published in September 2023.

2. Characteristics of studies

Most studies used datasets taken from state or national surveys; 29 from the Population Assessment of Tobacco and Health (PATH) study, eleven from the National Youth Tobacco Surveys (NYTS), five from the Truth Longitudinal Cohort (TLC), four each from the Southern California Children's Health Study (CHS) the COMPASS study, and the Monitoring the Future study (MTF), two from the Social and Emotional Contexts of Adolescent Smoking Patterns (SECASP) Study, two from the Taiwan Adolescent to Adult Longitudinal Study (TAALS), one using data from the Taiwan Adult Smoking Behaviour Survey and the Taiwan Global Youth Tobacco Survey (TGYTS) and three from the Youth Risk Behavior Surveillance System (YRBSS). Five studies used data from the Marketing and Promotions across Colleges in Texas project (Project M-PACT). Seven used a combination of datasets; two studies used data from the Southern California Children's Health Study (CHS), the Happiness and Health (H&H) Study and the Yale Adolescent Survey Study (YASS); one from the Canadian Tobacco, Alcohol and Drugs Survey (CTADS) and its predecessor, the Canadian Tobacco Use Monitoring Survey (CTUMS); one from the Smoking Drinking and Drug Use Among Young People in England Survey (SDDU), the Scottish Adolescent Lifestyle and Substance Use Survey (SALSUS), the Health Behaviour in School- aged Children (HBSC) survey, and the School Health Research Network (SHRN) survey; and Youth Risk Behavior Surveillance System (YRBSS); and one used CTADS, Action on Smoking and Health (ASH) and the National Drug Strategy Household Survey (NDSHS). Five studies derived data from cluster randomised controlled trials. The rest of the studies used data from various other longitudinal datasets or collected original data.

3. Risk of Bias

3.1 Risk of bias of population-level studies

Overall, we judged eleven population-level studies to be at moderate risk of bias, nine at serious risk of bias and seven at critical risk of bias. No studies were judged to be at low overall risk of bias.

Bias due to confounding

Thirteen of the population-level studies were judged to be at low risk of bias due to confounding. The fact that they were cross-context designs, including natural experiments, that met all judgment criteria supported this. Three were judged to be at moderate risk due to not testing for dose-response, seven at serious risk for being single context designs. Finally, four at critical risk of bias for reasons ranging from using self-reported and recall variables, not including state-level variables in their analysis to not including policy changes in the discussion.

Bias in selection of participants into the study

Twenty-one studies were judged to be at low risk of bias due to the selection of participants. Behind this judgment was the fact that the studies analyzed data derived from well-established sources. Four were deemed

at moderate risk for excluding participants with missing data and two at critical risk of bias for omitting dropouts from samples and using a non-anonymised convenience sample.

Bias due to misclassification of exposure

Twenty studies were at low risk of bias, Foxon 2020(1) at moderate risk for not reporting measures put at place to ensure anonymity of participants, three at serious risk for using a non-nationally representative sample or not specifying frequency of EC, and three at critical risk of bias for not specifying EC use or using a non-anonymized sample.

Bias due to deviations from intended exposures

Three studies were assessed as being at serious risk of bias for not discussing implementation of regulatory measures or discussing effects of measures on EC use, four critical for not considering concurrent policies/regulatory environment in their analyses and all the other studies were deemed to be at low risk of bias.

Bias in measurement of the outcome

Twenty- two studies were deemed to be at low risk of bias, two at serious risk of bias for not specifying frequency of EC use, Abouk 2017(2) and Gao 2020(3) at critical risk of bias due to omission of dropouts and for not specifying frequency of EC use, respectively.

Bias in selection of the reported results

All studies were judged to be at moderate risk of bias as protocols were not published or available, except for Shabab 2021(4) which was at low risk of bias. The authors published an analysis plan on the Open Science Framework website.

Table 1: Risk of bias assessment for Population level studies

Reference	1. Bias due to confounding	2. Bias in selection of participants into the study	3. Bias due to misclassification of exposure	4. Bias due to deviations from intended exposures	5. Bias due to missing data	6. Bias in measurement of outcomes	7. Bias in selection of the reported result	Overall bias
**Abouk 2017**(2)	Rating: **Low**<br><br>Judgement: Parallel trends assumptions are tested and met AND dose-response is tested for AND there are no concurrent policy changes or concurrent policy changes are controlled for AND fixed effects for place and time over which exposure varies are included.	Rating: **Critical**<br><br>Judgement: Omission of dropouts from senior samples "One limitation of the samples of high school seniors is that they do not include in the target population those young men and women who drop out of high school before the last few months of senior year. This excludes relatively small proportions of each age cohort—approximately 8–15% of each age cohort nationally, according to U.S. Census statistics."	Rating: **Low**<br><br>Judgement: Authors specify frequency of e-cig use and measures are put in place to ensure anonymity of respondents (and this is known to participants)<br>OR Exposure is not self-reported (e.g. sales data / e-cigarette ban.	Rating: **Serious**<br><br>Judgement: Exposure is a regulatory measure and no discussion of implementation measures.	Rating: NA<br><br>Judgement: NA	Rating: **Low**<br><br>Judgement: Authors specify frequency of CC use and measures are put in place to ensure anonymity of respondents (and this is known to participants) OR tobacco use was biochemically validated OR outcome is not self-reported (e.g. sales data).	Rating: **Moderate**<br><br>Judgement: All expected outcomes are reported. No protocol or analysis plan has been published beforehand.	**Critical**
**Abouk 2023a**(5)	Rating: **Low**<br><br>Judgement: Cross-context design (natural experiment)	Rating: **Low**<br><br>Judgement: Data from annual MTF dataset and the biennial YRBSS, policy data. As per individual level, or based on comprehensive data	Rating: **Low**<br><br>Judgement: Data from annual MTF dataset and the biennial YRBSS, policy data. Authors specify frequency of e-cig use and measures are put in place to ensure anonymity of	Rating: **Low**<br><br>Judgement: E-cigarettes taxes. All other studies.	Rating: NA<br><br>Judgement: NA	Rating: **Low**<br><br>Judgement: Data from annual MTF dataset and the biennial YRBSS, policy data. Authors specify frequency of CC use and measures are put in place to ensure anonymity of respondents (and this is known to participants) OR tobacco use was biochemically validated) OR outcome is not self-reported (e.g. sales data).	Rating: **Moderate**<br><br>Judgement: All expected outcomes are reported. No protocol or analysis plan has been published beforehand.	**Moderate**

		e.g. state level sales data).	respondents (and this is known to participants; this is to reduce risk of misreport).						
**Abouk 2023b**(6)	Rating: **Low**<br><br>Judgement: Parallel trends assumptions are tested and met AND dose-response is tested for AND concurrent policy changes are controlled for AND fixed effects for place and time over which exposure varies are included.	Rating: **Low**<br><br>Judgement: Data are administrative birth records with geocodes provided by the National Centre for Health Statistics (NCHS).	Rating: **Low**<br><br>Judgement: Exposure are E-cigarette taxes.	Rating: **Low**<br><br>Judgement: All other studies. Exposure is regulatory measure and, with discussion of effectiveness of implementation and showing that the exposure affects e-cigarette use.	Rating: NA<br><br>Judgement: NA	Rating: **Low**<br><br>Judgement: Anonymised data from the National Centre for Health Statistics<br>(NCHS). Authors specify frequency of CC use.	Rating: **Moderate**<br><br>Judgement: All expected outcomes and analyses reported. No protocol or analysis plan has been published beforehand.	**Moderate**
**Beard 2022**(7)	Rating: **Low**<br><br>Judgement: Cross-context design. Time–series analysis of population trends with autoregressive integrated moving average with exogeneous input.	Rating: **Low**<br><br>Judgement: Data from STS.<br>As per individual level, or based on comprehensive data e.g. state level sales data).	Rating: **Serious**<br><br>Judgement: Data from STS. Randomly selected sample from non-nationally representative population, or relevant subsample that is endogenously impacted by the exposure.	Rating: **Low**<br><br>Judgement: "A number of tobacco control policies were adjusted for in the analyses using a composite score." All other studies.	Rating: NA<br><br>Judgement: NA	Rating: **Low**<br><br>Judgement: Data from STS. Authors specify frequency of CC use and measures are put in place to ensure anonymity of respondents (and this is known to participants) OR tobacco use was biochemically validated) OR outcome is not self-reported (e.g. sales data).	Rating: **Moderate**<br>Judgement: All expected outcomes are reported. No protocol or analysis plan has been published beforehand.	**Serious**
**Cantrell 2020**(8)	Rating: **Critical**<br><br>Judgement: "We did not include state-level cigarette taxes as	Rating: **Moderate**<br><br>Judgement: Missing data on any variable were less than 1% and these cases were	Rating: **Low**<br><br>Judgement: Authors specify frequency of e-cig use and measures are put in	Rating: **Low**<br><br>Judgement: E-cigarettes prices. All other studies.	Rating: NA<br><br>Judgement: NA	Rating: **Low**<br><br>Judgement: Authors specify frequency of e-cig use and measures are put in place to ensure anonymity	Rating: **Moderate**<br>Judgement: All expected outcomes are reported. No protocol or analysis plan has been	**Critical**

	this variable was highly correlated with the cigarette price variable. We also did not include state-level smoking prevalence and measures of individual-level cigarette use (in the e-cigarette models) or e-cigarette use (in the cigarette models) as these variables are endogenous to price variables and inclusion would bias price coefficients."	deleted. The sample for this study reflects data collected at baseline through wave 5, but includes only participants who completed two or more surveys during this period and who had Nielsen price data (n=11 578). Analytical samples included only those individuals with change over time on the outcome variable for each analysis."	place to ensure anonymity of respondents (and this is known to participants)<br>OR exposure is not self-reported (e.g. sales data / e-cigarette ban).			of respondents (and this is known to participants)<br>OR exposure is not self-reported (e.g. sales data / e-cigarette ban).	published beforehand.	
**Creamer 2021**(9)	Rating: **Serious**<br><br>Judgement: Confounders evaluated and adjusted in single context designs (e.g. interrupted time series in one setting).	Rating: **Low**<br><br>Judgement: As per individual level, or based on comprehensive data e.g. state level sales data).	Rating: **Low**<br><br>Judgement: "Data Collection<br>Both the YTS and NYTS survey procedures were designed to protect student privacy by ensuring that student participation was anonymous and voluntary. The survey was administered during one class period."	Rating: **Low**<br><br>Judgement: Introduction of e-cigarettes. All other studies.	Rating: NA<br><br>Judgement: NA	Rating: **Low**<br><br>Judgement: Authors specify frequency of CC use and measures are put in place to ensure anonymity of respondents (and this is known to participants).	Rating: **Moderate**<br>Judgement: All expected outcomes are reported. No protocol or analysis plan has been published beforehand.	**Serious**
**Dave 2019** (10)	Rating: **Moderate**	Rating: **Low**<br><br>Judgement: Data from YRBSS. As per	Rating: **Low**<br><br>Judgement: Data from YRBSS, MLSA.	Rating: **Low**<br><br>Judgement: All other studies. Effects of MLSA	Rating: NA<br><br>Judgement: NA	Rating: **Low**<br><br>Judgement: Data from YRBSS. Authors specify frequency of CC use	Rating: **Moderate**<br>Judgement: All expected outcomes reported. No	**Moderate**

	Judgement: Cross-context experiments in which parallel trend assumptions are met and there are no concurrent policy changes or those changes are controlled for, but dose-response is not tested for.	individual level, or based on comprehensive data e.g. state level sales data).	Authors specify frequency of e-cig use and measures are put in place to ensure anonymity of respondents (and this is known to participants; this is to reduce risk of misreport).			and measures are put in place to ensure anonymity of respondents (and this is known to participants) OR tobacco use was biochemically validated) OR outcome is not self-reported (e.g. sales data).	protocol or analysis plan has been published beforehand.	
**Dutra 2017**(10)	Rating: **Serious**<br><br>Judgement: Single context design.	Rating: **Moderate**<br><br>Judgement: Participants with missing values were excluded.	Rating: **Serious**<br><br>Judgement: specifies between ever users (tried even just one time) and current e-cigarette users (past 30 days on at least 1 day).	Rating: **Low**<br><br>Judgement: All other studies.	Rating: **NA**<br><br>Judgement: NA	Rating: **Serious**<br><br>Judgement: Judgement: specifies between ever users (tried even just one time) and current e-cigarette users (past 30 days on at least 1 day).	Rating: **Moderate**<br><br>Judgement: All expected outcomes reported. No protocol or analysis plan has been published beforehand.	**Serious**
**Dutra 2018**(11)	Rating: **Serious**<br><br>Judgement: "We did not<br>Adjust for state-level fixed effects because we used individual level<br>Data."	Rating: **Moderate**<br>Judgement: Respondents<br>With missing values for any variable of interest were also excluded.	Rating: **Low**<br><br>Judgement: MLSA was the exposure.	Rating: **Critical**<br><br>Judgement: "This analysis did not account for local<br>E-cigarette MLSA laws; we also did not adjust for cigarette MLSA<br>Laws that increased to age 21 during this time"	Rating: **NA**<br><br>Judgement: NA	Rating: **Low**<br><br>Judgement: NYTS data	Rating: **Moderate**<br>Judgement: All expected outcomes reported. No protocol or analysis plan has been published beforehand.	**Critical**
**Friedman 2015a**(12)	Rating: **Low**<br><br>Judgement: Cross context design (Natural experiment)	Rating: **Low**<br><br>Judgement: Data from NSDUH, Bureau of Labor Statistic, CDC trends application	Rating: **Low**<br><br>Judgement: Authors specify frequency of e-cig use and measures are put in place to ensure	Rating: **Low**<br><br>Judgement: All other studies. Exposures are state bans on e-cigarettes sales to minors and implementation is discussed.	Rating: NA<br><br>Judgement: NA	Rating: **Low**<br><br>Judgement: Data from NSDUH, NSDUH, Bureau of Labor Statistic, CDC trends application. Authors specify frequency of CC use and measures are put in place to	Rating: **Moderate**<br>Judgement: All expected outcomes are reported.No protocol or analysis plan has been	**Moderate**

	Regression analyses exploring how state bans on e-cigarette sales impact smoking rates in 12-17 years old compared to states without bans (multiples specifications tested, several falsification and placebo checks).	"as per individual level, or based on comprehensive data e.g. state level sales data)."	anonymity of respondents (and this is known to participants) – data from NSDUS-<br>and Exposure is not self-reported (state level data - bans).			ensure anonymity of respondents (and this is known to participants) OR tobacco use was biochemically validated OR outcome is not self-reported (e.g. sales data).	published beforehand.	
**Friedman 2015b**(13)	Rating: **Serious**<br><br>Judgement: Uses federal data not considering difference between states (US). Single context.	Rating: **Low**<br><br>Judgement:<br>Data from NYTS. As per individual level, or based on comprehensive data e.g. state level sales data).	Rating: **Low**<br><br>Judgement:<br>Data from NYTS. Authors specify frequency of e-cig use and measures are put in place to ensure anonymity of respondents (and this is known to participants; this is to reduce risk of misreport).	Rating: **Low**<br><br>Judgement: Discussion of implementation.	Rating: NA<br><br>Judgement: NA	Rating: **Low**<br><br>Judgement: Data from NYTS. Authors specify frequency of CC use and measures are put in place to ensure anonymity of respondents (and this is known to participants) OR tobacco use was biochemically validated OR outcome is not self-reported (e.g. sales data).	Rating: **Moderate**<br>Judgement: All expected outcomes reported. No protocol or analysis plan has been published beforehand.	**Serious**
**Friedman 2022**(14)	Rating: **Low**<br><br>Judgement: Cross context designs (natural experiment).	Rating: **Low**<br><br>Judgement: Data from Population Survey's 2010–2019 Tobacco Use Supplements.<br>As per individual level, or based on comprehensive data e.g. state level sales data).	Rating: **Low**<br><br>Judgement: Authors specify frequency of e-cig use and measures are put in place to ensure anonymity of respondents (and this is known to participants; this is to reduce risk of misreport).	Rating: **Low**<br><br>Judgement: "Multivariable linear regressions estimated two-way fixed effects analyses to assess ENDS and cigarette tax rates' relationships to recent and daily smoking and vaping, adjusting for an array of potential sociodemographic and policy confounders along with state and year fixed effects." All other studies.	Rating: NA<br><br>Judgement: NA	Rating: **Low**<br><br>Judgement: Data from Population Survey's 2010–2019 Tobacco Use Supplements. Authors specify frequency of CC use and measures are put in place to ensure anonymity of respondents (and this is known to participants) OR tobacco use was biochemically validated OR outcome is not self-reported (e.g. sales data).	Rating: **Moderate**<br>Judgement: All expected outcomes reported. No protocol or analysis plan has been published beforehand.	**Moderate**

**Foxon 2020** (1)	Rating: **Critical**<br><br>Judgement: All other studies. Policy changes are not mentioned.	Rating: **Low**<br><br>Judgement: Data from NYTS. As per individual level, or based on comprehensive data e.g. state level sales data).	Rating: **Moderate**<br><br>Judgement: Authors specify frequency of e-cig use but do not report measures put in place to ensure anonymity of respondents.	Rating: **Critical**<br><br>Judgement: Study explores trends in use prevalence and initiation age for adolescents aged 12–17 years with respect to cigarettes and/or ECs after Ec introduction in US market, but does not consider regulatory environment.	Rating: NA<br><br>Judgement: NA	Rating: **Low**<br><br>Judgement: Data from NYTS. Authors specify frequency of CC use and measures are put in place to ensure anonymity of respondents (and this is known to participants) OR tobacco use was biochemically validated OR outcome is not self-reported (e.g. sales data).	Rating: **Moderate**<br>Judgement: All expected outcomes reported. No protocol or analysis plan has been published beforehand.	**Critical**
**Gao 2020**(3)	Rating: **Serious**<br><br>Judgment: single context design (time-trend population-based analysis in Taiwan)	Rating: **Low**<br><br>Judgement: as per individual level, or based on comprehensive data (national representative surveys).	Rating: **Critical**<br><br>Judgement: All other studies<br><br>Only defines e-cigarettes users without further details on frequency.	Rating: **Low**<br><br>Judgement: Exposure is increase in popularity of e-cigarettes and authors show that the exposure affects e-cigarette use.	Rating: NA<br><br>Judgement: NA	Rating: **Critical**<br><br>Judgement: All other studies<br><br>Only defines e-cigarettes users without further details on frequency.	Rating:<br><br>**Moderate**<br><br>Judgement: All expected outcomes reported. No protocol or analysis plan has been published beforehand	**Critical**
**Hallingberg 2020**(15)	Rating: **Critical**<br><br>Judgement: All other studies.	Rating: **Low**<br><br>Judgement: Nationally representative sample, datasets taken from the SDDU, SALSUS, HBSC survey and SHRN survey.	Rating: **Critical**<br><br>Judgement: All other studies.<br>Authors do not specify e-cig use.	Rating: **Critical**<br><br>Judgement: Exposure is unregulated growth of e-cigarette use.	Rating: NA<br><br>Judgement: NA	Rating: **Low**<br><br>Judgement: Data from SALSUS, SDDU, HBSC Authors specify frequency of CC use and measures are put in place to ensure anonymity of respondents (and this is known to participants) OR tobacco use was biochemically validated) OR outcome is not self-reported (e.g. sales data).	Rating: **Moderate**<br>Judgement: All expected outcomes reported. No protocol or analysis plan has been published beforehand.	**Critical**
**Harrell 2022**(16)	Rating: **Low**<br><br>Judgement: Cross context design (natural experiment).	Rating: **Low**<br><br>Judgement: Data from NYTS.<br>As per individual level, or based on comprehensive data e.g. state level sales data).	Rating: **Low**<br><br>Judgement: Data from NYTS. Authors specify frequency of e-cig use and measures are put in place to ensure anonymity of	Rating: **Low**<br><br>Judgement: Introduction of e-cigarettes to market.	Rating: NA<br><br>Judgement: NA	Rating: **Low**<br><br>Judgement: Data form NYTS. Authors specify frequency of CC use and measures are put in place to ensure anonymity of respondents (and this is known to participants) OR tobacco use was biochemically validated) OR	Rating: **Moderate**<br>Judgement: All expected outcomes reported. No protocol or analysis plan has been published beforehand.	**Moderate**

			respondents (and this is known to participants; this is to reduce risk of misreport).			outcome is not self-reported (e.g sales data).		
**Hawkins 2022**(17)	Rating: **Moderate**<br><br>Judgement: Dose response not tested.	Rating: **Low**<br><br>Judgement: Data from the Massachusetts Youth Health Survey (YHS). As per individual level, or based on comprehensive data e.g. state level sales data).	Rating: **Low**<br><br>Judgement: Authors specify frequency of e-cig use and measures are put in place to ensure anonymity of respondents (and this is known to participants; this is to reduce risk of misreport).	Rating: **Low**<br><br>Judgement: Study examined associations between smoke-free laws prohibiting e-cigarettes with adolescent cigarette and e-cigarette use. Implementation discussed.	Rating: NA<br><br>Judgement: NA	Rating: **Low**<br><br>Judgement: Authors specify frequency of CC use and measures are put in place to ensure anonymity of respondents (and this is known to participants) OR tobacco use was biochemically validated OR outcome is not self-reported (e.g. sales data).	Rating: **Moderate**<br>Judgement: All expected outcomes reported. No protocol or analysis plan has been published beforehand.	**Moderate**
**Kowitt 2022**(18)	Rating: **Critical**<br><br>Judgement: Self-reported and recall variables	Rating: **Critical**<br><br>Judgement: Non-anonymised convenience sample.	Rating: **Critical**<br><br>Judgement: Non-anonymised sample and exposures are self-reported.	Rating: **Critical**<br><br>Judgement: Self-reported regulatory measures.	Rating: NA<br><br>Judgement: NA	Rating: **Critical**<br>Judgement: Non anonymised self-reported measurements for regulatory variables.	Rating: **Moderate**<br>Judgement: All expected outcomes reported. No protocol or analysis plan has been published beforehand.	**Critical**
**Levy 2019**(19)	Rating: **Serious**<br><br>Judgement: Single context design	Rating: **Low**<br><br>Judgement: Data from MTF, NYTS, YRBS, NSDUH and NHIS. As per individual level, or based on comprehensive data e.g. state level sales data).	Rating: **Low**<br><br>Judgement: Data from MTF, NYTS, YRBS, NSDUH and NHIS. Authors specify frequency of e-cig use and measures are put in place to ensure anonymity of respondents (and this is known to participants; this is to reduce risk of misreport).	Rating: **Low**<br><br>Judgement: All other studies.	Rating: NA<br><br>Judgement: NA	Rating: **Low**<br><br>Judgement: Data from MTF, NYTS, YRBS, NSDUH and NHIS. Authors specify frequency of e-cig use and measures are put in place to ensure anonymity of respondents (and this is known to participants).	Rating:<br><br>**Moderate**<br><br>Judgement: All expected outcomes reported. No protocol or analysis plan has been published beforehand.	**Serious**

**Nguyen 2021**(20)	Rating: **Low**<br><br>Judgement: Cross context design "difference-in-differences (DD) method to test the association between the bans and outcomes of interest. The DD exploits the variation in the adoption status (ie, provinces with a ban vs provinces without a ban) and the policy implementation dates across provinces with a ban"	Rating: **Low**<br><br>Judgement: Data from CTADS and CUMTS. As per individual level, or based on comprehensive data e.g. state level sales data).	Rating: **Low**<br><br>Judgement: Data from CTADS and CUMTS. Authors specify frequency of e-cig use and measures are put in place to ensure anonymity of respondents (and this is known to participants; this is to reduce risk of misreport).	Rating: **Serious**<br><br>Judgement: Bans on e-cigarette use in public places and workplaces in Canadian provinces.	Rating: NA<br><br>Judgement: NA	Rating: **Low**<br><br>Judgement: Authors specify frequency of CC use and anonymity of respondents maintained	Rating: **Moderate**<br>Judgement: All expected outcomes reported. No protocol or analysis plan has been published beforehand.	**Serious**
**Pesko 2016** (21)	Rating: **Low**<br><br>Judgement: Cross context design (natural experiment).	Rating: **Low**<br><br>Judgement: Data from YRBSS, CDC. As per individual level, or based on comprehensive data e.g. state level sales data).	Rating: **Low**<br><br>Judgement: Data from YRBSS, CDC. Authors specify frequency of e-cig use and measures are put in place to ensure anonymity of respondents (and this is known to participants; this is to reduce risk of misreport).	Rating: **Low**<br><br>Judgement: ENDS minimum legal purchase ages. All other studies.	Rating: NA<br><br>Judgement: NA	Rating: **Low**<br><br>Judgement: Data from YRBSS, CDC. Authors specify frequency of CC use and measures are put in place to ensure anonymity of respondents (and this is known to participants) OR tobacco use was biochemically validated) OR outcome is not self-reported (e.g. sales data).	Rating: **Moderate**<br>Judgement: All expected outcomes reported. No protocol or analysis plan has been published beforehand.	**Moderate**
**Pesko 2019** (22)	Rating: **Low**<br><br>Judgement: Cross context designs	Rating: **Low**<br><br>Judgement: As per individual level, or based on	Rating: **Low**<br><br>Judgement: Authors specify frequency of e-cig use and	Rating: **Serious**<br><br>Judgement: Does not discuss effects of ENDS MLSA on ends use. Exposure is regulatory	Rating: NA<br><br>Judgement: NA	Rating: **Low**<br><br>Judgement: Authors specify frequency of CC use and measures are put in place to ensure anonymity	Rating: **Moderate**<br>Judgement: All expected outcomes reported. No protocol or analysis	**Serious**

	(Natural experiment)	comprehensive data e.g. state level sales data).	measures are put in place to ensure anonymity of respondents (and this is known to participants; this is to reduce risk of misreport).	measure and no discussion of effectiveness of implementation AND failing to show that the exposure affects e-cigarette use.		of respondents (and this is known to participants) OR tobacco use was biochemically validated) OR outcome is not self-reported (e.g. sales data).	plan has been published beforehand.	
**Pesko 2021**(23)	Rating: **Low**<br><br>Judgement: Cross context design (natural experiment).	Rating: **Low**<br><br>Judgement: Data from NYTS. As per individual level, or based on comprehensive data e.g. state level sales data).	Rating: **Low**<br><br>Judgement: Data from NYTS. Authors specify frequency of e-cig use and measures are put in place to ensure anonymity of respondents (and this is known to participants; this is to reduce risk of misreport).	Rating: **Low**<br><br>Judgement: All other studies. E-cigarette taxes were the exposure.	Rating: NA<br><br>Judgement: NA	Rating: **Low**<br><br>Judgement: Data from NYTS. Authors specify frequency of CC use and measures are put in place to ensure anonymity of respondents (and this is known to participants) OR tobacco use was biochemically validated OR outcome is not self-reported (e.g. sales data).	Rating: **Moderate**<br>Judgement: All expected outcomes reported. No protocol or analysis plan has been published beforehand.	**Moderate**
**Pesko 2023**(24)	Rating: **Low**<br><br>Judgement: Cross context design (Natural experiment)	Rating: **Low**<br><br>Judgement: Data from NYTS (nationally representative survey).	Rating: **Low**<br><br>Judgement: Exposure is MLSA.	Rating: **Low**<br><br>Judgement: All other studies. Exposure is regulatory measure and, with discussion of effectiveness of implementation and showing that the exposure affects e-cigarette use.	Rating: NA<br><br>Judgement: NA	Rating: **Low**<br><br>Judgement: Data from NYTS. Authors specify frequency of CC use and measures are put in place to ensure anonymity of respondents (and this is known to participants)	Rating: **Moderate**<br>Judgement: All expected outcomes and analyses reported in full. No protocol. No protocol or analysis plan has been published beforehand.	**Moderate**
**Schneller 2022**(25)	Rating: **Serious**<br><br>Judgement: Single context designs (e.g. interrupted time series in one setting).<br>NY state	Rating: **Low**<br><br>Judgement: NYS data from International Tobacco Control Policy Evaluation Project Youth Tobacco and E-cigarette Tobacco and Vaping Survey. As per	Rating: **Low**<br><br>Judgement: Authors specify frequency of e-cig use and measures are put in place to ensure anonymity of respondents (and this is known to	Rating: **Low**<br><br>Judgement: "State-wide vaping flavour restriction on policy."<br>All other studies.	Rating: NA<br><br>Judgement: NA	Rating: **Low**<br><br>Judgement: Authors specify frequency of CC use and measures are put in place to ensure anonymity of respondents (and this is known to participants) OR tobacco use was biochemically validated) OR outcome is not self-reported (e.g. sales data).	Rating: **Moderate**<br>Judgement: All expected outcomes reported. No protocol or analysis plan has been published beforehand.	**Serious**

		individual level, or based on comprehensive data (e.g. state level sales data).	participants; this is to reduce risk of misreport).					
**Shabab 2021**(4)	Rating: **Moderate**<br><br>Judgement: Cross-context experiments in which parallel trend assumptions are met and there are no concurrent policy changes or those changes are controlled for, but dose-response is not tested for.	Rating: **Low**<br><br>Judgement: Data from NYTS. As per individual level, or based on comprehensive data e.g. state level sales data).” “as per individual level, or based on comprehensive data e.g. state level sales data).	Rating: **Low**<br><br>Judgement: Data from NYTS. Authors specify frequency of e-cig use and measures are put in place to ensure anonymity of respondents (and this is known to participants; this is to reduce risk of misreport).	Rating: **Low**<br><br>Judgement: All other studies.	Rating: NA<br><br>Judgement: NA	Rating: **Low**<br><br>Judgement: Data from NYTS. Authors specify frequency of CC use and measures are put in place to ensure anonymity of respondents (and this is known to participants) OR tobacco use was biochemically validated) OR outcome is not self-reported (e.g. sales data).	Rating: **Low**<br><br>Judgement: “The analysis plan for this study was pre-specified and logged at the Open Science Framework (https://osf.io/9zsw3).”	**Moderate**
**Wu 2022**(26)	Rating: **Low**<br><br>Judgement: Cross context designs including: Parallel trends assumptions are tested and met AND dose-response is tested for AND there are no concurrent policy changes or concurrent policy changes are controlled for AND fixed effects for place and time over which	Rating: **Low**<br><br>Judgement: Data from national surveys (Canada, UK and Australia). As per individual level, or based on comprehensive data e.g. state level sales data).	Rating: **Serious**<br><br>Judgement: Prevalence of current e-cig use. Specifies between ever-use and current e-cig use without further detail.	Rating: **Low**<br><br>Judgement: Tobacco tax/cigarette price as potential confounders for each country. All other studies.	Rating: NA<br><br>Judgement: NA	Rating: **Serious**<br><br>Judgement: Specifies between ever-use and current CC use without further detail.	Rating: **Moderate**<br><br>Judgement: All expected outcomes reported. No protocol or analysis plan has been published beforehand.	**Serious**

	exposure varies are included.							

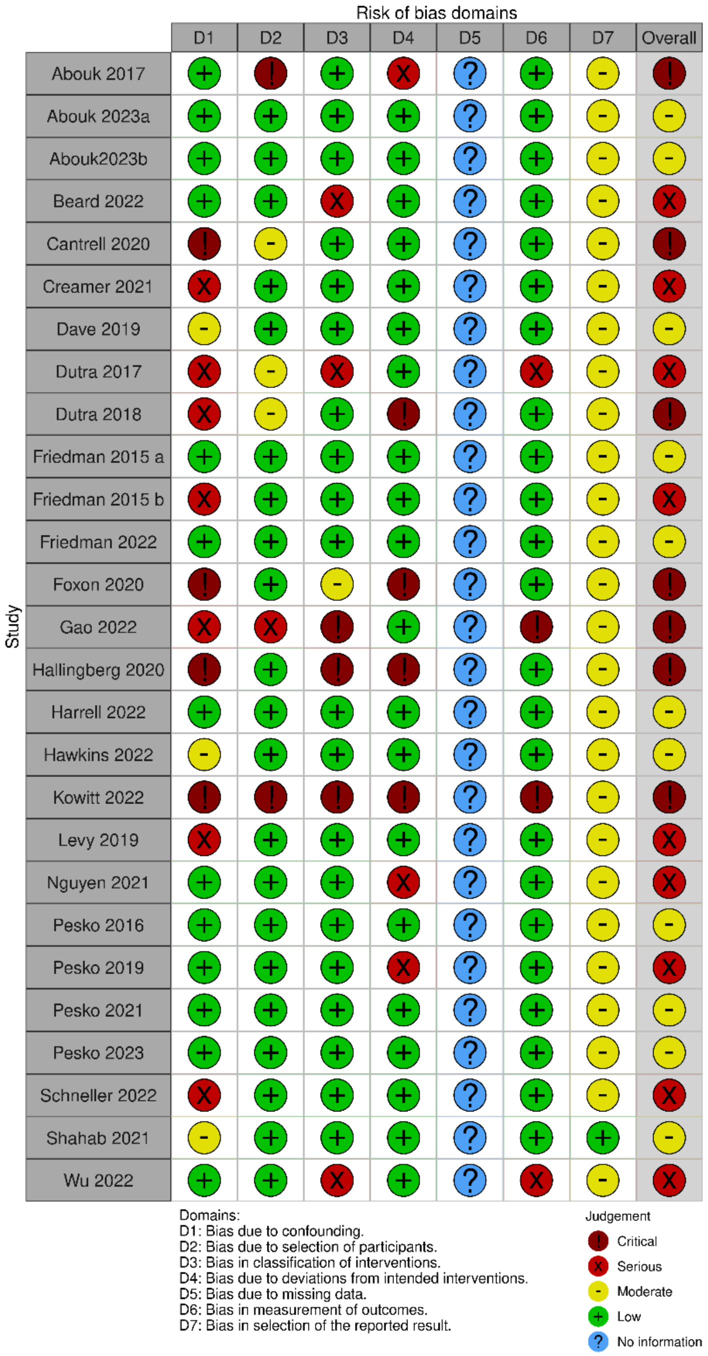

Figure 2. Risk of Bias graph: population level studies

3.2 Risk of bias of Tier 1 individual-level studies

Overall, 27 Tier 1 individual-level studies were deemed to be at serious risk of bias; whereas 13 were critical.

Bias due to confounding

Ten of the individual-level studies were judged to be at critical risk of bias due to confounding, mostly because propensity to smoke had not been adjusted for in statistical models. Thirty studies were deemed to be at serious risk of bias, with propensity to smoke adjusted for in these. None were considered at low risk of bias.

Bias in selection of participants into the study

Aleyan 2020 (27) was judged to be at critical risk of bias due to the use of a convenience sample in their analyses. By using a randomly selected sample from a non-nationally representative population, Lozano 2017 (28)was deemed to be at moderate risk of bias. All other studies were classified as low risk of bias for this domain.

Bias due to misclassification of exposure

Twenty-seven studies were considered to be at serious risk of bias as the frequency of EC use was not reported. Eleven studies were judged to be at low risk of bias. Do 2022(29) and Fearon 2023(30) were deemed to be at moderate risk of bias, since it was unclear if anonymity of data was ensured.

Bias due to missing data

Four studies were considered at critical risk of bias, eight studies at low risk of bias, and two studies at moderate risk. The remainder were considered at serious risk of bias, predominantly due to differences in missing data between groups.

Bias in measurement of the outcome

Lee 2019(31) was deemed at critical risk of bias as it was unclear how the outcomes were measured. Twenty-three studies were judged to be at serious risk of bias because the frequency of combustible tobacco use was not reported. Five studies were at moderate risk of bias for not reporting frequency of use. All other studies were judged at low risk of bias.

Bias in selection of the reported results

Kasza 2020 (32)and Lee 2019(31) were deemed at serious risk of bias, as there were incomplete data for expected outcomes. All the other studies were considered to be at moderate risk of bias, with all expected outcomes reported, but none reporting publishing a study protocol or analysis plan.

Table 2: Risk of bias assessment for Tier 1 Individual level studies

Study	1. Bias due to confounding	2. Bias in selection of participants into the study	3. Bias due to misclassification of exposure	4. Bias due to deviations from intended exposures	5. Bias due to missing data	6. Bias in measurement of outcomes	7. Bias in selection of the reported result	Overall bias
**Aleyan 2019**(33)	Rating: **Critical**<br>Judgement: Multiple factors related to propensity to smoke are measured at time of assessment of exposure.	Rating: **Low**<br><br>Judgement: Representative of districts.	Rating: **Low**<br><br>Judgement: Frequency reported.	Rating: NA<br><br>Judgement: NA	Rating: **Critical**<br>Judgement: Students with missing data were excluded	Rating: **Low**<br><br>Judgement: Frequency measured.	Rating: **Moderate**<br>Judgement:<br>All expected outcomes reported.	**Critical**
**Aleyan 2020**(27)	Rating: **Critical**<br>Judgement: Propensity to smoke is measured and controlled for.	Rating: **Critical**<br>Judgement: Convenience sample.	Rating: **Low**<br><br>Judgement: Frequency reported.	Rating: NA<br>Judgement: NA	Rating: **Critical**<br>Judgement: Only reports data from pupils who completed all three waves of study (do not report missing data overall at each wave).	Rating: **Low**<br><br>Judgement: Frequency measured.	Rating: **Moderate**<br>Judgement: All expected outcomes reported.	**Critical**
**Aleyan 2021**(34)	Rating: **Serious**<br>Judgement: Multiple factors related to propensity to smoke are measured at time of assessment of exposure. When confounders differ between groups, they are adjusted for/controlled using propensity score matching to assess the association of interest.	Rating: **Low**<br><br>Judgement: Representative of districts.	Rating: **Low**<br><br>Judgement: Frequency reported.	Rating: NA<br>Judgement: NA	Rating: **Low**<br><br>Judgement: "non-response rates for outcome measures including past 30-day cigarette smoking and dual use were low: 0.5% and 0.9%"	Rating: **Low**<br><br>Judgement: Frequency measured.	Rating: **Moderate**<br>Judgement: All expected outcomes reported.	**Serious**
**Barrington -Trimis 2018b**(35)	Rating: **Critical**<br>Judgement: Propensity to smoke not adjusted.	Rating: **Low**<br><br>Judgement: Nationally representative sample.	Rating: **Low**	Rating: NA<br>Judgement: NA	Rating: **Serious**<br>Judgement: Difference between groups not reported.	Rating: **Low**<br><br>Judgement: Frequency measured.	Rating: **Moderate**<br>Judgement: All expected outcomes reported.	**Critical**

			Judgement: Frequency measured although not reported.					
**Barrington -Trimis 2019**(36)	Rating: **Critical** Judgement: Propensity to smoke not adjusted.	Rating: **Low** Judgement: Nationally representative sample.	Rating: **Serious** Judgement: Frequency not measured	Rating: NA Judgement: NA	Rating: **Serious** Judgement: Difference between groups not reported.	Rating: **Low** Judgement: Frequency measured.	Rating: **Moderate** Judgement: All expected outcomes reported.	**Critical**
**Berry 2019**(35)	Rating: **Serious** Judgement: Multiple factors related to propensity to smoke are measured at time of assessment of exposure.	Rating: **Low** Judgement: Nationally representative sample.	Rating: **Serious** Judgement: No frequency reported.	Rating: NA Judgement: NA	Rating: **Serious** Judgement: Difference between groups not reported.	Rating: **Serious** Judgement: No frequency reported.	Rating: **Moderate** Judgement: All expected outcomes reported.	**Serious**
**Brouwer 2023**(37)	Rating: **Critical** Judgement: Propensity to smoke not adjusted.	Rating: **Low** Judgement: Nationally representative sample.	Rating: **Serious** Judgement: Frequency not reported.	Rating: NA Judgement: NA	Rating: **Serious** Judgement: Difference between groups not reported.	Rating: **Serious** Judgement: Frequency not reported.	Rating: **Moderate** Judgement: All expected outcomes reported.	**Critical**
**Cheng 2019** (38)	Rating: **Serious** Judgement: Multiple factors related to propensity to smoke are measured at time of assessment of exposure, and introduced into the RMSEA model.	Rating: **Low** Judgement: Nationally representative sample.	Rating: **Serious** Judgement: No frequency reported.	Rating: NA Judgement: NA	Rating: **Serious** Judgement: Follow up rate less than 80%, but did not report the different between groups.	Rating: **Serious** Judgement: No frequency reported.	Rating: **Moderate** Judgement: All expected outcomes reported.	**Serious**
**Chien 2019**(39)	Rating: **Serious** Judgement: Multiple factors related to propensity to smoke are measured at time of assessment of exposure.	Rating: **Low** Judgement: randomly selected from a national/state/province level representative survey OR relevant subsample from representative survey that is itself not impacted by the exposure variable (e.g., age is not impacted by e-cigarette use, but people with certain	Rating: **Serious** Judgement: No frequency reported. Specifies between ever-use and current e-cig use without further detail	Rating: NA Judgement: NA	Rating: **Low** Judgement: Attribution and sensitivity analysis.	Rating: **Serious** Judgement: No frequency reported.	Rating: **Moderate** Judgement: All expected outcomes reported.	**Serious**

		medical conditions could be) AND accounts for non-responders in weighting by population characteristics.						
**Do 2022** (29)	Rating: **Serious** Judgement: Propensity to smoke adjusted.	Rating: **Low** Judgement: Nationally representative sample.	Rating: **Moderate** Judgement: Frequency reported (Past 30-Day E-cigarette Use, Frequent to Daily E-Cigarette Use y/n); anonymity unknown.	Rating: NA Judgement: NA	Rating: **Low** Judgement: Additional participants recruited at each wave to account for attrition' and participants had to provide outcome data at both time points.	Rating: **Moderate** Judgement: Frequency reported (Past 30-Day E-cigarette Use, Frequent to Daily E-Cigarette Use y/n).	Rating: **Moderate** Judgement: All expected outcomes reported.	**Serious**
**Duan 2021** (40)	Rating: **Serious** Judgement: Multiple factors related to propensity to smoke are measured at time of assessment of exposure. When confounders differ between groups, they are adjusted for/controlled using propensity score matching to assess the association of interest.	Rating: **Low** Judgement: Nationally representative sample.	Rating: **Serious** Judgement: Frequency not reported.	Rating: NA Judgement: NA	Rating: **Low** Judgement: Sensitivity analysis found no difference; waves had different sample size - hard to tell the missing data.	Rating: **Serious** Judgement: Frequency not reported.	Rating: **Moderate** Judgement: All expected outcomes reported.	**Serious**
**Fearon 2023**(30)	Rating: **Critical** Judgement: Propensity to smoke not adjusted.	Rating: **Low** Judgement: Nationally representative sample.	Rating: **Moderate** Judgement: Frequency reported; anonymity unknown.	Rating: NA Judgement: NA	Rating: **Serious** Judgement: Difference between groups not reported.	Rating: **Moderate** Judgement: Frequency reported; anonymity unknown.	Rating: **Moderate** Judgement: All expected outcomes reported.	**Critical**
**Friedman 2020**(41)	Rating: **Serious** Judgement: Propensity to smoke adjusted.	Rating: **Low** Judgement: Nationally representative sample.	Rating: **Serious** Judgement: Frequency not reported.	Rating: NA Judgement: NA	Rating: **Serious** Judgement: Difference between groups not reported.	Rating: **Serious** Judgement: Frequency not reported.	Rating: **Moderate** Judgement: All expected outcomes reported.	**Serious**
**Glantz 2023**(42)	Rating: **Serious** Judgement: Propensity to smoke adjusted.	Rating: **Low** Judgement: Nationally representative sample.	Rating: **Serious** Judgement: Frequency not reported.	Rating: NA Judgement: NA	Rating: **Critical** Judgement: No sensitivity analysis.	Rating: **Serious** Judgement: Frequency not reported.	Rating: **Moderate** Judgement: All expected outcomes reported.	**Critical**

**Gueorguieva 2020** (43)	Rating: **Critical** Judgement: Propensity to smoke not adjusted.	Rating: **Low** Judgement: Nationally representative sample.	Rating: **Serious** Judgement: Frequency not reported.	Rating: NA Judgement: NA	Rating: **Serious** Judgement: Difference between groups not reported.	Rating: **Serious** Judgement: Frequency not reported.	Rating: **Moderate** Judgement: All expected outcomes reported.	**Critical**
**Hair 2019** (44)	Rating: **Serious** Judgement: Propensity to smoke adjusted.	Rating: **Low** Judgement: Nationally representative sample.	Rating: **Serious** Judgement: Frequency not reported.	Rating: NA Judgement: NA	Rating: **Serious** Judgement: Difference between groups not reported.	Rating: **Serious** Judgement: Frequency not reported.	Rating: **Moderate** Judgement: All expected outcomes reported.	**Serious**
**Hair 2021a**(45)	Rating: **Serious** Judgement: Propensity to smoke adjusted.	Rating: **Low** Judgement: Nationally representative sample.	Rating: **Serious** Judgement: Frequency not reported.	Rating: NA Judgement: NA	Rating: **Serious** Judgement: Difference between groups not reported.	Rating: **Serious** Judgement: Frequency not reported.	Rating: **Moderate** Judgement: All expected outcomes reported.	**Serious**
**Hair 2021b**(46)	Rating: **Serious** Judgement: Propensity to smoke adjusted.	Rating: **Low** Judgement: Nationally representative sample.	Rating: **Serious** Judgement: Frequency not reported.	Rating: NA Judgement: NA	Rating: **Moderate** Judgement: No sensitivity analyses were conducted, missing data listwise deleted but not accounted for in analyses.	Rating: **Serious** Judgement: Frequency not reported.	Rating: **Moderate** Judgement: No protocol or data analysis plan.	**Serious**
**Hammond 2017**(46)	Rating: **Critical** Judgement: propensity to smoke not adjusted.	Rating: **Low** Judgement: Nationally representative sample.	Rating: **Serious** Judgement: Frequency not reported.	Rating: NA Judgement: NA	Rating: **Serious** Judgement: Difference between groups not reported.	Rating: **Serious** Judgement: Frequency unclear.	Rating: **Moderate** Judgement: All expected outcomes reported.	**Critical**
**Han 2023**(47)	Rating: **Serious** Judgement: Propensity to smoke adjusted.	Rating: **Low** Judgement: Nationally representative sample.	Rating: **Serious** Judgement: Frequency not reported.	Rating: NA Judgement: NA	Rating: **Moderate** Judgement: No sensitivity analyses were conducted.	Rating: **Serious** Judgement: Frequency not reported.	Rating: **Moderate** Judgement: All expected outcomes reported.	**Serious**
**Harlow 2022**(48)	Rating: **Serious** Judgement: Propensity to smoke adjusted.	Rating: **Low** Judgement: Nationally representative sample.	Rating: **Low** Judgement: Frequency measured.	Rating: NA Judgement: NA	Rating: **Low** Judgement: Data were missing for <3% of participants for all variables except: wave 1 other tobacco use (4.4%), wave 3 flavor of first e-cigarette (8.7%), wave 4 ever cigarette smok- ing (4.4%), and wave	Rating: **Serious** Judgement: Frequency not reported.	Rating: **Moderate** Judgement: All expected outcomes reported.	**Serious**

					4 ever e-cigarette use (4.4%).			
**Huang 2023**(32)	Rating: **Serious** Judgement: Propensity to smoke adjusted.	Rating: **Low** Judgement: Nationally representative sample.	Rating: **Serious** Judgement: Frequency not reported.	Rating: NA Judgement: NA	Rating: **Serious** Judgement: Difference between groups not reported.	Rating: **Serious** Judgement: Frequency not reported.	Rating: **Moderate** Judgement: All expected outcomes reported.	**Serious**
**Kasza 2020**(32)	Rating: **Critical** Judgement: Propensity to smoke not adjusted.	Rating: **Low** Judgement: Nationally representative sample.	Rating: **Low** Judgement: Frequency measured, confidential.	Rating: NA Judgement: NA	Rating: **Serious** Judgement: Difference between groups unknown.	Rating: **Low** Judgement: Frequency measured, confidential.	Rating: **Serious** Judgement: For the initiation of frequent use; incomplete data reported.	**Critical**
**Lee 2019**(31)	Rating: **Serious** Judgement: Propensity to smoke adjusted.	Rating: **Low** Judgement: Nationally representative sample.	Rating: **Serious** Judgement: Frequency not reported.	Rating: NA Judgement: NA	Rating: **Serious** Judgement: Difference between groups unknown.	Rating: **Critical** Judgement: How outcome was measured is unclear.	Rating: **Serious** Judgement: Not all expected outcome reported.	**Critical**
**Loukas 2022**(28)	Rating: **Serious** Judgement: Propensity to smoke adjusted.	Rating: **Low** Judgement: Nationally representative sample.	Rating: **Serious** Judgement: Frequency not reported.	Rating: NA Judgement: NA	Rating: **Low** Judgement: Sensitivity analysis showed no significant difference.	Rating: **Low** Judgement: Frequency reported.	Rating: **Moderate** Judgement: All expected outcomes reported.	**Serious**
**Lozano 2017**(28)	Rating: **Serious** Judgement: Propensity to smoke adjusted.	Rating: **Serious** Judgement: Randomly selected sample from non-nationally representative population.	Rating: **Serious** Judgement: Frequency not reported.	Rating: NA Judgement: NA	Rating: **Serious** Judgement: Difference between groups unknown.	Rating: **Moderate** Judgement: Frequency reported, anonymity unknown.	Rating: **Moderate** Judgement: All expected outcomes reported.	**Serious**
**Melka 2021**(49)	Rating: **Serious** Judgement: Propensity to smoke adjusted.	Rating: **Low** Judgement: Nationally representative sample.	Rating: **Serious** Judgement: Frequency not reported.	Rating: NA Judgement: NA	Rating: **Serious** Judgement: Difference between groups unknown.	Rating: **Moderate** Judgement: Frequency reported, anonymity unknown.	Rating: **Moderate** Judgement: All expected outcomes reported.	**Serious**
**Osibogun 2020**	Rating: **Serious** Judgement: Propensity to smoke adjusted.	Rating: **Low** Judgement: Nationally representative sample.	Rating: **Low** Judgement: Frequency reported.	Rating: NA Judgement: NA	Rating: **Serious** Judgement: Difference between groups unknown.	Rating: **Low** Judgement: Frequency reported.	Rating: **Moderate** Judgement: All expected outcomes reported.	**Serious**
**Owotomo 2020**(50)	Rating: **Serious** Judgement: Propensity to smoke adjusted.	Rating: **Low** Judgement: Nationally representative sample.	Rating: **Serious** Judgement: Frequency not reported.	Rating: NA Judgement: NA	Rating: **Serious** Judgement: Difference between groups unknown.	Rating: **Serious** Judgement: Frequency not reported.	Rating: **Moderate** Judgement: All expected outcomes reported.	**Serious**

**Pierce 2021**	Rating: **Serious** Judgement: Propensity to smoke adjusted.	Rating: **Low** Judgement: Nationally representative sample.	Rating: **Low** Judgement: Frequency reported.	Rating: NA Judgement: NA	Rating: **Critical** Judgement: The response rate to the wave 1 household screener was 54%. The unweighted attrition rate among the wave 1 (2013–2014) sample was 16% at wave 2 (2015), 21% at wave 3 (2016), and 27% at wave 4 (2017).	Rating: **Low** Judgement: Frequency reported.	Rating: **Moderate** Judgement: All expected outcomes reported.	**Critical**
**Staff 2022**	Rating: **Serious** Judgement: Propensity to smoke adjusted.	Rating: **Low** Judgement: Nationally representative sample.	Rating: **Low** Judgement: Frequency reported, confidential.	Rating: NA Judgement: NA	Rating: **Serious** Judgement: Difference between groups unknown.	Rating: **Low** Judgement: Frequency reported, confidential.	Rating: **Moderate** Judgement: All expected outcomes reported.	**Serious**
**Stanton 2019**	Rating: **Serious** Judgement: Propensity to smoke adjusted.	Rating: **Low** Judgement: Nationally representative sample.	Rating: **Low** Judgement: Frequency reported.	Rating: NA Judgement: NA	Rating: **Serious** Judgement: Difference between groups unknown.	Rating: **Moderate** Judgement: Frequency unclear.	Rating: **Moderate** Judgement: All expected outcomes reported.	**Serious**
**Stanton 2020**	Rating: **Serious** Judgement: Propensity to smoke adjusted.	Rating: **Low** Judgement: Nationally representative sample.	Rating: **Serious** Judgement: Frequency not reported.	Rating: NA Judgement: NA	Rating: **Serious** Judgement: Difference between groups unknown.	Rating: **Serious** Judgement: Frequency not reported.	Rating: **Moderate** Judgement: All expected outcomes reported.	**Serious**
**Stanton 2023**	Rating: **Serious** Judgement: Propensity to smoke adjusted.	Rating: **Low** Judgement: Nationally representative sample.	Rating: **Low** Judgement: Frequency reported.	Rating: NA Judgement: NA	Rating: **Serious** Judgement: Difference between groups unknown.	Rating: **Low** Judgement: Frequency reported.	Rating: **Moderate** Judgement: All expected outcomes reported.	**Serious**
**Stokes 2021**	Rating: **Serious** Judgement: Propensity to smoke adjusted.	Rating: **Low** Judgement: Nationally representative sample.	Rating: **Serious** Judgement: Frequency not reported.	Rating: NA Judgement: NA	Rating: **Low** Judgement: Results were similar when using complete case versus multiple imputation.	Rating: **Serious** Judgement: Frequency not reported.	Rating: **Moderate** Judgement: All expected outcomes reported.	**Serious**
**Sumbe 2021**	Rating: **Critical** Judgement: Propensity to smoke not adjusted.	Rating: **Low** Judgement: Nationally representative sample.	Rating: **Serious** Judgement: Frequency not reported.	Rating: NA Judgement: NA	Rating: **Serious** Judgement: Difference between groups unknown.	Rating: **Serious** Judgement: Frequency unclear.	Rating: **Moderate** Judgement: All expected outcomes reported.	**Critical**

**Sun 2022**	Rating: **Serious**<br>Judgement: Propensity to smoke adjusted.	Rating: **Low**<br>Judgement: Nationally representative sample.	Rating: **Serious**<br>Judgement: Frequency not reported.	Rating: NA<br>Judgement: NA	Rating: **Serious**<br>Judgement: Difference between groups unknown.	Rating: **Serious**<br>Judgement: Frequency not reported.	Rating: **Moderate**<br>Judgement: All expected outcomes reported.	**Serious**
**Sun 2023**	Rating: **Serious**<br>Judgement: Confounders differ between groups.	Rating: **Low**<br>Judgement: Nationally representative sample.	Rating: **Serious**<br>Judgement: Frequency not reported.	Rating: NA<br>Judgement: NA	Rating: **Low**<br>Judgement: sensitivity analyses conducted	Rating: **Serious**<br>Judgement: Frequency not reported.	Rating: **Moderate**<br>Judgement: All expected outcomes reported.	**Serious**
**Watkins 2018**	Rating: **Serious**<br>Judgement: Propensity to smoke adjusted.	Rating: **Low**<br>Judgement: Nationally representative sample.	Rating: **Serious**<br>Judgement: Frequency not reported.	Rating: NA<br>Judgement: NA	Rating: **Serious**<br>Judgement: Difference between groups unknown.	Rating: **Serious**<br>Judgement: Frequency unclear.	Rating: **Moderate**<br>Judgement: All expected outcomes reported.	**Serious**
**Xu 2022**	Rating: **Serious**<br>Judgement: Propensity to smoke adjusted.	Rating: **Low**<br>Judgement: Nationally representative sample.	Rating: **Serious**<br>Judgement: Frequency not reported.	Rating: NA<br>Judgement: NA	Rating: **Serious**<br>Judgement: Difference between groups unknown.	Rating: **Serious**<br>Judgement: Frequency not reported.	Rating: **Moderate**<br>Judgement: All expected outcomes reported.	**S**

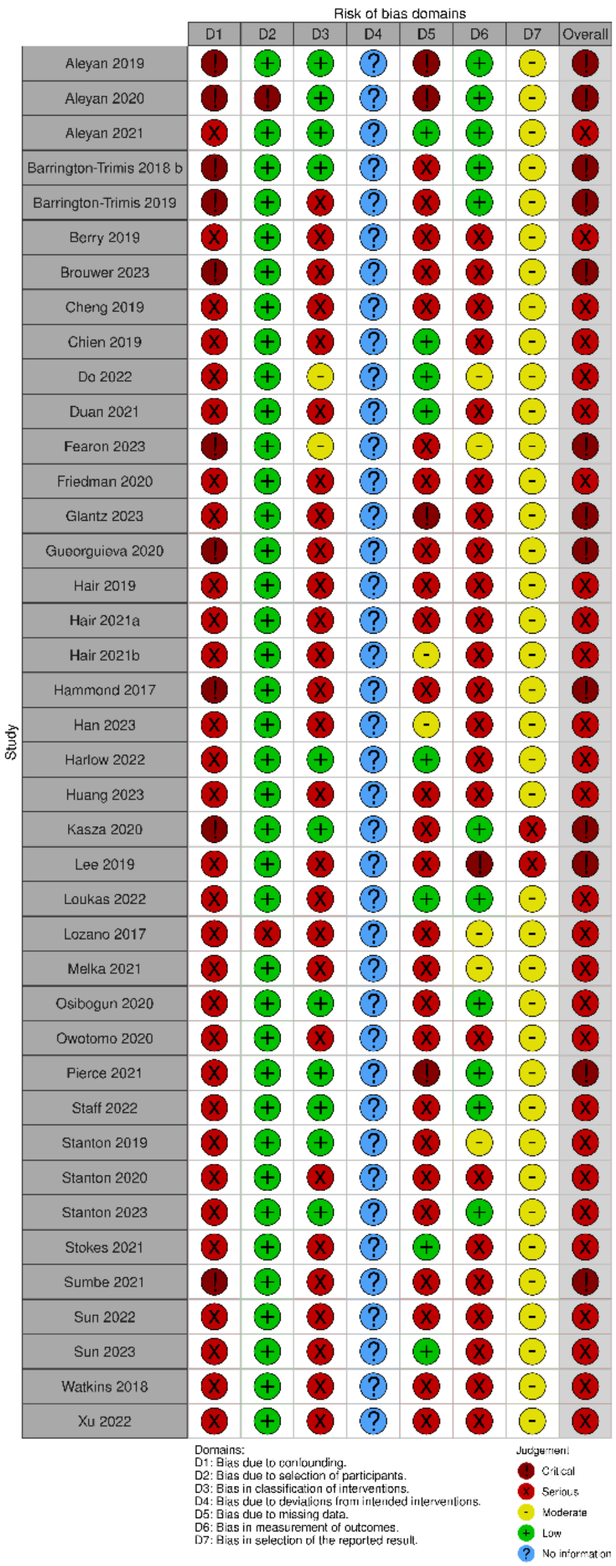

Figure 3. Risk of bias graph: Individual level studies

4. Qualitative Comparative Analysis (QCA)

4.1 QCA stage 0 - Identification of underlying theory and organisation of cases

Below we provide a description of each 'condition' considered ('conditions' are analogous to factors or variables), with each representing a binary construct (i.e. a study being a full member (1) or non-member (0) of each set). However, we also implemented 'fuzzy-set' coding for some conditions, including the outcome, to recognise ambiguities and to allow studies to be partial members of sets.

Table 3: Description of conditions

**Age**	Age is a core focus of the current review. US studies exploring the relationship between age and the incidence of electronic cigarette smoking suggest that prevalence rates accelerate between aged 12-17 years before slowing down thereafter.(51)	Two separate conditions initially reflecting whether studies included those aged under 18 and, separately, whether studies included those aged over 18 (up to 29). As we had a modest number of cases (n=18), after further exploration we developed a condition that reflected whether or not studies included participants aged under and over 18.
**Socioeconomic status (SES)**	As smoking rates differ via SES, it is plausible that the impact of vaping on smoking could also differ by SES.	Despite being a condition theorised to explain study-level variation, we were not able to include SES as a condition due to the majority of studies not including data on SES level or subgroup analyses by SES and a lack of comparability in available measures.
**Gender/sex**	There is some data to suggest that nicotine affects different sexes differently, and also that smoking rates can vary by gender/sex.	**Gender/sex:** reflected whether studies included fewer or the same/greater proportion of males/men (males is a sex) in the study compared to the median across studies. Where studies did not report these data, this was coded as 0.49 (equivalent to missing data).
**Level of youth cigarette use**	The proportion of youth using cigarettes in a population could plausibly impact changes in smoking rates (e.g. if smoking rates are high to start	**Level of youth cigarette use:** reflected whether the level of youth cigarette use within a study was lower or the same/above the median across studies included within the QCA. Where studies did not report these data, this was coded as 0.49 (equivalent to missing data).

	with, they may be less likely to increase further).	
**Level of youth EC use**	The proportion of youth using ECs could impact the ability of a study to detect associations between vaping and subsequent smoking.	**Level of youth electronic cigarette use:** reflected whether the level of youth EC use within a study was lower or the same/above the median across studies included within the QCA. Where studies did not report these data, this was coded as 0.49 (equivalent to missing data).
**Exposure**	It is plausible that different exposures could have different effects.	**Exposure:** Whether the study was evaluating a specific policy (coded as 1) or the influence of changes in the prevalence of vaping in the population (coded as 0).
**Comparator**	It is plausible that choice of comparator could impact the observed effects across studies.	**Comparator:** Whether the study was comparing policies/changes with a comparison within the same setting (0) or within a different setting with different levels of electronic cigarette use or policies around this (1).
**Definition of smoking used**	Studies use various definitions of smoking intensity, and existing literature suggests important differences between experimental smoking and regular cigarette use.	Whether the study relies on a definition of smoking that incorporates frequency and excluded infrequent smoking within its definition (once a month or less frequent) (1) versus a definition that does not incorporate frequency or includes infrequent smoking (0).
**Definition of vaping used**	Studies use varying definitions of vaping, and it is plausible that the frequency of vaping could affect pathways (e.g. nicotine addiction) that could explain relationships between vaping and smoking.	Whether the study relies on a definition of vaping that incorporates frequency and excluded infrequent smoking within its definition (once a month or less frequent) (1) versus a definition that does not incorporate frequency or includes infrequent vaping (0).

Given the number of cases (studies) that were available for analysis (n-18), we were unable to enter all conditions simultaneously without encountering issues around limited diversity and the possibility that the models would generate contradictions and have low consistency.(52) Drawing on the practice elsewhere (see

(53,54)), we organised these conditions into different sub-research questions initially before intending to develop a consolidated model that drew on conditions and configurations of significance. Therefore, in addition to the overall question being addressed around 'Which characteristics around Population, Intervention, Context, Outcome (measurement), and Study help to explain if policies to improve the accessibility of electronic cigarettes lead to decreases or increases in combustible tobacco use on a population level?', the following sub-questions were developed:

- Which study-level population characteristics explain whether policies to improve the accessibility of ECs lead to decreases or increases in combustible tobacco use on a population level?
    - Conditions operationalised: Gender; Age <18 included; Age ≥ 18 included
- Which study-level contextual characteristics explain whether policies to improve the accessibility of ECs lead to decreases or increases in combustible tobacco use on a population level?
    - Conditions operationalised: Level of youth cigarette use; Level of youth electronic cigarette use
- Which study-level intervention and methodological characteristics explain whether policies to improve the accessibility of ECs lead to decreases or increases in combustible tobacco use on a population level?
    - Conditions operationalised: Exposure, Comparator, Definition of smoking used; Definition of vaping used

4.2 QCA stage 1 – Configuration of datasets and creation of data tables

Next, we developed data tables for the QCA models reflecting the conditions (characteristics of studies) and the outcome for studies for the questions above as well as a fourth data table that reflected an intended consolidated model which would bring together conditions and configurations that appeared to explain variation in the data.

We aimed to allocate studies into sets based on the direction of the outcome, and recognised that studies could also be partial set members (i.e. the increased availability of e-cigarettes could have partially negative or partially positive impacts on combustible tobacco smoking) (see table 4 below).

Table 4: Allocation criteria used to group studies sets based on negative or positive associations

**Effectiveness rating**	**Numeric value**	**Allocation criteria**
Negative impacts: Evidence that greater access to e-cigarettes lead to increased levels of smoking	0	Increased e-cigarette use/availability led to more combusted tobacco use than would be expected OR decreased e-cigarette use/ introduction of e-cigarette restrictions led to less combustible use (statistically significant differences)
Partially negative impacts	0.33	Evidence that e-cigarette use/availability led to more combusted tobacco use than would be expected OR decreased e-cigarette use/

		introduction of e-cigarette restrictions led to less combustible use (non-statistically significant differences)
No difference	0.49	Evidence that there was no association in either direction
Partially positive impacts	0.66	Evidence that e-cigarette use/availability led to less combusted tobacco use than would be expected OR decreased e-cigarette use/ introduction of e-cigarette restrictions led to higher combustible use (non-statistically significant differences)
Positive impacts: Evidence that greater access to e-cigarettes lead to lower levels of smoking	1	Increased e-cigarette use/availability led to less combusted tobacco use than would be expected OR decreased e-cigarette use/ introduction of e-cigarette restrictions led to higher combustible use (statistically significant differences)

For each condition, a coding scheme was developed to determine whether the study was a member of the set (or not). Creation of the data table provided an indication that there may be issues with conducting a full QCA, with some conditions having uniform distributions and not adhering to QCA rules of thumb requiring around a quarter to a third of studies as having a condition or outcome to be present/absent.(55)

QCA stage 2 – Creation of truth tables

Our analytical strategy involved first creating a 'truth table' based on the theorised conditions; we then expected to revise this initial model based on the quality of the truth table before then seeking to produce a reduced truth table and minimised solution based on this. A 'truth table' sorts cases according to the configuration of conditions they exhibit.

The truth table below (Table 5) found that all three studies where there were fewer men than expected and where there were exclusions on age (either those under or over 18 were excluded in the study) showed a consistent positive impact - that ECs led to lower levels of combustible tobacco use. This latter condition combined information from two separate conditions reflecting age restrictions to better adhere to QCA convention.(56) However, a configuration showing the opposite pattern was supported by a mixture of studies that showed both negative and positive impacts of vaping. This suggests that while gender and the age distribution might be important in understanding some of the differences in outcomes, other conditions are needed and age and gender alone are not sufficient in explaining differences in outcomes. Given these inconsistencies, no further minimisation was appropriate in this case.

Table 5: Truth table of population characteristics

**Full age**	**Gender**	**Outcome**	**N (cases)**	**Consistency**	**PRI**	**Studies**

**distribution**						
0	0	1	3	1	1	Friedman 2015a(12); Nguyen 2021(20); Pesko 2019(22)
1	0	0	7	0.859	0.841	Beard 2022(7); Harrell 2022(16); Hawkins 2022(17); Pesko 2021(23); Wu 2022(26); Abouk 2023b(6),Pesko 2023(24)
0	1	0	2	0.829	0.795	Friedman 2022(14); Hallingberg 2020(15)
1	1	0	6	0.499	0.414	Abouk 2017(2); Abouk 2023a(5); Cantrell 2020(8); Dave 2019(57); Kowitt 2022(18); Schneller 2022(25)

Consistency/Sufficiency: A measure of the consistency of a subset relationship between the configuration of conditions and the outcome (note, consistency thresholds were set at 0.875)

PRI: Proportional Reduction in Inconsistency is an additional measure of consistency/sufficiency and refers to the extent in which a configuration reduces the level of inconsistency in predicting a successful outcome, with higher values indicating greater reductions in inconsistency

Truth table B - Exploration of smoking context characteristics

The data table supporting this model indicated a large degree of ambiguity in the coding for both contextual factors included in this truth table. No configuration of smoking contextual characteristics was observed to be sufficient to trigger a 'positive' configuration of studies (unlike the truth table above). Configurations tended to contain studies reporting both positive and negative impacts of increased access to EC. From this exploration, we do not see evidence that the smoking context (operationalised in two specific conditions) explained differences in study outcomes, and a large part of this is likely a reflection of the high level of missingness.

Truth table C - Exploration of study-level intervention and methodological characteristics

Explorations of the data table suggested that two conditions that we intended to include (the exposure and the definition of vaping used) would fail to adhere to QCA standards and were omitted from the construction

of the truth table (shown below). However, as was the case for truth table 1, there was suggestive evidence that the definition of smoking and type of comparator used could explain some patterns. Studies that drew on a definition of smoking that incorporated the frequency of smoking and that made comparisons with settings with different smoking policies/contexts were those that found that increasing access to EC reduced levels of combustible tobacco use (see first row of truth table). Meanwhile, the final configuration with the opposite set of characteristics was composed of mainly studies that found a negative association, albeit with a contradictory study in the form of Abouk (5). Similarly, both other configurations contained studies with both positive and negative associations, and overall the evidence suggests that other conditions are needed to explain differences in study impacts. Given these inconsistencies, no further minimisation was appropriate in this case.

Table 6: Truth table of methodological characteristics

**Definition of Smoking**	**Comparator**	**Outcome**	**N (cases)**	**Sufficiency**	**PRI**	**Studies**
1	1	1	2	1	1	Dave 2019(57); Pesko 2019(22)
0	1	0	7	0.857	0.857	Abouk 2017(2); Friedman 2015a(12); Friedman 2022(12); Pesko 2021(23); Wu 2022(26); Abouk 2023b(6),Pesko 2023(24)
1	0	0	3	0.607	0.459	Hallingberg 2020(15); Kowitt 2022(18); Nguyen 2021(20)
0	0	0	6	0.497	0.394	Abouk 2023a(5); Beard 2022(7); Cantrell 2020(8); Harrell 2022(16); Hawkins 2022(17); Schneller 2022(25)

Consistency/Sufficiency: A measure of the consistency of a subset relationship between the configuration of conditions and the outcome (note, consistency thresholds were set at 0.875)

PRI: Proportional Reduction in Inconsistency is an additional measure of consistency/sufficiency and refers to the extent in which a configuration reduces the level of inconsistency in predicting a is sufficient in triggering successful outcome, with higher values indicating greater reductions in inconsistency

Truth table D - Exploration of consolidated model

Building on the results of truth tables A and C, a consolidated truth table was constructed that included gender, whether the study excluded participants based on age, the definition of smoking used, and the comparator used. The truth table identified that a number of configurations could trigger a positive impact based on population and study quality indicators. However, several of these rows were supported by a single study. While on the whole, the truth table showed that the four conditions helped to distinguish studies with a positive association from those with a negative association; there were some contradictions with Abouk (5) again appearing in a configuration with studies that identified a negative association. Furthermore, studies that identified partially positive associations were also included in configurations with clearly negative associations. Such contradictions undermine our ability to say that the included conditions clearly differentiate between studies identifying positive and negative associations. Given that our underlying 'theory' is one that is based on 'PICOS' as a loose conceptual framework, and involves refining as much as testing theory in this instance, such contradictions are much more problematic than might be the case in other applications of QCA, particularly where a qualitative explanation could be offered for contradictions. Furthermore, the modest number of studies preclude our ability to only focus on studies that identify a clearly positive or negative association alone.

Table 7: Truth table of consolidated characteristics

**Definition of Smoking**	**Comparator**	**Full age** distribution	**Gender**	**Outcome**	**N (cases)**	**Sufficiency**	**PRI**	**Studies**
0	1	0	1	1	4	1	1	Pesko 2021(23); Wu 2022(26); Abouk 2023b(6),Pesko 2023(24)
0	1	0	0	1	1	1	1	Friedman 2015a(12)
0	1	1	0	1	1	1	1	Friedman 2022(14)
1	0	0	0	1	1	1	1	Nguyen 2021(20)
1	1	0	0	1	1	1	1	Pesko 2019(22)
1	1	1	1	1	1	1	1	Dave 2019(57)
1	0	1	0	0	1	0.658	0.49	Hallingberg 2020(15)
0	0	0	1	0	3	0.579	0.366	Beard 2022(7); Harrell 2022(16); Hawkins 2022(17)
0	0	1	1	0	3	0.54	0.445	Abouk 2023a(5); Cantrell 2020(8); Schneller 2022(25)
1	0	1	1	0	1	0.33	0	Kowitt 2022(18)
0	1	1	1	0	1	0	0	Abouk 2017(2)

Consistency/Sufficiency: A measure of the consistency of a subset relationship between the configuration of conditions and the outcome

PRI: Proportional Reduction in Inconsistency is an additional measure of consistency/sufficiency and refers to the extent in which a configuration reduces the level of inconsistency in predicting a is sufficient in triggering successful outcome, with higher values indicating greater reductions in inconsistency

In light of these concerns, we did not minimise the truth table further. Instead, we concluded that we are unable to fully explain differences in associations using QCA. Better reporting of the characteristics, the emergence of a clearer underlying theory to explain differences, and a larger number of studies would offer a rationale to revisit these analyses and examine these and other conditions in the future.

4. Associations between current EC use and subsequent smoking initiation

Three Tier 2 studies looked at associations between current vaping at baseline and subsequent smoking initiation. All found statistically significant direct associations.

Table 8. Association direction plot. Exposure: current e-cigarette use; outcome: smoking initiation at follow-up (=* statistically significant direct association; = direct association, not statistically significant). Tier 2 studies.

Study ID	Country (dataset)	Exposure	Association direction	Brief summary of contributing data
Miech 2017(58)	USA (MTF)	Past 30-day use	=*	Source: Monitoring the Future (MTF) in 2014 and 2015<br><br>Findings: among baseline never smokers, adolescents who reporting recently vaping at baseline were more likely to report past 12-month smoking at follow-up (aRR 4.78, 95% CI 1.91 to 11.96)
Niaura 2020 (59)	USA<br><br>(Truth)	Past 30--day use	=*	Source: Truth Initiative Young Adult Cohort Study (Dec 2011- July 2015)<br><br>Findings: 6 months transition probability from EC to combustible 0.114 (0.086 to 0.16); dual 0.062 (0.04 to 0.09). From non-current user to<br><br>combustible 0.054 (0.05 to 0.06); dual 0.002 (0.002 to 0.003).<br><br>3-year transition probability<br><br>from EC to combustible 0.417 (0.37 to 0.47); dual 0.068 (0.057 to 0.083). From non-current to combustible 0.258 (0.2 to 0.28); dual 0.031 (0.027 to 0.034)

				Note: though authors did not test for statistical significance, we judge this to be a statistically significant association as CI do not overlap
Yang 2022(60)	USA (H&H)	Past 6 months use	=*	Source: five consecutive, bi-annual waves of data (Fall 2013 through Fall 2015) from the Happiness and Health Study (H&H). Findings: "Use of e-cigarettes was positively associated with initiation of cigarettes (OR = 7.57; 95%CI:[5.32, 10.8])"

4.1 Associations between ever e-cigarette use and subsequent smoking initiation

A further 23 Tier 2 studies looked at associations between *ever vaping* at baseline and subsequent smoking initiation. Twenty-one of the 23 found statistically significant direct associations, one found a non-statistically significant direct association, and one found no association (Table 9).

Table 9. Association direction plot. Exposure: ever e-cigarette use; outcome: smoking initiation at follow-up (=* statistically significant direct association; = direct association, not statistically significant). Tier 2 studies.

Exposure: ever use /infrequent

Outcome: cigarette initiation

Study ID	Country (dataset)	Exposure	Association direction	Brief summary of contributing data
Barrington-Trimis 2016(61)	USA (SCCHS)	Ever use	=*	Source: Southern California Children's Health Study 2014-2016 Findings: "Cigarette initiation during follow-up was reported by 40.4% of e-cigarette users (*n* = 59) and 10.5% of never users (*n* = 16). E-cigarette users had 6.17 times (95% confidence interval: 3.30–11.6) the odds of initiating cigarettes as never e-cigarette users."

Barrington Trimis 2018a (62)	USA (SCCHS)	Ever use	=*	Source: Southern California Children's Health Study (CHS) 2014-2016.<br><br>Findings: Among never e-cigarette users at baseline, 140 of 1097 (12.7%) initiated cigarette smoking at follow up. Among ever e-cigarette users at baseline, 66 of 158 (41.7%) had initiated cigarette smoking at follow up.
Best 2017(63)	UK<br><br>(DISPLAY)	Ever use	=*	Source: Determining the Impact of Smoking Point-of-Sale Legislation Among Youth (DISPLAY) study February/March 2015 to 2016).<br><br>Findings: "Baseline e-cigarette use is a significant predictor of experimentation with cigarettes. In an unadjusted model, the OR for ever-smoking at follow-up in ever e-cigarette users versus never e-cigarette users was 4.62 (95% CI 3.34 to 6.38), giving a RR of 3.15 (95% CI 2.55 to 3.89)." Adjusted RRs show statistically significant associations (depending on what's adjusted for): 1.72 (95% CI 1.31 to 2.26), 4.09 (95% CI 2.57 to 6.52).
Conner 2018 (64)	UK (overlapping dataset with Conner 2020)	Ever use	=*	Source: Cluster randomised controlled trial of a school-based smoking initiation intervention in 20 schools in England (September–December 2015).<br><br>Findings: "Initiation of cigarette use at follow-up was predicted by having ever used e-cigarettes at baseline (table 4, model 1; OR 5.38, 95% CI 4.02 to 7.22) and remained so when controlling for covariates (table 4, model 2; OR 4.06, 95% CI 2.94 to 5.60)."
Conner 2020 (65)	UK (overlapping dataset with Conner 2018)	Ever use	=*	Source: Cluster randomised controlled trial (RCT) of a school- based intervention to prevent smoking initiation (45 schools in England with never smoking adolescents initially aged 11–12 years) (September–December 2014, September–December 2016)<br><br>Findings: "Ever smoked cigarettes at follow- up (table 3, left- hand panel) was significantly predicted by baseline ever used e- cigarettes (model 1;

				OR=4.03, p<0.001). It was attenuated but remained significant when controlling for covariates (model 2; OR=2.78, p<0.001)… Finally, in relation to regularly smoked cigarettes at follow- up, smoking was significantly predicted by baseline ever used e- cigarettes (model 1; OR=3.60, p<0.001) and was attenuated but remained significant when controlling for covariates (model 2; OR=1.27, p<0.001).”
East 2018 (66)	UK (ASH)	Ever use	=*	Source: 2016 Action on Smoking and Health Great Britain Youth longitudinal survey (April 6 and 20, 2016 -August 5 and October 7, 2016)<br><br>Findings: “Compared with baseline never e-cigarette users, ever e-cigarette users were more likely to initiate smoking at follow-up. Furthermore, respondents who escalated e-cigarette use between baseline and follow-up were also more likely to initiate smoking at follow-up compared with those who did not. In the causal mediation analysis), baseline ever e-cigarette use had a direct causal effect on smoking initiation at follow-up (odds ratio [OR] = 1.34, 95% confidence interval [CI] = 1.05–1.72, p = .018), and there was a significant total causal effect of the model (OR = 1.35, 95% CI = 1.04–1.74, p = .022).”
Epstein 2021 (67)	USA (CYDS)	Ever use	=*	Source: Community Youth Development Study (CYDS) (unclear-2016)<br><br>Findings: “using the full analytical sample, 4% of participants who did not use e-cigarettes at age 21 reported combustible cigarette use at age 23, compared to 11% of those who used e-cigarettes (OR = 3.13, CI = 1.93, 5.03). After applying IPW [inverse probability weighting to account for confounding variables], using e-cigarettes at age 21 was associated with double the odds of using combustible cigarettes 2 years later (OR = 2.16, CI = 1.23, 3.79).”
Hair 2021a (45)	USA (TRUTH)	Ever use	=	Source: Truth Longitudinal Cohort (TLC) - January – April 2017; late 2019 (September – December)

				Findings: "Compared with those who still had never used an e-cigarette, those who reported ever e-cigarette use in 2018 had significantly higher odds of … current cigarette use (aOR = 8.26, 95% CI [3.17, 21.53]), and current e-cigarette use (aOR = 9.70, 95% CI [6.41, 14.69]) one year later in 2019."
Keller-Hamilton 2021 (68)	USA (BUCKEYE)	Ever use	=*	Source: Buckeye Teen Health Study (Ohio) from January 2015 through June 2016<br><br>Findings: [study only in males] "Compared to male youth who never used e-cigarettes, those who used e-cigarettes were more than twice as likely to subsequently initiate ever cigarette smoking (RR = 2.71; 95% confidence interval [CI]: 1.89, 3.87)... They were also at increased risk of initiating current cigarette smoking (RR = 2.20; 95% CI: 1.33, 3.64)."
Kinnunen 2019 (69)	Finland<br><br>(METLOFIN)	Ever use	=*	Source: Metropolitan Longitudinal Finland (MetLoFIN), a longitudinal study of 12,248 children in the Helsinki metropolitan area of Finland. 2014-2016<br><br>Findings: "Among baseline never-smokers, experimentation with or use of nicotine e-cigarettes predicted the uptake of daily smoking at follow-up (AOR 2.92; 95% CI 1.09–7.85)"
Kintz 2020 (70)	USA<br><br>(SCCHS)	Ever use	=*	Source: Southern California Children's Health Study (2014 and 2015)<br><br>Findings: "e-cigarette use at baseline, marketing, and social environment factors were associated with cigarette initiation at follow-up in both the minimally adjusted (Ps < 0.05) and maximally adjusted (Ps < 0.05) models."
Leventhal 2015 (71)	USA	Ever use	=*	Source: Longitudinal survey of substance use and mental health among Californian high school students (fall 2013- fall 2014)

				Findings: “Baseline e-cigarette use was associated with greater likelihood of use of any combustible tobacco product averaged across the 2 follow-up periods in the unadjusted analyses (odds ratio [OR], 4.27 [95% CI, 3.19-5.71]) and in the analyses adjusted for sociodemographic, environmental, and intrapersonal risk factors for smoking (OR, 2.73 [95% CI, 2.00-3.73]). Product-specific analyses showed that baseline e-cigarette use was positively associated with combustible cigarette (OR, 2.65 [95% CI, 1.73-4.05]), cigar (OR, 4.85 [95% CI, 3.38-6.96]), and hookah (OR, 3.25 [95% CI, 2.29-4.62]) use and with the number of different combustible products used (OR, 4.26 [95% CI, 3.16-5.74]) averaged across the 2 follow-up periods.”
Loukas 2018 (72)	USA<br><br>(M-PACT)	ever use	=*	Source: First four waves of the Marketing and Promotions across Colleges in Texas project (Project M-PACT)- November 2014–February 2015 to approximately 18 months after baseline (the three subsequent waves were collected approximately every six months).<br><br>Findings: “Even after accounting for all other study variables, wave 1 ever ENDS use was associated with 1.36 greater odds of cigarette initiation”
Martinelli 2021 (73)	Netherlands and Belgium	Ever use	=*	Source: Online surveys between September 2018 and December 2019 throughout the Netherlands and Flanders.<br><br>Findings: “Among never smokers, baseline ever e-cigarette users were more likely than never users to have used combustible tobacco at 6-month (23.2% vs 5.5%; % difference=17.7; 95% CI 9.8 to 25.6) and 12-month (44.4% vs 10.8%; % difference=33.6; 95% CI 23.1 to 44.1) follow-ups."
Morgensten 2018 (74)	Germany	Ever use	=*	Source: Cohort in German states of Lower Saxony and Schleswig-Holstein (2015/2016 school year)

				Findings: “Experimental use of conventional cigarettes was at 21.6% in the group of e-cigarette consumers, while it was at 9.9% in the group of students without experience with e-cigarettes” (adjusted RR 2.18, 95% CI 1.68 to 2.83).
Ortega 2021 (75)	USA	Ever use	No association	Source: Longitudinal project examining factors contributing to emotional, behavioral, and academic outcomes in a Midwestern (USA) high school from Fall 2016 to Fall 2018.<br><br>Findings: “Initiation of e-cigarettes at baseline did not significantly predict risk for initiating traditional tobacco products use…at one-or two-year follows ups” Coefficient for EC initiation at baseline as a predictor of Tobacco initiation at one-year follow-up is 1.005, SE 0.72, p=0.163; coefficient for E-cig initiation at baseline as a predictor of Tobacco initiation at two-year follow-up is -2.388, SE 3407.27, p=0.999
Patanavanich 2022 (76)	Thailand	Ever use	=*	Source: Thailand Parental Supply and Use of Alcohol, Cigarettes & Drugs Longitudinal Study Cohort in Secondary School Students, a school- based nationally representative longitudinal survey of seventh grade students; wave 1 in 2019 (baseline) and wave 2 in 2020 (follow- up)<br><br>Findings: “Baseline never smokers who had used e- cigarettes had increased odds of having tried combustible cigarettes (OR 7.70), becoming current smokers (OR 6.43) or becoming dual users (OR 12.07) at 12- month follow- up compared with those who had never used e-cigarettes at baseline (all p<0.001). In the adjusted models, the results only remained statistically significant for ever smoking at follow- up: adjusted OR 4.44, p<0.001, and dual users: adjusted OR 5.31, p<0.001.”
Primack 2015 (77)	USA	Ever use	=*	Source: Dartmouth Media, Advertising, and Health Study (2012-2013, 2013-2014)<br><br>Findings: “those who smoked e-cigarettes at baseline had larger point estimates of progressing from non-susceptible non-smokers to cigarette

				smoking (AOR, 11.9; 95%CI, 2.1-68.7).” Data not reported re: progressing from susceptible non-smokers to cigarette smoking.
Primack 2018 (78)	USA	Ever use	=*	Source: Nationally representative probability-based online nonvolunteer access panel recruited and maintained by Growth from Knowledge (March 2013- October 2014)<br><br>Findings: “Among the 16 e-cigarette users at baseline, 6 (37.5%) initiated cigarette smoking at 18-month follow-up compared with 81 (9.0%) of 899 e-cigarette nonusers (P < .001)… Multivariable logistic regression analyses incorporating survey weights demonstrated that compared with baseline non e-cigarette smokers, baseline e-cigarette smokers had greater odds of initiating cigarette smoking (adjusted odds ratio [AOR], 6.82; 95% confidence interval [CI], 1.65-28.25).”
Treur 2018 (79)	Netherlands	Ever use	=*	Source: Two cohorts of Dutch adolescents; 2014–2015 from 19 secondary schools randomly selected across the Netherlands<br><br>Findings: “adolescents who ever used an e-cigarette with nicotine were at 11.90 higher odds of having smoked a conventional cigarette 6 months later, than those who never used an e-cigarette with nicotine (95% CI 3.36–42.11)”
Wang 2020 (79)	USA<br><br>(NHIS)	Ever use	=*	Source: National Health Interview Survey (NHIS) (2018, 2014, 2010).<br><br>Findings: “In both waves, after controlling sociodemographic factors, previous electronic cigarette smokers in 2014 (p < 0.001, OR = 10.428, 95% CI 8.502–12.790) and 2018 (p < 0.001, OR = 6.666, 95% CI 4.770–9.316) were more likely to be current smokers, compared to non-[electronic cigarette] smokers.”
Wills 2016 (80)	USA (note overlap with	Ever use	=	Source: Longitudinal school-based survey on Oahu, Hawaii (2013-2014)

	data from Wills 2017a)			Findings: "The primary structural modeling analysis, based on initial never-smokers, used an autoregressive model (entering T2 mediator values adjusted for T1 values) to test for mediational pathways in the relation between e-cigarette use at T1 and cigarette smoking status at T2. Results showed that e-cigarette use was related to all of the mediators and tests of indirect effects indicated that changes in expectancies, affiliations, and marijuana use were significant pathways in the relation between e-cigarette use and smoking onset... With the indirect pathways included in the model, the direct effect from T1 e-cigarette use to T2 smoking onset was not significant (p = .11)."
Wills 2017a (81)	USA (note overlap with data from Wills 2016)	everuse	=*	Source: Longitudinal school-based survey on Oahu, Hawaii (2013-2014)<br><br>Findings: "ORs for the likelihood of onset of smoking by T2 were significant for each level; that is, any level of T1 e-cigarette use was related to a significantly higher likelihood of onset of smoking."

4.2 Tier 2 studies - Associations between current use and subsequent smoking progression

Table 10. Association direction plot. Exposure: current e-cigarette use; outcome: smoking progression at follow-up (=* statistically significant direct association; = direct association, not statistically significant; X inverse association, not statistically significant). Tier 2 studies.

Study ID	Country (dataset)	Exposure	Association direction	Brief summary of contributing data
Chaffee 2018 (82)	USA (PATH)	Past 30 days use	=*	Source: PATH Study baseline (2013–2014) and Wave 2 (2014–2015)<br><br>Findings: "baseline e-cigarette past 30-day use statistically significantly predicted progression to past 30-day smoking (OR: 1.64; 95% CI: 1.12–2.41; P = .010)."
Miech 2017 (58)	USA (MTF)	Past 30d use	=*	Source: Monitoring the Future (MTF) in 2014 and 2015

				Findings: Among baseline ever smokers but who reported non-recent smoking at baseline, adolescents who reported recently vaping at baseline were more likely to report past 12 month smoking at follow-up (adjusted RR 2.26, 95% CI 1.22 to 4.18)
Pearson 2020 (83)	USA (PATH)	past 30d use	no association	Source: Population Assessment of Tobacco and Health (PATH) Study ((2013-2014, 2014-2015, and 2015-2016))<br><br>Findings: In ever cigarette smoking young adults, "no statistically significant associations were observed between any definition of wave 2 ENDS use and changes in either the frequency or intensity of smoking at wave 3" Directions of associations varied based on ENDS use definition and outcome. Never ENDS use versus any previous 30-day ENDS use: difference in change in cigarette smoking frequency 0.71 days smoked in the past 30 days (95% CI -0.94 to 2.37); difference in cigarettes smoked in the past 30 days 17.24 (-13.15 to 47.63).
Pokhrel 2022 (84)	USA	past 30d use	X*	Source: cohort conducted in students from two 4-year and four 2-year (community) colleges under the same university system on Oahu, Hawaii (2018-2020)<br><br>Findings: "individuals with higher initial levels of e-cigarette use were likely to show… a decreasing rate of cigarette smoking over time (B = −0.12, SE = 0.04, P < 0.01, 95% CI = −0.20 to −0.04; d = 0.12, Bayes' factor = 22.8)."

4.3 Associations between *ever use* and subsequent smoking progression

Four Tier 2 studies looked at associations between ever vaping at baseline and cigarette smoking progression at follow-up, in cohorts which also reported some smoking at baseline (Table 11). One found no association, one found a non-statistically significant direct association, and two found statistically significant direct associations.

Table 11. Association direction plot. Exposure: ever e-cigarette use; outcome: smoking progression at follow-up (=* statistically significant direct association; = direct association, not statistically significant). Tier 2 studies.

Study ID	Country (dataset)	Exposure	Association direction	Brief summary of contributing data
Chaffee 2018 (82)	USA (PATH)	Ever use	=*	Source: PATH Study baseline (2013–2014) and Wave 2 (2014–2015)<br><br>Findings: "Among baseline cigarette experimenters…having ever used e-cigarettes was positively associated with progression to established cigarette smoking in Wave 2. Compared with e-cigarette never users, e-cigarette ever users were twice as likely to report Wave 2 established smoking (19.3% vs 9.7%; P < .001) and current established smoking (15.6% vs 7.1%; P < .001) and were more likely to report past 30-day smoking (38.8% vs 26.6%; P < .001). In models adjusted for sex, age, and race and/or ethnicity, Wave 1 e-cigarette ever use (versus never use) was associated with approximately twice the odds of progression to Wave 2 established cigarette smoking (odds ratio [OR]: 2.23; 95% confidence interval [CI]: 1.55–3.21; P < .001), past 30-day smoking (OR: 1.75; 95% CI: 1.35–2.27; P < .001), and current established smoking (OR: 2.43; 95% CI: 1.55–3.80; P < .001). Associations were attenuated in fully adjusted models, but e-cigarette ever use remained a positive and statistically significant predictor of current established smoking (OR: 1.80; 95% CI: 1.04–3.12; P = .035). Associations did not reach the threshold for statistical significance for established smoking (OR: 1.57; 95% CI: 0.99–2.49; P = .055) and past 30-day smoking (OR: 1.32; 95% CI: 0.99–1.76; P = .059)."
Connor 2018(64)	UK	Ever use	=	Source: 4-year cluster randomised controlled trial of a school-based smoking initiation intervention in 20 schools in England. (September–December 2015) to (September–December 2015).<br><br>Findings: "ever use of e-cigarettes at baseline was a significant predictor of escalation of cigarette use (OR 2.16, 95% CI 1.01 to 4.62). In model 2, ever use of e-cigarettes at baseline became a non-significant predictor of escalation when controlling for covariates (OR 1.89, 95% CI 0.82 to 4.33)."
Leventhal 2016 (85)	USA	Ever use	=*	Source: longitudinal study conducted in 10 public high schools in Los Angeles County, California (fall 2014- spring 2015)<br><br>Findings: "Adjusting for baseline smoking, each increment higher on the 4-level baseline vaping frequency continuum was associated with proportionally higher odds of smoking at a greater level of frequency (odds ratio [OR], 2.17; 95% CI, 1.95-2.42) and heaviness (OR, 2.19; 95% CI, 1.85-2.58) by follow-up; associations persisted in covariate-adjusted analyses. The positive association between

				baseline vaping and follow-up smoking frequency was stronger among baseline nonsmokers (n = 2966;OR, 2.51; 95%CI, 2.30-2.75) than baseline infrequent (n = 63; OR, 1.47; 95% CI, 0.98-2.23) and frequent (n = 53; OR, 1.06; 95% CI, 0.72-1.55) smokers (P < .001 for interaction). Similar trends were found for smoking heaviness”
Selya 2018a (86)	USA	Ever use (SECAP)	no clear evidence of association (association varies based on level of nicotine dependence)	Source: Social and Emotional Contexts of Adolescent Smoking Patterns (SECASP) Study (5th year to 8th year of the cohort)<br><br>Findings: “Among participants with very low dependence (NDSS < 1.2), those who ever smoked e-cigarettes smoked slightly more frequently (by at most a coefficient (B) of 1.8 days out of the past month, 95% confidence interval [CI]: 0.2 to 3.2]), whereas among cigarette smokers with severe nicotine dependence (NDSS > 3.4), those who also ever used e-cigarettes smoked conventional cigarettes *less* frequently (by at most B = 5.6 fewer days, CI: [−8.2 to −3.1]). For those with moderate nicotine dependence (NDSS between 1.2 and 3.4), lifetime e-cigarette use had no significant effect on smoking frequency as shown by the confidence interval (CI) including zero (average B = 0.5 days, average CI: [−0.8 to 1.9).”

4.4 Associations between current e-cigarette use and smoking cessation

Seven Tier 2 studies examined associations between *current vaping* at baseline and subsequent smoking cessation, in young people who smoked at baseline (Table 12). Data were mixed: two found statistically significant associations between vaping and increased smoking cessation at follow-up (though in one of these the association was only present in those who reported using EC to quit smoking); two found statistically significant associations between vaping and decreased smoking cessation; two found no evidence of an association; and one found a non-statistically significant association between vaping and decreased smoking cessation.

Table 12. Association direction plot. Exposure: current e-cigarette use; outcome: smoking cessation at follow-up (=* statistically significant direct association; = direct association, not statistically significant; X inverse association, not statistically significant; X* inverse association, statistically significant). Tier 2 studies.

Study ID	Country (dataset)	Exposure	Association direction	Brief summary of contributing data

Huang 2016 (87)	USA<br><br>(NC YTS)	past-30 days use	=* (vaping associated with less smoking cessation)	Source: North Carolina Youth Tobacco Survey (2011, 2013)<br><br>Findings: “Among dual users [of e-cigarettes and combustible tobacco], current e-cigarette use was negatively associated with intention to quit cigarette smoking for good (relative risk ratio [RRR] = 0.51; 95% CI, 0.29–0.87) and with attempts to quit cigarette smoking in the past 12 months (RRR = 0.69; 95% CI, 0.49–0.97). Current e-cigarette smokers were less likely than those who only smoked cigarettes to have ever abstained from cigarette smoking for 6 months (RRR = 0.42; 95% CI, 0.21–0.82) or 1 year (RRR = 0.21; 95% CI, 0.09–0.51) and to have used any kind of aids for smoking cessation (RRR = 0.46; 95% CI, 0.29–0.74).”
Lin 2022 (88)	Taiwan (TAALS)	past 30 day use	No evidence of an association	Source: 2015 (baseline) and the 2017 (follow-up) waves of the Taiwan Adolescent to Adult Longitudinal Study (TAALS).<br><br>Findings: “for all current adolescent smokers, using e-cigarettes was not associated with smoking cessation as compared to current adolescent smokers who did not use e-cigarettes (aRR=0.99; CI=0.66, 1.50).”
Mantey 2017 (89)	USA (M-PACT)	past 30d use	If using for cessation:<br><br>X* (Vaping associated with more smoking cessation)<br><br>If using for other reasons:<br><br>=<br><br>(vaping associated with less smoking cessation)	Source: Waves 1–3 Marketing and Promotions across Colleges in Texas Project (Project M-PACT) (November 2014 to February 2015, October-November 2015)<br><br>Findings: “the use of e-cigarettes for cigarette smoking cessation increased odds of cigarette cessation by a factor of 1.66 (95% CI: 1.00 – 2.74), at 12-month follow-up, relative to non-e-cigarette users…no statistically significant differences in cigarette smoking cessation at 6-month (AOR: 0.72; 95% CI: 0.44 – 1.19) or at 12-month (AOR: 0.81; 95% CI: 0.50 – 1.30) follow-ups between non-e-cigarette users and those who used e-cigarettes for other reasons, adjusting for covariates.”

Saller 2022 (90)	USA<br><br>(NYTS)	past 30d use	=<br><br>(vaping associated with less smoking cessation)	Source: 2015–2018 National Youth Tobacco Survey (NYTS).<br><br>Findings: "there was no statistically significant association between … current established (aOR 0.65, 95% CI 0.41–1.03) e-cigarette use and past 30-day abstinence from cigarette smoking."
Selya 2018a (86)	USA<br><br>(SECASP)	past 30d use	No evidence of an association	Source: Social and Emotional Contexts of Adolescent Smoking Patterns (SECASP) Study (5th year to 8th year of the cohort)<br><br>Findings: "Among those who reported using e-cigarettes to quit smoking, the frequency of recent e-cigarette use was not associated with future cigarette smoking frequency, as indicated by the confidence band overlapping 0 throughout the range of nicotine dependence (average B = 0.1 days out of the past month, CI: −0.2 to 0.4]). On the other hand, among those who did not use e-cigarettes to quit smoking, recent e-cigarette use was associated with more frequent smoking for those at very low (NDSS < 1.2, by at most B = 1.1 days out of the past month, CI: 0.2 to 2.0) and high (NDSS between 3.1 and 3.8, by on average B = 0.9 days, CI: 0.2 to 1.6) levels of nicotine dependence"
Snow 2018 (91)	USA<br><br>(WATI)	past 30d use	X* (vaping associated with more smoking cessation)	Source: Web-assisted tobacco intervention (WATI) trial (follow up 12 months after baseline. Baseline date unclear)<br><br>Findings: "At 6-months, baseline e-cigarette users were more likely to report cessation of traditional cigarettes compared to non-users (OR 1.39, 95% CI 1.002–1.92)"
Yang 2022 (60)	USA<br><br>(H&H)	past 6 months use	=* (vaping associated with less smoking cessation)	Source: Five consecutive, bi-annual waves of data (Fall 2013 through Fall 2015) from the Happiness and Health Study (H&H).<br><br>Findings: "Use of e-cigarettes was …negatively associated with cigarette abstention (OR = 0.58; 95%CI:[0.33, 0.99])"

4.3 Associations between ever e-cigarette use and smoking cessation

The two Tier 2 studies examining *ever vaping* were similarly mixed: one found a non-statistically significant association with increased smoking cessation and the other found a non-statistically significant association with decreased smoking cessation (Table 13).

Table 13. Association direction plot. Exposure: ever e-cigarette use; outcome: smoking cessation at follow-up (= direct association, not statistically significant; X inverse association, not statistically significant). Tier 2 studies.

Study ID	Country (dataset)	Exposure	Association direction	Brief summary of contributing data
Saller 2022 (90)	USA<br><br>(NYTS)	Ever use	X (vaping associated with more smoking cessation)	Source: 2015–2018 National Youth Tobacco Survey (NYTS).<br><br>Findings: “there was no statistically significant association between prior established (aOR 1.56, 95% CI 0.96–2.56) …e-cigarette use and past 30-day abstinence from cigarette smoking.”
Wang 2017 (92)	Hong Kong	Ever use	= (vaping associated with less smoking cessation)	Source: Youth Quitline in Hong Kong (2014-2015, 6 months after baseline)<br><br>Findings: “31 (16.4%, 95% CI: 11.1 to 21.7%) participants reported abstinence at the 6-month follow-up with a lower PPA [point prevalence of abstinence] for the past 7 days for e-cig users than that for nonusers (13.4 vs. 20.8%). E-cig users had nonsignificant lower odds of quitting (adjusted OR: 0.56, 95% CI: 0.24 to 1.35)”

5 Other measures of smoking behaviour

A further group of individual-level studies provided data on smoking behaviour at follow-up, but did not provide sufficient information for us to categorise the outcome data as initiation, progression, or cessation, most commonly because smoking status at baseline was not specified. These are summarized below. Overall, most studies showed that vaping was directly associated with increased smoking at follow-up.

5.1 Associations between current vaping and other dimensions of cigarette use at follow-up

Four tier 1 studies, all judged to be at serious risk of bias, provided data on associations between current vaping at baseline and other dimensions of cigarette use at follow up (Table 19). Two found statistically significant direct associations, one found a direct association that was not statistically significant, and the fourth found different patterns based on frequency of EC use.

Table 14. Association direction plot. Exposure: current e-cigarette use; outcome: other dimensions of smoking behaviour at follow-up (=* statistically significant direct association; = direct association, not statistically significant; X* inverse association, statistically significant). Tier 1 studies.

Study ID	Country (dataset)	Risk of bias overall	Exposure	Association direction	Brief summary of contributing data
Aleyan 2019 (33)	Canada **(COMPASS)**	Serious	Past 30-day	=*	Source: Three waves of the COMPASS study (2014–2015, 2015-2016 and 2016–2017).<br>Findings: Past 30 day e-cigarette use at W1 predicted current tobacco use at W2 (OR= 1.54; 95% CI = 1.37,1.74). Past 30-day e-cigarette users at W2 had 1.18 times higher odds of being tobacco users at W3 (95% CI = 1.08–1.29). Smoking status not clear at baseline.
Han 2023 (47)	USA	Serious	Past 30-day	=	Source: Waves 1-5 (2013-2019) of the Population Assessment of Tobacco and Health (PATH) study.

	**(PATH)**				Findings: Compared with not escalated ENDS use, escalated ENDS use was not associated with the use/misuse of cigarettes (adjusted OR = 1.15; 95% CI = 0.87-1.52; p=0.333). Smoking status not clear at baseline. Taken from Supplemental table 2.
Osibogun 2020 (50)	USA<br><br>**(PATH)**	Serious	Past 30 day use	=*	Source: Waves 1-3 of the Population Assessment of Tobacco and Health (PATH) Study (2013-2016)<br><br>Findings: "For every unit increase in the number of days of e-cigarette use at baseline, there was an increase in the number of days of cigarette smoking by 0.4 (b=0.4, 95% CI=0.1, 0.7) in the 1-year progression"
Stanton 2019 (93)	USA<br><br>**(PATH)**	Serious	Past 30-day	X* (1-5 days ENDS use)<br><br>= (6+ ENDS use days)	Source: 2013-2014 (W1) and 2014-2015 (W2) waves of the Population Assessment of Tobacco and Health (PATH) study to examine bidirectional associations between ENDS use and cigarette use among 12-17 year olds.<br><br>Findings: Among ever smokers, "1–5 days ENDS use in the past month at W1 compared with W1 ever, no past-30-day ENDS use was associated with a decrease of 2.64 cigarette smoking days in past-30-day smoking at W2 (95% CI=-4.96, -0.32; p=0.03). W1 6+ day ENDS users did not show a decrease in frequency of cigarette smoking between W1 and W2 compared to 1–5 days ENDS users (beta=3.24, 95% CI=-0.79, 7.27, p=0.11)."

Of the nine tier 2 studies that looked at associations between *current vaping* and other dimensions of cigarette use at follow-up, five found a statistically significant direct association, three found a direct association that was not statistically significant, and one found no evidence of an association but did not report further details (Table 20).

Table 15. Association direction plot. Exposure: current e-cigarette use; outcome: other dimensions of smoking behaviour at follow-up (=* statistically significant direct association; = direct association, not statistically significant). Tier 2 studies.

Study ID	Country (dataset)	Exposure	Association direction	Brief summary of contributing data
Barrington-Trimis 2020 (94)	USA (SCCHS)	Ever use,	=*	Data from Southern California Children's Health Study from 2015 to 2016 (baseline) and 2016 to 2017 (follow-up).<br><br>Findings: "past-30-day e-cigarette users at baseline smoked an average of 5.42 (95% CI: 2.56–11.5) times as many cigarettes [as baseline never e-cigarette users] at follow-up in adjusted models."
Beck 2022 (95)	USA<br><br>(PATH)	past 30d use	No association	Data from the Population Assessment of Tobacco and Health (PATH) study (2013-2018).<br><br>Findings: "Past 30-day e-cigarette use during adolescence was not associated with either cigarette or e-cigarette use during the last 3 months of pregnancy among young women." No further information given.

Bold 2018 (96)	USA	past 30 day use	=*	Data from longitudinal survey from high school students in Connecticut (February/March 2015- 2016).<br><br>Findings: "there were significant reciprocal pathways between past-month e-cigarette use at each wave and future cigarette use. Specifically, individuals using e-cigarettes in the past month at wave 1 were >7 times more likely to report subsequent cigarette use at wave 2 (OR = 7.08, 95% CI = 2.34–21.42) when compared with those not using e-cigarettes. Furthermore, those using e-cigarettes in the past month at wave 2 were close to 4 times more likely to report subsequent cigarette use at wave 3 (OR = 3.87, 95% CI = 1.86–8.06) when compared with those not using e-cigarettes."
Creamer 2018 (97)	USA<br><br>(M-PACT)	past 30 day use	=*	Source: first two waves of the Marketing and Promotions Across Colleges in Texas Project (Project M-PACT)<br><br>Findings: "the odds of smoking at wave 2 were highest for those who reported wave 1 e-cigarette use and hookah use, [adjusted OR, compared to non-users] 1.50 (95% CI: 1.16, 1.94) and 1.55 (95% CI: 1.19, 2.01), respectively."
Dunbar 2019 (98)	USA	past 30 day use	=*	Source: survey of students from 16 middle schools from three districts in the Los Angeles area as part of a substance use prevention program, CHOICE (2015-2017)

				Findings: “Models revealed robust reciprocal associations between EC and cigarette use, such that more frequent EC use at one time predicted more frequent cigarette use at the subsequent time, and vice versa. Between-person analyses showed associations between shared risk factors and both EC and cigarette use. However, shared risk factors did not predict frequency of subsequent EC and cigarette use in within-person analyses.”
Meng 2022 (99)	USA<br><br>(CHIS)	past 30 days use	=*	Source: California Health Interview Survey (CHIS) 2017–2018.<br><br>Findings: “using e-cigarettes (AOR = 5.25, 95% CI = 2.21, 12.50) was associated with higher odds of smoking cigarettes.” (smoking status at baseline unclear)
Selya 2018b (100)	USA<br><br>(SECASP)	past 30 day use	=	Source: Social and Emotional Contexts of Adolescent Smoking Patterns (SECASP) Study (2011-2014)<br><br>Findings: “e-cigarette use was associated weakly and non-significantly with later smoking behavior, both directly (β = 0.021, P = 0.081) and mediated through nicotine dependence (β = 0.005, P = 0.693)”
Spindle 2017 (101)	USA<br><br>(S4S)	past 30d use	=	Data from Spit for Science (S4S) project (2014-2015)

				Findings: Associations with current cigarette use, compared to never e-cigarette use at baseline, Current e-cigarette use OR 1.15 (0.15–9.06)
Unger 2016 (102)	USA (RED)	past 30d use	=	Source: project RED (2014-2015)<br><br>Findings: “After adjusting for covariates, cigarette smokers who used e-cigarettes in 2014 were not significantly more or less likely to remain cigarette smokers in 2015 (OR = 1.31, 95% CI = 0.73, 2.36).”

5.2 Associations between ever vaping and other dimensions of cigarette use at follow-up

One Tier 1 study, Stanton 2020 (103)(serious risk of bias, PATH data), evaluated associations between ever vaping and other dimensions of cigarette use at follow up. They report that among baseline ever-cigarette smokers, baseline ever EC use "did not affect change in cigarette frequency at W2 (n=1020, beta=0.31 [cigarette smoking days], 95% CI=-0.76, 1.39, p=0.57).” A further four tier 2 studies provided data; all found a statistically significant association between EC use at baseline and subsequent smoking at follow-up (Table 16).

Table 16. Association direction plot. Exposure: ever e-cigarette use; outcome: other dimensions of smoking behaviour at follow-up (=* statistically significant direct association). Tier 2 studies.

Study ID	Country (dataset)	Exposure	Association direction	Brief summary of contributing data
Barrington-Trimis 2020 (94)	USA (SCCHS)	Ever use	=*	Source: Southern California Children’s Health Study from 2015 to 2016 (baseline) and 2016 to 2017 (follow-up).

				Findings: “Participants reporting previous e-cigarette use but no use in the past 30 days at baseline smoked an average of 3.47 (95% CI: 2.46–4.91) times as many cigarettes at follow-up as baseline never e-cigarette users.”
Penzes 2018 (104)	Romania	Ever use	=*	Source: school-based, cluster randomized controlled trial designed to test a web-based multimedia program to prevent the initiation of smoking among adolescents (Nov 2014-May 2015).<br><br>Findings: “Trying e-cigarettes at baseline predicted trying conventional cigarettes (AOR = 3.57, 95%CI:1.96–6.49) and trying waterpipe (AOR = 1.51, 95%CI: 1.07–2.14) at follow-up.”
Spindle 2017 (101)	USA (S4S)	Ever use	=*	Source: Spit for Science (S4S) project (2014-2015)<br><br>Findings: Associations with current cigarette use, compared to never e-cigarette use at baseline, Ever e-cigarette use 3.30 (1.20–9.05)
Sutfin 2015 (105)	USA	Ever use	=*	Source: Smokeless Tobacco Use in College Students study (fall 2010-fall 2013)<br><br>Findings: “Results showed that trying e-cigarettes compared with not trying them was associated with increased odds of current cigarette smoking at wave 6 (AOR= 2.48; 95% CI = 1.32, 4.66).”

Finally, 13 studies met our criteria for inclusion but did not provide data that could be integrated into any association direction plots. Eleven of these used latent class and/or other forms of transition analysis to examine transitions between tobacco use states: Aleyan 2020(27); Blank 2023(106); Boyd 2020(107); Brouwer 2023(37); Gueorguieva 2020(43); Hair 2019(44); Huang 2023(48); Martinez-Loredo 2022(108); Romm 2022(109); Stanton 2020(103); Stanton 2023(110). These studies did not set out to directly compare EC users to non-EC users in regards to subsequent smoking behaviours, though these data were inherent to their analyses. Fearon 2023 (30)(tier 1) looked specifically at myBlu ECs and associations with subsequent smoking, but not at other EC products. Westling 2017 (111)(tier 2) aimed to examine the use of ECs by adolescents

over time and in relation to other substances; they reported a positive association between EC and cigarette use but the temporality of this was unclear.

6 Analyses of potential sociodemographic differences in associations (PROGRESS-Plus characteristics)

Though many studies controlled for PROGRESS-Plus characteristics in their analyses, few reported whether the associations between vaping and smoking differed within subgroups as defined by PROGRESS-Plus. Of those that did, no clear patterns emerged for rurality, race/ethnicity, income, education, or age (which we considered falling under 'PLUS' as a personal characteristic). Though there was no evidence of a difference at the population level, individual-level studies suggested vaping was more strongly associated with subsequent smoking in males than females. Seven out of the nine individual-level studies that examined associations based on measures related to susceptibility to smoking found that the associations between vaping and subsequent smoking were higher in those with lowest susceptibility at baseline. The other two individual-level studies found the opposite, and no population-level studies provided a breakdown by this category. No studies reported associations broken down by other PROGRESS-Plus categories. More detail on all of the above follows.

6.1 Gender/sex

All studies providing data on these variables reported breakdowns by gender as a binary variable (e.g. male/female; boy/girl).

Though many studies controlled for this variable in their analyses, only three **population-level** studies (5,14,17) analysed data to determine whether associations differed by gender/sex (definitions varied by study). None found evidence of statistically or clinically significant differences by gender/sex. Specifically, Abouk 2023a (5)estimated the impact of EC taxes on youth tobacco use amongst American high schoolers. They found that EC taxes were directly correlated with smoking; this association was positive in both males and females and in the image provided, CIs overlapped. Friedman 2022 (14)performed a similar analysis in 18 to 25-year-old Americans, and also found that higher EC taxes were associated with increased smoking; again, this association was positive in both males and females and CIs overlapped. Hawkins 2022(17), which looked at county-level policies in Massachusetts, found no impact overall or by gender on cigarette smoking in 14 to 18-year olds following legislation prohibiting vaping in smoke-free restaurants.

Two **individual-level,** tier 1 studies analysed associations by gender/sex. Both used PATH data with partial but not complete overlap, and both found young people who reported vaping at baseline were more likely to go on to report smoking at follow-up, and that this was more pronounced in boys/males than girls/females. Duan 2021(40) found that past 30-day EC use at baseline was statistically significantly positively associated with cigarette smoking at follow-up waves (adjusted odds ratio (aOR) 3.90, 95% CI 2.51 to 6.08), with a stronger effect for boys (aOR = 6.17, 95% CI: 2.43–

15.68) than for girls (aOR = 1.10, 95% CI: 0.14–8.33). Sun 2023 (112)reported that the association between baseline EC use and sustained cigarette use at follow-up was less pronounced in females than males regardless of measure used, but that the difference was not statistically significant (p=0.79).

6.2 Place of residence (urban/rural)

Two **population-level** studies analysed associations by place of residence, with no clear patterns between them. Pesko 2019 (22)found the implementation of EC minimum legal sale ages (MLSAs) laws in the US was associated with increases in smoking in pregnant teens (0.2% increase; p<0.05) and that this was driven by smoking in rural pregnant teens (0.6% increase, p<0.05). Kowitt 2022(18) evaluated the impact of an EC tax in Malaysia; overall, they found that cigarette use also declined, but that in people who smoked and reduced vaping following the tax, cigarette use increased. Reductions in cigarette use were more pronounced in suburban (OR 0.81, 95% CI 0.51 to 1.28) and rural (OR 0.72, 95% CI 0.32 to 1.62) populations than in urban populations (reference category). Cigarettes per day were reduced in suburban compared to urban populations, but increased in rural compared to urban populations (OR 0.66, p=0.86 and OR 4.76, p=0.43, respectively). However, in all cases CIs (where given) included the possibility of no difference.

No individual-level studies contributed data on place of residence.

6.3 Race and ethnicity

Two **population-level** studies analysed data by race/ethnicity; one found the association was stronger in black participants, the other found the association was stronger in white participants.

- As noted above, Pesko 2019 (22)found that the positive association between EC MLSAs and smoking in pregnancy wasdriven by increases in rural populations. They broke the rural subgroup down further by ethnicity and found that the increase was most pronounced in black pregnant teens (11.1% of the mean smoking participation rate, p>0.10) compared to white pregnant teens (4.4% of the mean, p<0.10), though the estimate for black teens is imprecise as there were more white rural teens than black rural teens. There was no evidence of an impact on rural Hispanic pregnant teens (evidence from graph). Breakdowns were not provided for urban/suburban groups.
- Abouk 2023a (5) found that EC taxes increased smoking for most groups studied, but that it was strongest in white teens compared to non-White teens.

One **individual-level** tier 1 study reported data relevant to this variable. Sun 2023 (112)reported that the association between baseline EC use and sustained smoking at follow up was statistically significantly lower in black participants than in Hispanic and white participants (p=0.002).

One further individual-level tier 2 study reported data relevant to this variable. Stokes 2021 (113)stratified analyses between vaping and smoking by racial and ethnic groups and reported that 20.6% of cigarette initiation was attributable to EC use among white youth and 21.6% among Hispanic youth, while only 3.5% was attributable to EC use among black youth.

6.4 Socio-economic status

Only one **population-level** study analysed data by socio-economic status. Kowitt 2022(18) evaluated the impact of an EC tax in Malaysia; overall they found that cigarette use also declined, but that in people who smoked and reduced vaping following the tax, cigarette use increased. They analysed correlates of change in cigarette use status and cigarettes per week. No statistically significant differences were detected based on level of education. However, compared withthose in the lowest income bracket (USD<103), those making USD103-240 and those making USD240-480 had higher odds of an increase in smoking prevalence following the tax (OR 1.74, 95% CI 1.05 to 2.88 and OR 2.16, 95% CI 1.19 to 3.92, respectively). There was no clear difference for this outcome when comparing the highest income bracket (USD>480) to the lowest (OR 0.74, 95% CI 0.23 to 2.35). Among people who smoked, there were no clear differences by income bracket in terms of cigarettes per day. Only one individual-level tier 1 study analysed associations using a measure of socioeconomic status. Sun 2023(112) found no statistically significant differences in associations between baseline ECuse and sustained smoking at follow up by annual household income (p=0.42); but found the association was stronger in those whose highest parental education was some college or less (p=0.03).

6.5 Age

Only one **population-level** study analysed data by age. Abouk 2023a (5)found that EC taxes increased smoking for most groups studied, but that it was strongest in older teens compared with younger teens. One **individual-level** tier 2 study evaluated associations by age. Lin 2022(88) used data from the Taiwan Adolescent to Adult Longitudinal Survey to test associations between vaping and smoking cessation in adolescents. They broke down data by stage in the school system (a proxy measure for age); in all age groups, EC use was not statistically significantly associated with smoking cessation. The adjusted risk ratio (aRR) was lower in junior high students compared withhigh school students (aRR 0.72, 95% CI 0.41 to 1.28 and aRR 1.06, 95% CI 0.66 to 1.72, respectively). No individual-level studies provided data relevant to this indicator.

6.6 Categories relating to smoking and other drug use behaviours

No population-level studies provided data relevant to this indicator. Nine individual-level studies (three tier 1, six tier 2) provided relevant data.

Of the three tier 1 studies, two found that the association between vaping and subsequent smoking was strongest in those judged to be less susceptible to smoking at baseline. Berry 2019(35) found that the association between prior EC use and cigarette initiation was stronger in those judged to be at lower risk of smoking (categorized using nine variables relating to risky behaviours, sensation seeking personality traits, and cigarette susceptibility). In those judged to be at an intermediate or high risk of smoking, the OR for the association between EC use and current tobacco use was 2.16 (95% CI 1.23 to 3.79), compared to 10.36 (95% CI 3.1 to 34.5) in the low-risk group (p value for interaction 0.03). Similarly, Owotomo 2020(114) reported that among non-smoking adolescents who intended to smoke conventional cigarettes at baseline, EC use was not statistically significantly associated with smoking at follow-up (adjusted OR comparing those using EC with non-users at baseline, 1.57, 95% CI 0.94 to 2.63), whereas for those who did not have an intention to smoke, EC users had 4 times higher odds of smoking at follow up than never EC users (aOR 4.62, 95% CI 2.87 to 7.42). Contrastingly, Sun 2023(112) found associations between baseline EC use and sustained smoking at follow up were statistically significantly stronger for all measures relating to smoking susceptibility and other drug use tested (for all, $p<0.001$), including: family tobacco use; exposure to secondhand smoke in the home; peer cigarette use; ever use of other tobacco products at baseline; susceptibility to cigarettes; alcohol and cannabis use in the past 12 months; and higher scores related to internalizing and externalizing problems.

Of the six tier 2 individual-level studies, five found associations between vaping and subsequent smoking were stronger in people with measures related to lower susceptibility to smoke at baseline. Morgensten 2018(74) used data from 2186 tenth graders in Germany, and found that in baseline never cigarette users, the relative risk of experimenting with cigarettes six months later was 2.2 times higher in people who reported EC use at baseline (95% CI 1.65 to 2.83). This association was stronger among participants with low sensation seeking scores and with no experience of alcohol intoxication (estimates in these populations not provided, but interactions statistically significant at $p=0.05$). Selya 2018a(86) used data from 586 American adolescents who reported smoking at baseline to investigate associations between vaping and smoking reduction. They found that associations varied by level of nicotine addiction (as measured via the Nicotine Dependence Syndrome Scale Score). Among those with the lowest level of nicotine dependence (NDSS<1.2), those who had ever used EC smoked slightly more than those who had not ever used EC (beta 1.8 days of past month, 95% CI 0.2 to 3.2). In those with the highest level of dependence (NDSS>3.4), the pattern was reversed; those who had ever used EC smoked regular cigarettes less frequently (beta -5.6 days of past month, 95% CI -8.2 to -3.1). Wills 2017b (115) analysed data from 1136 high school students in Hawaii and reported a statistically significantly stronger association between vaping and subsequent smoking onset in adolescents with lower levels of rebelliousness, lower willingness to smoke, and higher levels of parental support ($p=0.01$). For those classed by authors in the lowest (10th) centile for propensity to smoke, the OR for the relationship between vaping and subsequent smoking (compared to never vapers) was 2.23

(95% CI 1.57 to 4.17), whereas in the highest (90th) centile for propensity to smoke, the corresponding OR was 1.32 (95% CI 1.19 to 1.47). Loukas 2018 (72) analysed data from 2558 Texan university students who had never smoked cigarettes at baseline. In participants only reporting ever use of ECs at baseline, EC use was positively correlated with subsequent cigarette smoking (OR 2.26, 95% CI 1.35 to 3.76), whereas in those using other non-combusted tobacco/nicotine products at baseline, the point estimate for the association between EC use and subsequent smoking was lower and CI incorporated the possibility of no difference (OR 1.13, 95% CI 0.81 to 1.58). Barrington-Trimis 2016(61) used data from 298 high school students in California; overall vaping was associated with smoking uptake at follow-up, but this association was stronger in those who were not classed as susceptible to smoking at initial evaluation (OR 9.69, 95% CI 4.02 to 23.4) compared to those who were classed as susceptible to smoking at initial evaluation (OR 2.12, 95% CI 0.79 to 5.74).

In contrast, Conner 2018 (64) used data from 2836 adolescents in England. The unadjusted OR for the association between vaping and smoking was 5.38 (95% CI 4.02 to 7.22); in the subset of participants who reported that most of their friends smoked at baseline, this rose to 8.75 (95% CI 3.68 to 20.8). In terms of esclatation of smoking, significant effects were not found for attitudes or intentions to smoke, and no other moderators were statistically significant (data not shown).

7 Categories relating to e-cigarette use characteristics (beyond frequency)

Conner 2021 (116) analysed data from the same group of participants as Connor 2018 (64), but focussed on age of EC initiation. They reported that adolescents reporting first using EC at age 13–14 years had higher rates of subsequently initiating cigarette use than adolescents reporting first using EC at age 14–15 years. Creamer 2019 (117) (tier 2, data from college students in Texas, USA) analysed associations between EC characteristics in people using EC at baseline, in relation to subsequent smoking, and found no statistically significant association with device type (p>0.06). Goldenson 2017 (118) analysed associations between nicotine levels in 181 young people who reported vaping at baseline, and subsequent cigarette smoking (tier 2, California high school students). They report that each successive increase in nicotine concentration (none to low, low to medium, and medium to high) vaped was associated with a 2.26 (95% CI, 1.28-3.98) increase in the odds of frequent (vs no) smoking at follow-up after adjustment for baseline frequency of smoking and vaping and other relevant covariates.

References

1. Foxon F, Selya AS. Electronic cigarettes, nicotine use trends and use initiation ages among US adolescents from 1999 to 2018. Addiction (Abingdon, England). 2020;115(12):2369–78.

2. Abouk R, Adams S. Bans on electronic cigarette sales to minors and smoking among high school students. J Health Econ. 2017;54(8410622, jhe):17–24.

3. Gao W, Sanna M, Chuluunbaatar E, Tsai MK, Levy DT, Wen CP. Are e-cigarettes reviving the popularity of conventional smoking among Taiwanese male adolescents? A time-trend population-based analysis for 2004-2017. Tob Control [Internet]. 2021 Mar 1;30(2):132. Available from: http://tobaccocontrol.bmj.com/content/30/2/132.abstract

4. Shahab L, Beard E, Brown J. Association of initial e-cigarette and other tobacco product use with subsequent cigarette smoking in adolescents: a cross-sectional, matched control study. Tob Control [Internet]. 2021 Mar 1;30(2):212. Available from: http://tobaccocontrol.bmj.com/content/30/2/212.abstract

5. Abouk R, Courtemanche C, Dave D, Feng B, Friedman AS, Maclean JC, et al. Intended and unintended effects of e-cigarette taxes on youth tobacco use. J Health Econ. 2023;87(8410622, jhe):102720.

6. Abouk R, Adams S, Feng B, Maclean JC, Pesko MF. The effect of e-cigarette taxes on pre-pregnancy and prenatal smoking. Journal of Policy Analysis and Management [Internet]. 2023 Sep 1;42(4):908–40. Available from: https://doi.org/10.1002/pam.22485

7. Beard E, Brown J, Shahab L. Association of quarterly prevalence of e-cigarette use with ever regular smoking among young adults in England: a time-series analysis between 2007 and 2018. Addiction (Abingdon, England). 2022;117(8):2283–93.

8. Cantrell J, Huang J, Greenberg MS, Xiao H, Hair EC, Vallone D. Impact of e-cigarette and cigarette prices on youth and young adult e-cigarette and cigarette behaviour: evidence from a national longitudinal cohort. Tob Control. 2020;29(4):374–80.

9. Creamer MR, Dutra LM, Sharapova SR, Gentzke AS, Delucchi KL, Smith RA, et al. Effects of e-cigarette use on cigarette smoking among U.S. youth, 2004-2018. Prev Med (Baltim). 2021;142:106316.

10. Dutra LM, Glantz SA. E-cigarettes and National Adolescent Cigarette Use: 2004–2014. Pediatrics [Internet]. 2017 Feb 1;139(2):e20162450. Available from: https://doi.org/10.1542/peds.2016-2450

11. Dutra LM, Glantz SA, Arrazola RA, King BA. Impact of E-Cigarette Minimum Legal Sale Age Laws on Current Cigarette Smoking. Journal of Adolescent Health [Internet]. 2018;62(5):532–8. Available from: https://www.sciencedirect.com/science/article/pii/S1054139X17309047

12. Friedman AS. How does electronic cigarette access affect adolescent smoking?. J Health Econ. 2015;44(8410622, jhe):300–8.

13. Friedman AS. Essays in health economics: Understanding risky health behaviors. Dissertation Abstracts International Section A: Humanities and Social Sciences. 2015;75(10-A(E)):No-Specified.

14. Friedman AS, Pesko MF. Young adult responses to taxes on cigarettes and electronic nicotine delivery systems. Addiction (Abingdon, England). 2022;(bm3, 9304118).

15. Hallingberg B, Maynard OM, Bauld L, Brown R, Gray L, Lowthian E, et al. Have e-cigarettes renormalised or displaced youth smoking? Results of a segmented regression analysis of repeated cross sectional survey data in England, Scotland and Wales. Tob Control. 2020;29(2):207–16.

16. Harrell MB, Mantey DS, Chen B, Kelder SH, Barrington-Trimis J. Impact of the e-cigarette era on cigarette smoking among youth in the United States: A population-level study. Prev Med (Baltim). 2022;(pm4, 0322116):107265.

17. Hawkins SS, Kruzik C, O'Brien M, Levine Coley R. Flavoured tobacco product restrictions in Massachusetts associated with reductions in adolescent cigarette and e-cigarette use. Tob Control. 2021;(clu, 9209612).

18. Kowitt SD, Anshari D, Orlan EN, Kim K, Ranney LM, Goldstein AO, et al. Impact of an e-cigarette tax on cigarette and e-cigarette use in a middle-income country: A study from Indonesia using a pre-post design. BMJ Open [Internet]. 2022;12(5):e055483-. Available from: http://bmjopen.bmj.com/content/early/by/section

19. Levy DT, Warner KE, Cummings KM, Hammond D, Kuo C, Fong GT, et al. Examining the relationship of vaping to smoking initiation among US youth and young adults: a reality check. Tob Control [Internet]. 2019 Nov 1;28(6):629. Available from: http://tobaccocontrol.bmj.com/content/28/6/629.abstract

20. Nguyen H V, Bornstein S. Changes in adults' vaping and smoking behaviours associated with aerosol-free laws. Tob Control. 2021;30(6):644–52.

21. Pesko MF, Hughes JM, Faisal FS. The influence of electronic cigarette age purchasing restrictions on adolescent tobacco and marijuana use. Prev Med (Baltim). 2016;87(pm4, 0322116):207–12.

22. Pesko MF, Currie JM. E-cigarette minimum legal sale age laws and traditional cigarette use among rural pregnant teenagers. J Health Econ. 2019;66(8410622, jhe):71–90.

23. Pesko MF, Warman C. Re-exploring the early relationship between teenage cigarette and e-cigarette use using price and tax changes. Health Econ. 2021;(bvq, 9306780).

24. Pesko MF. Effects of e-cigarette minimum legal sales ages on youth tobacco use in the United States. J Risk Uncertain [Internet]. 2023;66(3):261–77. Available from: https://doi.org/10.1007/s11166-022-09402-y

25. Schneller LM, Kasza KA, Hammond D, Bansal-Travers M, O'Connor R, Hyland A. E-cigarette and tobacco product use among NYS youth before and after a state-wide vaping flavour restriction policy, 2020-2021. Tob Control. 2022;31(Suppl 3):s161–6.

26. Wu DC, Essue BM, Jha P. Impact of vaping introduction on cigarette smoking in six jurisdictions with varied regulatory approaches to vaping: an interrupted time series analysis. BMJ Open. 2022;12(5):e058324-.

27. Aleyan S, Hitchman SC, Ferro MA, Leatherdale ST. Trends and predictors of exclusive e-cigarette use, exclusive smoking and dual use among youth in Canada. Addictive behaviors. 2020;109(2gw, 7603486):106481.

28. Lozano P, Barrientos-Gutierrez I, Arillo-Santillan E, Morello P, Mejia R, Sargent JD, et al. A longitudinal study of electronic cigarette use and onset of conventional cigarette smoking and marijuana use among Mexican adolescents. Drug Alcohol Depend. 2017;180(ebs, 7513587):427–30.

29. Do EK, Tulsiani S, Vallone DM, Hair EC. Transitions in Frequent to Daily Tobacco and Nicotine Use among Youth and Young Adults. Subst Use Misuse. 2022;57(11):1681–7.

30. Fearon IM, Seltzer RGN, Houser TL, Tope A, Cahours X, Verron T, et al. Curiosity and intentions to use myblu e-cigarettes and an examination of the "gateway" theory: Data from cross-sectional nationally representative surveys. Drug Test Anal. 2023;(101483449).

31. Lee P, Fry J. Investigating gateway effects using the PATH study. F1000Res. 2019;8(101594320):264.

32. Kasza KA, Edwards KC, Tang Z, Stanton CA, Sharma E, Halenar MJ, et al. Correlates of tobacco product initiation among youth and adults in the USA: findings from the PATH Study Waves 1-3 (2013-2016). Tob Control. 2020;29(Suppl 3):s191–202.

33. Aleyan S, Gohari MR, Cole AG, Leatherdale ST. Exploring the Bi-Directional Association between Tobacco and E-Cigarette Use among Youth in Canada. Int J Environ Res Public Health. 2019;16(21).

34. Aleyan S, Ferro MA, Hitchman SC, Leatherdale ST. Does having one or more smoking friends mediate the transition from e-cigarette use to cigarette smoking: a longitudinal study of Canadian youth. Cancer Causes Control. 2021;32(1):67–74.

35. Berry KM, Fetterman JL, Benjamin EJ, Bhatnagar A, Barrington-Trimis JL, Leventhal AM, et al. Association of Electronic Cigarette Use With Subsequent Initiation of Tobacco Cigarettes in US Youths. JAMA Netw Open. 2019;2(2):e187794-.

36. Barrington-Trimis JL, Bello MS, Liu F, Leventhal AM, Kong G, Mayer M, et al. Ethnic Differences in Patterns of Cigarette and E-Cigarette Use Over Time Among Adolescents. J Adolesc Health. 2019;65(3):359–65.

37. Brouwer AF, Jeon J, Jimenez-Mendoza E, Land SR, Holford TR, Friedman AS, et al. Changing patterns of cigarette and ENDS transitions in the USA: a multistate transition analysis of youth and adults in the PATH Study in 2015-2017 vs 2017-2019. Tob Control. 2023;(clu, 9209612).

38. Cheng HG, Largo EG, Gogova M. E-cigarette use and onset of first cigarette smoking among adolescents: An empirical test of the “common liability” theory. F1000Res. 2019;8(101594320):2099.

39. Chien YN, Gao W, Sanna M, Chen PL, Chen YH, Glantz S, et al. Electronic Cigarette Use and Smoking Initiation in Taiwan: Evidence from the First Prospective Study in Asia. Int J Environ Res Public Health. 2019;16(7).

40. Duan Z, Wang Y, Huang J. Sex Difference in the Association between Electronic Cigarette Use and Subsequent Cigarette Smoking among U.S. Adolescents: Findings from the PATH Study Waves 1-4. Int J Environ Res Public Health. 2021;18(4).

41. Friedman AS, Xu S. Associations of Flavored e-Cigarette Uptake With Subsequent Smoking Initiation and Cessation. JAMA Netw Open. 2020;3(6):e203826-.

42. Glantz SA. e-Cigarettes Used by Adolescents to Try to Quit Smoking Are Associated With Less Quitting: A Cross-Sectional Analysis of the National Youth Tobacco Survey. J Adolesc Health. 2023;72(3):359–64.

43. Gueorguieva R, Buta E, Simon P, Krishnan-Sarin S, O’Malley SS. Data Visualization Tools of Tobacco Product Use Patterns, Transitions and Sex Differences in the PATH Youth Data. Nicotine Tob Res. 2020;22(10):1901–8.

44. Hair EC, Romberg AR, Niaura R, Abrams DB, Bennett MA, Xiao H, et al. Longitudinal Tobacco Use Transitions Among Adolescents and Young Adults: 2014-2016. Nicotine Tob Res. 2019;21(4):458–68.

45. Hair EC, Barton AA, Perks SN, Kreslake J, Xiao H, Pitzer L, et al. Association between e-cigarette use and future combustible cigarette use: evidence from a prospective cohort of youth and young adults, 2017-2019. Addictive behaviors. 2021;112:106593.

46. Hammond D, Reid JL, Cole AG, Leatherdale ST. Electronic cigarette use and smoking initiation among youth: a longitudinal cohort study. CMAJ. 2017;189(43):E1328–36.

47. Han DH, Elam KK, Quinn PD, Huang C, Seo DC. Within-person associations of escalated electronic nicotine delivery systems use with cigarette, alcohol, marijuana and drug use behaviors among US young adults. Addiction (Abingdon, England). 2023;118(3):509–19.

48. Huang S, Chen Q, Griffin P, Liu G, Azagba S. Longitudinal transitions in tobacco use in youth and young adults: A latent transition analysis of the population assessment of tobacco and health study from Wave 1 to 5. Addictive behaviors. 2023;138(2gw, 7603486):107548.

49. Melka A, Chojenta C, Holliday E, Loxton D. E-cigarette use and cigarette smoking initiation among Australian women who have never smoked. Drug Alcohol Rev. 2021;40(1):68–77.

50. Osibogun O, Bursac Z, Maziak W. E-Cigarette Use and Regular Cigarette Smoking Among Youth: Population Assessment of Tobacco and Health Study (2013-2016). Am J Prev Med. 2020;58(5):657–65.

51. Chen X, Yu B, Wang Y. Initiation of Electronic Cigarette Use by Age Among Youth in the U.S. Am J Prev Med [Internet]. 2017;53(3):396–9. Available from: https://www.sciencedirect.com/science/article/pii/S0749379717301587

52. Marx A, Dusa A. Crisp-Set Qualitative Comparative Analysis (csQCA), Contradictions and Consistency Benchmarks for Model Specification. Methodological Innovations Online [Internet]. 2011 Aug 1;6(2):103–48. Available from: https://journals.sagepub.com/doi/abs/10.4256/mio.2010.0037

53. Zahroh RI, Kneale D, Sutcliffe K, Vazquez Corona M, Opiyo N, Homer CSE, et al. Interventions targeting healthcare providers to optimise use of caesarean section: a qualitative comparative analysis to identify important intervention features. BMC Health Serv Res [Internet]. 2022;22(1):1526. Available from: https://doi.org/10.1186/s12913-022-08783-9

54. Harris K, Kneale D, Lasserson TJ, McDonald VM, Grigg J, Thomas J. School-based self-management interventions for asthma in children and adolescents: a mixed methods systematic review. Cochrane Database of Systematic Reviews [Internet]. 2019;(1). Available from: https://doi.org//10.1002/14651858.CD011651.pub2

55. Kneale D, Thomas J, Bangpan M, Waddington H, Gough D. Conceptualising causal pathways in systematic reviews of international development interventions through adopting a causal chain analysis approach. J Dev Effect [Internet]. 2018 Oct 2;10(4):422–37. Available from: https://doi.org/10.1080/19439342.2018.1530278

56. Kneale D SKTJ. Critical Appraisal of Reviews Using Qualitative Comparative Analyses (CARU-QCA): a tool to critically appraise systematic reviews that use qualitative comparative analysis. In: Abstracts of the 26th Cochrane Colloquium, Santiago, Chile. Cochrane Database Syst ; 2020.

57. Dave D, Feng B, Pesko MF. The effects of e-cigarette minimum legal sale age laws on youth substance use. Health Econ. 2019;28(3):419–36.

58. Miech R, Patrick ME, O'Malley PM, Johnston LD. E-cigarette use as a predictor of cigarette smoking: results from a 1-year follow-up of a national sample of 12th grade students. Tob Control. 2017;26(e2):e106–11.

59. Niaura R, Rich I, Johnson AL, Villanti AC, Romberg AR, Hair EC, et al. Young Adult Tobacco and E-cigarette Use Transitions: Examining Stability Using Multistate Modeling. Nicotine Tob Res. 2020;22(5):647–54.

60. Yang Z, Berhane K, Leventhal AM, Liu M, Barrington-Trimis JL, Thomas DC. Modeling the longitudinal transitions of electronic cigarettes and conventional cigarettes with time-dependent covariates among adolescents. Prev Med (Baltim). 2022;164(pm4, 0322116):107294.

61. Barrington-Trimis JL, Urman R, Berhane K, Unger JB, Cruz TB, Pentz MA, et al. E-Cigarettes and Future Cigarette Use. Pediatrics. 2016;138(1).

62. Barrington-Trimis JL, Leventhal AM, Alonzo TA, Cruz TB, Urman R, Liu F, et al. Performance of cigarette susceptibility index among e-cigarette and hookah users. Drug Alcohol Depend. 2018;183(ebs, 7513587):43–50.

63. Best C, Haseen F, Currie D, Ozakinci G, MacKintosh AM, Stead M, et al. Relationship between trying an electronic cigarette and subsequent cigarette experimentation in Scottish adolescents: a cohort study. Tobacco control 2017 jul 22. 2017;

64. Conner M, Grogan S, Simms-Ellis R, Flett K, Sykes-Muskett B, Cowap L, et al. Do electronic cigarettes increase cigarette smoking in UK adolescents? Evidence from a 12-month prospective study. Tobacco Control: An International Journal. 2018;27(4):365–72.

65. Conner M, Grogan S, Simms-Ellis R, Flett K, Sykes-Muskett B, Cowap L, et al. Evidence that an intervention weakens the relationship between adolescent electronic cigarette use and tobacco smoking: a 24-month prospective study. Tob Control. 2020;29(4):425–31.

66. East K, Hitchman SC, Bakolis I, Williams S, Cheeseman H, Arnott D, et al. The Association Between Smoking and Electronic Cigarette Use in a Cohort of Young People. J Adolesc Health. 2018;62(5):539–47.

67. Epstein M, Bailey JA, Kosterman R, Rhew IC, Furlong M, Oesterle S, et al. E-cigarette use is associated with subsequent cigarette use among young adult non-smokers, over and above a range of antecedent risk factors: a propensity score analysis. Addiction (Abingdon, England). 2021;116(5):1224–32.

68. Keller-Hamilton B, Lu B, Roberts ME, Berman ML, Root ED, Ferketich AK. Electronic cigarette use and risk of cigarette and smokeless tobacco initiation among adolescent boys: A propensity score matched analysis. Addictive behaviors. 2021;114(2gw, 7603486):106770.

69. Kinnunen JM, Ollila H, Minkkinen J, Lindfors PL, Timberlake DS, Rimpela AH. Nicotine matters in predicting subsequent smoking after e-cigarette experimentation: A longitudinal study among Finnish adolescents. Drug Alcohol Depend. 2019;201(ebs, 7513587):182–7.

70. Kintz N, Liu M, Chou CP, Urman R, Berhane K, Unger JB, et al. Risk factors associated with subsequent initiation of cigarettes and e-cigarettes in adolescence: A structural equation modeling approach. Drug Alcohol Depend. 2020;207(ebs, 7513587):107676.

71. Leventhal AM, Strong DR, Kirkpatrick MG, Unger JB, Sussman S, Riggs NR, et al. Association of Electronic Cigarette Use With Initiation of Combustible Tobacco Product Smoking in Early Adolescence. JAMA. 2015;314(7):700–7.

72. Loukas A, Marti CN, Cooper M, Pasch KE, Perry CL. Exclusive e-cigarette use predicts cigarette initiation among college students. Addictive behaviors. 2018;76(2gw, 7603486):343–7.

73. Martinelli T, Candel MJJM, de Vries H, Talhout R, Knapen V, van Schayck CP, et al. Exploring the gateway hypothesis of e-cigarettes and tobacco: a prospective replication study among adolescents in the Netherlands and Flanders. Tob Control. 2021;(clu, 9209612).

74. Morgenstern M, Nies A, Goecke M, Hanewinkel R. E-Cigarettes and the Use of Conventional Cigarettes. Dtsch Arztebl Int. 2018;115(14):243–8.

75. Ortega A, Sutton M, McConville A, Fite PJ, Cushing CC. Longitudinal investigation of the bidirectional associations between initiation of e-cigarettes and other substances in adolescents. J Subst Use [Internet]. 2021;26(1):40–7. Available from: http://www.tandfonline.com/loi/ijsu20

76. Patanavanich R, Worawattanakul M, Glantz S. Longitudinal bidirectional association between youth electronic cigarette use and tobacco cigarette smoking initiation in Thailand. Tob Control. 2022;(clu, 9209612).

77. Primack BA, Soneji S, Stoolmiller M, Fine MJ, Sargent JD. Progression to Traditional Cigarette Smoking After Electronic Cigarette Use Among US Adolescents and Young Adults. JAMA Pediatr. 2015;169(11):1018–23.

78. Primack BA, Shensa A, Sidani JE, Hoffman BL, Soneji S, Sargent JD, et al. Initiation of Traditional Cigarette Smoking after Electronic Cigarette Use Among Tobacco-Naive US Young Adults. Am J Med. 2018;131(4):443.e1-443.e9.

79. Wang G, Wu L. Healthy People 2020: Social Determinants of Cigarette Smoking and Electronic Cigarette Smoking among Youth in the United States 2010-2018. Int J Environ Res Public Health. 2020;17(20).

80. Wills TA, Gibbons FX, Sargent JD, Schweitzer RJ. How is the effect of adolescent e-cigarette use on smoking onset mediated: A longitudinal analysis. Psychol Addict Behav. 2016;30(8):876–86.

81. Wills TA, Knight R, Sargent JD, Gibbons FX, Pagano I, Williams RJ. Longitudinal study of e-cigarette use and onset of cigarette smoking among high school students in Hawaii. Tob Control. 2017;26(1):34–9.

82. Chaffee BW, Watkins SL, Glantz SA. Electronic Cigarette Use and Progression From Experimentation to Established Smoking. Pediatrics. 2018;141(4).

83. Pearson JL, Sharma E, Rui N, Halenar MJ, Johnson AL, Cummings KM, et al. Association of Electronic Nicotine Delivery System Use With Cigarette Smoking Progression or Reduction Among Young Adults. JAMA Netw Open. 2020;3(11):e2015893-.

84. Pokhrel P, Kawamoto CT, Pagano I, Herzog TA. Trajectories of e-cigarette advertising exposure, e-cigarette use and cigarette smoking in a sample of young adults from Hawaii. Addiction (Abingdon, England). 2022;117(7):2015–26.

85. Leventhal AM, Stone MD, Andrabi N, Barrington-Trimis J, Sussman S, Strong DR, et al. Association of e-cigarette vaping and progression to heavier patterns of cigarette smoking. JAMA - Journal of the American Medical Association [Internet]. 2016;316(18):1918–20. Available from: http://jamanetwork.com/journals/jama/data/Journals/JAMA/935852/jld160034

86. Selya AS, Dierker L, Rose JS, Hedeker D, Mermelstein RJ. The Role of Nicotine Dependence in E-Cigarettes' Potential for Smoking Reduction. Nicotine Tob Res. 2018;20(10):1272–7.

87. Huang LL, Kowitt SD, Sutfin EL, Patel T, Ranney LM, Goldstein AO. Electronic Cigarette Use Among High School Students and Its Association With Cigarette Use And Smoking Cessation, North Carolina Youth Tobacco Surveys, 2011 and 2013. Prev Chronic Dis. 2016;13(101205018):E103-.

88. Lin LY, Chien YN, Chen YH, Shean R, Wu CY, Huang SC, et al. E-cigarettes and smoking cessation among adolescent smokers. Sci Rep. 2022;12(1):19489.

89. Mantey DS, Cooper MR, Loukas A, Perry CL. E-cigarette Use and Cigarette Smoking Cessation among Texas College Students. Am J Health Behav. 2017;41(6):750–9.

90. Saller FS, Agaku IT, Filippidis FT. Association between e-cigarette use initiated after cigarette smoking and smoking abstinence: a cross-sectional study among adolescent established smokers in the USA. Tob Control. 2022;31(3):416–23.

91. Snow E, Johnson T, Ossip DJ, Williams GC, Ververs D, Rahman I, et al. Does E-cigarette Use at Baseline Influence Smoking Cessation Rates among 2-Year College Students?. J Smok Cessat. 2018;13(2):110–20.

92. Wang MP, Li WH, Wu Y, Lam TH, Chan SS. Electronic cigarette use is not associated with quitting of conventional cigarettes in youth smokers. Pediatr Res. 2017;82(1):14–8.

93. Stanton CA, Bansal-Travers M, Johnson AL, Sharma E, Katz L, Ambrose BK, et al. Longitudinal e-Cigarette and Cigarette Use Among US Youth in the PATH Study (2013-2015). J Natl Cancer Inst. 2019;111(10):1088–96.

94. Barrington-Trimis JL, Yang Z, Schiff S, Unger J, Cruz TB, Urman R, et al. E-cigarette Product Characteristics and Subsequent Frequency of Cigarette Smoking. Pediatrics. 2020;145(5).

95. Beck DC, Boyd CJ, Evans-Polce R, McCabe SE, Veliz PT. An examination of how e-cigarette/cigarette use during adolescence is associated with future use during the third trimester of pregnancy. Subst Abus. 2022;43(1):344–8.

96. Bold KW, Kong G, Camenga DR, Simon P, Cavallo DA, Morean ME, et al. Trajectories of E-Cigarette and Conventional Cigarette Use Among Youth. Pediatrics. 2018;141(1).

97. Creamer MR, Loukas A, Clendennen S, Mantey D, Pasch KE, Marti CN, et al. Longitudinal predictors of cigarette use among students from 24 Texas colleges. J Am Coll Health. 2018;66(7):617–24.

98. Dunbar MS, Davis JP, Rodriguez A, Tucker JS, Seelam R, D'Amico EJ. Disentangling Within- and Between-Person Effects of Shared Risk Factors on E-cigarette and Cigarette Use Trajectories From Late Adolescence to Young Adulthood. Nicotine Tob Res. 2019;21(10):1414–22.

99. Y.-Y. M, Yu Y, Ponce NA. Cigarette, electronic cigarette, and marijuana use among young adults under policy changes in California. Addictive Behaviors Reports [Internet]. 2022;16((Meng, Yu, Ponce) UCLA Center for Health Policy Research, University of California, Los Angeles, Los Angeles, CA, United States(Ponce) Department of Health Policy and Management, Fielding School of Public Health, University of California, Los Angeles, Los):100459. Available from: http://www.journals.elsevier.com/addictive-behaviors-reports/

100. Selya AS, Rose JS, Dierker L, Hedeker D, Mermelstein RJ. Evaluating the mutual pathways among electronic cigarette use, conventional smoking and nicotine dependence. Addiction (Abingdon, England). 2018;113(2):325–33.

101. Spindle TR, Hiler MM, Cooke ME, Eissenberg T, Kendler KS, Dick DM. Electronic cigarette use and uptake of cigarette smoking: A longitudinal examination of U.S. college students. Addictive behaviors. 2017;67(2gw, 7603486):66–72.

102. Unger JB, Soto DW, Leventhal A. E-cigarette use and subsequent cigarette and marijuana use among Hispanic young adults. Drug Alcohol Depend. 2016;163(ebs, 7513587):261–4.

103. Stanton CA, Sharma E, Seaman EL, Kasza KA, Edwards KC, Halenar MJ, et al. Initiation of any tobacco and five tobacco products across 3 years among youth, young adults and adults in the USA: findings from the PATH Study Waves 1-3 (2013-2016). Tob Control. 2020;29(Suppl 3):s178–90.

104. Penzes M, Foley KL, Nadasan V, Paulik E, Abram Z, Urban R. Bidirectional associations of e-cigarette, conventional cigarette and waterpipe experimentation among adolescents: A cross-lagged model. Addictive behaviors. 2018;80(2gw, 7603486):59–64.

105. Sutfin EL, Reboussin BA, Debinski B, Wagoner KG, Spangler J, Wolfson M. The Impact of Trying Electronic Cigarettes on Cigarette Smoking by College Students: A Prospective Analysis. Am J Public Health. 2015;105(8):e83-9.

106. Blank MD, Romm KF, Childers MG, Douglas AE, Dino G, Bray BC. Longitudinal transitions in adolescent polytobacco use across waves 1-4 of the Population Assessment of Tobacco and Health study. Addiction (Abingdon, England). 2023;118(4):727–38.

107. Boyd CJ, Veliz PT. Latent Classes of Youths' Nicotine Use and Association with Nicotine Dependence. J Addict Med. 2020;14(6):e400–1.

108. Martinez-Loredo V, Gonzalez-Roz A, Dawkins L, Singh D, Murphy JG, MacKillop J. Is E-cigarette Use Associated With Persistence or Discontinuation of Combustible Cigarettes? A 24-Month Longitudinal Investigation in Young Adult Binge Drinkers. Nicotine Tob Res. 2022;24(7):962–9.

109. Romm KF, Childers MG, Douglas AE, Bray BC, Dino G, Blank MD. Transitions in tobacco use profiles among adolescents: Results from the Population Assessment of Tobacco and Health (PATH) study waves 3 and 4. Drug Alcohol Depend. 2022;232(ebs, 7513587):109272.

110. Stanton CA, Tang Z, Sharma E, Seaman E, Gardner LD, Silveira ML, et al. Predictors of E-cigarette and Cigarette Use Trajectory Classes from Early Adolescence to Emerging Adulthood Across Four Years (2013-2017) of the PATH Study. Nicotine Tob Res. 2023;25(3):421–9.

111. Westling E, Rusby JC, Crowley R, Light JM. Electronic Cigarette Use by Youth: Prevalence, Correlates, and Use Trajectories From Middle to High School. J Adolesc Health. 2017;60(6):660–6.

112. Sun R, Mendez D, Warner KE. Association of Electronic Cigarette Use by US Adolescents With Subsequent Persistent Cigarette Smoking. JAMA Netw Open. 2023;6(3):e234885-.

113. Stokes AC, Wilson AE, Lundberg DJ, Xie W, Berry KM, Fetterman JL, et al. Racial/Ethnic Differences in Associations of Non-cigarette Tobacco Product Use With Subsequent Initiation of Cigarettes in US Youths. Nicotine Tob Res. 2021;23(6):900–8.

114. Owotomo O, Stritzel H, McCabe SE, Boyd CJ, Maslowsky J. Smoking Intention and Progression From E-Cigarette Use to Cigarette Smoking. Pediatrics. 2020;146(6).

115. Wills TA, Sargent JD, Gibbons FX, Pagano I, Schweitzer R. E-cigarette use is differentially related to smoking onset among lower risk adolescents. Tob Control. 2016;26(5):534–9.

116. Conner M, Grogan S, Simms-Ellis R, Cowap L, Armitage CJ, West R, et al. Association between age at first reported e-cigarette use and subsequent regular e-cigarette, ever cigarette and regular cigarette use. Addiction (Abingdon, England). 2021;116(7):1839–47.

117. Creamer M, Case K, Loukas A, Cooper M, Perry CL. Patterns of sustained e-cigarette use in a sample of young adults. Addictive behaviors. 2019;92(2gw, 7603486):28–31.

118. Goldenson NI, Leventhal AM, Stone MD, McConnell RS, Barrington-Trimis JL. Associations of Electronic Cigarette Nicotine Concentration With Subsequent Cigarette Smoking and Vaping Levels in Adolescents. JAMA Pediatr. 2017;171(12):1192–9.

119. Levy DT, Warner KE, Michael Cummings K, Hammond D, Kuo C, Fong GT, et al. Examining the relationship of vaping to smoking initiation among US youth and young adults: A reality check. Tob Control. 2019;28(6).
