# Supplementary material for: Electronic cigarettes and subsequent cigarette smoking in young people: A systematic review
Source: Addiction. 2025 Jan 30;120(6):1090–111. doi: 10.1111/add.16773 (PMC12046492; doi:10.1111/add.16773)
Supplement: Supplementary file 3 — Data S3. Supporting Information. [file ADD-120-1090-s002.pdf]

Supplementary file 3 - Characteristics of included studies

Abouk R, Adams S. Bans on electronic cigarette sales to minors and smoking among high school students. Journal of Health Economics 2017; 54:17-24

Study characteristics

Methods	Design: Natural experiment (population level study)<br>Recruitment: via MTF surveys<br>Setting: school-based surveys<br>Study start date/end date: start 2007, end 2014<br>Number of datapoints: unclear<br>Primary dataset: MTF<br>Country: USA
Participants	Total N: approximately 50,000 eight, tenth, and twelfth graders from 420 public and private secondary schools in the US<br>Age: 15-18 years<br>15 year-old states without ban 0.08% states with ban 0.05%<br>16 year-old states without ban 0.95% states with ban 0.95%<br>17 year-old states without ban 97.57% states with ban 94.64%<br>18 year-old states without ban 1.36% states with ban 4.34%<br>EC use at baseline: NA<br>Gender/sex: Male states without ban 44.59%, states with ban 44.31%<br>Ethnicity/race: Black states without ban 11.37% states with ban 10.33%<br>Hispanic states without ban 12.47% states with ban 18.89%<br>Other states without ban 11.51% states with ban 17.99%<br>Measures of socioeconomic status:<br>Weekly labour income states without ban 69.3688 states with ban 60.8117<br>Mother high school states without ban 23.83% states with ban 21.34%<br>Mother some college states without ban 21.59% states with ban 20.92%<br>Mother college graduate states without ban 28.97% states with ban 27.90%<br>Mother graduate degree states without ban 12.91% states with ban 13.99%<br>Father below high school states without ban 12.70% states with ban 15.03%<br>Father high school states without ban 26.85% states with ban 24.31%<br>Father some college states without ban 17.02% states with ban 17.40%<br>Father college graduate states without ban 22.87% states with ban 22.23%
Exposures	Electronic cigarettes Use: NA<br>Details on EC devices: NA<br>Electronic cigarettes availability: complete bans, age of sales restrictions

Outcomes	Methods: "Bans were passed in 2010 in California, Minnesota, New Jersey, New Hampshire, and Utah. The years 2007–2009 give us a three year pre-treatment window for these earlier bans and 2011–2014 give us a four year post treatment window. We know the month the survey questions were posed to the student and her age in months, which allows us to exploit monthly variation in the legality of sales to minors."<br>“Comparison between states with and without bans. Effect of e-cigarette sales ban on smoking of underage high school seniors, linear probability model, 2007–2014 MTF Survey. Variables used in the model include "individual-level covariates that are measured in the MTF. They include a standard set of demographics, log weekly income from the respondent’s labor income and from other sources (allowances, etc.), and parental educational attainment."<br>Type of combustible tobacco use: cigarettes<br>Combustible tobacco use: prevalence of cigarette use, past 30 days
Study funding	Not reported
Author declarations	Not reported

Abouk R, Courtemanche C, Dave D, Feng Bo, Friedman AS, Maclean JC, et al. Intended and unintended effects of e-cigarette taxes on youth tobacco use. Journal of Health Economics 2023; 87:102720

Study characteristics

Methods	Design: Difference in differences model (population level study)<br>Recruitment: via MTF and YRBSS<br>Setting: School-based surveys<br>Study start date/end date: Start date 2014 for MTF and 2015 for YRBSS. Last time point 2019.<br>Number of datapoints: Matched policy data by quarter for MTF (2014-2019) and by year for YRBSS (2015-2019).<br>Primary dataset: MTF and YRBSS<br>Country: USA
Participants	Total N: MTF n=254,516 (highest n available), YRBSS n=600,877 (highest n available)<br>Age: Range 14-18, MTF overall: mean age 16 years, YRBSS overall: 16 years<br>EC use at baseline: Current ENDS use rates are 15.2% in MTF and 21.1% in YRBSS, with mean rates approximately 1.5 pp higher in non-treated than treated states.<br>Gender/sex: MTF: Females 51.6%, YRBSS: Females 48.9%<br>Ethnicity/race: MTF: White, non hispanic 55%; Black/African American, non-Hispanic 14.8%; Hispanic/Latino 23.7%; Other Races, non-Hispanic 6.6%. YRBSS: White, non hispanic 54.3%; Black/African American, non-Hispanic 14.8%; Hispanic/Latino 24.0%; Other Races, non-Hispanic 6.68%<br>Measures of socioeconomic status: Unemployment rate - MTF: mean=4.862 (SD=1.576). YRBSS: mean= 4.544 (SD=0.880). Poverty rates - MTF: mean= 14.200 (SD=5.219), YRBSS: mean =12.697 (SD=2.513).
Exposures	Electronic cigarettes Use: MTF asks about current ENDS use, frequent ENDS use (20 or more days over the past 30 days), ENDS initiation during the school year, ever ENDS use. YRBSS asks about ever use and frequency of past 30-day use for cigarettes, “electronic vapor product[s]” followed by example brand names marketed as nicotine ENDS (e.g., JUUL, Vuse, blu)

	Details on EC devices: YRBSS provides example brand names marketed as nicotine ENDS (e.g., JUUL, Vuse, blu)<br>Electronic cigarettes availability: ENDS taxes
Outcomes	Methods: "Between 2010 and mid-2019, ten states and two large counties adopted ENDS taxes. We use two large national surveys (MTF and the YRBSS) to estimate the impact of ENDS taxes on youth tobacco use. "<br>Adjustment for confounders: “Assess whether vaping- and smoking-responses to ENDS taxes differ across gender, age, and race. Also state "we control for unique variables available in the MTF: parental education and a respondent’s county’s urban/rural status."<br>Type of combustible tobacco use: cigarettes, cigars.<br>Combustible tobacco use: current cigarette or cigar use, smoked cigarettes in the past 30d, smoked >=one pack cigarettes /day in the past 30d, smoked cigarettes or cigars in the past 30d.
Study funding	"Research reported in this publication was supported by the National Institute on Drug Abuse of the National Institutes of Health under award number R01DA045016 (PI: Michael Pesko), R01DA039968 (PI: Dhaval Dave), and an Evidence for Action grant from the Robert Wood Johnson Foundation (grant #74869; PI: Friedman)"
Author declarations	None

Abouk R, Adams S, Feng B, Maclean JC, Pesko MF. The effect of e-cigarette taxes on pre-pregnancy and prenatal smoking. J Policy Anal Manage 2023; 42:908–940.

Study characteristics

Methods	Design: Natural experiment (population level study)<br>Recruitment: via data sets<br>Setting: National data<br>Study start date/end date: NCHS Jan 2013 to December 2019. PRAMS 2016 to 2019<br>Number of datapoints: Not reported<br>Primary datasets: Administrative birth records with geocodes provided by the National Centre for Health Statistics (NCHS). Pregnancy Risk Assessment Monitoring System data (PRAMS) for E-cigarette use.<br>Country: USA
Participants	Total N: 24,732,966 observations<br>Age: Regressions reported separately for mothers <30 years, mean age at birth 28.7 years for whole sample<br>EC use at baseline: Not reported<br>Gender/sex: All female<br>Ethnicity/race: Race: Non-Hispanic White 52%, Non-Hispanic Black 14%, Hispanic 24%<br>Measures of socioeconomic status:<br>Education status: Less than high school 14%, High school graduate 25%, Some college 29%, Bachelor or more 31%

	Primary source of payer: Medicaid 43%, Private Insurance 48%, Self-Pay 4%
Exposures	Electronic cigarettes Use: Any use, any use per month, and use and any use per month pre-pregnancy, any use and use per month third trimester.<br>Details on EC devices: Not reported<br>Electronic cigarettes availability: E-cigarette taxes between 2013 and 2019 in the US
Outcomes	Methods: "E-cigarette taxes are an active area of legislation and have important regulatory implications by proxying e-cigarette accessibility. We examine the effect of e-cigarette taxes on prepregnancy and prenatal smoking using the near-universe of births to mothers conceiving between 2013 and 2019 in the United States." "We first estimate the effects of e-cigarette taxes on prepregnancy and prenatal smoking in a repeated cross-sectional fixed-effect linear regression." "As a secondary analysis, we explore the effect of e-cigarette tax adoption that occurs during a mother's pregnancy on within-pregnancy smoking."<br>Adjustment for confounders: "We adjust for other tobacco control policies in our regressions. Specifically, at the county level we control for inflation-adjusted federal, state, and local cigarette taxes; state and county e-cigarette MLSA laws; Tobacco 21 laws covering the entire state or county; county-level share of the population covered by indoor vaping restrictions and indoor smoking restrictions in bars, restaurants, and private workplaces; and e-cigarette sales bans"<br>Type of combustible tobacco use: cigarettes<br>Combustible tobacco use: Number of cigarettes smoked per day during 3 months before pregnancy, during pregnancy and for each trimester
Study funding	"Research reported in this publication was supported by the National Institute on Drug Abuse of the National Institutes of Health under Award Number R01DA045016 (PI: Michael Pesko)."
Author declarations	"Dr. Pesko reports consulting revenue for e-cigarette tax research from Health Canada; authors have no other conflicts of interest to declare."

Aleyan S, Gohari MR, Cole AG, Leatherdale ST. Exploring the Bi-Directional Association between Tobacco and E-Cigarette Use among Youth in Canada. International Journal of Environmental Research and Public Health 2019;16(21):1-9.

Study characteristics

Methods	Design: Prospect cohort study (Individual level study)<br>Recruitment: via COMPASS<br>Setting: High school surveys<br>Study start date/end date: Wave 1 (W1): 2014–2015; Wave 3 (W3): 2016–2017]<br>Number of datapoints: 3<br>Primary dataset: COMPASS<br>Country: Canada
Participants	Total N: 6729<br>Age: Grade 9 to 12 grade 9 n= 3771 (56.0%); grade 10 n= 2958 (44.0%)

	EC use at baseline: E-cigarette users 382 (5.7%) [baseline characteristics of the linked longitudinal sample (n= 6729)]<br>Gender/sex: Female n= 3502 (52.2%); Male n= 3207 (47.8%)<br>Ethnicity/race: White n=5092(76.1%); Black n=210 (3.1%); Latin-American n=125 (1.9%); Asian n=361 (5.4%); Aboriginal n= 148 (2.2%); Other n= 759 (11.3%)<br>Measures of socioeconomic status: Weekly spending money $0 n=1506 (26.0%); $1–20 n= 2253 (44.1%); $20–100 n= 1322 (22.8%) Over $100 n=412 (7.1%)
Exposures	Electronic cigarettes Use: Past 30-days<br>Details on EC devices: Not reported<br>Electronic cigarettes availability: Not applicable
Outcomes	"A longitudinal sample of secondary students (n = 6729) attending 87 schools in Ontario and Alberta, Canada, who completed the COMPASS student questionnaire across three waves (from 2014–2015 to 2016–2017) was identified. Using cross-lagged models, the authors explored bi-directional associations between current tobacco and e-cigarette use, adjusting for relevant covariates."<br>Adjustment for confounders: "The statistical models were adjusted for baseline characteristics (e.g., gender, grade, and ethnicity), and nested structure of the data (i.e., students nested within schools). The models also accounted for having friends that smoke, weekly spending money, current cannabis use, and current binge drinking at each wave."<br>Type of combustible tobacco use: cigarettes<br>Combustible tobacco use: Past-30 days<br>Odds of current cigarette use at wave 2 or wave 3 following current e-cigarettes use at wave 1 or wave 3
Study funding	"The COMPASS study has been supported by a bridge grant from the CIHR Institute of Nutrition, Metabolism and Diabetes (INMD) through the "Obesity – Exposures to Prevent or Treat" priority funding awards (OOP-110788; awarded to SL), an operating grant from the CIHR Institute of Population and Public Health (IPPH) (MOP-114875; awarded to SL), a CIHR project grant (PJT-148562; awarded to SL), a CIHR bridge grant (PJT-149092; awarded to KP/SL), a CIHR project grant (PJT-159693; awarded to KP), and by a research funding arrangement with Health Canada (#1617-HQ-000012; contract awarded to SL). Adam Cole was funded by the Canadian Institute of Health Research (CIHR) during the time of the study. The funding sources noted above had no involvement in the study design, collection, analysis, interpretation of data and writing of the report."
Author declarations	"The authors declare no conflict of interest."
Notes	Total N represents final analytical sample.

Aleyan S, Hitchman SC, Ferro MA, Leatherdale ST. Trends and predictors of exclusive e-cigarette use, exclusive smoking and dual use among youth in Canada. Addictive Behaviors 2020; 109:106481.

Study characteristics

Methods	Design: Longitudinal cohort (Individual level study)<br>Recruitment: via COMPASS<br>Setting: High school surveys

	Study start date/end date: Wave 1 (W1): 2014–2015; Wave 3 (W3): 2016–2017]<br>Number of datapoints: 3<br>Primary dataset: COMPASS<br>Country: Canada
Participants	Total N: 5704<br>Age: 13–18 years old (grades 9 to 12)<br>EC use at baseline: Exclusive e-cigarette users n= 129 (2.33%)<br>Gender/sex: Female: 54.6%<br>Ethnicity/race: White: 72.5%<br>Measures of socioeconomic status: "26.9% reported having at least $20CAD in weekly spending money"
Exposures	Electronic cigarettes Use: Past 30-days<br>Details on EC devices: Not reported<br>Electronic cigarettes availability: Not applicable
Outcomes	Methods: "A longitudinal sample of adolescents (n=5704) who completed the three waves of COMPASS [Wave 1 (W1) 2015/16, Wave 2 (W2) 2016–17, Wave 3 (W3) 2017/18] was identified (N = 5,704). Using Multinomial logistic regression, the authors analysed whether frequency of e-cigarette use and cigarette smoking at Wave 1 predicted involvement in different usage groups at Wave 2 and Wave 3. They also explored whether changes in frequency of e-cigarette use and cigarette smoking between Wave 1 and Wave 2 predicted involvement in different usage groups at Wave 3."<br>Adjustment for confounders: "All models adjusted for sex, ethnicity, province, weekly spending money (a proxy measure for socio-economic status), having close friends who smoke, past month cannabis use and binge drinking at baseline. Model 3 also adjusted for the frequency of e-cigarette use and cigarette smoking measured at Wave 1."<br>Type of combustible tobacco use: cigarettes<br>Combustible tobacco use: Past-30 days<br>Odds of current e-cigarettes use and cigarette smoking at wave 2 following frequency of e-cigarette use in past month at baseline.
Study funding	"The COMPASS study has been supported by a bridge grant from the CIHR Institute of Nutrition, Metabolism and Diabetes (INMD) through the "Obesity – Exposures to Prevent or Treat" priority funding awards (OOP-110788; awarded to SL), an operating grant from the CIHR Institute of Population and Public Health (IPPH) (MOP-114875; awarded to SL), a CIHR project grant (PJT-148562; awarded to SL), a CIHR bridge grant (PJT-149092; awarded to KP/SL), a CIHR project grant (PJT-159693; awarded to KP), and by a research funding arrangement with Health Canada (#1617-HQ-000012; contract awarded to SL). Dr. Leatherdale is a Chair in Applied Public Health funded by the Public Health Agency of Canada (PHAC) in partnership with the Canadian Institute of Health Research (CIHR). Dr. Ferro holds the Canada Research Chair in Youth Mental Health and is supported by an Early Researcher Award from the Ministry of Research, Innovation and Science. The funding sources noted above had no involvement in the study design, collection, analysis, interpretation of data and writing of the report."
Author declarations	**"**The authors declare no conflict of interest."
Notes	Total N represents final analytical sample.

Aleyan S, Ferro MA, Hitchman SC, Leatherdale ST. Does having one or more smoking friends mediate the transition from e-cigarette use to cigarette smoking: a longitudinal study of Canadian youth. Cancer Causes Control 2021;32(1):67-74.

Study characteristics

Methods	Design: Longitudinal cohort (Individual level study)<br>Recruitment: via COMPASS<br>Setting: High school surveys<br>Study start date/end date: Year four referred to as Wave 1 (W1): 2015–2016; year six referred to as Wave 3 (W3): 2017–2018]<br>Number of datapoints: 3<br>Primary dataset: COMPASS<br>Country: Canada
Participants	Total N: 5535<br>Age: grades 9 to 12<br>EC use at baseline: Exclusive e-cigarette users n= 124 (2.3%)<br>Gender/sex: Female: n= 2940 (53.4%); Male: n= 2570 (46.6%)<br>Ethnicity/race: White: n=4029 (73.0%); Black: n=241 (4.4%); Asian n=447 (8.1%); Latin American n=181 (3.3%); Other: n=615 (11.2)<br>Measures of socioeconomic status: Weekly spending money $0: n=1245 (22.6%); $1-20: n=2020 (36.7%); $20-100: n=1143 (20.8%); over 100$: n=344 (6.3%); I don't know: 756 (13.6%)
Exposures	Electronic cigarettes Use: Past 30-days<br>Details on EC devices: Not reported<br>Electronic cigarettes availability: Not applicable
Outcomes	Methods: "A longitudinal sample of youth that participated in three waves of the COMPASS study (2015–2016 to 2017–2018) was identified (N=5,535). The product of coefficients method was used to assess whether having one or more smoking friends mediated the association between: (1) past 30-day e-cigarette use and cigarette smoking onset and (2) past 30-day e-cigarette use and subsequent dual use of e-cigarettes and cigarettes"<br>Adjustment for confounders: "All models were adjusted for demographics and behavioral covariates."<br>Type of combustible tobacco use: cigarettes<br>Combustible tobacco use: Past-30 days cigarette smoking, Past-30 days dual use<br>Regression coefficient estimates in the association between e-cigarette use and cigarette smoking/dual use.<br>Regression coefficient estimates of the indirect and direct effects of past-30-day cigarette smoking and past-30-day dual use among past 30-day e-cigarette users, via having one or more close friends who smoke.
Study funding	**"**The COMPASS study has been supported by a bridge grant from the CIHR Institute of Nutrition, Metabolism and Diabe[1]tes (INMD) through the "Obesity – Exposures to Prevent or Treat" priority funding awards (OOP-110788; awarded to SL), an operating grant from the CIHR Institute of Population and Public Health (IPPH) (MOP-114875; awarded to SL), a CIHR project grant (PJT-148562; awarded to SL), a CIHR bridge Grant (PJT-149092; awarded to KP/ SL), a CIHR project Grant (PJT-159693; awarded to KP), and by a research funding arrangement with Health Canada (#1617-HQ-000012; contract awarded to SL). The

COMPASS-Quebec project additionally benefts from funding from the Ministère de la Santé et des Services sociaux of the province of Québec, and the Direction régionale de santé publique du CIUSSS de la Capitale-Nationale. Dr. Leatherdale is a Chair in Applied Public Health funded by the Public Health Agency of Canada (PHAC) in partnership with the Canadian Institute of Health Research (CIHR). Dr. Ferro holds the Canada Research Chair in Youth Mental Health and is supported by an Early Researcher Award from the Ministry of Research, Innovation, and Science. The funding sources noted above had no involvement in the study design, collection, analysis, interpretation of data, and writing of the report."

Author declarations "The authors have no conflicts of interest to declare."

Barrington-Trimis, Jessica L; Urman, Robert; Berhane, Kiros; Unger, Jennifer B; Cruz, Tess Boley; Pentz, Mary Ann; Samet, Jonathan M; Leventhal, Adam M; McConnell, Rob. E-Cigarettes and Future Cigarette Use. Pediatrics 2016;138(1).

Study characteristics

Methods

Design: Longitudinal cohort (Individual level study)
Recruitment: via Southern California Children's Health Study (CHS)
Setting: High school surveys
Study start date/end date: First questionnaire January 2014 - June 2014 ; online follow-up questionnaire February- March 2016.
Number of datapoints: 2
Primary dataset: CHS
Country: USA

Participants

Total N: 298 (at follow up)
Age:17.4 years at baseline
EC use at baseline: for complete data (N=298)
Gender/Sex: Female 124 (41.6), Male 174 (58.4)
Ethnicity/race: Non-Hispanic white: 126 (42.3%); Hispanic white: 146(42.3%); Other: 26(8.7%)
Parental Education: ≤ 12th grade: 86 (30.4%); Some college: 100(35.3%); College degree or higher: 97(34.3%);
Baseline grade: 11: 160(53.7%); 12: 138(46.3%)

Exposures

Electronic cigarettes Use: Ever use,
Details on EC devices: Not reported
Electronic cigarettes availability: Not applicable

Outcomes

Methods: "Data on e-cigarette use were collected in 11th and 12th grade (mean age =17.4); follow-up data on tobacco product use were collected an average of 16 months later from never-smoking e-cigarette users at initial evaluation (n = 146) and from a sample of never-smoking, never e-cigarette users (n = 152) frequency matched to e-cigarette users on gender, ethnicity, and grade. Unconditional logistic regression models to evaluate the association

	between e-cigarette use at initial evaluation and subsequent cigarette use at follow-up. Odds ratios (ORs) and 95% confidence intervals (CIs) were used to estimate the odds of smoking initiation.”<br>Adjustments of confounders: "All models were adjusted for gender, ethnicity, grade and highest parental education."<br>Type of combustible tobacco use: cigarettes, cigars, other<br>Combustible tobacco use: Ever use, Past-30 days use<br>Association between e-cigarettes use and future cigarette use
Study funding	"Research reported in this publication was supported by grant P50CA180905 from the National Cancer Institute at the National Institutes of Health and the Food and Drug Administration Center for Tobacco Products. The funder had no role in the design and conduct of the study; collection, management, analysis, or interpretation of the data; or preparation, review, or approval of the manuscript. Funded by the National Institutes of Health (NIH)."
Author declarations	"The authors have indicated they have no financial relationships relevant to this article to disclose"<br>"The authors have indicated they have no potential conflicts of interest to disclose."

Barrington-Trimis JL, Leventhal AM, Alonzo TA, Cruz TB, Urman Robert, Liu F, et al . Performance of cigarette susceptibility index among e-cigarette and hookah users. Drug and Alcohol Dependence 2018; 183:43-50.

Study characteristics

Methods	Design: Longitudinal cohort (Individual level study)<br>Recruitment: via Southern California Children’s Health Study (CHS)<br>Setting: High school surveys<br>Study start date/end date: First questionnaire January 2014 – June 2014; online follow-up questionnaire February- July 2016.<br>Number of datapoints: 2<br>Primary dataset: CHS<br>Country: USA
Participants	Total N: 1266<br>Age: grades 11 or 12 at baseline<br>EC use at baseline: Not reported<br>Gender/Sex: Female n= 663; Male n= 603<br>Ethnicity/race: Non-Hispanic white: n=490; Hispanic white: n= 603; Other: n=173<br>Parental Education: High School diploma or GED or lower: n=367; Some college: n= 438; College degree or higher: n= 438; Missing n=76.<br>Baseline grade: 11: n= 748; 12: n= 518
Exposures	Electronic cigarettes Use: Ever use, Past 30-days, other<br>Details on EC devices: Not reported<br>Electronic cigarettes availability: Not applicable

Outcomes	Methods: “we used prospective data from the Southern California Children’s Health Study to evaluate the performance (sensitivity, specificity, predictive value) of a composite index assessing susceptibility to smoking, and to evaluate whether performance of the measure differed by use of e-cigarettes or hookah, or immersion in a tobacco-friendly social environment. Susceptibility to cigarette smoking was measured in 11th/12th grade (2014) among never cigarette-smokers (N = 1266); follow-up data on smoking initiation were obtained approximately 16 months later.”<br>Type of combustible tobacco use: cigarettes<br>Combustible tobacco use: Ever use, Past-30 days use<br>Association between susceptibility and subsequent cigarette use<br>Association between e-cigarette use and subsequent cigarette use
Study funding	"Research reported in this publication was supported by grant number P50CA180905 from the National Cancer Institute at the National Institutes of Health and the Food and Drug Administration (FDA) Center for Tobacco Products (CTP). The funder had no role in the design and conduct of the study; collection, management, analysis, or interpretation of the data; or preparation, review, or approval of the manuscript. Data sharing: no additional data available."
Author declarations	"We declare no conflicts of interest for any authors."

Barrington-Trimis JL Kong G, Leventhal AM, Liu F, Mayer M, Cruz TB, et al . E-cigarette Use and Subsequent Smoking Frequency Among Adolescents. Pediatrics 2018;142(6):e20180486.

Study characteristics

Methods	Design: Longitudinal cohort (Individual level study)<br>Recruitment: via The Southern California Children’s Health Study (CHS); The Happiness and Health (H&H) Study; The Yale Adolescent Survey Study (YASS); (baseline: 2013–2014; follow-up: 2014–2016).<br>Setting: high schools<br>Study start date/end date: 2013-2014/2014-2016<br>Number of datapoints: 2<br>Primary dataset: CHS, H&H and YASS<br>Country: USA
Participants	Total N: CHS (CA) N = 1553; H&H Study (CA) N = 3190; YASS (CT) N = 1404<br>Age: Not reported<br>EC use at baseline: 351 (22.7%) + 911 (29.2%) + 323 (23.1%) for 3 cohorts<br>Gender/sex: CHS (CA): Male 752 (48.4%), Female 801 (51.6%); H&H Study (CA): Male 1467 (46.0%), Female 1723 (54.0%): YASS (CT): Male 637(45.4%), Female 767 (54.6%)<br>Ethnicity/race: CHS: Non-Hispanic white 592 (38.1%), Hispanic white 758 (48.8%), Other 203 (13.1%); H&H Non-Hispanic 512 (16.0%), White Hispanic 1505 (47.2%), Other 1173 (36.8%); YASS: Non-Hispanic white 1198 (85.3%), Hispanic white 66 (4.7%), Other 140 (10.0%)<br>Measures of socioeconomic status: Not reported

Exposures	Electronic cigarettes Use: ever use; past-30 days use<br>Details on EC devices: Not reported<br>Electronic cigarettes availability: Not applicable
Outcomes	Methods: “Data were pooled from 3 prospective cohort studies in California and Connecticut (baseline: 2013–2014; follow-up: 2014–2016; N = 6258). Polytomous regression models were used to evaluate the association of baseline e-cigarette use (never or ever) with cigarette use frequency at follow-up (experimental: initiation but no past-30-day use; infrequent: 1–2 of the past 30 days; frequent: 3–5 or more of the past 30 days). Polytomous regression models were also used to evaluate transitions between baseline ever or past-30- day single or dual product use and past-30-day single or dual product use at follow-up.”<br>Adjustment for confounders: “All models were adjusted for gender, race/ethnicity, grade, and cohort by using a missing indicator when appropriate with a random effect for school “<br>Type of combustible tobacco use: cigarette<br>Combustible tobacco use: ever use, past-30 days use<br>Odds of smoking (experimentation, infrequent use, or frequent use relative to never use) associated with e-cigarette use.
Study funding	“Supported by grant P50CA180905 (Drs Barrington-Trimis, Leventhal, Cruz, and McConnell and Ms Liu) from the National Cancer Institute at the National Institutes of Health and the Food and Drug Administration Center for Tobacco Products and grants R01DA033296 (Dr Leventhal), P50DA036151 (Drs Kong and Krishnan-Sarin and Ms Mayer), and K01DA042950 (Dr Barrington-Trimis) from the National Institute on Drug Abuse at the National Institutes of Health. The funders had no role in the design and conduct of the study; collection, management, analysis, or interpretation of the data; or preparation, review, or approval of the article. Funded by the National Institutes of Health (NIH).”
Author declarations	"The authors have indicated they have no potential conflicts of interest to disclose."

Barrington-Trimis JL, Bello MS, Liu F, Leventhal AM, Kong G, Mayer M, et al . Ethnic Differences in Patterns of Cigarette and E-Cigarette Use Over Time Among Adolescents. Journal of Adolescent Health 2019;65(3):359-36

Study characteristics

Methods	Design: Longitudinal cohort (Individual level study)<br>Recruitment: via Southern California Children’s Health Study (CHS), Yale Adolescent Survey Study (YASS) and the Happiness and Health (H&H) Study<br>Setting: High school surveys<br>Study start date/end date: First questionnaire January 2014 – June 2014; online follow-up questionnaire February- July 2016.<br>Number of datapoints: 2<br>Primary dataset: CHS (CA), H&H Study (CA) and Yass (CT)<br>Country: USA
Participants	Total N: 6147<br>Age: grades 11 and 12 at baseline<br>EC use at baseline: Gender/sex: CHS -351 (22.7%); H&H study -911 (29.2%); YASS- 323 (23.1%)

	Gender/sex: CHS - Female: n= 801(51.6%); Male: n= 752 (48.4%)/ H&H study - Female: n= 1723 (54.0%); Male: n= 1467 (46.0%)/YASS- Female: n= 767(54.6%); Male: n= 637 (45.4%)<br>Ethnicity/race: CHS- Non-Hispanic white: n=592 (38.1%); Hispanic white: n=758 (48.8%); Other: n=203 (13.1%) / H&H study- Non-Hispanic white: n=512 (16.0%); Hispanic white: n=1505 (47.2%); Other: n=1173 (36.8%) / YASS- Non-Hispanic white: n=1198 (85.3%); Hispanic white: n=66 (4.7%); Other: n=140 (10.0%)
Exposures	Electronic cigarettes Use: Ever use, Past 30-days<br>Details on EC devices: Not reported<br>Electronic cigarettes availability: Not applicable
Outcomes	Methods: “Data were pooled from 3 prospective cohort studies in California and Connecticut (baseline: 2013–2014; follow-up: 2014–2016; N = 6258). Polytomous regression models were used to evaluate the association of baseline e-cigarette use (never or ever) with cigarette use frequency at follow-up (experimental: initiation but no past-30-day use; infrequent: 1–2 of the past 30 days; frequent: 3–5 or more of the past 30 days). Polytomous regression models were also used to evaluate transitions between baseline ever or past-30- day single or dual product use and past-30-day single or dual product use at follow-up.”<br>Adjustment for confunders: "All models were adjusted for gender, grade, and cohort (CHS, H&H, YASS), using a missing indicator where appropriate, with a random effect for school (H&H/YASS) or community (CHS)."<br>Type of combustible tobacco use: cigarettes<br>Combustible tobacco use: Ever use, Past-30 days cigarette use<br>Odds ratio of e-cigarette use and subsequent smoking among never baseline smokers
Study funding	**“**Supported by grant P50CA180905 (Drs Barrington-Trimis, Leventhal, Cruz, and McConnell and Ms Liu) from the National Cancer Institute at the National Institutes of Health and the Food and Drug Administration Center for Tobacco Products and grants R01DA033296 (Dr Leventhal), P50DA036151 (Drs Kong and Krishnan-Sarin and Ms Mayer), and K01DA042950 (Dr Barrington-Trimis) from the National Institute on Drug Abuse at the National Institutes of Health. The funders had no role in the design and conduct of the study; collection, management, analysis, or interpretation of the data; or preparation, review, or approval of the article. Funded by the National Institutes of Health (NIH).”
Author declarations	“The authors have indicated they have no potential conflicts of interest to disclose.”
Notes	Total N represents final analytical sample across the 3 datasets.

Barrington-Trimis JL, Yang Z, Schiff S, Unger J, Cruz TB, Urman R, et al. E-cigarette Product Characteristics and Subsequent Frequency of Cigarette Smoking. Pediatrics 2020;145(5): e20191652.

Study characteristics

Methods	Design: Longitudinal cohort (individual level study)<br>Recruitment: via Children's Health Study (CHS)<br>Setting: school<br>Study start date/end date: from 2015 to 2016 (baseline) and 2016 to 2017 (follow-up)<br>Number of datapoints: 2<br>Primary dataset: Southern California Children's Health Study<br>Country: USA
Participants	Total N: 1312<br>Age: mean 18.9 (SD 0.6)<br>EC use at baseline: N (%) Never 828 (63.1), Previous 346 (26.4), Past 30 d 138 (10.5)<br>Gender/sex: female: n=685 (52.2%); male n= 627 (47.8%)<br>Ethnicity/race:<br>Hispanic white n=635 (48.4%)<br>Non-Hispanic white n=506 (38.6%)<br>African American n=51 (3.9%)<br>Asian American n=13 (1.0%)<br>Other n=107 (8.2%)<br>Measures of socioeconomic status:<br>Highest parental education<br>less or equal than 12th grade n=391 (29.8%)<br>Some college n=459 (35.0%) College degree or higher n=386 (29.4%)
Exposures	Electronic cigarettes Use: ever use, past 30 days<br>Details on EC devices: "Participants were asked to select the type of e-cigarette device they had used most often in the past 30 days (disposable and/or cigalike; vape pen and/or penlike; or mod, mechanical mod, and/or box mod[variations of modifiable devices]), the level of nicotine used (no nicotine use or 1–3or $4–6 mg/mL) and whether they had ever dripped with an e-cigarette device (yes versus no)."<br>Electronic cigarettes availability: not applicable
Outcomes	Methods: "There is a dearth of evidence regarding the association of use of electronic cigarettes (e-cigarettes) with certain product characteristics and adolescent and young adult risk of unhealthy tobacco use patterns (eg, frequency of combustible cigarette smoking), which is needed to inform the regulation of e-cigarettes. Data were collected via an online survey of participants in the Southern California Children's Health Study from 2015 to 2016 (baseline) and 2016 to 2017 (follow-up) (*N* = 1312). We evaluated the association of binary categories of 3 nonmutually exclusive characteristics of the e-cigarette used most frequently with the number of cigarettes smoked in the past 30 days at 1-year follow-up. Product characteristics included device (vape pen and/or modifiable electronic cigarette [mod]), use of nicotine in electronic liquid (e-liquid; yes or no), and use for dripping (directly dripping e-liquid onto the device; yes or no)."<br>Adjustment for confounders: "Coadjusted for sex, race and/or ethnicity, parental education, log-transformed number of cigarettes at baseline, e-cigarette use at follow-up, and number of days of e-cigarette use in the past 30 d at baseline by using Poisson regression models."<br>Type of combustible tobacco use: cigarette

	Combustible tobacco use: ever use, past 30 days
Study funding	"Supported by the National Cancer Institute of the National Institutes of Health and the Food and Drug Administration Center for Tobacco Products (grants P50CA180905 and U54CA180905), the National Institute on Drug Abuse of the National Institutes of Health (grant K01DA042950), and the Tobacco-Related Disease Research Program (grant 27-IR-0034). The funders had no role in the design and conduct of the study; collection, management, analysis, or interpretation of the data; or preparation, review, or approval of the article. Funded by the National Institutes of Health (NIH)."
Author declarations	"Mr Urman began a position at Amgen on April 15, 2019, and did not contribute to the article after that date; the other authors have indicated they have no potential conflicts of interest to disclose."

Beard E, Brown J, Shahab L. Association of quarterly prevalence of e-cigarette use with ever regular smoking among young adults in England: a time-series analysis between 2007 and 2018. Addiction 2022;117(8):2283-2293.

Study characteristics

Methods	Design: Time–series analysis of population trends (population level study)<br>Recruitment: via Smoking Toolkit Study (STS) dataset<br>Setting: monthly survey of a representative sample of the population in England aged 16+<br>Study start date/end date: start 2007, end 2018<br>Number of datapoints: data were aggregated quarterly.<br>Primary dataset: STS<br>Country: UK
Participants	Total N: 37105<br>Age: 16-24 years<br>EC use at baseline: not introduced in 2007<br>Gender/sex: Not reported<br>Ethnicity/race: Not reported<br>Measures of socioeconomic status: Not reported
Exposures	Electronic cigarettes Use: NA<br>Details on EC devices: NA<br>Electronic cigarettes availability: prevalence of use
Outcomes	Methods: "To assess how changes in the prevalence of e-cigarette use among young adults have been associated with changes in the uptake of smoking in England between 2007 and 2018. Time–series analysis of population trends with autoregressive integrated moving average with exogeneous input (ARIMAX) analysis was used to assess the association between changes in the prevalence of e-cigarette use and prevalence of ever regular smoking as a measure of uptake. "<br>Sensitivity analyses stratified the sample into those aged 16–17 and 18–24.

	Type of combustible tobacco use: cigarettes, cigars<br>Combustible tobacco use: prevalence of ever regular smoking
Study funding	"The STS is currently primarily funded by Cancer Research UK (C1417/A14135; C36048/A11654; C44576/A19501), and has previously also been funded by Pfizer, GlaxoSmithKline and the Department of Health. J.B. and L.S. are members of SPECTRUM a UK Prevention Research Partnership Consortium (MR/S037519/1). UKPRP is an initiative funded by the UK Research and Innovation Councils, the Department of Health and Social Care (England) and the UK-devolved administrations and leading health research charities. No funders had<br>any involvement in the design of the study, the analysis or interpretation of the data, the writing of the report or the decision to submit the paper for publication. "
Author declarations	"E.B. and J.B. have received unrestricted research funding from Pfizer. E.B. and J.B. are funded by CRUK (C1417/A14135). L.S. has received honoraria for talks, an unrestricted research grant and travel expenses to attend meetings and workshops from Pfizer, and has acted as paid reviewer for grant awarding bodies and as a paid consultant for health-care companies. All authors declare there are no other relationships or activities that could appear to have influenced the submitted work."

Beck DC, Boyd CJ, Evans-Polce R, McCabe SE, Veliz PT. An examination of how e-cigarette/cigarette use during adolescence is associated with future use during the third trimester of pregnancy. Substance Abuse 2022;43(1):344-348.

Study characteristics

Methods	Design: Longitudinal cohort (Individual level study)<br>Recruitment: via Population Assessment of Tobacco and Health (PATH) study<br>Setting: survey-based<br>Study start date/end date: September 2013–December 2014/ December 2016–January2017<br>Number of datapoints: 4<br>Primary dataset: PATH<br>Country: USA
Participants	Total N: 246<br>Age: 14-17 years<br>EC use at baseline: past 30-days 5.2% (13) at wave 1<br>Gender/sex: Female 100%<br>Ethnicity/race: White 143 (62.1%), Black 52 (25.7%), Other 36 (12.2%), Non- Hispanic 161 (70.9%), Hispanic 83 (29.1%)<br>Measures of socioeconomic status: Household income $24,999 or lower 46 (18.4%), $25,000–$49,999 37 (13.7%), $50,000–$99,000 19(9.1%), $100,000 or higher 12(5%), Missing 132(53.9%)
Exposures	Electronic cigarettes Use: past-30 days use<br>Details on EC devices: NR<br>Electronic cigarettes availability: Not applicable

Outcomes	Methods: “National longitudinal data (2013–2018) from the Population Assessment of Tobacco and Health (PATH) study were used. Young adults ages 18–20 who indicated past-year pregnancy made up the analytic sample (N ¼ 246). Logistic regression was used to evaluate the association between history of past 30-day use of cigarettes/e-cigarettes during adolescence (i.e., 14–17) and later use during the third trimester of pregnancy among young women (i.e., 18–20).”<br>Adjustment of confounders: “All binary logistic regression models adjust for key covariates as they relate to nicotine/tobacco use during adolescence and young adulthood (i.e., past 30-day marijuana use at Wave 1 or 2, past 30-day alcohol use at Wave 1 or 2, wave of pregnancy, race, ethnicity, parental household income and US region).”<br>Type of combustible tobacco use: cigarette<br>Combustible tobacco use: past-30 days use<br>Odds of ever e-cigarette use in adolescence and future use during third trimester of pregnancy.
Study funding	“Supported by grants [R01 DA044157] (C. J. Boyd) and [R01CA203809] (S. E. McCabe) from the National Institutes of Health (NIH), National Institute on Drug Abuse (NIDA), and National Cancer Institute (NCI). Dana Beck is supported by the VA Office of Academic Affiliations through the VA/National Clinician Scholars Program and the University of Michigan. The contents do not represent the view of the U.S. Department of Veterans Affairs or the United States Government.”
Author declarations	“Not reported.”

Berry KM, Fetterman JL, Benjamin EJ, Bhatnagar A, Barrington-Trimis JL, Leventhal AM, et al . Association of Electronic Cigarette Use With Subsequent Initiation of Tobacco Cigarettes in US Youths. JAMA Network Open 2019;2(2):e187794.

Study characteristics

Methods	Design: Longitudinal cohort (Individual level study)<br>Recruitment: via Population Assessment of Tobacco and Health Study (PATH)<br>Setting: High school surveys<br>Study start date/end date: Wave 1 September and December 2014; Wave 3 between 2015 and 2016.<br>Number of datapoints: 3<br>Primary dataset: PATH<br>Country: USA
Participants	Total N: 6123<br>Age: 13-17 years. Mean= 13.4 (1.2) years<br>EC use at baseline: 4.8%<br>Gender/sex: Female 49.5%<br>Ethnicity/race: Non-Hispanic, white 54.1%, Non-Hispanic, black 13.9%, Hispanic 22.8%, Non-Hispanic, other 9.2%<br>Parent completed college or higher: 35.9%
Exposures	Electronic cigarettes Use: Ever use, temporal ordering of use

	Details on EC devices: Not reported<br>Electronic cigarettes availability: Not applicable
Outcomes	Methods: “Data from waves 1 through 3 of PATH was computed to multivariable logistic regression analyses to evaluate the odds of ever and current cigarette use at wave 3 as a function of prior tobacco product use (prior use of e-cigarettes, prior use of other products, or no prior tobacco use) in youth aged 12 to 15 years who had never used cigarettes, e-cigarettes or other tobacco products at wave 1.”<br>Adjustment for confounders: “We adjusted each model for wave 1 characteristics including sex, race and ethnicity, parental education, urban residence, living with a tobacco user, frequency of noticing health warnings on cigarette packages, and ability to recall a favourite tobacco advertisement. We also adjusted the models for wave 1 risk-taking behaviours, sensation-seeking personality traits, and cigarette susceptibility. We calculated predicted probabilities of ever and current cigarette use through marginal standardization using the coefficients produced by regression models.”<br>Type of combustible tobacco use: cigarettes<br>Combustible tobacco use: Ever use, Past-30 days use<br>Odds of prior e-cigarette use with subsequent cigarette initiation within 2 years of follow-up.
Study funding	“Drs Fetterman, Benjamin, Bhatnagar, and Stokes and Ms Berry were supported by grants P50HL120163 and 2U54HL120163-06 from the National Heart, Lung, and Blood Institute of the National Institutes of Health and Center for Tobacco Products. Drs Barrington-Trimis and Leventhal were supported by grants P50CA180905 and U54CA180905 from the National Cancer Institute of the National Institutes of Health. Dr Stokes reported receiving research funding from Johnson & Johnson outside of the submitted work. No other disclosures were reported"
Author declarations	“Drs Fetterman, Benjamin, Bhatnagar, and Stokes and Ms Berry were supported by grants P50HL120163 and 2U54HL120163-06 from the National Heart, Lung, and Blood Institute of the National Institutes of Health and Center for Tobacco Products. Drs Barrington-Trimis and Leventhal were supported by grants P50CA180905 and U54CA180905 from the National Cancer Institute of the National Institutes of Health. Dr Stokes reported receiving research funding from Johnson & Johnson outside of the submitted work. No other disclosures were reported"

Best C, Haseen F, Currie D, Ozakinci G, MacKintosh AM, Stead M, et al . Relationship between trying an electronic cigarette and subsequent cigarette experimentation in Scottish adolescents: a cohort study. Tobacco Control 2017;27(4):373-378.

Study characteristics

Methods	Design: Longitudinal cohort (Individual level study)<br>Recruitment: via Determining the Impact of Smoking Point-of-Sale Legislation Among Youth (DISPLAY) study<br>Setting: High school surveys<br>Study start date/end date: February/March 2015, 2016<br>Number of datapoints: 2

	Primary dataset: DISPLAY<br>Country: Scotland
Participants	Total N: 2125<br>Age: 11-18 years<br>EC use at baseline: 183 (8.6%)<br>Gender/sex: NR<br>Ethnicity/race: NR
Exposures	Electronic cigarettes Use: ever use<br>Details on EC devices: Not reported<br>Electronic cigarettes availability: Not applicable
Outcomes	Methods: "Prospective cohort survey conducted in four high schools in Scotland, UK during February/March 2015 (n=3807) with follow-up 1 year later. All pupils (age 11–18) were surveyed. Response rates were high m in both years (87% in 2015) and 2680/3807 (70.4%) of the original cohort completed the follow-up survey. Analysis was restricted to baseline 'never smokers' (n=3001/3807), 2125 of whom were available to follow-up (70.8%)."<br>Adjustment for confounders: "Multivariate logistic regression was used to control for potential confounding factors—sex, age, ethnicity, family affluence, smoking within the family, smoking by friends and susceptibility to smoking."<br>Type of combustible tobacco use: cigarettes<br>Combustible tobacco use: Past-30 days use
Study funding	"This project was funded by the UK National Institute for Health Research (NIHR) PHR project 10/3000/07. The study sponsor had no influence on study design and the collection, analysis, and interpretation of data and the writing of the article and the decision to submit it for publication."
Author declarations	"None declared."
Notes	NR: Not reported<br>Total N: analytical sample

Blank MD, Romm KF, Childers MG, Douglas AE, Dino G, Bray BC. Longitudinal transitions in adolescent polytobacco use across waves 1-4 of the Population Assessment of Tobacco and Health study. Addiction 2023;118(4):727-738.

Study characteristics

Methods	Design: Longitudinal cohort (Individual level study)<br>Recruitment: via Population Assessment of Tobacco and Health (PATH) study [waves 1–4 (2013–18)]<br>Setting: survey-based<br>Study start date/end date: W1 (2013–14)/ W4 (2016–18)<br>Number of datapoints: 4<br>Primary dataset: PATH<br>Country: USA
Participants	Total N: 975<br>Age: 13.29 (0.86) years<br>EC use at baseline: n=47 (4.8%)<br>Gender/sex: Male 440 (45.1%); Female 395 (40.5%)<br>Ethnicity/race: White 450 (46.2%); Black 75 (7.70%); Hispanic 211 (21.60%); Other 82 (8.40%)<br>Measures of socioeconomic status:<br>Adolescents' parents reported on their highest level of education (continuous; less than high school to 4-year college graduate). Parent education 2.57 (1.06)
Exposures	Electronic cigarettes Use: ever use, past 30 days use<br>Details on EC devices: NR<br>Electronic cigarettes availability: Not applicable
Outcomes	Methods: "Longitudinal analysis using data derived from waves 1–4 (2013–18) of the Population Assessment of Tobacco and Health (PATH) study. Transitions in tobacco use patterns were examined via latent transition analysis, and then, socio-demographic characteristics were used to predict transitions via logistic regression."<br>Adjustment for confounders: "For each covariate, this provided 'population-average' effect estimates. Gender and race/ethnicity covariates were treated as time-invariant based on W4 (no missing data); age and parent education were treated as time-variant."<br>Type of combustible tobacco use: cigarettes, large/traditional cigars, cigarillos, small/filtered cigars<br>Combustible tobacco use: ever use, past-30 days use<br>Latent classes of e-cigarette and cigarette use.
Study funding	"Supported by the National Institute on Drug Abuse of the National Institutes of Health (NIH) and the Center for Tobacco Products of the U.S. Food and Drug Administration (FDA) (R21DA051628). The content is solely the responsibility of the authors and does not necessarily represent the views of the NIH or FDA. Support also provided by the National Institute of General Medical Sciences (NIGMS) predoctoral training grant (T32 GM132494) to A.E.D."
Author declarations	None.

Bold KW, Kong G, Camenga DR, Simon P, Cavallo DA, Morean ME, et al . Trajectories of E-Cigarette and Conventional Cigarette Use Among Youth. Pediatrics 2018;141(1): e20171832.

Study characteristics

Methods	Design: Longitudinal cohort (Individual level study)<br>Recruitment: “Individual paper-and-pencil surveys were distributed during homeroom periods at each wave of the survey administration. Parents were contacted in advance of the study and could indicate if they did not want their child to participate. Students were informed that their participation was voluntary and that data were anonymous.”<br>Setting: High school surveys<br>Study start date/end date: Fall 2013/ Spring 2015<br>Number of datapoints: 3<br>Primary dataset: longitudinal survey from high school students in Connecticut<br>Country: USA
Participants	Total N: 808<br>Age: 13-17 years Mean (SD)=15.04 (0.90) years<br>EC use at baseline: 72 (8.9%)<br>Gender/sex: Male 380 (47.0%); Female 428 (53.0%)<br>Ethnicity/race: White 708 (87.6%); Asian 46 (5.7%); Hispanic and/or Latino 41 (5.1%); Black or African American 21 (2.6%); American Indian or Alaskan Native 8 (1.0%); Native Hawaiian or Pacific Islander 6 (0.7%); Middle Eastern 7 (0.9%); Other 3 (0.4%)<br>SES: Family Affluence Scale [mean (SD)] 5.92 (1.38)
Exposures	Electronic cigarettes Use: ever use, past-30 days use<br>Details on EC devices: Not reported<br>Electronic cigarettes availability: Not applicable
Outcomes	Methods: “Students completed surveys across 3 waves in 3 public schools in Connecticut. Using autoregressive cross-lagged models, we examined bidirectional relationships between past-month cigarette and e-cigarette use over time.”<br>Adjustment for confounders: “Models were adjusted for covariates related to tobacco use (i.e., sex, race/ethnicity, socioeconomic status, and use of other tobacco products)”<br>Type of combustible tobacco use: cigarettes<br>Combustible tobacco use: Past-30 days use<br>Odds ratio of Past-30 days cigarette use by e-cigarette use in previous waves.
Study funding	“Supported in part by grants from the National Institute on Drug Abuse and the Food and Drug Administration Center for Tobacco Products (P50DA036151, P50DA009241, T32DA019426, and L40DA042454). The content is solely the responsibility of the authors and does not necessarily represent the official views of the National Institutes of Health or the Food and Drug Administration. Funded by the National Institutes of Health (NIH).”
Author declarations	“The authors have indicated they have no potential conflicts of interest to disclose."
Notes	Total N: analytical sample

Boyd CJ, Veliz PT. Latent Classes of Youths' Nicotine Use and Association with Nicotine Dependence. Journal of Addiction Medicine 2020;14(6):e400-e401.

Study characteristics

Methods	Design: Longitudinal cohort (Individual level study)<br>Recruitment: via Population Assessment of Tobacco and Health (PATH) Study<br>Setting: High school surveys<br>Study start date/end date: Wave 1 (September 2013–December 2014); Wave 3 (October 2015–October 2016)<br>Number of datapoints: 3<br>Primary dataset: PATH<br>Country: USA
Participants	Total N: 1101<br>Age: 12-17 years<br>EC use at baseline: n= 121<br>Gender/sex: Male 52.4 (0.018); Female 47.6 (0.018)<br>Ethnicity/race: White 74 % (0.0018); Black 13 % (0.015); Other: 13% (0.011)<br>Household income: $24,999 or lower 22.6% (0.017); $25,000 to $49,999 25.9% (0.015); $50,000 to $99,000 26.4% (0.014); $100,000 or higher 25.1% (0.022)
Exposures	Electronic cigarettes Use: past-30 days use<br>Details on EC devices: Not reported<br>Electronic cigarettes availability: Not applicable
Outcomes	Methods: “Adolescents who indicated past 30-day nicotine/tobacco use at least once were included (n = 1101). We used latent class analysis (LCA) to identify nicotine/tobacco trajectories across three waves of PATH data and their association with six symptoms consistent with nicotine dependence from the Wisconsin Inventory of Smoking Dependence Motives (WISDM-68).”<br>Type of combustible tobacco use: cigarettes; cigarillos; cigars<br>Combustible tobacco use: Past-30 days use<br>Estimated latent class analysis probabilities.
Study funding	“This study was supported by grants from the National Institute on Drug Abuse [R01 DA044157] and National Cancer Institute [R01CA203809].”
Author declarations	“The authors declare that they have no known competing financial interests or personal relationships that could have appeared to influence the work reported in this paper.”

Brouwer AF, Jeon J, Jimenez-Mendoza E, Land SR, Holford TR, Friedman AS, et al . Changing patterns of cigarette and ENDS transitions in the USA: a multistate transition analysis of youth and adults in the PATH Study in 2015-2017 vs 2017-2019. Tobacco Control 2023 Mar 28 [Epub ahead of print]tc-2022-057905. [DOI: 10.1136/tc-2022-057905]

Study characteristics

Methods	Design: Longitudinal cohort (individual level study)<br>Recruitment: vai PATH<br>Setting: Nationally representative sample<br>Study start date/end date: start wave 1, October 2014, end wave 5 2019<br>Number of datapoints: 2<br>Primary dataset: PATH<br>Country: USA
Participants	Total N: 2015-2017 n= 12 067, 2017-2019 n=12 538<br>Age: 12-17 years, 12-14 years 57.5%, 15-17 years 42.5%<br>EC use at baseline: Youth Waves 2-4<br>Tobacco & ENDS use state<br>Never established use 96.6% n=11692<br>Non-current use 1.1% n=127<br>Cigarette-only use 1.0% n=116<br>Non-daily 0.5% n=59<br>Daily 0.5% n=57<br>ENDS-only use 1.0% n=99<br>Non-daily 0.8% n=77<br>Daily 0.2% n=22<br>Dual cigarette and ENDS use 0.3% n=33<br>Non-daily cigarette, non-daily ENDS 0.1% n=16<br>Non-daily cigarette, daily ENDS 0.1% n=6<br>Daily cigarette, non-daily ENDS 0.1% n=8<br>Daily cigarette, daily ENDS 0.0% n=3<br>Gender/sex: Female 48.6% n=5861, Male 51.3% n=6206<br>Ethnicity/race: Race/ethnicity wave 2-4<br>Non-Hispanic White 51.9% n=5509<br>Non-Hispanic Black 22.9% n=3504<br>Hispanic 12.7% n=1586<br>Non-Hispanic Other/Unknown 12.5% n=1468<br>Measures of socioeconomic status: Not reported
Exposures	Electronic cigarettes Use: past 30 days<br>Details on EC devices: Not reported<br>Electronic cigarettes availability: Not applicable

Outcomes	Methods: "A multistate transition model was applied to 24 242 adults and 12 067 youth in waves 2–4 (2015–2017) and 28 061 adults and 12 538 youth in waves 4 and 5 (2017–2019) of the Population Assessment of Tobacco and Health Study."<br>Adjustment for confounders: "Transition rates for initiation, cessation and product transitions were estimated in multivariable models, accounting for gender, age group, race/ethnicity and daily versus non-daily product use."<br>Type of combustible tobacco use: cigarettes<br>Combustible tobacco use: past 30 days<br>Underlying transition hazard rates between product use and covariate hazard rates.
Study funding	"This project was funded through National Cancer Institute and Food and Drug Administration (grant U54CA229974). The opinions expressed in this article are the authors' own and do not reflect the views of the National Institutes of Health, the Department of Health and Human Services or the US government."
Author declarations	"No, there are no competing interests."

Cantrell J, Huang J, Greenberg MS, Xiao H, Hair EC, Vallone D. Impact of e-cigarette and cigarette prices on youth and young adult e-cigarette and cigarette behaviour: evidence from a national longitudinal cohort. Tobacco Control 2020;29(4):374-380.

Study characteristics

Methods	Design: Longitudinal cohort (population level study)<br>Recruitment: via Truth Longitudinal Cohort (TLC)<br>Setting: address-based sampling with online data collection surveys<br>Study start date/end date: April to July 2014; July to October 2016.<br>Number of datapoints: 5<br>Primary dataset: TLC<br>Country: USA
Participants	Total N: 11578<br>Age: 15-21 years.<br>EC use at baseline: Individuals (n=11578) Past 30-day e-cigarette use: 9.73%; Observations (n=44771) Past 30-day e-cigarette use: 10.69%<br>Gender/sex: Individuals (n=11578) Female: 49.36%; Male: 50.64% Observations (n=44 771) Female: 50.04%; Male: 49.96%<br>Ethnicity/race: Individuals (n=11578) White, non- Hispanic 55.74%; Black, non- Hispanic 14.26%; Other, non- Hispanic 7.31%; Hispanic 19.97%; 2+ races 2.72% Observations (n=44 771) White, non- Hispanic 54.90%; Black, non- Hispanic 14.11%; Other, non- Hispanic 7.88%; Hispanic 20.24%; 2+ races 2.80%
Exposures	Electronic cigarettes Use: Past 30-days use<br>Details on EC devices: Not reported<br>Electronic cigarettes availability: Prices of e-cigarettes

Outcomes	Methods: “The authors conducted separate conditional likelihood logistic regression models with past 30-day e-cigarette use and past 30-day cigarette use outcomes on the sample of individuals who participated in at least two survey waves (n=11 578) with linked Nielsen market-level price data for rechargeable e-cigarettes and cigarettes.”<br>Adjustment for confounders: “Models controlled for time-varying variables at the individual and state policy levels, and fixed effects at the individual, wave and market levels<br>Type of combustible tobacco use: cigarettes<br>Combustible tobacco use: Past-30 days use<br>Impact of e-cigarettes and cigarettes prices on subsequent e-cigarettes use and cigarettes use.
Study funding	“This study was funded by Truth Initiative.”
Author declarations	“None declared.”

Chaffee BW, Watkins SL, Glantz SA. Electronic Cigarette Use and Progression From Experimentation to Established Smoking. Pediatrics 2018;141(4):e20173594.

Study characteristics

Methods	Design: Longitudinal cohort study (Individual level study)<br>Recruitment: via PATH<br>Setting: High school surveys<br>Study start date/end date: wave 1 (2013–2014)/wave 2 (2014–2015).<br>Number of datapoints: 2<br>Primary dataset: PATH<br>Country: USA
Participants	Total N:1295<br>Age: 18-24 years<br>EC use at baseline: ever use n=582<br>Gender/sex: Female 48.3%<br>Ethnicity/race: NR<br>Measures of socioeconomic status: NR
Exposures	Electronic cigarettes Use: Past-30 days<br>Details on EC devices: Not reported<br>Electronic cigarettes availability: Not applicable
Outcomes	Methods: “Among participants (age 12–17 years) in the nationally representative Population Assessment of Tobacco and Health survey who had smoked a cigarette (≥1 puff) but not yet smoked 100 cigarettes (*N* = 1295), we examined 3 outcomes at 1-year follow-up as a function of baseline e-cigarette use: (1) having smoked ≥100 cigarettes (established smoking), (2) smoking during the past 30 days, and (3) both having smoked ≥100 cigarettes and past 30-day

	smoking (current established smoking). Survey-weighted multivariable logistic regression models were fitted to obtain odds ratios (ORs) and 95% confidence intervals (CIs) adjusted for smoking risk factors."<br>Adjustment for confounders: "Model covariates include the following: sex, age, and race and/or ethnicity, parent education, urban residence, household tobacco use, alcohol ever use, tobacco advertisement receptivity, sensation-seeking score, cigarette warning label exposure, interview time of year, and ever use of any other tobacco product."<br>Type of combustible tobacco use: cigarettes<br>Combustible tobacco use: Past-30 days, ever use, number of lifetime uses<br>Odds ratios of e-cigarettes use and subsequent tobacco product use.
Study funding	"Supported in part by the US National Cancer Institute (T32CA113710), the US Food and Drug Administration Center for Tobacco Products (P50CA180890), and the National Institute on Drug Abuse (R01DA043950). The content is solely the responsibility of the authors and does not necessarily represent the official views of the National Institutes of Health or the US Food and Drug Administration. The funding agencies played no role in the conduct of the research or the decision to submit the article for publication. Funded by the National Institutes of Health (NIH)."
Author declarations	"The authors have indicated they have no potential conflicts of interest to disclose."
Notes	Total N represents final analytical sample.

Cheng HG, Largo EG, Gogova M. E-cigarette use and onset of first cigarette smoking among adolescents: An empirical test of the 'common liability' theory. F1000 Research 2019; 8:2099.

Study characteristics

Methods	Design: Longitudinal cohort (Individual level study)<br>Recruitment: via Population Assessment of Tobacco and Health Study (PATH)<br>Setting: address-based sampling with online data collection surveys<br>Study start date/end date: wave 1 (2013–2014) and wave 2 (2014–2015)<br>Number of datapoints: 2<br>Primary dataset: PATH<br>Country: USA
Participants	Total N: 9,045<br>Age: 12-17 years<br>EC use at baseline: 3.7%<br>Gender/sex: male: 51%<br>Race/ethnicity: non-Hispanic whites 54%
Exposures	Electronic cigarettes Use: Ever use<br>Details on EC devices: Not reported

	Electronic cigarettes availability: NA
Outcomes	Methods: "A structural equation modelling approach was used to estimate the relationship between e-cigarette use at wave 1 and the onset of cigarette smoking at wave 2 after controlling for a latent construct representing a "common liability to use tobacco products."<br>Adjustment for confounders: "Models included the following covariates: age, sex, race/ethnicity, availability of tobacco products in the household, novelty seeking, self-rated health, harm perception of cigarette smoking, and school performance. "<br>Type of combustible tobacco use: cigarettes<br>Combustible tobacco use: Ever use<br>Estimated relationship linking e-cigarette use to onset of ever cigarette smoking from structural equation models.
Study funding	NR
Author declarations	"All authors are full-time employees of Altria Client Services LLC."

Chien YN, Gao W, Sanna M, Chen PL, Chen YH, Glantz S, et al . Electronic Cigarette Use and Smoking Initiation in Taiwan: Evidence from the First Prospective Study in Asia. International Journal of Environmental Research and Public Health 2019;16(7):1-11.

Study characteristics

Methods	Design: Longitudinal cohort (Individual level study)<br>Recruitment: via Taiwan Adolescent to Adult Longitudinal Study (TAALS)<br>Setting: school-based surveys<br>Study start date/end date: Wave 1 2014; wave 2 2016.<br>Number of datapoints: 2<br>Primary dataset: TAALS<br>Country: Taiwan
Participants	Total N: 15795<br>Age: 13-16 years<br>EC use at baseline: n= 661<br>Gender/sex: male: n= 5661; female: n= 11839<br>Susceptible to smoking at Baseline: n= 879<br>Father's education: Below Junior High School n=2283; Senior or Vocational High School n= 4848; Above College n=4593<br>Mother's ethnicity: Native n=12114; Indigenous n=389; Foreigner n=1002<br>Parents' employment status: Full-time Job n=12114; Part-time Job n=242; Unemployed n=462<br>Family living arrangement: Parents or extended family n=10 227; single parents n=2128; grandparents n=278; other relatives n=321
Exposures	Electronic cigarettes Use: Ever use<br>Details on EC devices: Not reported

	Electronic cigarettes availability: NA
Outcomes	Methods: "Data from this TAALS were analysed via logistic regression to estimate the association between ever use of e-cigarettes at baseline and smoking initiation at follow-up, accounting for susceptibility to smoking, socio-demographic profile, depression status, and peer support. "<br>Adjustment for confounders: "Adjusted model including the following covariates: smoking susceptibility at baseline, socio-demographic profile, psychological status, and peer support. "<br>Type of combustible tobacco use: cigarettes<br>Combustible tobacco use: Ever use<br>Odds of ever use of e-cigarettes at baseline and ever use of cigarettes at follow up (defined as smoking initiation).
Study funding	"The work was supported by the Health Promotion Administration, Ministry of Health and Welfare, Taiwan (Grant Number: MOHW105-HPA-H-114-133708), from the Health and Welfare Surcharge on Tobacco Products—Grant Number: 03724606—Project Code: 1051218-107), and grants R01DA043950 from the US National Institute of Drug Abuse and P50CA180890 from the National Cancer Institute, the Food and Drug Administration (FDA) Center for Tobacco Products. The content is solely the responsibility of the authors and does not necessarily represent the official views of Health Promotion Administration, NIH or the Food and Drug Administration. The funding agencies had no role in study design, data collection, analysis, and interpretation, or writing of this study. The corresponding author had full access to all data in the study and had final responsibility for the decision to submit for publication."
Author declarations	"The authors declare no conflict of interest."

Coleman M, Donaldson CD, Crano WD, Pike JR, Stacy AW. Associations Between Family and Peer E-Cigarette Use With Adolescent Tobacco and Marijuana Usage: A Longitudinal Path Analytic Approach. Nicotine & Tobacco Research 2021;23(5):849-855.

Study characteristics

Methods	Design: Secondary analysis of a 3-year longitudinal cohort (individual level study)<br>Recruitment: a longitudinal study examining the influence of point-of-sale advertising on the use of nicotine and tobacco products by alternative high school students in southern California<br>Setting: Alternative high schools<br>Study start date/end date: started recruitment between October 2014 and May 2015, questionnaires complete by September 2015, end date 2 years after baseline<br>Number of datapoints: 3<br>Primary dataset: original dataset, longitudinal cohort<br>Country: US
Participants	Total N: 1025 at T1<br>Age: 14 and 18 at T1<br>EC use at baseline: Adolescent e-cigarette use (T1; past 30 days)<br>0 times n = 780<br>1–10 times n = 103

	11–20 times n = 30<br>21 or more times n = 59<br>Missing n = 53<br>Gender/sex: Male/female/missing n = 522/ n = 499/n = 4<br>Ethnicity/race: Non-Hispanic/Hispanic/missing n = 250/ n = 752/n = 23<br>Measures of socioeconomic status: Not reported
Exposures	Electronic cigarettes Use: Past 30 days<br>Details on EC devices: Not reported<br>Electronic cigarettes availability: Not applicable
Outcomes	Methods: " Relationships were examined in a secondary analysis of a 3-year longitudinal cohort subsample involving adolescents enrolled in alternative California high schools (N = 1025). Analyses examined responses over three yearly observations. Family, peer, and respondents' e-cigarette use, respondents' positive cigarette expectancies and willingness to use combustible tobacco cigarettes (CTCs) were assessed in the study's first year (T1). CTC use in the survey's second year (T2) and marijuana use in the third year (T3) were assessed via path analysis."<br>Adjustment for confounders: Path analytic model of proposed study relationships. Age, sex, and ethnicity (all T1) were entered into the model as covariates.<br>Type of combustible tobacco use: cigarettes<br>Combustible tobacco use: Past 30 days
Study funding	"Research reported in this publication was supported by the National Institute of Child Health and Human Development and the Food and DrugAdministration Center for Tobacco Products (R01HD077560). The content is solely the responsibility of the authors and does not necessarily represent the views of the National Institutes of Health or the Food and Drug Administration."
Author declarations	"None declared."

Conner M, Grogan S, Simms-Ellis R, Flett K, Sykes-Muskett B, Cowap L, et al . Evidence that an intervention weakens the relationship between adolescent electronic cigarette use and tobacco smoking: a 24-month prospective study. Tobacco Control 2020;29(4):425-431.

Study characteristics

Methods	Design: Secondary prospective analysis of 4-year cluster randomized controlled trial (RCT) (Individual level study)<br>Recruitment: "Head teachers consented to school participation with parents given option to withdraw children from the study. Adolescents consented by completing questionnaires matched across time points by code."<br>Setting: High school surveys<br>Study start date/end date: September–December 2014/September–December 2016<br>Number of datapoints: 2<br>Primary dataset: 4-year cluster randomized controlled trial (RCT)<br>Country: England

Participants	Total N: 3994<br>Age: 11-12 years<br>EC use at baseline: 758 (19.0%)<br>Gender/sex: Female n= 2090 (52.3%); Male n= 1904 (47.7%)<br>Ethnicity/race: White n= 688 (17.2%); Non- white n= 3306 (82.8%)<br>Measures of socioeconomic status: Family affluence [Mean (SD)] 2.72 (0.49)]; Free school meals<br>Low 22 (48.9%); High 23(51.1%)
Exposures	Electronic cigarettes Use: Ever use<br>Details on EC devices: Not reported<br>Electronic cigarettes availability: Not applicable
Outcomes	Methods: "Data were from 2836 adolescent never smokers (aged 13–14 years at baseline) as part of a cluster randomised controlled trial. Self-report measures of smoking, e-cigarette use and covariates were assessed and used to predict ever smoked cigarettes, any recent tobacco smoking and regularly smoked cigarettes at 24-month follow-up."<br>Adjustment for confounders: "Model covariates include the following: sex, age, and race and/or ethnicity, parent education, urban residence, household tobacco use, alcohol ever use, tobacco advertisement receptivity, sensation-seeking score, cigarette warning label exposure, interview time of year, and ever use of any other tobacco product."<br>Type of combustible tobacco use: cigarettes; cigars<br>Combustible tobacco use: Past-30 days, ever use, other<br>Odds ratios of e-cigarettes use and subsequent tobacco product use.
Study funding	"The research was supported by a grant from the UK Medical Research Council/National Preventive Research Initiative. CA is additionally supported by the National Institute for Health Research Manchester Biomedical Research Centre and the National Institute of Health Research Greater Manchester Patient Safety Translational Research Centre. All authors report receiving grants from the National Prevention Research Initiative during the study."
Author declarations	"None declared."
Notes	Total N represents final analytical sample.

Conner M, Grogan S, Simms-Ellis R, Cowap L, Armitage CJ, West R et al . Association between age at first reported e-cigarette use and subsequent regular e-cigarette, ever cigarette and regular cigarette use. Addiction 2021;116(7):1839-1847.

Study characteristics

Methods	Design: Secondary prospective analysis of 4-year cluster randomized controlled trial (RCT) (Individual level study)<br>Recruitment: "Head teachers consented to school participation with parents given option to withdraw children from the study. Adolescents consented by completing questionnaires matched across time points by code."<br>Setting: High school surveys<br>Study start date/end date: September–December 2014/September–December 2016<br>Number of datapoints: 3<br>Primary dataset: 4-year cluster randomized controlled trial (RCT)<br>Country: England
Participants	Total N: 3289<br>Age: 13-14 years<br>EC use at baseline: 649 (19.7%)<br>Gender/sex: Female 1743 (53%); Male 1546 (47%)<br>Ethnicity/race: White 2747 (83.5%); Non-white 542 (16.5%)<br>Measures of socioeconomic status: Family affluence [Mean (SD)] 2.72 (0.49)]; Free school meals<br>Low 22 (48.9%); High 23(51.1%)
Exposures	Electronic cigarettes Use: Ever use, regular use<br>Details on EC devices: Not reported<br>Electronic cigarettes availability: Not applicable
Outcomes	Methods: "Secondary analysis of a cluster RCT of 45 schools in England, 12- and 24-month follow-up of e-cigarette/cigarette ever/regular use. The sample was divided into groups of e-cigarette users: early users (at 13–14 years), late users (at 14–15 years) and never users (at 13–14 and 14–15 years). Dependent variables were self-reported regular e-cigarette and cigarette use and ever cigarette use at 15–16 years. Covariates were assessed."<br>"Data reported are from wave 3 (September–December 2014 in 13–14-year-olds), wave 4 (September–December 2015 in 14–15-year-olds) and wave 5 (September–December 2016 in 15–16-year-olds) of the trial when measures of e-cigarette use were added to data collection. Only respondents reporting having never smoked a cigarette at wave 3 were analysed."<br>Adjustment for confounders:" Multiple logistic regression that controlled for clustering by school and covariates gender, ethnicity, family affluence, school level measures (portion eligible for free school meals, condition - control or intervention, friends and family smoking, impulsiveness and views about smoking."<br>Type of combustible tobacco use: cigarettes<br>Combustible tobacco use: ever use, regular use<br>Odds ratios of regular and ever cigarette use following early and late<br>users of e-cigarettes compared with never users.
Study funding	"The research was supported by a grant MR/J000264/1<br>from the UK Medical Research Council/National Preventive Research Initiative. The UK Medical Research Council<br>had no role in the design and conduct of the study; collection, management, analysis and interpretation of the data; preparation, review or approval of the manuscript; and the decision to submit the manuscript for publication. C.J.A. is additionally supported by the NIHR Manchester Biomedical Research Centre and the NIHR Greater Manchester Patient Safety Translational Research Centre."

Author declarations	"All authors report receiving grants from the National Prevention Research Initiative during the study. The authors have no conflicts of interest."

Conner M, Grogan S, Simms-Ellis R, Flett K, Sykes-Muskett B, Cowap L, et al . Do electronic cigarettes increase cigarette smoking in UK adolescents? Evidence from a 12-month prospective study. Tobacco Control 2018;27(4):365–72.

Study characteristics

Methods	Design: secondary analysis of cluster RCT (individual level study)<br>Recruitment: via cluster RCT<br>Setting: 20 schools in England.<br>Study start date/end date: September–December 2014/ September–December 2015<br>Number of datapoints: 2<br>Primary dataset: part of a 4-year cluster RCT of a school-based smoking initiation intervention based on implementation intentions. adolescents (aged 13–14 years at baseline) in 20 schools in England.<br>Country: UK
Participants	Total N: full data were available on 2836 adolescents at baseline, baseline longitudinal sample of never used cigarettes was 1726.<br>Age: 13-14 Years, mean 13.2 years<br>EC use at baseline: everuse n= 969; 34.2%<br>Gender/sex: female 50.2%<br>Ethnicity/race: not reported<br>Measures of socioeconomic status:<br>Free school meals mean 14.24 (SD 6.63) - based on school level data
Exposures	Electronic cigarettes Use: ever use<br>Details on EC devices: not reported<br>Electronic cigarettes availability: not applicable
Outcomes	Methods: "Data were from 2836 adolescents (aged 13–14 years at baseline) in 20 schools in England. At baseline, breath carbon monoxide levels, self-reported e-cigarette and cigarette use, sex, age, friends and family smoking, beliefs about cigarette use and percentage receiving free school meals (measure of socioeconomic status) were assessed. At 12-month follow-up, self-reported cigarette use was assessed and validated by breath carbon monoxide levels. Multilevel logistic regressions were used to assess model fit (Akaike Information Criterion) and, for each predictor, the odds ratios (OR), 95% CIs and p value. The main analyses used the same analysis to predict follow-up initiation (1=smoked; 0=never smoked) or escalation (0=never, once or used to smoke cigarettes; 1=rarely, occasional or frequent cigarette smoking) of smoking based on ever use of e-cigarettes and covariates."<br>Adjustment for confounders: "Model 1 controlled for the clustering of adolescents within schools, and baseline e-cigarette ever use was a predictor; model 2 added baseline covariates; and model 3 tested interactions between each covariate and e-cigarettes ever use."

Type of combustible tobacco use: cigarette
Combustible tobacco use: ever use

Study funding "The research was supported by grant MR/J000264/1 from the UK Medical Research Council/National Preventive Research Initiative."

Author declarations "All authors report receiving grants from the National Prevention Research Initiative during the study. The authors have no conflicts of interest."

Creamer MR, Loukas A, Clendennen S, Mantey D, Pasch KE, Marti CN, et al . Longitudinal predictors of cigarette use among students from 24 Texas colleges. J Am Coll Health 2018;66(7):617-624.

Study characteristics

Methods
Design: Longitudinal cohort (individual level study)
Recruitment: via Project M-PACT
Setting: School-based surveys
Study start date/end date: November 2014-February 2015 / six months after wave 1
Number of datapoints: 2
Primary dataset: Project M-PACT
Country: USA

Participants
Total N: 4,296
Age: 18-29 years
EC use at baseline: Current e-cigarette use (n=696)
Gender/sex: Male (n=1532); Female (n=2763)
Ethnicity/race: White (n=1547); Hispanic (n=1304); African-American (n=330); Asian-American (n=795); Other (n=320)
Measures of socioeconomic status:
Parental Education
Mother
No high school (n=214)
Some high school (n=221)
Graduated high school (n=630)
Vocational/Technical School (n=130)
Some College (n=972)
Associate’s Degree (n=345)
Bachelor’s Degree (n=1170)
Graduate Degree (n=548)
Father
No high school (n=251)

	Some high school (n=307)<br>Graduated high school (n=621)<br>Vocational/Technical School (n=159)<br>Some College (n=563)<br>Associate’s Degree (n=215)<br>Bachelor’s Degree (n=1107)<br>Graduate Degree (n=876)
Exposures	Electronic cigarettes Use: past 30 days<br>Details on EC devices: Not reported<br>Electronic cigarettes availability: Not applicable
Outcomes	Methods: "Participants included young adults (n=4,296) from 24 Texas colleges, participating in a young adult cohort study, beginning in fall 2014. Mixed effects logistic regressions were conducted accounting for school-level variability."<br>Adjustment for confounders: included age, gender, ethnicity, other substance use, and parental and friend smoking in multivariable regression model of wave 1 socio-demographic and behavioral predictors of current cigarette use at wave 2.<br>Type of combustible tobacco use: cigarettes<br>Combustible tobacco use: past 30 days
Study funding	"Research reported in this publication was supported by grant number [1 P50 CA180906] from the National Cancer Institute and the FDA Center for Tobacco Products (CTP). The content is solely the responsibility of the authors and does not necessarily represent the official views of the NIH or the Food and Drug Administration."
Author declarations	"The authors have no conflicts of interest to report. The authors confirm that the research presented in this article met the ethical guidelines, including adherence to the legal requirements, of the United States and received approval from the Institutional Review Board of the University of Texas at Austin."

Creamer M, Case K, Loukas A, Cooper M Perry CL. Patterns of sustained e-cigarette use in a sample of young adults. Addictive Behaviors 2019;92:28-31.

Study characteristics

Methods	Design: Longitudinal cohort (Individual study)<br>Recruitment: via Marketing and Promotions Across Colleges in Texas (Project M-PACT) study.<br>Setting: Colleges<br>Study start date/end date: Autumn 2014/ Winter 2016<br>Number of datapoints: 5<br>Primary dataset: Project M-PACT<br>Country: USA
Participants	Total N: 3510 took part in the 5 surveys, analysis focussed on N=75 (2%) sustained users of e-cigarettes over the 5 waves.<br>Demographics reported for the N=75 sustained e-cigarette users:

	Age: mean 20.9 (SD 2.3) years<br>EC use at baseline: 100%<br>Gender/sex: Male 55%<br>Ethnicity/race: White 40%; Hispanic 28%
Exposures	Electronic cigarettes Use: Past-30 days use<br>Details on EC devices: "Device type was derived from the questions, "During the past 30 days, have you used a disposable e-cigarette or an e-cigarette with a disposable nicotine cartridge? Neither requires the addition of e-liquid/ e-juice;" and "During the past 30 days, have you used a vape pen, personal vaporizer, or any other device as intended (i.e., with nicotine e-liquid/e-juice)?"<br>At wave 1 amongst N=75 sustained users of e-cigarettes: Disposable 3 (4.2%), Rechargeable 54 (75%), Both 15 (20.8%).<br>Electronic cigarettes availability: Not applicable
Outcomes	Methods: "Data are drawn from five waves of the Project M-PACT cohort. Analyses are limited to those reporting past 30-day e-cigarette use at each wave (n = 75). Mixed effects regressions were conducted for the following dependent variables: device type, number of days used, combustible tobacco product use, and symptoms of nicotine dependence."<br>Adjustment for confounders: "Each model used survey wave as the time variable, and controlled for sociodemographic variables".<br>Type of combustible tobacco use: cigarettes; cigars<br>Combustible tobacco use: Past-30 days use<br>Odds ratios of combustible tobacco product use in a sample of sustained e-cigarette users over a two-year period time.
Study funding	"Research reported in this publication was supported by grant number [1 P50 CA180906] from the National Cancer Institute and the FDA Center for Tobacco Products (CTP). The content is solely the responsibility of the authors and does not necessarily represent the official views of the NIH or the Food and Drug Administration."
Author declarations	"All authors declare they have no conflicts of interest."
Notes	Total N represents final analytical sample.

Creamer MR, Dutra LM, Sharapova SR, Gentzke AS, Delucchi KL, Smith RA, et al. Effects of e-cigarette use on cigarette smoking among U.S. youth, 2004-2018. Preventive Medicine 2021; 142:106316.

Study characteristics

Methods	Design: Interrupted time series analysis (Population level study)<br>Recruitment: via National Youth Tobacco Surveys (NYTS)<br>Setting: Middle and high schools<br>Study start date/end date: 2004/2018<br>Number of datapoints: 11<br>Primary dataset: NYTS

	Country: USA
Participants	Total N: 17,711<br>Age: 11-18 years<br>EC use at baseline: ever use at 2011 3.3%<br>Gender/sex: measured but not reported<br>Ethnicity/race: measured but not reported<br>Measures of socioeconomic status: not reported
Exposures	Electronic cigarettes Use: ever use, past 30-days use<br>Details on EC devices: not reported<br>Electronic cigarettes availability: Not applicable
Outcomes	Methods: “An interrupted time series analysis was used for cross-sectional data from the 2004 to 2018 National Youth Tobacco Surveys (NYTS) to assess changes in cigarette and e-cigarette use over time. A multivariable logistic regression model used 2004–2009 NYTS data on psychosocial risk factors to predict individual-level cigarette smoking risk from 2011 to 2018. Model-predicted and actual cigarette smoking behaviour were compared”<br>Adjustment for confounders: "All models include NYTS-provided weights, stratification, design variables, male, race/ethnicity, age, living with a smoker, intention to smoke in the next year, likelihood to smoke a cigarette when offered from a friend, other tobacco use, and year (centered on 2009)."<br>Type of combustible tobacco use: cigarettes<br>Combustible tobacco use: ever use; past 30 days use
Study funding	"Dr. Delucchi’s work was supported by grant U54HL147127 from the National Heart, Lung, and Blood Institute, and from the Food and Drug Administration (FDA) Center for Tobacco Products. Dr. Glantz’s work was supported by grants R01DA043950 from the National Institute of Drug Abuse, U54HL147127 from the National Heart, Lung, and Blood Institute, and from the Food and Drug Administration (FDA) Center for Tobacco Products. Dr. Dutra’s work was funded by a contract between RTI International and the CDC’s Office of Smoking and Health. The content is solely the responsibility of the authors and does not necessarily represent the official views of NIH or FDA."
Author declarations	Not reported

Dave D, Feng B, Pesko MF. The effects of e-cigarette minimum legal sale age laws on youth substance use. Health Economics 2019; 3:419-436.

Study characteristics

Methods	Design: natural experiment (Population level study)<br>Recruitment: via Youth Risk Behavior Surveillance System (YRBSS)<br>Setting: schools<br>Study start date/end date: 2005/2015<br>Number of datapoints: 6<br>Primary dataset: YRBSS

	Country: USA
Participants	Total N: close to 800,000 person-year observations<br>Age: 9th-12th grades<br>EC use at baseline: Questions related to youth e-cigarette use are first included in the YRBSS in 2015, using data from this wave 45% of high school students have tried e-cigarettes in their lifetime and 24% are current (past 30 days) e-cigarette users.<br>Gender/sex: Female 51%<br>Ethnicity/race: White 56%, Black 14%, Hispanics 16%, Other races 14%<br>Measures of socioeconomic status: not reported
Exposures	Electronic cigarettes Use: not applicable<br>Details on EC devices: not applicable<br>Electronic cigarettes availability: Flavour restrictions, age of sales restrictions, setting restrictions
Outcomes	Methods: "The authors used difference-in-differences models and individual-level data from the national and state Youth Risk Behavior Surveillance System from 2005 to 2015 to examine the effects of e-cigarette minimum legal sale age (MLSA) laws on youth cigarette smoking, alcohol consumption, and marijuana use."<br>Comparators: "To isolate the ceteris paribus relationship between the laws and youth substance use, the authors controlled for an extensive set of policy controls"<br>Type of combustible tobacco use: cigarettes<br>Combustible tobacco use: current smoker, past-month smoker<br>Effects of e-cigarette minimum legal sale age (MLSA) laws on youth cigarette smoking.
Study funding	"Research reported in this publication was supported by the National Institute on Drug Abuse of the National Institutes of Health under Award Numbers R01DA039968 (PI: Dhaval Dave) and R01DA045016 (PI: Michael Pesko)."
Author declarations	Not reported

Do EK, Tulsiani S, Vallone DM, Hair EC. Transitions in Frequent to Daily Tobacco and Nicotine Use among Youth and Young Adults. Substance Use & Misuse 2022;57(11):1681-1687.

Study characteristics	
Methods	Design: Longitudinal cohort (Individual level study)<br>Recruitment: via Truth Longitudinal Cohort (TLC) (wave 7 and wave 9)<br>Setting: community<br>Study start date/end date: February–May 2018/ September-December 2019<br>Number of datapoints: 2

	Primary dataset: TLC<br>Country: USA
Participants	Total N: 5274<br>Age: range 15-27 years, 15–20 years n=1406 (26.7%), 21–27 years n=3868 (73.3%)<br>EC use at baseline: past 30-days n=1135 (21.5%), frequent to daily use n=191 (5.7%)<br>Gender/sex: Male n=2283 (43.3%); Female n=2991 (56.7%)<br>Ethnicity/race: non-hispanic, white n=3348 (63.5%); non-hispanic, black n=3348 (63.5%); Hispanic or Latino n=491 (9.3%) non-Hispanic, another race/ethnicity n=861 (16.3%)<br>Measures of socioeconomic status: Parent education, missing = 62 High School Diploma / GeD or less n=940 (18.1%); any education higher than High School Diploma / GeD n=4272 (81.9%) / Perceived financial state, missing = 22 Just meet basic expenses with nothing left over/ don't meet basic expenses n=1402 (26.7%); Live comfortably or meet needs with a little left over n=3850 (73.35%)
Exposures	Electronic cigarettes Use: past-30 days use, frequent to daily use<br>Details on EC devices: Not reported<br>Electronic cigarettes availability: Not applicable
Outcomes	Methods: "Data were obtained from the Truth Longitudinal Cohort (TLC), a nationally representative longitudinal cohort of youth and young adults. Latent class analysis was conducted to classify participants (n = 5274) into subgroups based upon frequency of use of cigarettes, e-cigarettes, and little cigars, cigarillos, and cigars (LCCs) in the past 30 days. Latent transition analysis was used to estimate the probability of use pattern transitions across 23 months (February 2018 to December 2019), adjusted for the effects of gender, race/ethnicity, financial situation, sensation seeking, parent education, and household smoking."<br>Type of combustible tobacco use: cigarette, cigarillos, cigars<br>Combustible tobacco use: past-30 days use, frequent to daily use<br>Transitions between e-cigarettes use and cigarette use in young people.
Study funding	"This research was internally funded by the Truth Initiative Schroeder Institute. Researchers did not receive grant funding from external agencies in the public or commercial sectors."
Author declarations	"The authors report no conflicts of interest."

Doran N, Brikmanis K, Petersen A, Delucchi K, Al-Delaimy WK, Luczak S, et al. Does e-cigarette use predict cigarette escalation? A longitudinal study of young adult non-daily smokers. Preventive Medicine 2017; 100:279-284.

Study characteristics

Methods	Design: Longitudinal cohort (Individual level study)

	Recruitment: “Participants were recruited via paid online advertisements, primarily on Facebook. Advertisements appeared to users with profiles indicating they met age and residency criteria. Accompanying text indicated that eligibility criteria included recent smoking. Eligible and interested individuals provided informed consent, then completed the baseline assessment. Additional assessments were completed 3, 6, 9, and 12 months later. Participants were compensated $25 at baseline and 12 months, and up to $40 each at 3, 6, and 9 months”<br>Setting: community<br>Study start date/end date: March 2015/ December 2016<br>Number of datapoints: 5<br>Primary dataset: Original dataset<br>Country: USA
Participants	Total N: 391<br>Age: 18-24 years<br>EC use at baseline: “During the six months pre-baseline, 19% of participants reported no e-cigarette use, 32% 1–3 uses, 27% 1–2 uses/month, 10% weekly use, 6% 2–4 uses/week, and 6% daily/almost daily use.”<br>Gender/sex: Male 52%<br>Ethnicity/race: not reported<br>Measures of socioeconomic status: not reported
Exposures	Electronic cigarettes Use: "How frequently have you used e-cigarettes in the past 6 months?” Response options included: 0 times; 1–3 times; 1–2 times per month; weekly; 2–4 times per week; and daily/almost daily<br>Details on EC devices: Not reported<br>Electronic cigarettes availability: Not applicable
Outcomes	Methods: "Participants were 18–24-year-old non-daily cigarette smokers recruited from across California. Cigarette and e-cigarette use were assessed online or via mobile phone every three months for one year between March 2015 and December 2016. Longitudinal negative binomial regression models adjusted for propensity for baseline e-cigarette use were used.”<br>Adjustment for confounders: "To reduce the possibility of confounding due to baseline differences we used covariate adjustment for propensity scores"<br>Type of combustible tobacco use: cigarettes<br>Combustible tobacco use: "How frequently have you used cigarettes in the past 6 months?” Response options included: 0 times; 1–3 times; 1–2 times per month; weekly; 2–4 times per week; and daily/almost daily”<br>Association between e-cigarettes use and cigarettes smoking over 12 months.
Study funding	“This work was supported by the National Institutes of Health (grant R01 DA037217 to N.D.), who provided financial support but had no other role in this project.”
Author declarations	“None.”

Duan Z, Wang Y, Huang J. Sex Difference in the Association between Electronic Cigarette Use and Subsequent Cigarette Smoking among U.S. Adolescents: Findings from the PATH Study Waves 1-4. International Journal of Environmental Research and Public Health 2021;18(4):1695.

Study characteristics

Methods	Design: Longitudinal cohort (Individual level study)<br>Recruitment: via Population Assessment of Tobacco and Health Study (PATH)<br>Setting: Nationally representative longitudinal sample<br>Study start date/end date: Wave 1 (September 2013 to December 2014), Wave 4 (December 2016 to January 2018).<br>Number of datapoints: 4<br>Primary dataset: PATH<br>Country: USA
Participants	Total N: In this study, Wave 1, Wave 2, and Wave 3 were each considered as the baseline wave for its corresponding 12-month follow-up wave. N=5001 wave 1, 6637 wave 2, 8177 wave 3.<br>Demographic data reported for wave 1 unless stated.<br>Age: 12-17 years<br>Age group: 12-14 years n=4388 (96.7%); 15-17 years n=168 (3.3%)<br>EC use at baseline: wave 1 n=19 (0.4%), wave 2 n=53 (0.9%), wave 3 n=112 (1.5%)<br>Gender/sex: Female: n=2450 (49.2%); Male: n=2551 (50.08%)<br>Ethnicity/race: Non-Hispanic White n=2334 (53.4%), Non-Hispanic Black n=722 (14.4%), Hispanic n=1498 (23.0%), Non-Hispanic Other n=447 (9.2%)<br>Measures of socioeconomic status: Parental education Less than high school n=1009 (17.4%); High school graduate n=907 (17.3%); Some college or associate degree n=1024 (19.9%); Bachelor’s degree or above n=2032 (45.4%)
Exposures	Electronic cigarettes Use: Past-30 days use<br>Details on EC devices: Not reported<br>Electronic cigarettes availability: Not applicable
Outcomes	Methods: “This study analyzed data from Wave 1 to 4 of the Population Assessment of Tobacco and Health (PATH) Study, a nationally representative longitudinal survey. Generalized estimation equations (GEE) were performed to estimate the associations between baseline e-cigarette use and subsequent cigarette smoking, controlling for sociodemographic characteristics, mental health conditions, and other tobacco use. Effect modifications by sex were examined."<br>Type of combustible tobacco use: cigarettes<br>Combustible tobacco use: Past-30 days use<br>Odds ratios of past 30 days cigarette smoking at 12-month follow-up waves among adolescents who were never cigarette smokers at baseline.
Study funding	“This research was funded by the National Institutes of Health (NIH), grant number R01CA194681.”

Author declarations	"The authors declare no conflict of interest."

Dunbar MS, Davis JP, Rodriguez A, Tucker JS, Seelam R, D'Amico EJ. Disentangling Within- and Between-Person Effects of Shared Risk Factors on E-cigarette and Cigarette Use Trajectories From Late Adolescence to Young Adulthood. Nicotine & Tobacco Research 2019;21(10):1414-1422.

Study characteristics

Methods	Design: Longitudinal cohort (Individual level study)<br>Recruitment: "Participants were recruited from 16 middle schools from three districts in the Los Angeles area as part of a substance use prevention program, CHOICE."<br>Setting: middle schools and high schools<br>Study start date/end date: 2015/ 2017<br>Number of datapoints: 3<br>Primary dataset: Original dataset<br>Country: USA
Participants	Total N: 2039<br>Age: 16-20 years, mean age 17.3 (SD 0.68)<br>EC use at baseline: Any lifetime e-cigarette use 25.5%; Any past-month use 7.9%<br>Gender/sex: Male 44%<br>Ethnicity/race: Hispanic 45%, White 20%, Asian 21%, Multi-ethnic 10%, Black 2%, Other 1%<br>Measures of socioeconomic status: 55% reported that their mother had a college degree or higher.
Exposures	Electronic cigarettes Use: Past-30 days use<br>Details on EC devices: Not reported<br>Electronic cigarettes availability: Not applicable
Outcomes	Methods: "Between 2015 and 2017, 2039 youths completed three Web-based surveys, allowing us to model EC and cigarette use from ages 16 to 20. Auto-regressive latent growth models with structured residuals (ALT-SR) examined both between-person and within-person associations between past-month frequency of EC use, cigarette use, and third variables (alcohol and marijuana use, mental health symptoms) over time."<br>Adjustment for confounders: "All models controlled for participant self-reported biological sex, race/ethnicity, mother's education, and intervention status at wave 1."<br>Type of combustible tobacco use: cigarettes<br>Combustible tobacco use: Past-30 days use<br>Association between e-cigarettes use and cigarettes smoking and shared risk factors.
Study funding	"This work was supported by two grants from the National Institute of Alcohol Abuse and Alcoholism at the National Institutes of Health (R01AA016577, R01AA020883: D'Amico)."

Author declarations	“The authors have no conflicts of interest to declare.”

Dutra LM, Glantz SA, Arrazola RA, King BA. Impact of E-Cigarette Minimum Legal Sale Age Laws on Current Cigarette Smoking. Journal of Adolescent Health 2018;62(5):532-538.

Study characteristics

Methods	Design: repeated cross-sectional survey (Population level study)<br>Recruitment: via National Youth Tobacco Surveys (NYTS)<br>Setting: middle and high schools<br>Study start date/end date: 2009/2014<br>Number of datapoints: 5<br>Primary dataset: NYTS<br>Country: USA
Participants	Total N: 83,026 (2009 N=17,855, 2011 N=15,008, 2012 N=19,488, 2013 N=14,074, 2014 N=16,952)<br>Age: 12-17 years, mean (SE) 2009 14.54 (0.10)<br>EC use at baseline: past 30-day use % (SE) 2009 0.00; 2011 1.09 (0.14); 2012 1.90 (0.19); 2013 2.78 (0.26); 2014 2.88 (0.19).<br>Gender/sex: % (SE) 2009 Female 49.72 (0.74), Male 50.28 (0.74)<br>Ethnicity/race: 2009 (% SE) White 57.54 (3.08), Hispanic 18.73 (1.87), Non-Hispanic black 14.78 (2.16), Non-Hispanic other 8.95 (0.73)<br>Measures of socioeconomic status: Median household income $ (SE) 2009 54.70 (0.98); Mean unemployment rate % (SE) 2009 9.01 (0.13)
Exposures	Electronic cigarettes Use: Past 30-days use<br>Details on EC devices: not applicable<br>Electronic cigarettes availability: minimum legal sale age (MLSA)
Outcomes	Methods: “In 2016 and 2017, we regressed (logistic) current (past 30-day) cigarette smoking (from 2009–2014 National Youth Tobacco Surveys [NYTS]) on lagged (laws enacted each year counted for the following year) and unlagged (laws enacted January–June counted for that year) state e-cigarette MLSA laws prohibiting sales to youth aged below 18 or 19 (depending on the state). Models were adjusted for year and individual- (e-cigarette and other tobacco use, sex, race/ ethnicity, and age) and state-level (smoke-free laws, cigarette taxes, medical marijuana legalization, income, and unemployment) covariates.”<br>Type of combustible tobacco use: cigarettes<br>Combustible tobacco use: past-30 days use<br>Effects of E-Cigarette Minimum Legal Sale Age Laws on Current Cigarette Smoking
Study funding	“Dr. Stanton Glantz’s research was supported by the National Institute on Drug Abuse (R01DA-043950) and the National Cancer Institute (R25CA-113710). Dr. Lauren Dutra’s research was supported by the Food and Drug Administration Center for Tobacco Products (P50CA180890) and the Centers for Disease Control and Prevention.”

Author declarations	"The authors have no financial disclosures."

Dutra LM, Glantz SA. E-cigarettes and National Adolescent Cigarette Use: 2004–2014. Pediatrics. 2017 Feb 1;139(2):e20162450

Study characteristics

Methods	Design: repeated cross-sectional survey (Population level study)<br>Recruitment: via National Youth Tobacco Surveys (NYTS)<br>Setting: schools (sixth- to 12th-grade)<br>Study start date/end date: 2004/2014<br>Number of datapoints: 7<br>Primary dataset: NYTS<br>Country: USA
Participants	Total N: 15 664 in 2013 to 24 690 in 2004<br>Age: 9-21 years, mean (SE)<br>EC use at baseline (2011): 0.3%<br>Gender/sex: Not reported.<br>Ethnicity/race: Not reported.<br>Measures of socioeconomic status: Median household income $ (SE) 2009 54.70 (0.98); Mean unemployment rate % (SE) 2009 9.01 (0.13)
Exposures	Electronic cigarettes Use: Ever use, Past 30-days use<br>Details on EC devices: not applicable<br>Electronic cigarettes availability: E-cigarette introduction to market
Outcomes	Methods: "Data were collected by using cross-sectional, nationally representative school-based samples of sixth- through 12th-graders from 2004–2014 National Youth Tobacco Surveys (samples ranged from 16 614 in 2013 to 25 324 in 2004). Analyses were conducted by using interrupted time series of ever (≥1 puff) and current (last 30 days) cigarette smoking. Logistic regression was used to identify psychosocial risk factors associated with cigarette smoking in the 2004–2009 samples; this model was then applied to estimate the probability of cigarette smoking among cigarette smokers and e-cigarette users in the 2011–2014 samples."<br>Type of combustible tobacco use: cigarettes<br>Combustible tobacco use: ever use; past 30d use (current use); other: number of days smoked in the past 30 days among current smokers (1–2 days, 3–5 days, 6–9 days, 10–19 days, 20–29 days, or all 30 days)<br>Changes in smoking after e-cigarettes were introduced to the US market
Study funding	"This research was supported by R01 CA-061021 (Dr Glantz) and R25CA-113710 and P50CA180890 (Dr Dutra) from the National Institutes of Health and the Food and Drug Administration's Center for Tobacco Products. The content is solely the responsibility of the authors and does not necessarily represent the official views of the National Institutes of Health or the US Food and Drug Administration. Funded by the National Institutes of Health (NIH)."

Author declarations	"The authors have indicated they have no potential conflicts of interest to disclose."

East K, Hitchman SC, Bakolis I, Williams S, Cheeseman H, Arnott D, et al. The Association Between Smoking and Electronic Cigarette Use in a Cohort of Young People. Journal of Adolescent Health 2018;62(5):539-547.

Study characteristics

Methods	Design: Longitudinal cohort (Individual level study)<br>Recruitment: "A non-probability quota sampling approach was adopted using Ipsos MORI's online panels to recruit respondents aged 11–18 years. Quotas were set in respect of age, gender, and Government Office Region (GOR) using data from Eurostat 2012 to ensure sample representativeness. Respondents were invited by email to participate in an online survey about smoking."<br>Setting: online surveys<br>Study start date/end date: April 6 and 20, 2016/ August 5 and October 7, 2016<br>Number of datapoints: 2<br>Primary dataset: 2016 Action on Smoking and Health Great Britain Youth longitudinal survey<br>Country: UK
Participants	Total N: 1152<br>Age: 11-18 years<br>11–13 n=438 (38.02%)<br>14–15 n=338 (29.34%)<br>16–18 n=376 (32.64%)<br>EC use at baseline: Ever use n=132 (11.46%)<br>Gender/sex: Female n=620 (53.82%)<br>Ethnicity/race: not reported<br>Measures of socioeconomic status: not reported
Exposures	Electronic cigarettes Use: ever use<br>Details on EC devices: Not reported<br>Electronic cigarettes availability: Not applicable
Outcomes	Methods: "A longitudinal survey of 1,152 11- to 18-year-olds was conducted with baseline in April 2016 and follow-up between August and October 2016. Logistic regression models and causal mediation analyses assessed whether (1) ever e-cigarette use and escalation were associated with smoking initiation (ever smoking at follow-up) among baseline never smokers (n = 923), and (2) ever smoking and escalation were associated with e-cigarette initiation (ever e-cigarette use at follow-up) among baseline never e-cigarette users (n = 1,020)."<br>Adjustment for confounders: "Models were adjusted for age, gender, school performance, problem behaviour, monthly alcohol use, EC susceptibility, some friends smoke, some friends use EC, at least one parent smokes, at least one parent uses EC, sibling(s) smoke, sibling(s) use EC, public approve of smoking, public approve of ECs."

	Type of combustible tobacco use: cigarettes<br>Combustible tobacco use: ever use<br>Odds of smoking initiation at follow-up and e-cigarette use.
Study funding	“This work was funded by Cancer Research UK grant code A21559. Thanks are also given to the UK Public Health Research Consortium (grant number PHPEHF50/13) for funding the development of some of the covariates included in this study.”
Author declarations	“Katherine East, Sara Hitchman, and Ann McNeill are members of the UK Centre for Tobacco and Alcohol Studies. Ioannis Bakolis is supported by the National Institute for Health Research (NIHR) Biomedical Research Centre at South London and Maudsley NHS Foundation Trust and by the NIHR Collaboration for Leadership in Applied Health Research and Care South London at King’s College Hospital NHS Foundation Trust. Sarah Williams is an employee at Public Health England and was previously an employee at Action on Smoking and Health at the time this study was conducted. Hazel Cheeseman and Deborah Arnott are employees of Action on Smoking and Health, which receives funding from the British Heart Foundation, Cancer Research UK (CRUK), and the Department of Health. The views expressed are those of the author(s) and not necessarily those of Public Health England, CRUK, Action on Smoking and Health, the NHS, the NIHR or the Department of Health.”

Epstein M, Bailey JA, Kosterman R, Rhew IC, Furlong M, Oesterle S, et al . E-cigarette use is associated with subsequent cigarette use among young adult non-smokers, over and above a range of antecedent risk factors: a propensity score analysis. Addiction 2021;116(5):1224-1232.

Study characteristics

Methods	Design: Longitudinal cohort (Individual level study)<br>Recruitment: via CYDS<br>Setting: Communities That Care prevention system in 24 small towns in seven states (Colorado, Illinois, Kansas, Maine, Oregon, Utah and Washington)<br>Study start date/end date: unclear/ 2016<br>Number of datapoints: 2<br>Primary dataset: Community Youth Development Study (CYDS)<br>Country: USA
Participants	Total N: 1825 (those who had not reported combustible cigarette use by age 21)<br>Age: 21-23 years<br>EC use at baseline: n = 226 (12%) at age 21<br>Gender/sex: For full sample aged 11-23 in 2016 (N=4407) Female 50%; Male 50%<br>Ethnicity/race: For full sample aged 11-23 in 2016 (N=4407) 20% Hispanic, 64% white, 3% black and 12% other race or ethnicity
Exposures	Electronic cigarettes Use: ever use<br>Details on EC devices: Not reported<br>Electronic cigarettes availability: Not applicable
Outcomes	Methods: “The current study is a quasi-experimental test of the relationship between e-cigarette use and subsequent combustible cigarette use among young adult nonsmokers, accounting for a wide range of common risk factors. Logistic regression was used to predict combustible cigarette use on three or

	more occasions at age 23 years based on age 21 e-cigarette use. Inverse probability weighting (IPW) was used to account for confounding variables. 22 common predictors of both e-cigarette and combustible cigarette use (e.g., pro-cigarette attitudes, peer smoking, family monitoring) were used to create IPWs."<br>Type of combustible tobacco use: cigarettes<br>Combustible tobacco use: ever use<br>Odds ratio of combustible cigarettes use following e-cigarette use.
Study funding	"This work was supported by research grants from the National Cancer Institute under awards R37CA225690 and R01CA203809, and the National Institute on Drug Abuse of the National Institutes of Health under awards R01DA015183 and R01DA044522, with co-funding from the National Institute of Child Health and Human Development, the National Institute of Mental Health, the Center for Substance Abuse Prevention and the National Institute on Alcohol Abuse and Alcoholism. The content is solely the responsibility of the authors and does not necessarily represent the official views of the funding agencies. Dr McCabe is a paid consultant for award R37CA225690."
Author declarations	Not reported

Evans-Polce RJ, Patrick ME, McCabe SE, Miech RA. Prospective associations of e-cigarette use with cigarette, alcohol, marijuana, and nonmedical prescription drug use among US adolescents. Drug and Alcohol Dependence 2020; 216:108303.

Study characteristics

Methods	Design: Longitudinal cohort (Individual level study)<br>Recruitment: via MTF<br>Setting: schools<br>Study start date/end date: 2014/ 2016<br>Number of datapoints: 2<br>Primary dataset: Monitoring the Future (MTF)<br>Country: USA
Participants	Total N: 717<br>Age: not reported<br>EC use at baseline: past 30-day use 12.14 % (SE 1.04)<br>Gender/sex: male 44.68 % (SE 1.62)<br>Ethnicity/race: Black 14.07% (SE 1.35); White 57.36 % (SE 1.64); Hispanic 17.89 % (SE 1.33); Other Black 10.68 % (SE 0.85)<br>Measures of socioeconomic status: Parent with college degree 53.03 % (SE 1.64)
Exposures	Electronic cigarettes Use: past 30-days use<br>Details on EC devices: Not reported<br>Electronic cigarettes availability: Not applicable

Outcomes	Methods: “The analytic sample included 2014–2016 MTF cohorts that were selected and completed follow up one year later (modal age 19; n = 717). Using logistic regression, we examined cross-sectional and prospective associations of past 30-day e-cigarette use with past 30-day cigarette, alcohol, marijuana, and NMPD use. We examined prospective associations among the full sample and associations with incidence of each of these substances among those who reported no history of use in 12th grade.”<br>Adjustment for confounders: "Multivariable regression analyses controlled for sex, race/ethnicity, region, parent education, and college plans measured at age 18. Cross-sectional analyses also controlled for the other three substance use outcomes (e.g., analyses examining the association of e-cigarettes and cigarette use controlled for past 30-day alcohol use, past 30-day marijuana use, and past 30-day NMPD use) at age 18."<br>Type of combustible tobacco use: cigarette<br>Combustible tobacco use: Past-30 days use; past 12-month use
Study funding	“Nothing declared. This work was supported by the National Institute on Drug Abuse [R01 DA001411 and R01 DA016575]; the National Institute on Alcohol Abuse and Alcoholism [R01AA023504]; and the National Cancer Institute [R01CA203809].”
Author declarations	“No conflict declared.”

Fearon IM, Seltzer RGN, Houser TL, Tope A, Cahours X, Verron T, et al . Curiosity and intentions to use myblu e-cigarettes and an examination of the 'gateway' theory: Data from cross-sectional nationally representative surveys . Drug Test Anal 2023;15(10):1257-1269.

Study characteristics

Methods	Design: Repeated cross- sectional surveys (individual level study)<br>Recruitment: Young adults (aged 18–24 years) who were enrolled members of an online research panel maintained by Qualtrics.<br>Setting: Nationally representative sample<br>Study start date/end date: August 2019/October 2020<br>Number of datapoints: 3<br>Primary dataset: Two independent, cross-sectional, US nationally representative surveys<br>Country: USA
Participants	Total N: Population composed of 4970, 5188 and 4646 participants from Waves 1 to 3 of the prevalence survey and 2500, 2612 and 2316 participants from Waves 1 to 3 of the perceptions survey (Total 22,232)<br>Age: 18-24 years, mean 21.5<br>EC use at baseline: Unclear<br>Gender/sex: Male 10,077 (50.1%), Female 11,798 (48.3%)<br>Transgender 357 (1.6%)<br>Ethnicity/race:<br>White 13,675 (73.3%)<br>Black or African American 4693 (15.0%)<br>American Indian or Alaska Native 502 (1.3%)

	Asian 985 (3.7%)<br>Multiracial 2377 (6.7%)<br>Measures of socioeconomic status: Not reported
Exposures	Electronic cigarettes Use: ever use, past 30 days<br>Details on EC devices: myblu ENDS<br>Electronic cigarettes availability: Not applicable
Outcomes	Methods: Data were obtained from two independent, cross-sectional, US nationally representative surveys; one study assessed prevalence of use of myblu ENDS, and the other study assessed perceptions of myblu ENDS use. Logistic regressions were used to test the relationship between smoking status and ever use of myblu ENDS.<br>Adjustment for confounders: Logistic regressions were performed using sex, race and age as covariates.<br>Type of combustible tobacco use: cigarettes<br>Combustible tobacco use: ever use, past 30 days
Study funding	"This work was funded by Fontem US LLC, a subsidiary of Imperial Brands PLC and manufacturer of the myblu™ products assessed in this study. This work was contracted by Imperial Brands PLC as a service provider on behalf of Fontem US LLC. Study design and data collection were performed independently of the study sponsor."
Author declarations	"IMF is an independent consultant contracted to e-cigarette/tobacco product manufacturers, including Imperial Brands PLC, to CROs including LA Clinical Trials, LLC (LACT) and to smoking cessation medication manufacturers to provide scientific support for clinical and behavioural studies and general regulatory support. RGNS was an employee of LACT at the time of survey conduct and analyses. TLH is an independent consultant contracted to LACT to provide analytical and writing support for clinical and behavioural studies. AT, TV, XC, LM, TN and GO'C were employees of Imperial Brands PLC, a company of which Fontem US LLC is a subsidiary, at the time of the study. MN is the president of LACT, which was contracted by Imperial Brands PLC to perform behavioural survey and clinical studies. MN has also contracted to consult with, and conducted behavioural and clinical studies for, other ENDS and smoking cessation medication manufacturers."

Foxon F, Selya AS. Electronic cigarettes, nicotine use trends and use initiation ages among US adolescents from 1999 to 2018. Addiction 2020;115(12):2369-2378.

Study characteristics

Methods	Design: natural experiment (Population level study)<br>Recruitment: via National Youth Tobacco Surveys (NYTS)<br>Setting: schools<br>Study start date/end date: 1999/2018<br>Number of datapoints: 14<br>Primary dataset: NYTS<br>Country: USA
Participants	Total N: ~12500 to ~31000 observations per wave.

	Age: 12-17 years<br>EC use at baseline: not reported<br>Gender/sex: not reported<br>Ethnicity/race: not reported<br>Measures of socioeconomic status: not reported
Exposures	Electronic cigarettes Use: not applicable<br>Details on EC devices: not applicable<br>Electronic cigarettes availability: Introduction of e-cigarettes to market in 2009
Outcomes	Methods: "The aims of this study were to examine prevalence trends of exclusive EC use, exclusive<br>cigarette use and dual use to determine the corresponding ages of initiation and to investigate hypothetical trends in total nicotine use and cigarette use in the absence of ECs among US adolescents. Observational study using data from the National Youth Tobacco Survey (NYTS) to statistically model trends in the prevalences of each user group and their initiation ages. Projections from counterfactual models based on data from 1999 to 2009 (before EC introduction) were compared with actual trends based on data from 1999 to 2018. Rigorous error analyses were applied, including Theil proportions. "<br>Type of combustible tobacco use: cigarettes<br>Combustible tobacco use: prevalence of total cigarette use<br>Exclusive cigarette use prevalence after introduction of e-cigarettes.
Study funding	"This work was supported by the National Institute of General Medical Sciences under grant number 1P20GM121341. The content is solely the responsibility of the authors and does not necessarily represent the official views of the NIH or NIGMS."
Author declarations	"None"

Friedman AS. How does electronic cigarette access affect adolescent smoking? Journal of Health Economics 2015;44:300-8.

Study characteristics

Methods	Design: natural experiment (Population level study)<br>Recruitment: via National Survey on Drug Use and Health<br>Setting: household interviews/surveys<br>Study start date/end date: 2002-2003/2012-2013<br>Number of datapoints: 6<br>Primary dataset: National Survey on Drug Use and Health (NSDUH)<br>Country: USA
Participants	Total N: unclear<br>Age: 12-17 years<br>EC use at baseline: not reported

	Gender/sex: not reported<br>Ethnicity/race: year 2002-2003 Percent under age 18 24,9%; Black 11,3%; other non-white race 7.2%; Hispanic 8.6%<br>Measures of socioeconomic status: Year 2002-2003 State demographics Median household income 54,932 (8210); State unemployment rate 5.47%; Population size 5673,825 (6386,612)
Exposures	Electronic cigarettes Use: not applicable<br>Details on EC devices: not applicable<br>Electronic cigarettes availability: Age of sale restrictions
Outcomes	Methods: “This paper examines the causal impact of e-cigarette access on conventional cigarette use by adolescents. Regression analyses consider how state bans on e-cigarette sales to minors influence smoking rates among 12 to 17-year olds.”<br>Type of combustible tobacco use: cigarettes<br>Combustible tobacco use: state-specific two-year averages of 12 to 17-year olds recent smoking rates; past-30 days use<br>Effects of e-cigarette access on conventional cigarette use by adolescents.
Study funding	“Radcliffe Institute for Advanced Study, for fellowship funding that helped support this research.”
Author declarations	Not reported

Friedman AS. Essays in health economics: Understanding risky health behaviors. Dissertation Abstracts International Section A: Humanities and Social Sciences 2015;75:(10-A(E)).

Study characteristics

Methods	Design: Natural experiment (population level study)<br>Recruitment: via NYTS<br>Setting: School surveys<br>Study start date/end date: start 2004, end 2012<br>Number of datapoints: 5<br>Primary dataset: NYTS<br>Country: USA
Participants	Total N: 2004: 13,413, 2006: 13,431, 2009: 12,093, 2011: 9,477, 2012: 12,695<br>Age: 12-18 years, mean 16 years<br>EC use at baseline: First available in 2011 - Ever tried e-cig: 4.5%, Used e-cigs in past 30 days: 1.4%<br>Gender/sex: 2004: 51% female, 2006: 51% female, 2009: 49% female, 2011: 49% female, 2012: 49% female<br>Ethnicity/race:<br>2004 Hispanic 11%, White 72%, Black 16%, Asian 5%,<br>American Indian or Alaska Native 3%, Native Hawaiian or Pacific Islander 2%

	2006 Hispanic 13%, White 71%, Black 17%, Asian 4%, American Indian or Alaska Native 4%, Native Hawaiian or Pacific Islander 2%<br>2009 Hispanic 17%, White 66%, Black 19%, Asian 5%, American Indian or Alaska Native 4%, Native Hawaiian or Pacific Islander 2%<br>2011 Hispanic 19%, White 67%, Black 18%, Asian 5%<br>American Indian or Alaska Native 5%, Native Hawaiian or Pacific Islander 2%<br>2012, Hispanic 20%, White 66%, Black 19%<br>Asian 6%, American Indian or Alaska Native 5%, Native Hawaiian or Pacific Islander 2%<br>Measures of socioeconomic status: Not reported
Exposures	Electronic cigarettes Use: everuse (2011), past-30 days use (2011)<br>Details on EC devices: Not reported<br>Electronic cigarettes availability: Sales data
Outcomes	Methods: "to study the extent to which gateway effects, dual use, and harm reduction shape the relationship between youth smoking and electronic cigarette use. Change in e-cig sales (in $100m) is used in regression to test if change in sales data is associated with change in current smokers. Specification checks include quantile means of fixed effects for respondent sex, year of age, grade, race, and ethnicity, as well as how often the respondent sees actors using tobacco on TV or in movies and whether he or she would smoke a cigarette if a friend offered it, lives with someone who smokes, lives with someone who uses smokeless tobacco, and believes smoking makes people look cool/fit in."<br>Type of combustible tobacco use: Cigarettes<br>Combustible tobacco use: Current smoker (i.e. Ever smoker [smoked >100 in life] + smoked in the past 30 days)
Study funding	"would also like to recognize the Agency for Healthcare Research and Quality, Mathematica Policy Research, and the Radcliffe Institute, for fellowship funding that helped support my training and research. Additionally, the United States Bureau of Labor Statistics and the Centers for Disease Control and Prevention both collect and provide access to the survey data I use in these studies."
Author declarations	Not reported
Notes	Dissertation

Friedman AS, Xu S. Associations of Flavored e-Cigarette Uptake With Subsequent Smoking Initiation and Cessation. JAMA Network Open 2020;6:e203826.

Study characteristics

Methods	Design: Longitudinal cohort (Individual level study)<br>Recruitment: via Population Assessment of Tobacco and Health Study (PATH)

	Setting: Nationally representative sample<br>Study start date/end date: Wave 1 (September 2013 to December 2014), Wave 4 (December 2016 to January 2018).<br>Number of datapoints: 4<br>Primary dataset: PATH<br>Country: USA
Participants	Total N: participants aged 12-17 years n = 7311; participants aged 18-24 years n=4634<br>Demographics for those who did not smoke or vape at baseline.<br>Age: 12-24 years<br>EC use at baseline: youth 12-17 years 164 yes/7096 no, emerging adult 18-24 years 102 yes/4517 no<br>Gender/sex: Male youths 12-17 years 51.18% (95% CI 49.79-52.57), Male emerging adults 47.00%(95% CI 45.43-48.57)<br>Ethnicity/race: % (95%CI)<br>Youth 12-17 years<br>Race<br>White 66.88 (64.15-69.51)<br>Black 15.28 (13.27-17.53)<br>Other 13.68 (12.28-15.21)<br>Hispanic 22.40 (19.66-25.41)<br>Emerging adults 18-24 years<br>Race<br>White 65.44 (62.53-68.24)<br>Black 15.50 (13.46-17.79)<br>Other 15.36 (13.46-17.49)<br>Hispanic 21.82 (19.29-24.57)<br>Measures of socioeconomic status:<br>Youth 12-17 years<br>Parental education % (95% CI)<br><High school 17.18 (15.67-18.79)<br>High school graduate 17.35 (16.14-18.63)<br>Some college 30.79 (28.96-32.68)<br>≥College degree 34.17 (31.60-36.83)<br>Parental household income, $ % (95% CI)<br><10 000 7.23 (6.21-8.40)<br>10 000-24 999 13.86 (12.64-15.19)<br>25 000-49 999 20.02 (18.78-21.32)<br>50 000-99 999 23.41 (22.15-24.72)<br>≥100 000 23.61 (21.61-25.73)<br>Emerging adults 18-24 years

	Any college at baseline % (95% CI) 59.40 (57.26-61.51)<br>Household income, $ % (95% CI)<br><10 000 24.17 (22.47-25.94)<br>10 000-24 999 20.14 (18.46-21.93)<br>25 000-49 999 16.89 (15.56-18.30)<br>50 000-99 999 15.40 (14.16-16.73)<br>≥100 000 10.64 (9.25-12.22)
Exposures	Electronic cigarettes Use: Past-30 days use, ever use<br>Details on EC devices: Flavoured vs unflavoured<br>Electronic cigarettes availability: Not applicable
Outcomes	Methods: “Multivariable analyses of nationally representative, longitudinal survey data evaluated differences in smoking initiation and cessation subsequent to vaping uptake among those who used flavoured vs unflavoured e-cigarettes.”<br>Adjustment for confounders: “All analyses adjusted for the sociodemographic controls and, for initiation analyses, whether the respondent had ever tried conventional cigarettes at baseline.”<br>Type of combustible tobacco use: cigarettes<br>Combustible tobacco use: Past-30 days use
Study funding	“Ms Xu was supported by a T32 National Research Service Award from the Agency for Healthcare Research and Quality (HS017589).”
Author declarations	“None reported.”

Friedman AS, Pesko MF. Young adult responses to taxes on cigarettes and electronic nicotine delivery systems. Addiction 2022;117(12):3121-3128.

Study characteristics

Methods	Design: Prospective cohort (population level study)<br>Recruitment: Not reported<br>Setting: Nationally representative data<br>Study start date/end date: 2010/2019<br>Number of datapoints: 9<br>Primary dataset: nine waves of the Current Population Survey’s Tobacco Use Supplement (CPS-TUS), collected between 2010 and 2019 (response rate ≈58%)<br>Country: USA
Participants	Total N: 38 906<br>Age: 18-25<br>EC use at baseline: binary indicators for recent and daily ENDS use, based on survey questions asking whether respondents “now use” each product “every day, some days, or not at all.”

	Gender/sex: Female ENDS tax 50.3%, no ENDS tax 50.7%<br>Ethnicity/race:<br>Non-Hispanic White ENDS tax 50.9%, no ENDS tax 58.8%<br>Non-Hispanic Black ENDS tax 13.8%, no ENDS tax 14.5%<br>Non-Hispanic Asian ENDS tax 8.0%, no ENDS tax 5.4%<br>Non-Hispanic, other race ENDS tax 2.0%, no ENDS tax 2.2%<br>Hispanic ENDS tax 25.3%, no ENDS tax 19.1%<br>Measures of socioeconomic status:<br>Employed ENDS tax 60.4%, no ENDS tax 62.8%<br>Unemployed ENDS tax 11.0%, no ENDS tax 10.1%<br>Not in labor force ENDS tax 28.7%, no ENDS tax 27.0% Poverty rate ENDS tax 13.9%, no ENDS tax 14.5%<br>Unemployment rate ENDS tax 7.6%, no ENDS tax 6.8%
Exposures	Electronic cigarettes Use: Not applicable<br>Details on EC devices: Not applicable<br>Electronic cigarettes availability: Taxes
Outcomes	Methods: "This study measures the relationship between ENDS and cigarette tax rates and ENDS use and smoking in young adulthood, a key period for initiation of regular tobacco use. Observational study of data from the Current Population Survey's 2010–2019 Tobacco Use Supplements. Multivariable linear regressions estimated two-way fixed effects analyses to assess ENDS and cigarette tax rates' relationships to recent and daily smoking and vaping, adjusting for an array of potential sociodemographic and policy confounders along with state and year fixed effects."<br>Adjustment for confounders: "Covariates adjust for state and month-by-year fixed effects, individual sociodemographics—indicators for sex, year of age, race/ethnicity, any college education, and employment status—and state covariates: unemployment and poverty rates, smoke- and vape-free indoor air law indexes, percent of population covered by tobacco-21 laws, beer tax rates, and binary indicators for whether the respondent could legally be sold cigarettes, medical and recreational marijuana legalization, and significant Medicaid expansions."<br>Type of combustible tobacco use: cigarettes<br>Combustible tobacco use: ever use, binary indicators for recent and daily cigarette use, based on survey questions asking whether respondents "now use" each product "every day, some days, or not at all."
Study funding	"This research was supported by an Evidence for Action grant from the Robert Wood Johnson Foundation (74869) (A.S.F.), the National Institute on Drug Abuse of the National Institutes of Health (NIDA-NIH) (R01DA045016) (M.F.P.), and the University of Kentucky's Institute for the Study of Free Enterprise (M.F.P.). None of the funders had any role in the study's design or conduct; the data's collection, analysis, or interpretation, or the manuscript's preparation. Content is solely the responsibility of the authors and does not necessarily represent the official views of the Robert Wood Johnson Foundation, NIDA-NIH, or the Institute for the Study of Free Enterprise."
Author declarations	"Neither author has conflicts of interest to disclose."

Gao W, Sanna M, Chuluunbaatar E, Tsai MK, Levy DT, Wen CP. Are e-cigarettes reviving the popularity of conventional smoking among Taiwanese male adolescents? A time-trend population-based analysis for 2004-2017. Tob Control [Internet]. 2021 Mar 1;30(2):132

Study characteristics

Methods	Design: repeated cross- sectional surveys (interrupted time series)<br>Recruitment: via Taiwan Adult Smoking Behaviour Survey (TASS) and Taiwan Global Youth Tobacco Survey (TGYTS)<br>Setting: High schools<br>Study start date/end date: 2005/2017<br>Number of datapoints: ~ 10<br>Primary dataset: Taiwan Adult Smoking Behaviour Survey and Taiwan Global Youth Tobacco Survey (TGYTS)
Participants	Total N: Not reported<br>Age: 12-18 years<br>EC use at baseline: 2.5% in 2014<br>Gender/sex: All male<br>Ethnicity/race: Not reported<br>Measures of socioeconomic status: Not reported
Exposures	Electronic cigarettes Use: ever-use, past-30 days use<br>Details on EC devices: Not applicable<br>Electronic cigarettes availability: Popularity of e-cigarettes
Outcomes	Methods: Authors “examined conventional cigarette and e-cigarette prevalence among male high school students (aged 16–18 years) and adults from 2004 to 2017, using data from cross-sectional nationally representative surveys. Applying interrupted time series analysis, they assessed whether there was a change in trend in 2014, when e-cigarette use started to gain popularity from long-term trends in prior years (2004–2013)."<br>Type of combustible tobacco use: cigarettes<br>Combustible tobacco use: past-30 days use; current conventional smokers (smoked at least 100 cigarettes in lifetime)
Study funding	"The authors have not declared a specific grant for this research from any funding agency in the public, commercial or not- for- profit sectors."
Author declarations	"None declared.”

Glantz SA. e-Cigarettes Used by Adolescents to Try to Quit Smoking Are Associated With Less Quitting: A Cross-Sectional Analysis of the National Youth Tobacco Survey . J Adolesc Health 2023;72(3):359-364.

Study characteristics

Methods	Design: Repeated cross- sectional surveys / longitudinal cohort (individual level study)<br>Recruitment: via NYTS<br>Setting: a nationally representative survey of middle and high school students (age 11-19)<br>Study start date/end date: 2015/2021<br>Number of datapoints: 7<br>Primary dataset: NYTS<br>Country: USA
Participants	Total N: 6,435 youth who started using cigarettes at least one year before starting to use e-cigarettes<br>Age: 11-19, mean 15.9<br>EC use at baseline: All years (%, SE) =50.6% (1.0%)<br>Gender/sex: Male all years: 55.4% (1.1%)<br>Ethnicity/race: NH-White All years (%,SE) = 7.9% (0.7%),<br>NH-Black All years (%,SE) = 24.8% (1.3%)<br>Hispanic All years (%,SE) = 1.5% (0.2%)<br>NH-Asian All years (%,SE) = 1.5% (0.2%)<br>NH-AI/AN All years (%,SE) = 1.0% (0.4%)<br>NH-HOPI All years (%,SE) = 5.8% (0.4%)<br>Multiple All years (%,SE) = 7.9% (0.7%)<br>NH = Non-Hispanic; AI/AN = American Indian/Alaska Native; HOPI = Hawaiian or other Pacific Islander<br>Measures of socioeconomic status: Not reported
Exposures	Electronic cigarettes Use: ever use<br>Details on EC devices: not reported<br>Electronic cigarettes availability: not applicable
Outcomes	Methods: "This study uses data from the NYTS from 2015 through 2021, focusing on youth who started smoking cigarettes before they started using e-cigarettes. Associations between using e-cigarettes to quit and having stopped smoking were computed using logistic regression accounting for the complex survey design and adjusting for level of nicotine dependence, year, age, gender, and race/ethnicity."<br>Type of combustible tobacco use: cigarettes<br>Combustible tobacco use: ever use, past 30 days
Study funding	"This research did not receive any specific grant from funding agencies in the public, commercial, or not-for-profit sectors."
Author declarations	"Dr. Glantz serves as a consultant to the World Health Organization."

Goldenson NI, Leventhal AM, Stone MD, McConnell RS, Barrington-Trimis JL. Associations of Electronic Cigarette Nicotine Concentration With Subsequent Cigarette Smoking and Vaping Levels in Adolescents. JAMA Pediatrics 2017;171(12):1192-1199.

Study characteristics

Methods	Design: Longitudinal cohort (Individual level study)<br>Recruitment: "Participants included 10th grade students from 10 high schools in the Los Angeles, California, metropolitan area followed up as part of a longitudinal survey of substance use and mental health. All data were collected using paper questionnaires at the participants' high schools; participants who were not available on the day that the data were collected completed telephone or internet surveys. Individual participants were not monetarily compensated; however, each participating high school's general fund was remunerated for staff time. The study was approved by the institutional review board of University of Southern California. Parents provided written informed consent, and youths assented to participation."<br>Setting: high schools<br>Study start date/end date: spring 2015/ autumn 2015<br>Number of datapoints: 2<br>Primary dataset: Original cohort (cohort study involving students from 10 high schools in the Los Angeles, California, metropolitan area)<br>Country: USA
Participants	Total N: 181<br>Age: 16.1 (SD 0.4) years<br>EC use at baseline: 100%<br>Gender/sex: male 53%<br>Ethnicity/race: Hispanic 38 (21.5%); White 34 (19.2%); Asian 17 (9.6%); Other 88 (49.7%)<br>Measures of socioeconomic status: Parental college degree or higher 67 (43.5%)
Exposures	Electronic cigarettes Use: past 30-days use<br>Details on EC devices: Not reported<br>Electronic cigarettes availability: Not applicable
Outcomes	Methods: "In this prospective cohort study involving students from 10 high schools in the Los Angeles, California, metropolitan area, surveys were administered during 10th grade in the spring (baseline) and 11th grade in the fall (6-month follow-up) of 2015 to students who reported using e-cigarettes within the past 30 days and the nicotine concentration level they used at baseline. Primary analyses used generalized linear mixed models with a random intercept to account for the clustering of students within their respective high schools."<br>Adjustment for confounders: "Adjusted for age, sex, race/ethnicity, highest parental educational level, baseline lifetime other tobacco product use, baseline e-cigarette or combustible cigarette topography in the past 30 days, baseline e-cigarette or combustible cigarette use in the past 30 days, baseline peer vaping, baseline peer smoking, sensation seeking, depressive symptoms, and delinquent behavior."<br>Type of combustible tobacco use: cigarette<br>Combustible tobacco use: Past-30 days use<br>Odds of frequent smoking after increasing levels of vaping nicotine.
Study funding	Not reported

Author declarations	"None reported."

Gueorguieva R, Buta E, Simon P, Krishnan-Sarin S, O'Malley SS. Data Visualization Tools of Tobacco Product Use Patterns, Transitions and Sex Differences in the PATH Youth Data. Nicotine & Tobacco Research 2020;22(10):1901-1908.

Study characteristics

Methods	Design: Longitudinal cohort (individual level study)<br>Recruitment: via PATH<br>Setting: Nationally representative household survey<br>Study start date/end date: start 2013-2014, end 2014-2015<br>Number of datapoints: 2<br>Primary dataset: PATH<br>Country: USA
Participants	Total N: 11996<br>Age: 12-17 years<br>EC use at baseline: e-cigarettes only (N = 263, 2.3%), both cigarettes and e-cigarettes (N = 186, 1.6%)<br>Gender/sex: Not reported<br>Ethnicity/race: Not reported<br>Measures of socioeconomic status: Not reported
Exposures	Electronic cigarettes Use: ever use<br>Details on EC devices: Not reported<br>Electronic cigarettes availability: Not applicable
Outcomes	Methods: "Set intersection bar plots describe ever use of five tobacco products among 12–17 years old youth in wave 1 of PATH) study (*N* = 11 497). Heat maps visualize unweighted frequencies of transitions from ever use at wave 1 (2013–2014) to past 12-month use at wave 2 (2014–2015). Weighted calibrated heat maps assess differences in relative frequencies of transitions by pattern at wave 1 and identify differences in transitions by sex."<br>Adjustment for confounders: Gender used for calibration difference analysis but no adjustment for other covariates.<br>Type of combustible tobacco use: cigarette, cigar, hookah<br>Combustible tobacco use: ever use and use within last 12 months
Study funding	"Research reported in this publication was supported by grant number P50DA036151 and U54DA036151 from the National Institute on Drug Abuse (NIDA) and Food and Drug Administration (FDA) Center for Tobacco Products (CTP), and by CTSA grant number UL1TR000142 from the National Center for Advancing Translational Science (NCATS), a component of the National Institutes of Health (NIH). The content is solely the responsibility of the authors and does not necessarily represent the official views of the NIH or the FDA."

Author declarations	"SSO discloses the following activities in the past year unrelated to this article: honoraria and travel reimbursement for participation in the American Society of Clinical Psychopharmacology Alcohol Clinical Trials Initiative supported with funding by Alkermes, Ethypharm, Lundbeck, Otsuka, and Mitsubishi; honoraria from Emmes Corporation for participation on NIDA DSMB; donated study medications: Novartis, Astra Zeneca; consultant: Alkermes, Amygdala, and Opiant (and travel reimbursement).R.G. discloses the following activities unrelated to this article: consultant for Knopp Biosciences and Cohen Veterans Bioscience, royalties from book "Statistical Methods in Psychiatry and Related Fields" published by CRC Press and from patent submission by Yale University: Chekroud, AM., Gueorguieva, R., & Krystal, JH. "Treatment Selection for Major Depressive Disorder" (filing date June 3, 2016, USPTO docket number Y0087.70116US00)."

Hair EC, Romberg AR, Niaura R, Abrams DB, Bennett MA, Xiao H, et al . Longitudinal Tobacco Use Transitions Among Adolescents and Young Adults: 2014-2016. Nicotine Tob Res 2019;21(4):458-468.

Study characteristics

Methods	Design: Longitudinal Study (individual level study)<br>Recruitment: via TLC<br>Setting: Truth Longitudinal Cohort (TLC), a probability-based, nationally representative survey of youth and young adults.<br>Study start date/end date: April and August 2014/January to April 2017<br>Number of datapoints: 6<br>Primary dataset: TLC<br>Country: USA
Participants	Total N: Participants who completed two or more surveys across six waves of data collection were included in the analyses (n = 15,275)<br>Age: 15-21 years, mean 18.6<br>EC use at baseline: 3.1% ENDS-only users, 6.1% Dual users<br>Gender/sex: 48.7% female, 51.3% male<br>Ethnicity/race: 66.6% white, 33.1% non-white, 0.003% missing<br>Measures of socioeconomic status:<br>Parent education: 84.0% greater than high school,14.6% high-school graduate or less, and 1.4% missing.
Exposures	Electronic cigarettes Use: ever use, past 30 days, noncurrent use (reported having used an ENDS but not in the past 30 days)<br>Details on EC devices: Not reported<br>Electronic cigarettes availability: Not applicable
Outcomes	Methods: "A nationally representative longitudinal survey of 15- to 21-year olds (n = 15,275) was used to describe transitions between never use, noncurrent use, and past 30-day use of combustible tobacco, e-cigarettes (ENDS), and dual use of both kinds of products. A multistate model was fit to observations collected every 6 months across 2.5 years to estimate the probability of transitions between states (TPs), the average time in state (sojourn time), and the effect of age on transitions."<br>Adjustment for confounders: Adjusted for age, gender, ethnicity and parents' education.

	Type of combustible tobacco use: cigarette, cigar, hookah<br>Combustible tobacco use: ever use, past 30 days, noncurrent use (reported having used a combustible product but not in the past 30 days)
Study funding	"This study was funded by Truth Initiative."
Author declarations	"None declared."

Hair EC, Barton AA, Perks SN, Kreslake J, Xiao H, Pitzer L, et al . Association between e-cigarette use and future combustible cigarette use: evidence from a prospective cohort of youth and young adults, 2017-2019. Addictive Behaviors 2021; 112:106593.

Study characteristics

Methods	Design: Longitudinal cohort (individual level study)<br>Recruitment: Truth Longitudinal Cohort (TLC). Participants were recruited at ages 15–21 through address-based sampling and surveyed online every six months to one year.<br>Setting: National anti-tobacco truth® campaign<br>Study start date/end date: January – April 2017 to late 2019 (September to December)<br>Number of datapoints: 4<br>Primary dataset: TLC<br>Country: US
Participants	Total N: model 1 N = 3289; model 2 N=3289<br>Age: range 15 to 24<br>EC use at baseline: model 1<br>Never used e-cigarettes, including JUUL 4639 (83.3%)<br>Ever used e-cigarettes but never used JUUL 549 (10.3%)<br>Past 30-day e-cigarette user but never used JUUL 66 (1.2%)<br>Ever used JUUL<br>298 (5.2%)<br>model 2<br>Never used e-cigarettes, including JUUL 5862 (69.7%)<br>Ever used e-cigarettes but never used JUUL 1467 (19.5%)<br>Past 30-day e-cigarette user but never used JUUL 170 (2.5%)<br>Ever used JUUL 697 (8.3%)<br>Gender/sex:<br>model 1<br>Male: 2202 (39.4%); Female: 3384 (60.6%)<br>model 2

	Male: 3593 (43.6%)<br>4647 (56.4%)<br>Ethnicity/race:<br>Model 1<br>White, non-Hispanic 3720 (55.7%)<br>Black/African American, non-Hispanic 467 (12.8%)<br>Hispanic 709 (20.6%)<br>Other 681 (10.9%)<br>Model 2<br>White, non-Hispanic 5460 (54.8%)<br>Black/African American, non-Hispanic 754 (14.3%)<br>Hispanic 1067 (21.3%)<br>Other 948 (9.6%)<br>Measures of socioeconomic status:<br>Model 1<br>Perceived Financial Situation<br>Does not meet needs/just meets needs 1209 (22.3%)<br>Meets needs with little left over/lives comfortably 4348 (77.7%)<br>Parental Education<br>Less than high school/high school graduate 759 (17.5%)<br>Some college/associate's degree 1261 (23.6%)<br>College graduate or more 3532 (58.9%)<br>Model 2<br>Perceived Financial Situation<br>Does not meet needs/just meets needs 1870 (23.6%)<br>Meets needs with little left over/lives comfortably 6334 (76.4%)<br>Parental Education<br>Less than high school/high school graduate 1145 (18.0%)<br>Some college/associate's degree 1893 (24.8%)<br>College graduate or more 5143 (57.2%)
Exposures	Electronic cigarettes Use: ever use, past 30-day use<br>Details on EC devices: e-cigarettes and JUUL<br>Electronic cigarettes availability: Not applicable
Outcomes	Methods: the sample is drawn from the TLC.<br>The sample for Model 1 are participants in the 2018 survey participants who<br>were never CLCC users in 2017. The sample for the multinomial Model 2 are all 2018 survey participants.

	Model 1: Logistic regression of e-cigarette use (baseline to 2018) as a predictor of cigar,<br>little cigar or cigarillo (CLCC) initiation from 2018 to 2019 (N = 5482).<br>Model 2: Multinomial logistic regression of e-cigarette use (baseline to 2018) and current flavoured CLCC use from 2018 to late 2019 (N = 7582)<br>Adjustment for confounders: age, gender, race/ethnicity, SES, sensation seeking score, hookah and marijuana use.<br>Type of combustible tobacco use: cigar,<br>little cigar or cigarillo (CLCC)<br>Combustible tobacco use: Ever use for CLCC initiation, past 30 days for flavoured CLCC use.
Study funding	"This study was internally funded by Truth Initiative."
Author declarations	"The authors declare that they have no known competing financial interests or personal relationships that could have appeared to influence the work reported in this paper."

Hair EC, Kreslake JM, Mowery P, Pitzer L, Schillo B, Vallone DM. A longitudinal analysis of e-cigarette use and cigar, little cigar or cigarillo initiation among youth and youth adults: 2017-2019. Drug Alcohol Depend 2021; 226:108821.

Study characteristics

Methods	Design: Longitudinal cohort (individual level study)<br>Recruitment: via Truth Longitudinal<br>Cohort (TLC)<br>Setting: national anti-tobacco truth® campaign<br>Study start date/end date: January – April 2017/ September - December 2019<br>Number of datapoints: 4<br>Primary dataset: TLC<br>Country: USA
Participants	Total N: model 1 n = 5586, model 2 n=8240<br>Age: 15–17 years model 1 n=777 (17.9%), model 2 n=919 (14.8%)<br>18–24 years model 1 n=4809 (82.2%), model 2 n= 6800 (85.2%)<br>EC use at baseline: Never used e-cigarettes, including JUUL model 1 n=4639 (83.3%), model 2 n= 5862 (69.7%)<br>Ever used e-cigarettes but never used JUUL model 1 n=549 (10.3%), model 2 n= 1467 (19.5%)<br>Past 30-day e-cigarette user but never used JUUL model 1 n=66 (1.2%), model 2 n= 170 (2.5%)<br>Ever used JUUL model 1 n=298 (5.2%), model 2 n= 697 (8.3%)<br>Gender/sex: Male model 1 n=2202 (45.1%), model 2 n= 3593 (50.2%)<br>Female model 1 n=3384 (54.9%), model 2 n= 4.647 (49.8%)<br>Ethnicity/race: White, non-Hispanic model 1 n=3720 (55.7%), model 2 n= 5460 (54.8%)<br>Black/African American, non-Hispanic model 1 n=467 (12.8%), model 2 n= 754 (14.3%)

	Hispanic model 1 n=709 (20.6%), model 2 n= 1067 (21.3%)<br>Other model 1 n=681 (10.9%), model 2 n= 948 (9.6%)<br>Measures of socioeconomic status:<br>Perceived Financial Situation<br>Does not meet needs/just meets needs model 1 n=1209 (22.3%), model 2 n= 1870 (23.6%)<br>Meets needs with little left over/lives comfortably model 1 n=4348 (77.7%), model 2 n= 6334 (76.4%)<br>Parental Education<br>Less than high school/high school graduate model 1 n=759 (17.5%), model 2 n= 1145 (18.0%)<br>Some college/associate's degree model 1 n=1261 (23.6%), model 2 n= 1893 (24.8%)<br>College graduate or more model 1 n=3532 (58.9%), model 2 n= 5143 (57.2%)
Exposures	Electronic cigarettes Use: ever use, past 30 days<br>Details on EC devices: e-cigarettes and JUUL<br>Electronic cigarettes availability: Not applicable
Outcomes	Methods: Examined whether youth and young adult e-cigarette use is associated with initiation of cigars, little cigars, or cigarillos (CLCCs) and current use of flavoured CLCCs. Used the Truth Longitudinal Cohort, the sample for this study was CLCC-naïve defined as those who had never used CLCCs as of 2017 (N = 5586). Weighted and adjusted logistic regression models were conducted to<br>assess the relationship between ever use of e-cigarettes by 2018 and initiation of CLCCs from 2018 to late 2019 (Model 1) and a multinomial model of the following outcomes from 2018 to late 2019: current (past<br>30-day) flavored CLCC use; current non-flavored CLCC use (reference group for the outcome); or no current CLCC use (Model 2).<br>Adjustment for confounders: Models controlled for combustible cigarette or hookah use, sensation seeking, demographic variables including age, race/ethnicity, gender, financial situation and parental education.<br>Type of combustible tobacco use: cigarillo, cigar, little cigar<br>Combustible tobacco use: ever use<br>The outcomes were the odds of (1) initiating any CLCC use and (2) reporting current (past 30-day) use of flavored CLCCs from 2018 to late 2019. The main predictor was use of e-cigarettes by 2018.
Study funding	"This research did not receive any specific grant from funding agencies in the public, commercial, or not-for-profit sectors."
Author declarations	"No conflict declared."

Hallingberg B, Maynard OM, Bauld L, Brown R, Gray L, Lowthian E, et al . Have e-cigarettes renormalised or displaced youth smoking? Results of a segmented regression analysis of repeated cross sectional survey data in England, Scotland and Wales. Tobacco Control 2020;29(2):207-216.

Study characteristics	
Methods	Design: Interrupted time- series analysis of repeated cross- sectional time- series data (Population level study)

	Recruitment: via The annual Smoking Drinking and Drug Use Among Young People in England Survey (SDDU), the biennial Scottish Adolescent Lifestyle and Substance Use Survey (SALSUS), and for Wales, the Health Behaviour in School- aged Children (HBSC) survey (from 1998 to 2013) and the School Health Research Network (SHRN) survey (2015).<br>Setting: schools<br>Study start date/end date: 2010/2015<br>Number of datapoints: Data from at least one UK country were available for each of 18 time points<br>Primary dataset: SDDU, HBSC, SHRN<br>Country: UK (England, Wales and Scotland)
Participants	Total N: 248 324<br>Age: SALSUS: 13-15 years; SDDU + HBSC/SHRN: 11-16 years<br>EC use at baseline: NR<br>Gender/sex: NR<br>Ethnicity/race: NR<br>Measures of socioeconomic status: NR
Exposures	Electronic cigarettes Use: N/A<br>Details on EC devices: N/A<br>Electronic cigarettes availability: Unregulated growth of e-cigarette use (following the year 2010, until 2015)
Outcomes	Methods: Interrupted time-series analysis of repeated cross-sectional time-series data was conducted to examine to examine whether during a period of limited e-cigarette regulation and rapid growth in their use, smoking began to become renormalised among young people.<br>Type of combustible tobacco use: cigarettes<br>Combustible tobacco use: Other<br>Cigarette use prevalence after introduction of e-cigarettes.
Study funding	"This work presents independent research funded by the National Institute for Health Research (NIHR) in England under its Public Health Research Board (grant number 16/57/01). The views expressed in this article are those of the authors and do not necessarily reflect those of the National Health Service (NHS), the NIHR or the Department of Health for England. The work was also undertaken with the support of The Centre for the Development and Evaluation of Complex Interventions for Public Health Improvement (DECIPHer), a UKCRC Public Health Research Centre of Excellence. Joint funding (MR/KO232331/1) from the British Heart Foundation, Cancer Research UK, Economic and Social Research Council, Medical Research Council, the Welsh Government and the Wellcome Trust, under the auspices of the UK Clinical Research Collaboration, is gratefully acknowledged."
Author declarations	"Competing interests LB declares a secondment post with Cancer Research UK and all other authors report no support from any organisation for the submitted work; no financial relationships with any organisations that might have an interest in the submitted work in the previous three years; no other relationships or activities that could appear to have influenced the submitted work."

Hammond D, Reid JL, Cole AG, Leatherdale ST. Electronic cigarette use and smoking initiation among youth: a longitudinal cohort study. CMAJ 2017;189(43):E1328-E1336.

Study characteristics

Methods	Design: Cohort study (individual level study)<br>Recruitment: via COMPASS<br>Setting: secondary school students (grades 9–12)<br>Study start date/end date: start 2013/2014, end 2014/2015<br>Number of datapoints: 2<br>Primary dataset: COMPASS<br>Country: Canada
Participants	Total N:<br>44163 for baseline; 41262 at follow up; 19 310 students who provided data for both waves were included in the longitudinal analysis.<br>Age:<br>≤ 14 overall sample n=9743 (22.1%), longitudinal sample n=6255 (32.4%)<br>15 overall sample n=11 283 (25.6%), longitudinal sample n=6517 (33.7%)<br>16 overall sample n=11 175 (25.3%), longitudinal sample n=5238 (27.1%)<br>17 overall sample n=9122 (20.7%), longitudinal sample n=1198 (6.2%)<br>≥ 18 overall sample n=2840 (6.4%), longitudinal sample n=102 (0.5%)<br>EC use at baseline: past 30-day e-cigarette use 7.2%<br>Gender/sex:<br>Female overall sample n=21 901 (49.6%), longitudinal sample n=10 303 (53.4%), Male overall sample n=22 262 (50.4%), longitudinal sample n=9007 (46.6%)<br>Ethnicity/race:<br>White overall sample n=32 886 (74.8%), longitudinal sample n=14 940 (77.7%)<br>Black overall sample n=1689 (3.8%), longitudinal sample n=603 (3.1%)<br>Asian overall sample n=2241 (5.1%), longitudinal sample n=979 (5.1%)<br>Aboriginal overall sample n=1546 (3.5%), longitudinal sample n=478 (2.5%)<br>Latin American/Hispanic overall sample n=830 (1.9%), longitudinal sample n=305 (1.6%)<br>Other/mixed overall sample n=4759 (10.8%), longitudinal sample n=1929 (10.0%)<br>Measures of socioeconomic status:<br>Spending money, $<br>0: overall sample n=7046 (16.0%), longitudinal sample n=3605 (18.7%)<br>1–20: overall sample n=12 680 (28.7%), longitudinal sample n=6594 (34.1%)<br>21–100: overall sample n=11 749 (26.6%), longitudinal sample n=4650 (24.1%)<br>> 100: overall sample n=6994 (15.8%), longitudinal sample n=1850 (9.6%)<br>Don’t know/not stated: overall sample n=5694 (12.9%), longitudinal sample n=2611 (13.5%)

Exposures	Electronic cigarettes Use: past 30 days<br>Details on EC devices: not reported<br>Electronic cigarettes availability: not applicable
Outcomes	Methods: "analyzed data from students in grades 9–12 who participated in 2 waves of COMPASS, a cohort study of purposefully sampled secondary schools in Ontario and Alberta, Canada, at baseline (2013/14) and 1-year follow-up (2014/15). We assessed cigarette smoking and e-cigarette use at baseline and follow-up using self-completed surveys. We used generalized linear mixed-effects models to examine correlates of past 30-day e-cigarette use at baseline and smoking initiation between waves within the longitudinal sample."<br>Adjustment for confounders: included covariables for age, sex, race/ethnicity, spending money, smoking status (as fixed effects) and a random effect of school in the models.<br>Type of combustible tobacco use: cigarettes<br>Combustible tobacco use: ever use, past 30 days
Study funding	"The COMPASS study was supported by a bridge grant from the Canadian Institutes of Health Research (CIHR) Institute of Nutrition, Metabolism and Diabetes through the Obesity — Interventions to Prevent or Treat priority funding awards (OOP-110788, awarded to Scott Leatherdale) and an operating grant from the CIHR Institute of Population and Public Health (MOP-114875,awarded to Scott Leatherdale). Additional support for this paper was provided by an Ontario Ministry of Health and Long-Term Care Health Systems Research Fund grant (06697, awarded to David Hammond), a CIHR New Investigator Award (awarded to David Hammond), a CIHR Doctoral Research Award— Frederick Banting and Charles Best Canada Graduate Scholarship (awarded to Adam Cole) and CIHR Public Health Agency of Canada Chairs in Applied Public Health (awarded to David Hammond and Scott Leatherdale). The researchers are independent from all sources of funding; the study sponsors had no role in study design; the collection, analysis or interpretation of data; the writing of the article; or the decision to submit it for publication."
Author declarations	"None declared."

Han DH, Elam KK, Quinn PD, Huang C, Seo DC. Within-person associations of escalated electronic nicotine delivery systems use with cigarette, alcohol, marijuana and drug use behaviors among US young adults. Addiction 2023;118(3):509-519.

Study characteristics

Methods	Design: Longitudinal cohort (individual level study)<br>Recruitment: via PATH<br>Setting: nationally representative longitudinal data on substance use behaviors among young people in the USA.<br>Study start date/end date: wave 1 (2013)/wave 5 (2019)<br>Number of datapoints: 5<br>Primary dataset: PATH<br>Country: USA
Participants	Total N: 5042<br>Age: 18-24 years

	EC use at baseline: not clear, n= 255 (4.0%) were current established ENDS users at wave 1<br>Gender/sex: Male n=2361 (49.9%), Female n=2681 (50.1%)<br>Ethnicity/race:<br>White n=3440 (70.8%)<br>Non-white n=1602 (29.2%)<br>Hispanic n=1237 (20.6%)<br>Non-Hispanic n= 3805 (79.3%)<br>Measures of socioeconomic status:<br>Current degree program<br>Not enrolled n=3090 (58.3%)<br>Enrolled n=1939 (41.7%)<br>Federal poverty level<br>≥ 200% (at or above twice poverty level) n=1263 (32.5%)<br>100–199% (at or near poverty level) n=968 (21.6%)<br>< 100% (below poverty level) n=2255 (45.9%)
Exposures	Electronic cigarettes Use: current established use, past 30 days<br>Details on EC devices: Association between device type (tank/mod, replaceable cartridge, disposables) and multiple substance use behaviors among established ENDS-using young adults at Wave 5.<br>Electronic cigarettes availability: not applicable
Outcomes	Methods: "the study aimed to examine within-person changes in escalated ENDS use and their associations with individual and combined substance use over a 6-year period. This study used a longitudinal cohort design with US young adults. A generalized linear mixed-model approach was employed to fit a series of weighted logistic regression models. Data were drawn from waves 1-5 of the Population Assessment of Tobacco and Health (PATH) study in the United States. Of the 9110 young adults at baseline, aged 18-24 years, a total of 5042 individuals had matched data across all five waves of assessments."<br>Adjustment for confounders: "Time-fixed covariates included baseline socio-demographic characteristics such as sex (female versus male), ethnicity (Hispanic versus non-Hispanic), race (white versus non-white), enrollment in a degree program (currently enrolled versus not enrolled) and federal poverty level (≥ 200, 100–199 or < 100% of poverty guideline). "<br>Type of combustible tobacco use: cigarettes<br>Combustible tobacco use: current established use
Study funding	"This research did not receive any specific grant from funding agencies in the public, commercial or not-for-profit sectors."
Author declarations	"None of the authors have any conflicts of interest to disclose, including relevant financial interests, activities, relationships and affiliations."

Harlow AF, Stokes AC, Brooks DR, Benjamin EJ, Barrington-Trimis JL, Ross CS. e-Cigarette Use and Combustible Cigarette Smoking Initiation Among Youth: Accounting for Time-Varying Exposure and Time-Dependent Confounding. Epidemiology 2022;33(4):523-532.

Study characteristics

Methods	Design: Longitudinal cohort study (Individual level study)<br>Recruitment: via PATH<br>Setting: High school surveys<br>Study start date/end date: wave 1 (2013–2014)/wave 5 (2018–2019).<br>Number of datapoints: 5<br>Primary dataset: PATH<br>Country: USA
Participants	Total N: 9584<br>Age: 12-17 years<br>EC use at baseline: 0% at wave 2. The authors excluded youth who used e-cigarettes at wave 1 to avoid prevalent exposure bias.<br>Gender/sex: Female n= 4784<br>Ethnicity/race: Non-Hispanic Black n=1381; Non-Hispanic White n=4539; Non-Hispanic Asian or other race n= 871; Hispanic n= 799<br>Measures of socioeconomic status: Parental education <High school or equivalent n= 3652; Some college or associates degree n=1125; ≥Bachelors degree n= 2962.
Exposures	Electronic cigarettes Use: Past-30 days, ever use<br>Details on EC devices: Not reported<br>Electronic cigarettes availability: Not applicable
Outcomes	Methods: “Using five waves of the Population Assessment of Tobacco and Health (2013–2019), we estimated marginal structural models with inverse probability of treatment and censoring weights to examine the association between time-varying e-cigarette initiation and subsequent cigarette smoking initiation among e-cigarette– and cigarette-naïve youth (12–17 years) at baseline. Time-dependent confounders used as predictors in inverse probability weights included tobacco-related attitudes or beliefs, mental health symptoms, substance use, and tobacco-marketing exposure."<br>Adjustment for confounders: “Model includes time-varying exposure, time-specific intercept, and wave 1 values of age, sex, race/ethnicity, parental education, living with tobacco user, grades, externalizing mental health, alcohol, marijuana, and other tobacco use (past 12 months), cigarette susceptibility, perceived harm of vaping, having a favorite tobacco ad.”<br>Type of combustible tobacco use: cigarettes<br>Combustible tobacco use: Past-30 days, ever use<br>Risk ratios for the association between e-cigarette use and cigarette smoking initiation.
Study funding	“Supported by grant U54HL120163 from the National Heart, Lung, and Blood Institute and FDA Center for Tobacco Products.”

Author declarations	“The authors report no conflicts of interest.”
Notes	Total N represents final analytical sample.

Harrell MB, Mantey DS, Chen B, Kelder SH, Barrington-Trimis J. Impact of the e-cigarette era on cigarette smoking among youth in the United States: A population-level study. Prev Med 2022; 164:107265.

Study characteristics

Methods	Design: interrupted time-series analyses (population level study)<br>Recruitment: via NYTS<br>Setting: The National Youth Tobacco Survey (NYTS) is an annual, cross-sectional survey of tobacco use behaviors among school students in the US.<br>Study start date/end date: pooled thirteen, non-consecutive years of NYTS data (2002–2019)<br>Number of datapoints: 13<br>Primary dataset: NYTS<br>Country: USA
Participants	Total N: A total of n = 283,201 middle and high school students completed the NTYS from 2002–2019. Sample for analysis includes only participants who reported their grade level and past 30-day cigarette smoking status. N = 8650 (2.7%) participants were missing, reflecting a final sample size of n = 274,551 (weighted N =340,403,754) for statistical analysis.<br>Age: Not reported<br>EC use at baseline: assume very low in 2002; because E-cigarettes first appeared on the US market in 2007.<br>Gender/sex: Not reported<br>Ethnicity/race: Not reported<br>Measures of socioeconomic status: Not reported
Exposures	Electronic cigarettes Use: not applicable<br>Details on EC devices: note applicable<br>Electronic cigarettes availability: prevalence of use
Outcomes	Methods:" Aim was to examine and compare trends in past 30-day cigarette smoking among adolescents in the US from 2002 to 2019, before and after the onset of the “e-cigarette era” in 2014. Interrupted time series analyses were used to examine changes in cigarette smoking over time and compare trends in cigarette smoking pre- and post-2014. Models were applied to the full sample and stratified by middle (6th–8th grade) and high school (9th–12th grade). "<br>Type of combustible tobacco use: cigarettes<br>Combustible tobacco use: past 30 days

Study funding	"Research reported in this paper was supported by grant number [R01-CA239097] from the National Cancer Institute and by the University of Texas Health Science Center at Houston School of Public Health Cancer Education. Additional support was provided by the Career Development Program – National Cancer Institute/NIH Grant – National Cancer Institute/NIH Grant T32/CA057712 (Author: DSM).<br>The content is solely the responsibility of the authors and does not necessarily represent the official views of the National Cancer Institute or the National Institutes of Health. The manuscript team received partial funding from the Michael & Susan Dell Foundation to the Michael & Susan Dell Center for Healthy Living and contributions from the UTHealth School of Public Health to complete the analyses and produce the manuscript."
Author declarations	"The authors declare the following financial interests/personal relationships which may be considered as potential competing interests: Drs Harrell, Mantey, and Kelder are a consultants in litigation against the vaping industry. This does not alter our adherence to policies on sharing data and materials."

Hawkins SS, Kruzik C, O'Brien M, Levine Coley R. Flavoured tobacco product restrictions in Massachusetts associated with reductions in adolescent cigarette and e-cigarette use. Tobacco Control 2022;31(4):576-579.

Study characteristics

Methods	Design: difference in difference models (Population level study)<br>Recruitment: via biennial Massachusetts Youth Health Surveys<br>Setting: high schools<br>Study start date/end date: 2011/2017<br>Number of datapoints: 14<br>Primary dataset: MYHS<br>Country: USA
Participants	Total N: 9988 for cigarette use and 10 168 for e-cigarette use.<br>Age: 14-17 years<br>EC use at baseline: 3.9%<br>Gender/sex: Female 49%<br>Ethnicity/race: 67% White, 15% Hispanic, 9% Black and 5% Asian<br>Measures of socioeconomic status: NR
Exposures	Electronic cigarettes Use: ever use, past 30 days use<br>Details on EC devices: N/A<br>Electronic cigarettes availability: Flavour restrictions, age of sales restrictions, setting restrictions
Outcomes	Methods: “We conducted difference-in-differences models to link changes in county-level tobacco-control policies to changes in adolescents’ use of cigarettes and e-cigarettes using 2011–2017 biennial Massachusetts Youth Health Surveys. “<br>Type of combustible tobacco use: cigarettes<br>Combustible tobacco use: lifetime and prior<br>Level of cigarette use after changes in county-level tobacco-control policies.

Study funding	"This work was supported in part by a grant from the American Lung Association (PP- 625245) (PI: S.S.H.)."
Author declarations	"None declared."

Huang LL, Kowitt SD, Sutfin EL, Patel T, Ranney LM, Goldstein AO. Electronic Cigarette Use Among High School Students and Its Association With Cigarette Use And Smoking Cessation, North Carolina Youth Tobacco Surveys, 2011 and 2013. Preventing Chronic Disease 2016;13:E103.

Study characteristics

Methods	Design: Longitudinal cohort (Individual level study)<br>Recruitment: via North Carolina Youth Tobacco Survey (2011, 2013). The NCYTS is a voluntary, anonymous, school-based survey of middle and high school students administered biannually since 1999. The NCYTS survey uses a 2-stage cluster probability sampling design to produce a representative sample of students in grades 6 through 12.<br>Setting: high schools<br>Study start date/end date: 2011/2013<br>Number of datapoints: 2<br>Primary dataset: NCYTS<br>Country: USA
Participants	Total N: 4092<br>Age: NR<br>EC use at baseline: 1.7%<br>Gender/sex: Female 48.9%; Male 51.1%<br>Ethnicity/race: Non-hispanic black 27.3%; non-hispanic white: 54.0% Non-hispanic other 7.5%; hispanic 11.2%<br>Measures of socioeconomic status: NR
Exposures	Electronic cigarettes Use: ever use, past-30 days use<br>Details on EC devices: NR<br>Electronic cigarettes availability: Not applicable
Outcomes	Methods: "Data came from high school students who completed the school-based, cross-sectional North Carolina Youth Tobacco Survey in 2011 and 2013. This study assessed changes in prevalence of e-cigarette and cigarette use from 2011 through 2013, and cessation-related factors associated with those students' current and past use of e-cigarettes in 2013. Multinomial logistic regression analyses were conducted to examine associations between predictors (ie, cigarette use, quit intention, quit attempt, length of last abstinence period) and use of 3 outcome categories: never, past, and current e-cigarette use in 2013. Adjusted relative risk ratios (RRRs) were calculated in reference to the base group (ie, never e-cigarette users). Separate models were used for each cessation-related predictor because of collinearity between the predictors."<br>Type of combustible tobacco use: cigarette<br>Combustible tobacco use: ever use, past-30 days use<br>Adjustment for confounders: "All models adjusted for sociodemographic variables."

	Relative risk ratio of cigarette smoking by past and current e-cigarette use.
Study funding	"This work was supported by the National Cancer Institute at the National Institutes of Health and the Center for Tobacco Products at the Food and Drug Administration (P50CA180907); and the Centers for Disease Control and Prevention (DP 14-1415). The content is solely the responsibility of the authors and does not necessarily represent the official views of the National Institutes of Health, the Food and Drug Administration, or the Centers for Disease Control and Prevention."
Author declarations	"The authors declare no conflicts of interest."

Huang S, Chen Q, Griffin P, Liu G, Azagba S. Longitudinal transitions in tobacco use in youth and young adults: A latent transition analysis of the population assessment of tobacco and health study from Wave 1 to 5. Addict Behav 2023; 138:107548.

Study characteristics

Methods	Design: Longitudinal cohort (individual level study)<br>Recruitment: via PATH<br>Setting: nationally representative sample of U.S youth<br>Study start date/end date: Wave 1 (September 2013)/ Wave 5 (November 2019)<br>Number of datapoints: 5<br>Primary dataset: PATH<br>Country: USA
Participants	Total N: 7575<br>Age: 12-18+ years<br>EC use at baseline: No current use 97.3%, Infrequent use 2.0%, Frequent use 0.2%<br>Gender/sex: Female n=3721 (49.3%) , Male n=3834 (50.7%)<br>Ethnicity/race:<br>White n=4,776 (63.1%)<br>Black n=1,217 (16.1%)<br>Other n=1,149 (15.2%)<br>Measures of socioeconomic status:<br>Parent education<br>Less than high school n=1580 (20.9%)<br>High school or equivalent n=1381 (18.2%)<br>Some college/ associates degree n=2326 (30.7%)<br>Bachelor's degree n=1441 (19.0%)<br>Advanced degree n=799 (10.6%)

Exposures	Electronic cigarettes Use: past 30 days<br>Details on EC devices: not reported<br>Electronic cigarettes availability: not applicable
Outcomes	Methods: "aimed to understand transitions in tobacco use patterns among these groups and the factors that affect transition patterns. Used five waves of data from the PATH Study (2013–2019), and conducted latent class analysis and latent transition analysis to understand tobacco use classes and the longitudinal transitions between classes. "<br>Adjustment of confounders: "Adjusted for covariates, including demographics, individual behaviors, household environment, and psychosocial factors, to capture their effects on class transition probabilities."<br>Type of combustible tobacco use: Cigarettes. Traditional cigar, Hookah, Pipe, Snus pouches, Smokeless tobacco - put together as combined product category of 'other products'. Cigarillo and Filtered cigar put together as combined category of 'small cigar'.<br>Combustible tobacco use: past 30 days
Study funding	"This work was supported by Penn State Center for Socially Responsible Artificial Intelligence (CSRAI) Seed Funding."
Author declarations	"The authors declare that they have no known competing financial interests or personal relationships that could have appeared to influence the work reported in this paper."

Kasza KA, Edwards KC, Tang Z, Stanton CA, Sharma E, Halenar MJ, et al. Correlates of tobacco product initiation among youth and adults in the USA: findings from the PATH Study Waves 1-3 (2013-2016). Tob Control 2020;29(3):s191-s202.

Study characteristics

Methods	Design: Longitudinal cohort (individual level study)<br>Recruitment: via PATH<br>Setting: nationally representative, longitudinal cohort study of US youth and adults.<br>Study start date/end date: 2013/2016<br>Number of datapoints: 3<br>Primary dataset: PATH<br>Country: USA
Participants	Total N: Never users of at least one type of tobacco product at Wave 1 (W1, 2013/14) or Wave 2 (W2, 2014/15) were included (n=12 987 youth; n=25 116 adults).<br>Age: 12-17 years at baseline<br>EC use at baseline: measured but not reported<br>Gender/sex: measured but not reported<br>Ethnicity/race: measured but not reported<br>Measures of socioeconomic status: not reported
Exposures	Electronic cigarettes Use: everuse, past week use

	Details on EC devices: not reported<br>Electronic cigarettes availability: not applicable
Outcomes	Methods: "report on demographic and tobacco product use correlates of tobacco product initiation (cigarettes, ENDS, cigars, hookah and smokeless tobacco) among the US population. Data were from the first three waves (2013–2016) of the PATH study. Never users of at least one type of tobacco product at Wave 1 (W1, 2013/14) or Wave 2 (W2, 2014/15) were included (n=12 987 youth; n=25 116 adults). Generalised estimating equations were used to evaluate the association between demographic and tobacco product use characteristics at baseline, and tobacco product initiation at follow-up (ever, past 30 day, frequent (use on 20 or more of the past 30 days)) over two 1-year periods (W1–W2 and W2–Wave 3)."<br>Adjustment for confounders: "Analyses were adjusted for age group, sex, race/ethnicity, each tobacco use correlate and wave."<br>Type of combustible tobacco use: cigarette, cigar, hookah<br>Combustible tobacco use: everuse, past 30-day use, frequent use frequent (use on 20 or more of the past 30 days)
Study funding	"This study is supported by Federal funds from the National Institute on Drug Abuse, National Institutes of Health and the Center for Tobacco Products, Food and Drug Administration, Department of Health and Human Services, under a contract to Westat (Contract No. HHSN271201100027C)."
Author declarations	"WMC reports long- term stock holdings in General Electric Company, 3M Company, and Pfizer Incorporated, unrelated to this manuscript. No financial disclosures were reported by the other authors of this paper."

Keller-Hamilton B, Lu B, Roberts ME, Berman ML, Root ED, Ferketich AK. Electronic cigarette use and risk of cigarette and smokeless tobacco initiation among adolescent boys: A propensity score matched analysis. Addictive Behaviors 2021; 114:106770.

Study characteristics

Methods	Design: Longitudinal cohort (Individual level study)<br>Recruitment: via Buckeye Teen Health Study<br>Setting: community (address-based sampling)<br>Study start date/end date: January 2015/ June 2016<br>Number of datapoints: 4<br>Primary dataset: Buckeye Teen Health Study<br>Country: USA
Participants	Total N: 1220<br>Age: 11-16 years at baseline<br>EC use at baseline: unclear<br>Gender/sex: Male 100%<br>Ethnicity/race: Minority race/ethnicity E-cigarette ever users:20.0%; E-cigarette never users: 20.4%<br>Measures of socioeconomic status: Parent graduated college 54.4 %
Exposures	Electronic cigarettes Use: Ever use<br>Details on EC devices: Not reported

	Electronic cigarettes availability: Not applicable
Outcomes	Methods: “Boys from urban and Appalachian Ohio (N = 1220; ages 11–16 years at enrollment) reported use of e-cigarettes, cigarettes, and SLT at baseline and every six months for two years. A propensity score matching design was implemented, matching one e-cigarette user to two similar e-cigarette non-users. This analysis was completed in 25 multiple imputed datasets to account for missing data. Risk ratios (RRs) comparing risk of initiating cigarettes and SLT for e-cigarette users and nonusers were estimated. “<br>Adjustment for confounders: “confounders were adjusted in propensity to smoke score.”<br>Type of combustible tobacco use: cigarette<br>Combustible tobacco use: ever use; Past-30 days use<br>Risk ratios (RRs) comparing risk of initiating cigarettes and SLT for e-cigarette users and nonusers
Study funding	“This work was supported by grant P50CA180908 from the National Cancer Institute and Food and Drug Administration’s Center for Tobacco Products. The content is solely the responsibility of the authors and does not necessarily represent the official views of the NIH or the Food and Drug Administration.”
Author declarations	“The authors declare that they have no known competing financial interests or personal relationships that could have appeared to influence the work reported in this paper.”

Kinnunen JM, Ollila H, Minkkinen J, Lindfors PL, Timberlake DS, Rimpela AH. Nicotine matters in predicting subsequent smoking after e-cigarette experimentation: A longitudinal study among Finnish adolescents. Drug and Alcohol Dependence 2019(7513587):182-187.

Study characteristics

Methods	Design: Longitudinal cohort (Individual level study)<br>Recruitment: via Metropolitan Longitudinal Finland (MetLoFIN) Study<br>Setting: Schools<br>Study start date/end date: Spring 014/ 2016<br>Number of datapoints: 2<br>Primary dataset: MetLoFIN<br>Country: Finland
Participants	Total N: 3474 (2016 for analyses for daily smoking)<br>Age: 15-16 years<br>EC use at baseline: Tried only non-nicotine e-cigarettes 4.5% (n=151), Tried nicotine e-cigarettes 24.9% (n=839)<br>Gender/sex: females 51.8% (n=1798), males 48.2%(n=1676)<br>Ethnicity/race: NR<br>Measures of socioeconomic status: NR
Exposures	Electronic cigarettes Use: Ever use<br>Details on EC devices: nicotine and non-nicotine e-cigarettes<br>Electronic cigarettes availability: Not applicable

Outcomes	Methods: "A survey was conducted in lower secondary schools of the Helsinki metropolitan area, Finland, with 15 − 16-year-olds in 2014 (baseline) and in upper secondary schools in 2016 when the cohort was 17 − 18-year-olds (follow-up). Firth logistic regression and generalized linear mixed models (GLMM) were used. "<br>Adjustment for confounders: "Adjusting variables included gender but not age, as it varied only slightly among the students. The adjusted models included also socioeconomic background, measured with parents' education, and other tobacco product (snus and waterpipe) and drug use to control for possible confounders."<br>Type of combustible tobacco use: cigarette<br>Combustible tobacco use: ever use<br>Odds ratios of use of nicotine e-cigarettes predicting the uptake of daily smoking at follow-up.
Study funding	"Nothing declared."
Author declarations	"Nothing declared."

Kintz N, Liu M, Chou C-P, Urman R, Berhane K, Unger JB, et al . Risk factors associated with subsequent initiation of cigarettes and e-cigarettes in adolescence: A structural equation modeling approach. Drug and Alcohol Dependence 2020; 207:107676.

Study characteristics

Methods	Design: Longitudinal cohort (Individual level study)<br>Recruitment: via Southern California Children's Health Study (CHS)<br>Setting: Schools<br>Study start date/end date: January 2014 to June 2014/ February 2015 and March 2016<br>Number of datapoints: 2<br>Primary dataset: CHS<br>Country: USA
Participants	Total N: 1293 (follow up)<br>Age: 17.3 (0.6) years at baseline<br>EC use at baseline: n=66 (41.8%)<br>Gender/sex: no cig initiation: Female 576 (86.9%) Male 482 (79.9%); with cig initiation Female 87 (13.1%) Male 121 (20.1)<br>Ethnicity/race: no cig initiation Non-Hispanic white 399 (81.43%); Hispanic white 507 (84.08%), Other 152 (87.86%); for no cig initiation Non-Hispanic white 91 (18.57%), Hispanic white 96 (15.92%), Other 21 (12.14%)<br>Measures of socioeconomic status: Parental Education no cig initiation High School or less 256 (84.5 %), Some college 308 (82.4%), College or above 389 (83.3%), Missing 105 (86.1%); cig initiation High School or less 47 (15.5%), Some college 66 (17.6%), College or above 78 (16.7%), missing 17 (13.9%)
Exposures	Electronic cigarettes Use: Ever use<br>Details on EC devices: NR<br>Electronic cigarettes availability: Not applicable

Outcomes	Methods: “Structural equation models (SEM) were developed to investigate associations of susceptibility, marketing, and the social environment (as latent factors), and other tobacco use at baseline with cigarette or e-cigarette initiation between baseline and follow-up. Analyses were restricted to baseline never cigarette users (N = 1293) for models evaluating cigarette initiation, and to never e-cigarette users (N = 1197) for models evaluating e-cigarette initiation. “<br>Adjustment for confounders: “confounders included in the SEM model looking at Relationship between baseline susceptibility, marketing and social environment with subsequent cigarette Initiation at follow-up.”<br>Type of combustible tobacco use: cigarette, cigar<br>Combustible tobacco use: ever use, other<br>Odds ratios of e-cigarette use with cigarette initiation.
Study funding	“Research reported in this publication was supported by grant numbers P50CA180905 and U54CA180905 from the National Cancer Institute at the National Institutes of Health (NIH) and the Food and Drug Administration (FDA) Center for Tobacco Products (CTP), and grant number K01DA042950 from the National Institute for Drug Abuse at NIH. The funders had no role in the design and conduct of the study; collection, management, analysis, or interpretation of the data; or preparation, review, or approval of the manuscript.”
Author declarations	“The authors have no conflicts of interest relevant to this article to disclose. Robert Urman began a position at Amgen on April 15, 2019 and did not contribute to the paper after that date.”

Kowitt SD, Anshari D, Orlan EN, Kim K, Ranney LM, Goldstein AO, et al. Impact of an e-cigarette tax on cigarette and e-cigarette use in a middle-income country: A study from Indonesia using a pre-post design. BMJ Open 2022;12(5): e055483.

Study characteristics

Methods	Design: Pre-post online survey (Population level study)<br>Recruitment: via “Facebook and Instagram ads to recruit Indonesian participants before the e-liquid tax (Finance Ministerial Regulation No. 146/010/2017) went into effect on 1 October 2018.”<br>Setting: community<br>Study start date/end date: 16–21 September 2018 / 8 November through 3 December 2018<br>Number of datapoints: 2<br>Primary dataset: original dataset<br>Country: Indonesia
Participants	Total N: 1039<br>Age: 18–24 n=679 (65.4)<br>EC use at baseline: Non-daily n= 250 (24.1%) ; Daily n= 789 (75.9%)<br>Gender/sex: N.B. Across all age categories Male n=1001 (96.4%); Female n=37 (3.6%)<br>Ethnicity/race: NR<br>Measures of socioeconomic status: Across all age categories<br>Income, per month

	IDR 0–1.5 million (<US$103) 334 (32.3)<br>IDR 1.5–3.5 million (US$103–US$240) 400 (38.7)<br>IDR 3.5–7 million (US$240–US$480) 2239 (3.1)<br>More than IDR 7 million (>US$480) 62 (6.0)
Exposures	Electronic cigarettes Use: Past-7 days e-cigarette use, e-cigarette use status, e-cigarette use frequency<br>Details on EC devices: NR<br>Electronic cigarettes availability: e-liquid tax
Outcomes	Methods: “We conducted a pre–post online survey of a cohort of adults in Indonesia. We analysed data from all participants who were eligible for and completed both surveys, regardless of whether there were missing data. We first observed if there was change over time in the variables. To assess change in continuous variables, we used paired t-tests. To assess change in dichotomous variables, we used McNemar’s test. To assess change in ordinal variables, we used a Wilcoxon signed-rank test, which is appropriate for paired ordinal data “<br>Comparator: “On 1 October 2018, Indonesia implemented its first e-cigarette regulation, a 57% ad-valorem tax on the retail price of e-cigarette liquid (e-liquid) applied at the manufacturer level, which exceed the 40% average tax on cigarettes.”<br>Type of combustible tobacco use: cigarettes<br>Combustible tobacco use: ‘How often, if at all, do you currently smoke cigarettes?’<br>Effects of the e-liquid tax in cigarettes use among adults in Indonesia.
Study funding	“This work was supported by an award from the Institute for Global Tobacco Control at the Johns Hopkins Bloomberg School of Public Health with funding from the Bloomberg Initiative to Reduce Tobacco Use.”
Author declarations	“None declared.”

Lee P, Fry J. Investigating gateway effects using the PATH study. F1000Res 2019; 8:264.

Study characteristics

Methods	Design: Longitudinal cohort (individual level study)<br>Recruitment: via PATH<br>Setting: Nationally representative dataset<br>Study start date/end date: 2013–2014/ 2014-2015<br>Number of datapoints: 2<br>Primary dataset: PATH<br>Country: USA
Participants	Total N: 10671<br>Age: 12-17 years

	EC use at baseline: measured but not reported<br>Gender/sex: measured but not reported<br>Ethnicity/race: measured but not reported<br>Measures of socioeconomic status: Highest grade or year at school completed by parent; measured but not reported
Exposures	Electronic cigarettes Use: ever use<br>Details on EC devices: not reported<br>Electronic cigarettes availability: not applicable
Outcomes	Methods: "The main analyses considered Wave 1 never cigarette smokers who, at Wave 2, had data on smoking initiation. Constructed a propensity score for ever e-cigarette use from Wave 1 variables, using this to predict ever cigarette smoking. Sensitivity analyses accounted for other tobacco product use, linked current e-cigarette use to subsequent current smoking, or used propensity scores for ever smoking or ever tobacco product use as predictors. Also considered predictors using data from both waves, attempting to reduce residual confounding from misclassified responses."<br>Adjustment for confounders: "Wave 1 data were used to develop a propensity score for e-cigarette use based on the five demographic variables (age, sex, Hispanic origin, race, region) and on 60 smoking predictor variables." The final model used 12 predictor variables.<br>Type of combustible tobacco use: cigarette<br>Combustible tobacco use: ever use
Study funding	"Financial support was provided by Philip Morris Products SA, through Project Agreement No. 19 with P N Lee Statistics and Computing Ltd. The funders had no role in study design, data collection and analysis, decision to publish, or preparation of the manuscript."
Author declarations	"Peter Lee, director of P N Lee Statistics and Computing Ltd, is an independent consultant to a number of tobacco companies. John Fry is a former employee of Peter Lee's company."

Leventhal AM, Strong DR, Kirkpatrick MG, Unger JB, Sussman S, Riggs NR, et al . Association of Electronic Cigarette Use With Initiation of Combustible Tobacco Product Smoking in Early Adolescence. JAMA 2015;314(7):700-7

Study characteristics

Methods	Design: Longitudinal cohort (Individual level study)<br>Recruitment: via a longitudinal survey of substance use and mental health among high school students in approximately 40 public high schools in the Los Angeles metropolitan. Parental and students' consents were sought.<br>Setting: High Schools<br>Study start date/end date: Fall 2013/Fall 2014<br>Number of datapoints: 3<br>Primary dataset: longitudinal study conducted in 40 public high schools in Los Angeles County, California<br>Country: USA
Participants	Total N: 2530<br>Age: 14.1 years (9th Grade)

	EC use at baseline: Ever Use n = 222<br>Gender/sex: Males 1181 (46.8%); Females 1343 (53.2%)<br>Ethnicity/race: American Indian/Alaska Native 21 (0.8%), Asian 472 (19.0%), Black 119 (4.8%), Hispanic 1099 (44.2%), Native Hawaiian/Pacific Islander 89 (3.6%), White 404 (16.2%), Other 142 (5.7%), Multiethnic or multiracial 141 (5.7%)<br>Measures of socioeconomic status: Highest parental education level ≤8th grade 72 (3.3%); Some high school 171 (7.8%); High school graduate 334 (15.2%); Some college 428 (19.5%); College graduate 741 (33.7%); Graduate degree 454 (20.6%)
Exposures	Electronic cigarettes Use: Ever use, use in past 6 months<br>Details on EC devices: Not reported<br>Electronic cigarettes availability: Not applicable
Outcomes	Methods: "Primary analyses used repeated-measures, generalized- linear mixed models, an extension of logistic regression, in which each participant had 2 time points of follow-up data (at 6 and 12 months). Separate models were constructed for each binary outcome (ie, any combustible tobacco product, cigarettes, cigars, hookah) and the ordinal number of combustible products (cumulative logit) outcome at the 6- and 12-month follow-up periods. "<br>Adjustment for confounders: "All models included baseline e-cigarette use, school, and<br>time (6-month vs 12-month follow-up) as fixed effects and were fit with and without adjustment for all covariates." Covariates included sociodemographic characteristics, environmental factors and intrapersonal factors.<br>Type of combustible tobacco use: cigarette, cigarillo, cigars<br>Combustible tobacco use: ever use, use in past 6 months<br>Odds ratios of combustible tobacco use at follow up after baseline e-cigarette use.
Study funding	"This research was supported by grants R01-DA033296 and P50-CA180905 from the National Institutes of Health."
Author declarations	"The authors have completed and submitted the ICMJE Form for Disclosure of Potential Conflicts of Interest and none were reported."

Leventhal AM, Stone MD, Andrabi N, Barrington-Trimis J, Sussman S Strong DR, et al . Association of e-cigarette vaping and progression to heavier patterns of cigarette smoking. JAMA 2016;316(18):1918-1920.

Study characteristics

Methods	Design: Longitudinal cohort (Individual level study)<br>Recruitment: via longitudinal study conducted in 10 public high schools in Los Angeles County, California. Parental consent and student consent were sought.<br>Setting: High Schools<br>Study start date/end date: Fall 2014/Fall 2015<br>Number of datapoints: 2<br>Primary dataset: longitudinal study conducted in 10 public high schools in Los Angeles County, California<br>Country: USA

Participants	Total N: 3084<br>Age: 15.5 years (10th Grade)<br>EC use at baseline: prior use n=730 (23.6%), infrequent n=133 (4.3%), frequent vaper n=146 (4.7%)<br>Gender/sex: Female 54.3%<br>Ethnicity/race: Hispanic 47.3%<br>Measures of socioeconomic status: Not reported
Exposures	Electronic cigarettes Use: Past 30 days use (never, prior [ever-vaper with no past 30-day vaping], infrequent [vaped 1-2 days during past 30 days], or frequent [vaped ≥3 days]).<br>Details on EC devices: Not reported<br>Electronic cigarettes availability: Not applicable
Outcomes	Methods: "Generalized estimating equation ordinal (cumulative logit) logistic regression models were used to assess the association between baseline vaping and follow-up frequency or heaviness of smoking, with adjustment for baseline smoking frequency or heaviness using SAS (SAS Institute). The baseline vaping × baseline smoking interaction term was then added to test differential associations of baseline vaping with follow-up smoking by baseline smoking status. "<br>Adjustment for confounders: "Each model was retested after adjusting for age, sex, ethnicity, highest parental education, whether the student lived with both parents, ever use of alcohol or drugs, ever use of any combustible tobacco product, family history of smoking, depressive symptoms (Cronbach α = .94), UPPS Impulsive Behavior Scale lack of premeditation (α = .94) and sensation-seeking (α = .91) subscales, delinquent behavior (α = .81), peer smoking, smoking susceptibility (α = .87), and smoking expectancies (α = .46)."<br>Type of combustible tobacco use: cigarette<br>Combustible tobacco use: past 30-days use (nonsmoker, infrequent smoker [1-2 days], frequent smoker [≥3 days]) and heaviness (0, <1, 1, or ≥2 cigarettes per day on smoking days).<br>Odds ratios of stronger smoking frequency after baseline vaping.
Study funding	"This research was supported by grants R01-DA033296 and P50-CA180905 from the National Institutes of Health."
Author declarations	"The authors have completed and submitted the ICMJE Form for Disclosure of Potential Conflicts of Interest and none were reported."

Levy DT, Warner KE, Cummings KM, Hammond D, Kuo C, Fong GT, et al. Examining the relationship of vaping to smoking initiation among US youth and young adults: a reality check. Tob Control. 2019 Nov 1;28(6):629

Study characteristics

Methods	Design: Trend time analysis<br>Recruitment: via MTF, NYTS, YRBS, NSDUK and NHIS<br>Setting: nationally representative surveys

	Study start date/end date: 2011/Fall 2017<br>Number of datapoints: 7<br>Primary datasets: Monitoring the Future (MTF) survey; National Youth Tobacco Survey (NYTS); Youth Risk Behavior Survey (YRBS); National Survey of Drug Use and Health (NSDUH); and National Health Interview Survey (NHIS) for young adults.<br>Country: USA
Participants	Total N: Not reported<br>Age: 12- 25 years<br>EC use at baseline: NYTS Past-30 days vaping in high schoolers 2011 1.6%; MTF 2014 16.3% for 10th graders and 17.2% for 12th graders; NATS 2013-2014 18-24 years 5.5%; NHIS 2014 18-24 years 5.1%<br>Gender/sex: Not reported<br>Ethnicity/race: Not reported<br>Measures of socioeconomic status: Not reported
Interventions	Electronic cigarettes Use: Past-30 days use<br>Details on EC devices: Not reported<br>Electronic cigarettes availability: Not applicable
Outcomes	Methods: The authors first ascertained trends in vaping after searching for nationally representative surveys on youth and young adult vaping. They conducted a time trend analysis using data from five US surveys.<br>Type of combustible tobacco use: cigarette<br>Combustible tobacco use: past 30d use; established smoking (daily and half-pack-a-day smoking prevalence in MTF and NSDUH surveys, prevalence of current smokers who had smoked more than 100 cigarettes lifetime in NHIS).<br><br>Temporal relationship between vaping and youth smoking using multiple data sets to explore the question of whether vaping promotes smoking initiation in the USA.
Study funding	“RB, KMC, GTF, MLG, DH, DTL and JFT received funding from the National Cancer Institute under grant P01CA200512.”
Author declarations	“MLG was a member of the National Academies of Sciences, Engineering, and Medicine (NASEM) Committee on the Review of the Health Effects of Electronic Nicotine Delivery Systems who wrote the report. The report was funded by the Food and Drug Administration (FDA), but the FDA was not involved in the drafting or review of the NASEM Report or this manuscript. The policy implications written in this manuscript are the views of the authors and do not necessarily represent the views of the other members of the Committee, the NASEM or the FDA. MLG also received a research grant and served as an advisory board member to pharmaceutical companies that manufacture smoking cessation medications. KMC and DH have served as an expert witness in litigation against the cigarette industry.”

Lin LY, Chien YN, Chen YH, Shean R, Wu CY, Huang SC, et al . E-cigarettes and smoking cessation among adolescent smokers. Scientific Reports 2022;12(1):19489.

Study characteristics

Methods	Design: Longitudinal cohort (Individual level study)<br>Recruitment: via Taiwan Adolescent to Adult Longitudinal Study (TAALS)<br>Setting: survey-based<br>Study start date/end date: 2015/ 2017<br>Number of datapoints: 2<br>Primary dataset: TAALS<br>Country: Taiwan
Participants	Total N: 474<br>Age: 17.9 (SD1.3) years<br>EC use at baseline: n=143<br>Gender/sex: Male 365; Female 109<br>Ethnicity/race: Not reported<br>Measures of socioeconomic status: Father's highest education level: Below junior high school 136; Senior or vocational high school 227, Above college 111; Mother's ethnicity: Han Chinese 387; indigenous 57; Foreigner 30 ; Parents' employment status Full-time 428; Part-time 18; Unemployed 28
Exposures	Electronic cigarettes Use: past 30 days use<br>Details on EC devices: Not reported<br>Electronic cigarettes availability: Not applicable
Outcomes	Methods: "The study uses data from the 2015 (baseline) and from the 2017 (follow-up) waves of the Taiwan Adolescent to Adult Longitudinal Study (TAALS), which is a large nationwide representative cohort study of health behaviors among adolescents in Taiwan. The authors analyzed the data using logistic regression and multivariate regression with a post-stratifcation weighting procedure."<br>Adjustment for confounders: "The multivariable logit regression model was adjusted for ever use of other tobacco products, depression (CES-D), peer support, father's education, mother's ethnicity, parents' employment status, sex, age, and family living arrangement."<br>Type of combustible tobacco use: cigarettes<br>Combustible tobacco use: ever use, past-30 days use<br>Transitions between e-cigarette and cigarette use.
Study funding	"The work was supported by the Health Promotion Administration, Ministry of Health and Welfare,Taiwan (Grant Number: MOHW105-HPA-H-114-133708), from Taiwan's Health and Welfare Surcharge on Tobacco Products—Grant Number: 03724606—Project Code: 1051218-107), and grants R01DA043950 from the US National Institute of Drug Abuse and P50CA180890 from the US National Cancer Institute, and the US Food and Drug Administration (FDA) Center for Tobacco Products. Te content is solely the responsibility of the authors and does not necessarily represent the ofcial views of Health Promotion Administration, US National Institutes of Health (NIH), or the US Food and Drug Administration. The funding agencies had no role in study design, data collection, analysis, and interpretation, or writing of this study. The corresponding author had full access to all data in the study and had fnal responsibility for the decision to submit for publication."
Author declarations	"The authors declare no competing interests."

Loukas A, Marti NC, Cooper M, Pasch KE, Perry CL. Exclusive e-cigarette use predicts cigarette initiation among college students. Addictive Behaviors 2018;76:343-347.

Study characteristics

Methods	Design: Longitudinal cohort (Individual level study)<br>Recruitment: via Marketing and Promotions across Colleges in Texas project (Project M-PACT)<br>Setting: Colleges<br>Study start date/end date: November 2014–February 2015/ approximately 18 months after baseline (the three subsequent waves were collected approximately every six months)<br>Number of datapoints: 4<br>Primary dataset: Project M-PACT<br>Country: USA
Participants	Total N: 2558<br>Age: 19.71 (SD 1.61) years<br>EC use at baseline: 22.2%<br>Gender/sex: Male 32.3%<br>Ethnicity/race: White 31.8%, Hispanics/Latinos 27.4%, African American 9.8%, Asian 23.4%, Other 7.5%<br>Measures of socioeconomic status: Not reported
Exposures	Electronic cigarettes Use: ever use<br>Details on EC devices: Not reported<br>Electronic cigarettes availability: Not applicable
Outcomes	Methods: "This study examined if: a) cigarette-naïve young adults (i.e., never cigarette users) who ever used ENDS had a greater odd of initiating cigarettes than non-ENDS users over a 1.5-year period and b) the odds of cigarette initiation was consistent across exclusive ENDS users and users of ENDS and at least one tobacco product. Multivariable, multilevel discrete-time hazard models were fit using the R glmer function to evaluate if ENDS use predicted cigarette initiation over the 1.5-year period. Discrete-time hazard models are applied when the exact time of the event cannot be identified."<br>Type of combustible tobacco use: cigarettes<br>Combustible tobacco use: ever use<br>Association between cigarette initiation and previous e-cigarettes use.
Study funding	"Research reported in this publication was supported by grant number [1 P50 CA180906] from the National Cancer Institute and the Food and Drug Administration (FDA) Center for Tobacco Products. The content is solely the responsibility of the authors and does not necessarily represent the official views of the NIH or the FDA."

Author declarations	"All authors declare that they have no conflicts of interest."

Loukas A, Marti CN, Harrell MB. Electronic nicotine delivery systems use predicts transitions in cigarette smoking among young adults. Drug Alcohol Depend 2022;231:109251.

Study characteristics

Methods	Design: Longitudinal cohort (individual level study)<br>Recruitment: via Project M-PACT<br>Setting: Marketing and Promotions across Colleges in Texas project (Project M-PACT), a longitudinal, web-based, study that assessed tobacco use across a 4.5-year<br>period from 2014 to 2019.<br>Study start date/end date: 2014/2019<br>Number of datapoints: 8<br>Primary dataset: Project M-PACT<br>Country: USA
Participants	Total N: 5029<br>Age: 18-29 years, mean 21 (SD 2.3)<br>EC use at baseline: measured but not reported<br>Gender/sex: Male 35.8%<br>Ethnicity/race:<br>Non-Hispanic, White 36.1%<br>Hispanic/Latinx 31.0%<br>Black/African American 8.1%<br>Asian 17.4%<br>Other race/ethnicity 7.5%<br>Measures of socioeconomic status: not reported
Exposures	Electronic cigarettes Use: ever use, past 30 days<br>Details on EC devices: not reported<br>Electronic cigarettes availability: not applicable
Outcomes	Methods: "The purpose of this longitudinal study was to examine the role of ENDS use on three transitions in cigarette smoking among young adults; initiation, desistance, and re-uptake. Participants were 5029 18–29-year-olds (64.2% female) enrolled in one of 24 Texas colleges at baseline and involved in an eight-wave, 4.5-year study. A multi-state, continuous time Markov model was used to assess the role of current/past 30-day and ever ENDS use on three

	transitions, spanning at least six months 1) never to current smoking (initiation); 2) current to non-current smoking (desistance); and 3) non-current to current smoking (re-uptake). The model also contained time-invariant socio-demographic, and time-varying intrapersonal (other tobacco use, nicotine dependence, sensation seeking, depressive symptoms) and interpersonal (peer cigarette use) covariates."<br>Type of combustible tobacco use: cigarettes<br>Combustible tobacco use: ever use, past 30 days
Study funding	"This work was supported by the National Institutes of Health [1P50CA180906; R01CA249883; R01CA239097], from the National Cancer Institute (NCI) and the FDA Center for Tobacco Products (CTP). The content is solely the responsibility of the authors and does not necessarily represent the official views of the National Institutes of Health (NIH) or the Food and Drug Administration (FDA)."
Author declarations	"MBH is a consultant in litigation involving the vaping industry."

Lozano P, Barrientos-Gutierrez I, Arillo-Santillan E, Morello P, Mejia R, Sargent JD, et al T. A longitudinal study of electronic cigarette use and onset of conventional cigarette smoking and marijuana use among Mexican adolescents. Drug Alcohol Depend 2017;180:427-430.

Study characteristics

Methods	Design: Longitudinal cohort (individual level study)<br>Recruitment: Survey given in school<br>Setting: Middle school<br>Study start date/end date: February 2016/November 2016<br>Number of datapoints: 2<br>Primary dataset: A school-based, longitudinal survey was conducted in 60 public middle schools from the three largest cities in Mexico (Mexico City, Guadalajara, and Monterrey)<br>Country: Mexico
Participants	Total N: 6574<br>Age: 11-12 years 33%, 13 or more 67%<br>EC use at baseline: ever tried EC 5%<br>Gender/sex: Female 52%, Male 48%<br>Ethnicity/race: not reported<br>Measures of socioeconomic status:<br>Parental education (%)<br>Primary 16<br>Secondary 38<br>High school 19<br>University 19<br>Unknown 8

Exposures	Electronic cigarettes Use: ever use<br>Details on EC devices: not reported<br>Electronic cigarettes availability: not applicable
Outcomes	Methods: "evaluated whether e-cigarette trial among Mexican adolescents increased the likelihood of trial and use of conventional cigarettes or marijuana use at follow-up. A school-based longitudinal survey was conducted in 60 public middle schools from the three largest cities in Mexico. Students (12–13 years old) were surveyed in 2015 and followed up 20 months later (n = 6574). Generalized estimating equations models were used to evaluate the association between e-cigarette trial at baseline and conventional cigarettes smoking and marijuana use at follow-up."<br>Adjustment for confounders: "Models were adjusted for: sex, age, parent SES, sensation seeking, friends that smoke, parents that smoke, siblings that smoke, tried alcohol, binge drinking and internet tobacco product advertising."<br>Type of combustible tobacco use: cigarette<br>Combustible tobacco use: past 30 days
Study funding	"This research was supported by a grant from the Fogarty International Center and the National Cancer Institute of the United States' National Institute of Health (R01 TW009274)."
Author declarations	"No conflict declared."

Mantey DS, Cooper MR, Loukas A, Perry CL. E-cigarette Use and Cigarette Smoking Cessation among Texas College Students. American Journal of Health Behavior 2017;41(6):750-759.

Study characteristics

Methods	Design: Longitudinal cohort (Individual level study)<br>Recruitment: via Marketing and Promotions across Colleges in Texas Project (Project M-PACT) (waves 4- 5)<br>Setting: Colleges<br>Study start date/end date: November 2014 to February 2015/October-November 2015<br>Number of datapoints: 3<br>Primary dataset: Project M-Pact<br>Country: USA
Participants	Total N: 627<br>Age: 18-29 years mean 22.2 (SD 3.1) years<br>EC use at baseline: 19.1% (N = 120) reported use of e-cigarettes in the past 30-days for reasons other than cigarette smoking cessation, and 18.5% (N = 116) reported use of e-cigarettes for cigarette smoking cessation.<br>Gender/sex: Female 57.3%<br>Ethnicity/race: non-Hispanic white 50.4%, Hispanic/Latino 28.6%, African-American, 3.2%, Asian-American 9.1%, "other" 8.8%<br>Measures of socioeconomic status: Not reported

Exposures	Electronic cigarettes Use: past-30 days use<br>Details on EC devices: Not reported<br>Electronic cigarettes availability: Not applicable
Outcomes	Methods: “Multi-level, multivariable logistic regression models, accounting for school clustering, examined the impact of self-reported use of e-cigarettes on cigarette smoking status at 6- and 12-month follow-ups. Two mutually-exclusive groups of e-cigarette users were examined: those that used for cigarette smoking cessation and those that used for reasons other than cessation. Baseline covariates included socio-demographics, past quit attempts, nicotine dependence, cigarettes per day, and other tobacco use."<br>Type of combustible tobacco use: cigarettes<br>Combustible tobacco use: past 30 days<br>Odds of cigarette cessation after the use of e-cigarettes.
Study funding	"Research reported in this presentation was supported by grant number [1 P50 CA180906] from the National Cancer Institute and the FDA Center for Tobacco Products (CTP). The content is solely the responsibility of the authors and does not necessarily represent the official views of the NIH or the Food and Drug Administration."
Author declarations	“No conflicts of interest to declare.”

Martinelli T, Candel MJJM, de Vries H, Talhout R, Knapen V, van Schayck CP et al . Exploring the gateway hypothesis of e-cigarettes and tobacco: a prospective replication study among adolescents in the Netherlands and Flanders. Tobacco Control 2021;32:170-178.

Study characteristics

Methods	Design: Longitudinal cohort (Individual level study)<br>Recruitment: via online surveys throughout the Netherlands and Flanders.<br>Setting: High schools<br>Study start date/end date: September 2018/December 2019<br>Number of datapoints: 2<br>Primary dataset: Original dataset<br>Country: Netherlands and Belgium
Participants	Total N: 2185<br>Age: mean 13.62 (95% CI 13.56 to 13.67) years<br>EC use at baseline: Ever use n=191<br>Gender/sex: Females 1160 (53.1%); Males 1025 (46.9%)<br>Ethnicity/race: Dutch 1978 (90.5%); Belgian 76 (3.5%); Polish 8 (0.4%); Turkish 30 (1.4%); German 5 (0.2%); British 4 (0.2%); Moroccan 16 (0.7%); Chinese 6 (0.3%); Italian 5 (0.2%); Spanish 5 (0.2%); Other, don’t want to say or unknown 52 (2.4%)

	Measures of socioeconomic status: Highest parental education Graduate degree 271 (12.4%); College graduate 478 (21.9%); Secondary higher education 368 (16.8%); Vocational education 301 (13.8%); Secondary vocational education 211 (9.7%); Lower vocational education 42 (1.9%); Primary school 14 (0.6%); None 23 (1.1%); Don't know 477 (21.8%)
Exposures	Electronic cigarettes Use: ever use<br>Details on EC devices: Not reported<br>Electronic cigarettes availability: Not applicable
Outcomes	Methods: "Ten high schools were recruited as a convenience sample. The analyses involved (1) associations of baseline e-cigarette use and subsequent tobacco smoking among never smokers; (2) associations of e-cigarette use frequency at baseline and tobacco smoking frequency at follow-up; and (3) the association of baseline tobacco smoking and subsequent e-cigarette use among non-users of e-cigarettes."<br>Adjustment for confounders: "Adjusted for covariates."<br>Type of combustible tobacco use: cigarette, cigar, hookah<br>Combustible tobacco use: ever use, use in past 6 months
Study funding	"This study was funded by NWO (401.16.012)."
Author declarations	"None declared"

Martinez-Loredo V, Gonzalez-Roz A, Dawkins L, Singh D, Murphy JG, MacKillop J. Is E-cigarette Use Associated With Persistence or Discontinuation of Combustible Cigarettes? A 24-Month Longitudinal Investigation in Young Adult Binge Drinkers. Nicotine & Tobacco Research 2022;24(7):962-969.

Study characteristics

Methods	Design: Longitudinal cohort (Individual level study)<br>Recruitment: via two independent studies comprising young adult binge drinkers from Memphis, Tennessee, USA (N = 602) and Hamilton, Ontario, Canada (N = 400)<br>Setting: community<br>Study start date/end date: December 2017/July 2020<br>Number of datapoints: 4<br>Primary dataset: one original US dataset and one original Canadian dataset<br>Country: Canada and USA
Participants	Total N: 1002<br>Age: 22.14 (SD 1.26) years<br>EC use at baseline: monthly or more 167 (16.7%)<br>Gender/sex: Female 572 (57.1%)<br>Ethnicity/race: white 572 (57.1%)<br>Measures of socioeconomic status: Not reported

Exposures	Electronic cigarettes Use: past 30 days, other (frequency of e-cigarette use (none, monthly, weekly, daily, and multiple times daily)<br>Details on EC devices: Not reported<br>Electronic cigarettes availability: Not applicable
Outcomes	Methods: "This study examined longitudinal patterns of combustible tobacco and e-cigarette use over 24 months in young adult binge drinkers. The primary outcomes were past month combustible tobacco and e-cigarette use. Nicotine dependence was measured using the Fagerström Test of Cigarette Dependence. Alcohol severity was measured using the Young Adult Alcohol Consequences Questionnaire. Latent transition analysis (LTA) was used to identify patterns of cigarette smoking and e-cigarette use over 24 months."<br>Type of combustible tobacco use: cigarette<br>Combustible tobacco use: past 30 days, other (frequency of combustible cigarette use (none, monthly, weekly, daily, and multiple times daily)<br>Longitudinal patterns of combustible tobacco and e-cigarette use over 24 months.
Study funding	"This study was supported by the National Institute on Alcohol Abuse and Alcoholism (R01AA024930; JGM and JM), the Canadian Institutes of Health Research (#365297; JM), the Peter Boris Chair in Addictions Research and the Canada Research Chair in Translational Addiction Research."
Author declarations	"JM is a principal in a private company, BEAM Diagnostics, Inc., but no commercial products fall within the scope of the study. No other authors have declarations."

Melka A, Chojenta C, Holliday E, Loxton D. E-cigarette use and cigarette smoking initiation among Australian women who have never smoked. Drug and alcohol review 2021;40(1):68-77.

Study characteristics

Methods	Design: Longitudinal cohort (individual level study)<br>Recruitment: via Australian Longitudinal Study on Women's Health<br>Setting: In 2012/2013 women aged 18–23 years were recruited to form a new young cohort born in 1989–1995<br>Study start date/end date: 2015/2016<br>Number of datapoints: 2<br>Primary dataset: new young cohort of Australian women born in 1989–1995 who participated in the Australian Longitudinal Study on Women's Health.<br>Country: Australia
Participants	Total N: 6710 at baseline (third survey), 5398 followed up (fourth survey)<br>Age: mean (± SD) age at baseline 22.5 (1.7)<br>EC use at baseline: never smokers 268/6710 (4%) had ever used e-cigarettes at baseline<br>Gender/sex: all female<br>Ethnicity/race: not reported<br>Measures of socioeconomic status:<br>Higher level of education, No. (%)

	Less than Year 12 ever EC 6 (2.2), no EC 131 (2.0), Year 12 and equivalent ever EC 91 (34.0), no EC 1708 (26.5) Trade/certificate/diploma ever EC 70 (26.1), no EC 1547 (24.0), University degree ever EC 98 (36.6), no EC 2907 (45.2), Missing ever EC 1 (1.1), no EC 149 (2.3)<br>Employment status, No. (%)<br>Unemployed ever EC 44 (16.4), no EC 696 (10.8), Employed ever EC 221 (82.5), no EC 5596 (86.9), Missing ever EC 1 (1.1), no EC 150 (2.3)
Exposures	Electronic cigarettes Use: ever use<br>Details on EC devices: not reported<br>Electronic cigarettes availability: not applicable
Outcomes	Methods: "This study aimed to determine the association between lifetime e-cigarette use and subsequent initiation of cigarette smoking among tobacco-naïve Australian women aged 20–27. The current study used data (n= 5398) from the third (2015) and fourth (2016) surveys collected from a cohort of Australian women born in 1989–1995 who participated in the Australian Longitudinal Study on Women's Health. Multivariable logistic regression was used to identify the association between lifetime e-cigarette use at the base line survey and initiation of cigarette smoking (smoked 100 cigarettes or more in the last year) at the follow up adjusting for possible confounders. Effects were expressed as odds ratios with 95% confidence interval."<br>Adjustment for confounders: "The final model adjusted for age, education, ability to manage income, ever had depression, Kessler psychological distress scale, Binge drinking, adverse childhood experiences score."<br>Type of combustible tobacco use: cigarette<br>Combustible tobacco use: ever use
Study funding	"We are indebted to the Australian Government Department of Health for financing the study and to the study respondents who voluntarily participated in the study. The findings and conclusions stated in the submitted article are those of the authors and not an official position of the Australian Government Department of Health. The funder was not involved in the analysis, interpretation, report writing and decision to submit the report for publication."
Author declarations	"The authors have no conflicts of interest."

Meng YY, Yu Y, Ponce NA. Cigarette, electronic cigarette, and marijuana use among young adults under policy changes in California. Addict Behav Rep 2022;16:100459.

Study characteristics

Methods	Design: Longitudinal cohort (Individual level study)<br>Recruitment: via California Health Interview Survey (CHIS) 2017–2018<br>Setting: survey-based<br>Study start date/end date: 2017/2018<br>Number of datapoints: 2<br>Primary dataset: CHIS<br>Country: USA
Participants	Total N: 3929<br>Age: 18–25 years, 18–20 years old 43.0%, 21–25 years old 57.0%

	EC use at baseline: 14.9%<br>Gender/sex: Male 51.0%; Female 49.0%<br>Ethnicity/race: Latino 30.0%; White 27.0%; Asian 17.0%; African American 5.0%; Other Single Race/Multiracial 21.0%<br>Measures of socioeconomic status: Income as % of Federal Poverty Level (FPL) 0–200% FPL 43.0%; >200% FPL 57.0%
Exposures	Electronic cigarettes Use: ever use, past-30 days use<br>Details on EC devices: Not reported<br>Electronic cigarettes availability: Not applicable
Outcomes	Methods: “We used the data from the California Health Interview Survey (CHIS) 2017–2018 to compare the rates of using cigarettes, e-cigarettes, and marijuana separately or any use of the three. Using CHIS 2018 data, weighted logistic regression models were used to examine associations of using cigarettes, e-cigarettes, and marijuana separately or any use of these products/substance with demo-socioeconomic factors, psychological distress, and use of each product/substances.”<br>Adjustment for confounders: "models have adjusted for covariates, which include age, race/ethnicity, sex, FPL, urban/rural status, region of residence, and psychological distress"<br>Type of combustible tobacco use: cigarette<br>Combustible tobacco use: ever use, past-30 days use
Study funding	“Funding for this study was provided by the California Tobacco Control Program, California Department of Public Health. The funder had no role in the study design, collection, analysis, or interpretation of the data, writing the manuscript, or the decision to submit the paper for publication.”
Author declarations	“The authors declare that they have no known competing financial interests or personal relationships that could have appeared to influence the work reported in this paper.”

Miech R, Patrick ME, O'Malley PM, Johnston LD. E-cigarette use as a predictor of cigarette smoking: results from a 1-year follow-up of a national sample of 12th grade students. Tobacco Control 2017;26:e106-e111.

Study characteristics

Methods	Design: Longitudinal cohort (Individual level study)<br>Recruitment: via Monitoring the Future (MTF) (waves 2014 and 2015)<br>Setting: schools<br>Study start date/end date: 2014/2015<br>Number of datapoints: 2<br>Primary dataset: MTF<br>Country: USA
Participants	Total N: 347<br>Age: 12th graders<br>EC use at baseline: Past 30-days use 15.60%

	Gender/sex: Females 56.26%<br>Ethnicity/race: non-white 39.89%<br>Measures of socioeconomic status: Not reported
Exposures	Electronic cigarettes Use: Past-30 days use<br>Details on EC devices: Not reported<br>Electronic cigarettes availability: Not applicable
Outcomes	Methods: "Analysis of prospective longitudinal panel data from the nationally representative Monitoring the Future study. The analysis is based on 347 12th grade students who were part of a randomly selected subsample that completed in-school surveys in 2014 and were resurveyed 1-year later."<br>Adjustment for confounders: "The multivariable models include additional controls for demographics as well as baseline levels of marijuana use and binge drinking, which serve as measures of proclivity for general substance use."<br>Type of combustible tobacco use: cigarette<br>Combustible tobacco use: past 30 days, lifetime use<br>Risk ratio of reporting past-year cigarette smoking at follow up after vaping at baseline.
Study funding	"This study was supported by the National Institute on Drug Abuse, part of the National Institutes of Health, by grants numbers R01DA001411 and R01DA016575."
Author declarations	"None declared."

Morgenstern M, Nies A, Goecke M, Hanewinkel R. E-Cigarettes and the Use of Conventional Cigarettes. Dtsch Arztebl Int 2018;115(14):243-248

Study characteristics

Methods	Design: Longitudinal cohort (Individual level study)<br>Recruitment: Secondary analysis of cluster RCT of a school-based binge drinking prevention programme. Data from schools in the German states of Lower Saxony and Schleswig-Holstein.<br>Setting: schools<br>Study start date/end date: 2015/2016 school year/ 6 months after baseline<br>Number of datapoints: 2<br>Primary dataset: Secondary analysis of cluster RCT of a school-based binge drinking prevention programme. Data from schools in the German states of Lower Saxony and Schleswig-Holstein.<br>Country: Germany
Participants	Total N: 2186<br>Age: mean 15.49 (0.65) years<br>EC use at baseline: 313 (14.3 %)<br>Gender/sex: Females 53.6%

	Ethnicity/race: Not reported<br>Measures of socioeconomic status:<br>School type- not upper secondary school 48.8%<br>Parents % no secondary school certification 58.9%<br>SES (M, SD) R1-10 5.99 (1.52)
Exposures	Electronic cigarettes Use: ever use<br>Details on EC devices: Not reported<br>Electronic cigarettes availability: Not applicable
Outcomes	Methods: “During the 2015/2016 school year, 2186 tenth-graders in the German states of Lower Saxony and Schleswig-Holstein who had never smoked conventional cigarettes before took part in a survey over a 6-month period." Multiple regression was used to examine the association between e-cigarette consumption and the use of conventional cigarettes.”<br>Adjustment for confounders: “Statistical control for age, sex, state, immigrant background, type of school, socioeconomic status, various personality traits (sensation-seeking, impulsivity, anxiety, hopelessness, extraversion, agreeableness, conscientiousness, neuroticism, openness), and the use of alcohol, cannabis, and other illicit drugs.”<br>Type of combustible tobacco use: cigarette<br>Combustible tobacco use: ever use<br>Risk ratio of onset of cigarette after e-cigarettes use.
Study funding	"This study was funded by the Federal Center for Health Education on behalf of the Federal Ministry for Health."
Author declarations	"The authors declare no conflict of interest."

Nguyen HV, Bornstein S. Changes in adults' vaping and smoking behaviours associated with aerosol-free laws. Tobacco Control 2021;30(6):644-652.

Study characteristics

Methods	Design: Difference-in-differences (Population level study)<br>Recruitment: via Canadian Tobacco, Alcohol and Drugs Survey (CTADS) and the Canadian Tobacco Use Monitoring Survey (CTUMS).<br>Study start date/end date: 2013/ 2017<br>Number of datapoints: 4<br>Primary dataset: CTADS and CTUMS<br>Country: Canada
Participants	Total N: Adults aged 19 and older from CTADS 2013–2017 for e-cigarette outcomes (N=36 562) and from CTUMS/CTADS 2004–2017 for combustible cigarette outcomes (N=178 654).<br>E-Cigarette sample: Provinces with ban (N=24 605); Provinces without ban (N=11 957)<br>Combustible cigarette sample: Provinces with ban (N=122 282); Provinces without ban (N=56360)

	Age: 19+ mean (SD)<br>E-Cigarette sample: Provinces with ban 48.3(17.7) years; Provinces without ban 47.1 (17.5) years<br>Combustible cigarette sample: Provinces with ban 47.2 (1) years; Provinces without ban 46.2 (17.2) years<br>EC use at baseline: Pre- ban mean (age 19+): Ever e- cigarette use (N=36 562) 12.2%; Past 30- day e- cigarette use (N=36 555) 2.8%; E- cigarette use for cessation (n=2847) 49.5%; E- cigarette use when unable to smoke combustible cigarettes (n=3003) 35.6%; Everyday e- cigarette use (n=988) 33.7%<br>Gender/sex: Male 49%<br>Ethnicity/race: Not reported<br>Measures of socioeconomic status: Not reported
Exposures	Electronic cigarettes Use: ever use, past 30-day use<br>Details on EC devices: Not reported<br>Electronic cigarettes availability: Bans on e- cigarette use in public places and workplaces in Canadian provinces
Outcomes	Methods: "Adults aged 19 and older from CTADS 2013–2017 for e-cigarette outcomes (N=36 562) and from CTUMS/CTADS 2004–2017 for combustible cigarette outcomes (N=178 654). The DD analyses were implemented using regression models. In the DD regressions, the covariate of interest was an indicator variable for ban exposure, equal to 1 if the respondent was in one of the provinces with a ban and interviewed in the post- ban period, and 0 otherwise. We included control variables at both individual level (age, gender, marital status, household size and urban status) and provincial level (unemployment rates). "<br>Comparator: "The extension of the existing smoke- free laws to include e- cigarettes was part of broader policy efforts to address rising e- cigarette use. In addition to these provincial bans on e- cigarette use in public places and workplaces, there were concurrent provincial bans on sales of e- cigarettes to minors."<br>Type of combustible tobacco use: cigarettes<br>Combustible tobacco use: ever use, current use<br>Effects of ban in past 30 days cigarette use.
Study funding	"Dr Nguyen is supported by a Canada Research Chair in Health Policy Evaluation and a CIHR New Investigator Award."
Author declarations	"None declared."

Niaura R, Rich I, Johnson AL, Villanti AC, Romberg AR, Hair EC, et al . Young Adult Tobacco and E-cigarette Use Transitions: Examining Stability Using Multistate Modeling. Nicotine Tob Res 2020;22(5):647-654.

Study characteristics

Methods	Design: Longitudinal cohort (individual level study)<br>Recruitment: The sample was recruited via address-based sampling methods to provide a statistically valid representation of the US population, including cell phone-only households.<br>Setting: nationally representative sample of adults aged 18–34 years at study entry, online survey.<br>Study start date/end date: December 2011/July 2015

	Number of datapoints: 7<br>Primary dataset: Truth Initiative Young Adult Cohort Study<br>Country: USA
Participants	Total N: 8060 were eligible/available for the analysis.<br>Age: 18–24 (n = 3314), 25–34 (n= 4827). Mean age 25.97 (SD 4.87) years.<br>EC use at baseline: At the baseline wave (n = 3541), the prevalence of e-cigarette only use was 0.23%, and dual use was 1.57%<br>Gender/sex: Male (n = 3415) 41.9%, Female (n = 4726) 58%<br>Ethnicity/race: White (n = 4905) 60.2%<br>Black (n = 756) 9.3%<br>Other (n = 617) 7.6%<br>Hispanic (n = 1854) 22.8%<br>Measures of socioeconomic status:<br><High school (n = 766) 9.5%<br>High school (n = 1773) 21.8%<br>Some college (n = 3234) 39.7%<br>Bachelor or graduate degree (n = 2368) 29.1%
Exposures	Electronic cigarettes Use: ever use, past 30 days<br>Details on EC devices: not reported<br>Electronic cigarettes availability: not applicable
Outcomes	Methods: "A national sample of young adult tobacco product users and nonusers between the ages<br>of 18 and 34 years at baseline was surveyed at 6-month intervals for 3 years. Use and nonuse states were defined as mutually exclusive categories based on self-reported, past 30-day use of the various products. Never use, noncurrent use, and current use of combustible, noncombustible tobacco, and electronic cigarette (e-cigarette) products was assessed at each interval. A multistate model was fit to estimate transition probabilities between states and length of stay within each state."<br>Adjustment for confounders: "Participant age (in years) at baseline was included as a covariate in the analytic model."<br>Type of combustible tobacco use: cigarette, cigarillos, cigars, waterpipe, others<br>Combustible tobacco use: every use, past 30 days
Study funding	"This study was funded by Truth Initiative, but the views in this article do not necessarily represent those of Truth Initiative."
Author declarations	"Niaura receives funding from the Food and Drug Administration Center for Tobacco Products via contractual mechanisms with Westat and the National Institutes of Health. Within the past 3 years, he has served as a paid consultant to the Government of Canada via a contract with Industrial Economics Inc and has received an honorarium for a virtual meeting from Pfizer Inc. The other authors have no conflicts of interest to disclose."

Ortega A, Sutton M, McConville A, Cushing CC, Fite PJ . Longitudinal investigation of the bidirectional associations between initiation of e-cigarettes and other substances in adolescents. Journal of Substance Use 2021;26(1):40-47.

Study characteristics

Methods	Design: Longitudinal cohort (individual level study)<br>Recruitment: via the longitudinal study<br>Setting: 9th and 10th grade youth from a single high school<br>Study start date/end date: Fall 2016/Fall 2018<br>Number of datapoints: 3<br>Primary dataset: longitudinal project examining factors contributing to emotional, behavioral, and academic outcomes in a Midwestern high school.<br>Country: USA
Participants	Total N: 211 9th and 10th grade students enrolled. Of the 176 consented youth, 141 students were included in the current study.<br>Age: 13-16 years, mean 14.6 (SD 0.6) years<br>EC use at baseline: initiation: 11.9%<br>Gender/sex: 47.4% Male<br>Ethnicity/race: not reported<br>Measures of socioeconomic status: not reported
Exposures	Electronic cigarettes Use: ever use<br>Details on EC devices: not reported<br>Electronic cigarettes availability: not applicable
Outcomes	Methods: "The current study longitudinally evaluated the risk for initiation of e-cigarettes and examined associations with this risk and initiation of other substances (alcohol, traditional tobacco products, and marijuana). 141 youth from a single high school were in 9th and 10th grade at the initial data collection. Data were collected annually for the following two years. Rates of initiation were calculated. Cross-lagged panel models estimated how prior initiation of e-cigarette use affected the risk for initiation of other substances and vice-versa."<br>Adjustment for confounders: not reported<br>Type of combustible tobacco use: traditional tobacco products or cigarettes<br>Combustible tobacco use: ever use
Study funding	"This research did not receive any specific grant from funding agencies in the public, commercial, or not-for-profit sectors."
Author declarations	"The authors report no conflict of interest."

Osibogun O, Bursac Z, Maziak W. E-Cigarette Use and Regular Cigarette Smoking Among Youth: Population Assessment of Tobacco and Health Study (2013-2016). Am J Prev Med 2020;58(5):657-665.

Study characteristics

Methods	Design: Longitudinal cohort (individual level study)

	Recruitment: via PATH<br>Setting: Nationally representative dataset of youth in US<br>Study start date/end date: 2013/2016<br>Number of datapoints: 3<br>Primary dataset: PATH<br>Country: USA
Participants	Total N: Analyses were restricted to youth who had complete information on follow-up waves: 7,438 (for 1-year progression model) and 7,185 (for 2-year progression model).<br>Age: 12−14 years n=5,656; 15−17 years n= 1,782<br>EC use at baseline: e-cigarette use in the past 30 days (exclusive of never e-cigarette users) among cigarette nonsmokers (those who did not report cigarette smoking in the past 30 days) 221/7438<br>Gender/sex: Female 3,584, Male 3,854<br>Ethnicity/race:<br>White 3,512<br>African American 1,028<br>Hispanic 2,196<br>Other 702<br>Measures of socioeconomic status:<br>Parent's education level<br>High school or less 2,853<br>Some college 2,316<br>Bachelor's degree or higher 2,229
Exposures	Electronic cigarettes Use: past 30 days<br>Details on EC devices: not reported<br>Electronic cigarettes availability: not applicable
Outcomes	Methods: "examines the association between current e-cigarette use at baseline and regular cigarette smoking at follow-up among U.S. youth. A longitudinal analysis of youth (aged 12–17 years) data from Waves 1–3 of the Population Assessment of Tobacco and Health Study (2013–2016) was conducted between January 2019 and December 2019. Youth who reported past-30-day current e-cigarette use at baseline were identified and followed for regular cigarette smoking (≥20 days) at follow-up."<br>Adjustment for confounders: "Models adjusted for age, sex, race/ethnicity, parent's education level, other tobacco product use, any risk taking, living with a tobacco user, and noticed cigarette health warning label."<br>Type of combustible tobacco use: cigarette<br>Combustible tobacco use: past 30 day use, regular cigarette smoking at Wave 2 and Wave 3 (defined as reporting cigarette smoking on ≥20 days in the past 30 days)

Study funding	"supported by the NIDA T32DA043449 grant. ZB is supported by the FIU-Research Center in Minority Institution (grant U54MD012393-01). WM is supported by NIH (grants R01-DA035160, R01-TW010654, R01-DA042477) and the NIDAT32DA043449 grant. The content is solely the responsibility of the authors and does not necessarily represent the official views of NIH."
Author declarations	Not reported

Owotomo O, Stritzel H, McCabe SE, Boyd CJ, Maslowsky J. Smoking Intention and Progression From E-Cigarette Use to Cigarette Smoking. Pediatrics. 2020;146(6):e2020002881.

Study characteristics

Methods	Design: Longitudinal cohort (individual level study)<br>Recruitment: via PATH<br>Setting: Nationally representative sample<br>Study start date/end date: wave 2, 2014/wave 3, 2016<br>Number of datapoints: 2<br>Primary dataset: PATH<br>Country: USA
Participants	Total N: total final sample of 8661 youth; when analysing bivariate association of E-Cigarette use at Wave 2 and smoking initiation at Wave 3, by smoking intention at Wave 2, n = 8242.<br>Age: 12-14 years n=5484, 15-17 years n=3177<br>EC use at baseline: Ever using e-cigarettes at wave 2<br>Yes 701 (8.5%), No 7552 (91.5%)<br>Gender/sex: Male sex n=4452 (51.5%), Female sex n=4188 (48.5%)<br>Ethnicity/race:<br>White non-Hispanic n=4014 (46.9%)<br>Black non-Hispanic n=1187 (13.9%)<br>Other non-Hispanic n=795 (9.3%)<br>Hispanic n=2570 (30.0%)<br>Measures of socioeconomic status:<br>Parent’s education<br>Less than high school n=1323 (15.5%)<br>GED n=421 (5.0%)<br>High school graduate n=1604 (18.8%)<br>Some college n=2584 (30.4%)<br>Bachelor’s degree n=1683 (19.8%)<br>Advanced degree n=898 (10.6%)

Exposures	Electronic cigarettes Use: ever use<br>Details on EC devices: Electronic nicotine products, such as e-cigarettes, e-cigars, e-pipes, e-hookahs, personal vaporizers, vape pens and hookah pens<br>Electronic cigarettes availability: not applicable
Outcomes	Methods: "Multivariable logistic regression was used to analyze whether smoking intention moderated the association between e-cigarette use and ever smoking among adolescent never-smokers of conventional cigarettes. In model 1, smoking intention and ever using e-cigarettes at wave 2 predicted ever smoking at wave 3, with all covariates included except the interaction term (smoking intention X e-cigarette use). Model 2 added the interaction term to test whether the association between e-cigarette use and ever smoking is statistically different for adolescents with previous smoking intention versus those without. Generated odds ratios of ever smoking for each group on the basis of weighted marginal least square means coefficients."<br>Adjustment for confounders: "All models control for sex, age, race and ethnicity, parent education, subjective norms toward smoking, perceived behavioural control over smoking, other tobacco product use at wave 2, protobacco advertisement exposure, antitobacco advertisement exposure, peer smoking, perceived harm of conventional cigarette smoking, perceived addictiveness of conventional cigarette smoking, access to cigarettes in store, and parental monitoring."<br>Type of combustible tobacco use: cigarettes<br>Combustible tobacco use: ever use
Study funding	"Supported by grants from the Eunice Kennedy Shriver National Institute of Child Health and Human Development (K01HD091416 and P2CHD042849), National Cancer Institute (R01CA203809), National Institute on Drug Abuse (R01DA44157), and from the William T. Grant Foundation Scholars Program. The content is solely the responsibility of the authors and does not necessarily represent the official views of the funders. Funded by the National Institutes of Health (NIH)."
Author declarations	"The authors have indicated they have no potential conflicts of interest to disclose."

Patanavanich R, Worawattanakul M, Glantz S. Longitudinal bidirectional association between youth electronic cigarette use and tobacco cigarette smoking initiation in Thailand. Tob Control 2022057491. [DOI: 10.1136/tc-2022-057491]

Study characteristics

Methods	Design: logitudinal cohort (individual level study)<br>Recruitment: Paper based survey administered in schools.<br>Setting: a school- based nationally representative longitudinal survey of seventh grade students (average age 13 years) using a multistage random sampling scheme.<br>Study start date/end date: wave 1 in 2019 (baseline) and wave 2 in 2020 (follow- up). Follow-up in February 2020 before schools were closed due to COVID-19.<br>Number of datapoints: 2<br>Primary dataset: Thailand Parental Supply and Use of Alcohol, Cigarettes & Drugs Longitudinal Study Cohort in Secondary School Students.<br>Country: Thailand
Participants	Total N: 4389 (72.6%) completed the 12-month follow-up. 4116 students had never smoked at baseline.

	Age: mean 12.9 (SD 0.6) years (data for 4116 who had never smoked at baseline)<br>EC use at baseline: Of 4116 who had never smoked at baseline EC use at baseline: n (%) Never 3687 (97.3); Ever 93 (2.4); Current 41 (1.1)<br>Gender/sex: male n=1807 (43.9%), female n=2309 (56.1%) (data for 4116 who had never smoked at baseline).<br>Ethnicity/race: not reported<br>Measures of socioeconomic status:<br>Parental college education, n=2134 (51.9%) (data for 4116 who had never smoked at baseline)
Exposures	Electronic cigarettes Use: ever use, past 30 days<br>Details on EC devices: not reported<br>Electronic cigarettes availability: not applicable
Outcomes	Methods: "Data from a longitudinal survey of 6045 Thai seventh grade students with baseline in 2019 and the 12-month follow-up in 2020 were analysed using complex survey multivariate logistic regressions to assess whether e-cigarette use was associated with subsequent cigarette smoking (ever, current and dual product users at follow-up) among baseline never smokers."<br>Adjustment for confounders: "adjusted each model for baseline characteristics including age, sex, academic achievement, parental education, parental and peer smoking behaviour, peer approval of smoking, living with both parents, current alcohol use, prosocial behaviour and mental health status"<br>Type of combustible tobacco use: cigarette<br>Combustible tobacco use: ever use, past 30 days
Study funding	"This work was supported by the Faculty of Medicine Ramathibodi Hospital, Mahidol University, Thailand. The funding agencies played no role in study design; collection, analysis, and interpretation of data; writing the report; or the decision to submit for publication."
Author declarations	"SG serves as a consultant to the World Health Organization. The other authors declare that there are no competing interests."

Pearson JL, Sharma E, Rui N, Halenar MJ, Johnson AL, Cummings KM, et al . Association of Electronic Nicotine Delivery System Use With Cigarette Smoking Progression or Reduction Among Young Adults. JAMA Network Open 2020;3(11):e2015893.

Study characteristics

Methods	Design: Longitudinal cohort (Individual level study)<br>Recruitment: via Population Assessment of Tobacco and Health (PATH) Study (waves 2013-2014, 2014-2015, and 2015-2016)<br>Setting: survey-based<br>Study start date/end date: Wave 1 (2013-2014) and Wave 3 (2015 and 2016)<br>Number of datapoints: 3<br>Primary dataset: PATH<br>Country: USA
Participants	Total N: 1096<br>Age: 21.4 years

	EC use at baseline: Never ENDS users at wave 1<br>Gender/sex: Females 55.6%<br>Ethnicity/race: Hispanic 25.2%; white 63.7%<br>Measures of socioeconomic status: Educational level <High school degree 13.9%; GED 5.9%; High school degree 26.9%; Some college or associate degree 39.7%; ≥College 13.6%
Exposures	Electronic cigarettes Use: past- 30 days use<br>Details on EC devices: Not reported<br>Electronic cigarettes availability: Not applicable
Outcomes	Methods: "This cohort study used 3 waves of data (2013-2014, 2014-2015, and 2015-2016) from the Population Assessment of Tobacco and Health (PATH) Study, an ongoing longitudinal cohort study of adults and youth. Unweighted 1:6 propensity score matching was used to match participants on wave 1 risk factors for ENDS use at wave 2. The changes in smoking between wave 2 and wave 3 were assessed using the matched sample. In total, 1096 ENDS-naive, ever cigarette–smoking YAs (18-24 years of age) at wave 1 who participated in wave 2 and wave 3 and who had complete data in the PATH Study were included in the analyses, which were conducted from August 2018 to October 2019."<br>Adjustment for confounders: "To address the potential of confounding on the association between ENDS use and later cigarette smoking, we used propensity score matching (PSM) to create a sample matched on wave 1 risk factors for ENDS use at wave 2. Matching variables included age, race/ethnicity, educational level, alcohol use, binge alcohol consumption, marijuana use, other substance use, previous 30-day cigarette smoking (daily, nondaily, and no previous 30-day use), previous 30-day noncigarette combustible use, previous 30-day non-ENDS noncombustible use, previous 12-month quit attempt, intention to quit, tobacco advertising receptivity, e-cigarette harm perceptions, cigarette harm perceptions, nicotine dependence, and the Global Appraisal of Individual Needs–Short Screener substance use, internalizing and externalizing scales."<br>Type of combustible tobacco use: cigarette<br>Combustible tobacco use: past-30 days use<br>Association between wave 2 ENDS use and wave 3 changes in cigarette smoking.
Study funding	"This work was supported with funds from the NIDA, NIH, CTPs, FDA, Department of Health and Human Services, under a contract to Westat."
Author declarations	"Dr Pearson reported receiving personal fees fromWestat during the conduct of the study. Drs Pearson and Cummings reported receiving payment for expert testimony in lawsuits filed against the tobacco industry. Mr Halenar reported having a Population Assessment of Tobacco and Health (PATH) Study contract from the US Food and Drug Administration (FDA) Center for Tobacco Products (CTP) during the conduct of the study. Ms Johnson reported receiving grants and personal fees fromWestat and from the PATH Study during the conduct of the study. Dr Cummings reported receiving payment as a consultant to Pfizer for services on an external advisory panel to assess ways to improve smoking cessation delivery in health care settings. Dr Goniewicz reported receiving grants from the National Institutes of Health (NIH) and the FDA during the conduct of the study; receiving grants from Pfizer and receiving personal fees from Johnson and Johnson outside the submitted work; and being a member of the National Academies of Sciences, Engineering, and Medicine Committee on the Review of the Health Effects of Electronic Nicotine Delivery Systems. Dr Tanski reported receiving grants from the National Institute on Drug Abuse (NIDA) and from the National Cancer Institute during the conduct of the study. Dr Compton reported having long-term stock holdings in General Electric Co, 3MCompanies, and Pfizer outside the submitted work. Dr Abrams reported receiving grants from a Westat subcontract from the NIH-NIDA–FDA during the conduct of the study. Dr Hyland reported receiving a peer-reviewed contract from NIDA during the conduct of the study. Dr Stanton reported receiving a PATH Study contract from NIDA-CTP during the conduct of the study. No other disclosures were reported."

Penzes M, Foley KL, Nadasan V, Paulik E, Abram Z, Urban R. Bidirectional associations of e-cigarette, conventional cigarette and waterpipe experimentation among adolescents: A cross-lagged model. Addictive Behaviors 2018;80:59-64.

Study characteristics

Methods

Design: Longitudinal cohort (Individual level study)
Recruitment: via school-based, cluster randomized controlled trial
Setting: high schools
Study start date/end date: November 2014/May 2015
Number of datapoints: 2
Primary dataset: Original dataset - "secondary analysis of the data collected in a school-based, cluster randomized controlled trial designed to test a web-based multimedia program to prevent the initiation of smoking among adolescents."
Country: Romania

Participants

Total N: 1369
Age: mean 14.88 (SD 0.48)
EC use at baseline: 490 (35.8%)
Gender/sex: Measured but not reported
Ethnicity/race: Not reported
Measures of socioeconomic status: Not reported

Exposures

Electronic cigarettes Use: ever use
Details on EC devices: Not reported
Electronic cigarettes availability: Not applicable

Outcomes

Methods: "Longitudinal assessment of conventional cigarette, e-cigarette and waterpipe use initiation was conducted in a school-based cohort of 1,369 9th graders (mean age=14.88 SD=0.48 at baseline) during fall 2014 and reassessed 6-months later using online self-reported questionnaires. Autoregressive cross-lagged analysis within structural equation modeling framework was performed to simultaneously estimate the initiation of these products over a six-month period, controlling for age, gender, and participation in an intervention program to reduce conventional cigarette initiation."
Adjustment for confounders: "Intervention/control condition, gender, and age were included in the analyses in order to control for confounding variables."
Type of combustible tobacco use: cigarette
Combustible tobacco use: ever use
Odds of experimenting cigarette smoking after trying e-cigarettes at baseline.

Study funding

"This work was supported by the Fogarty International Center and National Cancer Institute of the National Institutes of Health under Grant Number 1R01TW009280. The content is solely the responsibility of the authors and does not necessarily represent the official views of the National Institutes of Health. Fogarty International Center and National Cancer Institute of the National Institutes of Health had no involvement in study design, collection, analysis, or interpretation of data, writing the manuscript, and the decision to submit the manuscript for publication."

Author declarations	"None."

Pesko MF, Hughes JM, Faisal FS. The influence of electronic cigarette age purchasing restrictions on adolescent tobacco and marijuana use. Prev Med 2016;87:207-212.

Study characteristics

Methods	Design: Natural experiment (population level study)<br>Recruitment: state-level aggregated data from the Youth Risk Behavior Surveillance System (YRBSS)<br>Setting: School surveys<br>Study start date/end date: 2007/2013<br>Number of datapoints: 4<br>Primary dataset: Youth Risk Behavior Surveillance System<br>Country: USA
Participants	Total N: Not reported<br>Age: students in grades 9–12<br>EC use at baseline: not reported<br>Gender/sex: not reported<br>Ethnicity/race: measured but not reported<br>Measures of socioeconomic status: not reported
Exposures	Electronic cigarettes Use: not applicable<br>Details on EC devices: not applicable<br>Electronic cigarettes availability: ENDS age purchasing restrictions.
Outcomes	Methods: "In the United States, many states have established minimum legal purchase ages for electronic nicotine delivery systems (ENDS) to ban adolescent purchases, but these policies may also affect other related substance use. We explore whether ENDS are substitutes or complements for cigarettes, cigars, smokeless tobacco, and marijuana among adolescents by using variation in state-level implementation of ENDS age purchasing restrictions. We linked data on ENDS age purchasing restrictions to state- and year-specific rates of adolescent tobacco and marijuana use in 2007–2013 from the Youth Risk Behavior Surveillance System. This data provides a nationally representative sample of adolescents who attend public and private schools. We performed a fixed effect regression analysis exploring the influence of ENDS age purchasing restrictions on outcomes of tobacco use and marijuana use, controlling for state and year fixed characteristics, age-race cohorts, cigarette excise taxes, and cigarette indoor use restrictions."<br>Type of combustible tobacco use: cigarettes, cigars, marijuana.<br>Combustible tobacco use: 1) recent cigarette use (at least 1 day over the past 30 days), 2) casual cigarette use (between 1 and 19 days over the past 30 days) 3) regular cigarette use (at least 20 days over the past 30 days), 4) and heavy cigarette use (every day over the past 30 days). Also use recent (past 30 days) cigar use, smokeless tobacco use, and marijuana use as outcome measures.
Study funding	Not reported

Author declarations	"The authors declare that there are no conflicts of interest"

Pesko MF, Currie JM. E-cigarette minimum legal sale age laws and traditional cigarette use among rural pregnant teenagers. J Health Econ. 2019;66:71-90

Study characteristics

Methods	Design: Natural experiment (population level study)<br>Recruitment: not applicable<br>Setting: revised birth records reporting prenatal smoking for underage teenagers<br>Study start date/end date: 2010 to 2016<br>Number of datapoints: not applicable<br>Primary dataset: revised administrative birth records with geocoded information provided by National Center for Health Statistics.<br>Country: USA
Participants	Total N: Total observations 1,313,612<br>Age: (SD)<br>ENDS MLSA not in place at any point during the pregnancy: 14 years or less 4% (19.7%), 15 years 12.2% (32.7%), 16 years 29.2% (45.5%), 17 years 54.6% (49.8%)<br>ENDS MLSA 3 months prior to conception and birth: 14 years or less 3.8% (19%), 15 years 11.5% (31.9%), 16 years 29.5% (45.6%), 17 years 55.2% (49.7%)<br>ENDS MLSA 3 months prior to conception: 14 years or less 3.6% (18.6%), 15 years 11.6% (32%), 16 years 29.2% (45.4%), 17 years 55.6% (49.7%)<br>EC use at baseline: Not reported<br>Gender/sex: All female<br>Ethnicity/race: (SD)<br>ENDS MLSA not in place at any point during the pregnancy: White non-Hispanic 33% (47%), Black non-Hispanic 21.1% (40.8%), Hispanic 42.5% (49.4%), Other non-Hispanic or Missing 3.4% (18.1%)<br>ENDS MLSA 3 months prior to conception and birth: White non-Hispanic 29% (45.4%), Black non-Hispanic 18.2% (38.6%), Hispanic 47.9% (50%), Other non-Hispanic or Missing 4.9% (21.6%)<br>ENDS MLSA 3 months prior to conception: White non-Hispanic 26.9% (44.3%), Black non-Hispanic 15.7% (36.4%), Hispanic 51.8% (50%), Other non-Hispanic or Missing 5.6% (23.1%)<br>Measures of socioeconomic status: Not reported
Exposures	Electronic cigarettes Use: Not applicable<br>Details on EC devices: Not applicable<br>Electronic cigarettes availability: ENDS minimum legal sale age laws in 32 states.
Outcomes	Methods: "Teenagers under 18 could legally purchase e-cigarettes until states passed minimum legal sale age laws. These laws may have curtailed teenagers' use of e-cigarettes for smoking cessation. We investigate the<br>effect of e-cigarette minimum legal sale age laws (ENDS MLSAs) on prenatal cigarette smoking and birth outcomes for underage rural teenagers using data on

	all births from 2010 to 2016 from 32 states. In our primary analysis, we exploit the impact of ENDS MLSAs on smoking during pregnancy using a panel data analysis."<br>Adjustment for confounders: "control for pregnancy fixed effects to remove individual-level heterogeneity and trimester-by-year-by-month fixed effects. Also control for time-varying tobacco control policies including cigarette taxes, smoking indoor use laws in private workplaces, restaurants, and bars, and the percent of the population covered by vaping indoor use laws. Report findings by area (urban/suburban and rural) and by race."<br>Type of combustible tobacco use: cigarettes<br>Combustible tobacco use: The smoking<br>dependent variable takes one of three forms: 1) any smoking during the period in question; 2) moderate or heavy smoking (average daily cigarettes smoked ≥ 5), and 3) heavy smoking (average daily cigarettes smoked ≥ 10).
Study funding	"Research reported in this publication was supported by the National Institute on Drug Abuse of the National Institutes of Health under Award Number R01DA045016 (PI: Michael Pesko), P30DA040500 (PI: Bruce Schackman), and R01DA039968 (PI: Dhaval Dave)."
Author declarations	Not reported

Pesko MF, Warman C. Re-exploring the early relationship between teenage cigarette and e-cigarette use using price and tax changes. Health Econ 2022;31(1):137-153

Study characteristics

Methods	Design: Natural experiment (population level study)<br>Recruitment: via NYTS<br>Setting: school-based surveys and state level data on pricing and taxation<br>Study start date/end date: 2011 to 2015<br>Number of datapoints: 5<br>Primary dataset: match price and tax variation to survey data on current use of e-cigarettes and cigarettes for over 94,000 students between grades 6 and 12 in the National Youth Tobacco Survey (NYTS) for years 2011-2015<br>Country: USA
Participants	Total N: 94,651 observations<br>Age: mean 14.6 (SD 2.05) years<br>EC use at baseline: 2011 ever tried 3%, past 30 days 1%<br>Gender/sex: Female 49.5%<br>Ethnicity/race: White 52.4%, Black 13.6%, Hispanic 20.8%, Other 9.6%, Missing 3.5%<br>Measures of socioeconomic status: (SD)<br>County poverty rate 15.6% (0.5%), County median household income 55,704 (14,244), County unemployment rate 7.391 (2.458)
Exposures	Electronic cigarettes Use: not applicable<br>Details on EC devices: not applicable<br>Electronic cigarettes availability: e-cigarette and cigarette price and tax changes.

Outcomes	Methods: "study the contemporaneous and intertemporal effects of e-cigarette and cigarette price and tax changes. We estimate a traditional demand equation for cigarettes and e-cigarettes using regression analysis. We evaluate four separate dependent variables: (1) any e-cigarette use over the past 30 days, (2) any cigarette use over the past 30 days, (3) days of e-cigarette use over the past 30 days (4) total cigarettes consumed over the past 30 days. We also estimate the effect on conditional e-cigarette use days and conditional number of cigarettes smoked."<br>Adjustment for confounders: "control for gender, age, ethnicity, urban/rural classification scheme, percent of the population living in poverty, median household income, unemployment rate, indoor smoking restrictions, indoor vaping restrictions, e-cigarette minimum legal sale age laws, and percent of the population covered by a Tobacco 21 law. At the state level, control for beer taxes, medical marijuana laws, medical decriminalization laws, and the minimum wage."<br>Type of combustible tobacco use: cigarettes<br>Combustible tobacco use: past 30 days
Study funding	"National Institute on Drug Abuse, Grant/Award Number: R01DA045016"
Author declarations	"Dr. Pesko reports current or recent funding from the National Institutes of Health, American Cancer Society, Agency for Healthcare Research and Quality, Virginia Foundation for Healthy Youth, and the University of Kentucky's Institute for the Study of Free Enterprise. Dr. Warman has no conflicts of interest."

Pesko MF. Effects of e-cigarette minimum legal sales ages on youth tobacco use in the United States. J Risk Uncertain 2023;66:261–277.

Study characteristics

Methods	Design: Natural experiment (population level study)<br>Recruitment: via National Youth Tobacco Survey<br>Setting: survey on middle and high school youth's tobacco use.<br>Study start date/end date: 2000/2017<br>Number of datapoints: 12 waves<br>Primary dataset: National Youth Tobacco Survey (NYTS)<br>Country: USA
Participants	Total N: 251.229 respondents 2000-2017, 125,820 respondents 2011-2017<br>Age: <18 years, mean 14.2 years<br>EC use at baseline: Between 2011 and 2017 14.2% reported ever having used an EC and 5.5% reported current use<br>Gender/sex: 2011-2017: Female 49.9%, 2000-2017: Female 50.2%<br>Ethnicity/race:<br>2011-2017: Non-Hispanic White 42%, Non-Hispanic Black 15.1%, Other/Multiple 12%<br>Hispanic 26.9%<br>2000-2017: Non-Hispanic White 44%, Non-Hispanic Black 16.3%, Other/Multiple 10.8%<br>Hispanic 25.9%<br>Measures of socioeconomic status:

	Poverty Rate: 2011-2017, 14.094 [2.902], 2000-2017, 13.436 [2.956]<br>Unemployment Rate: 2011-2017, 6.725 [1.975], 2000-2017, 6.254 [2.050]
Exposures	Electronic cigarettes Use: E-cigarette use during lifetime, current e-cigarette use (use in the past 30 days)<br>Details on EC devices: Not applicable<br>Electronic cigarettes availability: Minimal Legal Sale Age (MLSA) between 2010 and 2016
Outcomes	Methods: "In the United States, individual states established a minimum legal sale age (MLSA) for e-cigarettes between 2010 and 2016 when a federal MLSA came into place. These policies provide a natural experiment from which we can better understand the effect that e-cigarettes have on youth combustible tobacco use. This paper uses National Youth Tobacco Survey data to estimate the effect of the gradual roll-out of e-cigarette MLSAs in the United States on youth e-cigarette use, cigarette use, and cigar use (i.e., cigars, cigarillos, or little cigars)."<br>Adjustment for confounders: “controls for age, gender, ethnicity/race, "state-level policy and environment characteristics: cigarette taxes, e-cigarette taxes, cigar taxes, smoking and vaping restrictions, Tobacco-21 laws (state + local population-weighted), beer taxes, medical and recreational marijuana laws, minimum wage, poverty rate, and unemployment rate."<br>Type of combustible tobacco use: cigarettes, cigars, cigarillos, or little cigars<br>Combustible tobacco use: Current cigarette use (use in past 30 days), daily cigarette use, current cigar use (i.e., cigars, cigarillos, or little cigars), and daily cigar use.
Study funding	"Dr. Pesko was supported by R01DA045016 from the National Institute on Drug Abuse of the National Institutes of Health and by a grant from the Institute for the Study of Free Enterprise at the University of Kentucky."
Author declarations	"No conflicts of interest to report."

Pierce JP, Chen R, Leas EC, White MM, Kealey S, Stone MD, et al . Use of E-cigarettes and Other Tobacco Products and Progression to Daily Cigarette Smoking. Pediatrics 2021;147(2):e2020025122.

Study characteristics

Methods	Design: Longitudinal cohort (individual level study)<br>Recruitment: via PATH<br>Setting: Nationally representative sample<br>Study start date/end date: Wave 1 September 2013 to December 2014/ wave 4 complete early January 2018<br>Number of datapoints: 4<br>Primary dataset: PATH<br>Country: USA
Participants	Total N: 15 826<br>Age: 12-24 years

	12–14 n=5315<br>15–17 n=4771<br>18–21 n=3230<br>22–24 n=2510<br>EC use at baseline: Not reported<br>Gender/sex: Male n=7888, Female n=7938<br>Ethnicity/race:<br>Non-Hispanic white n=7637<br>Non-Hispanic Black n=2389<br>Hispanic n=4338<br>Asian n=438<br>Multiracial n=1024<br>Measures of socioeconomic status: Not reported
Exposures	Electronic cigarettes Use: ever use, past 30 days<br>Details on EC devices: not reported<br>Electronic cigarettes availability: not applicable
Outcomes	Methods: "To examine associations with progression to daily use of cigarettes at wave 4, we ascertained the subsample who were not daily users of tobacco at wave 1 and who did use at least 1 tobacco product by wave 3. Adjusted risk differences (aRDs) were computed from multivariable logistic regression."<br>Adjustment for confounders: The logistic regression was adjusted for age, sex, race and/or ethnicity, having smoke-free home at wave 1, exposure to other smokers at wave 1, and age at first use of any tobacco product.<br>Type of combustible tobacco use: cigarettes<br>Combustible tobacco use: Use: ever use, past 30 days, daily use.
Study funding	"Supported by the National Institutes of Health (grant 1R01CA234539) and by the Tobacco-Related Disease Research Program of the University of California, Office of the President (grant 28IR-0066). Funded by the National Institutes of Health (NIH)."
Author declarations	"The authors have indicated they have no potential conflicts of interest to disclose."

Pokhrel P, Kawamoto CT, Pagano I, Herzog TA. Trajectories of e-cigarette advertising exposure, e-cigarette use and cigarette smoking in a sample of young adults from Hawaii. Addiction 2022;117(7):2015-2026.

Study characteristics

Methods	Design: Longitudinal cohort (Individual level study)<br>Recruitment: via cohort conducted in students from two 4-year and four 2-year (community) colleges under the same university system on Oahu, Hawaii.<br>Setting: community colleges

	Study start date/end date: 2018/2020<br>Number of datapoints: 4<br>Primary dataset: original dataset<br>Country: USA
Participants	Total N: 2335<br>Age: 21.2 (2.2) years at baseline<br>EC use at baseline: Past 30-day e-cigarette use 0 days 77 % (1994), 1–2 days 8% (198), 3–5 days 3 % (76), 6–9 days 2% (52), 10–19 days 2% (63), 20–29 days 1 % (28), All 30 days 7% (179)<br>Gender/sex: Female 54% (1416), Male 46% (1206)<br>Ethnicity/race: White 24% (636), Asian 26% (681), Filipino 18 % (470), NHPI 21% (556), Other 11% (279)<br>Measures of socioeconomic status: Family/household income at baseline 0–39 999 $ 23% (603), 40 000–79 999 $ 34% (891), 80 000–119 999 $ 25% (656), 120 000–159 999 $ 10% (262), 160 000 or over $ 8% (210)
Exposures	Electronic cigarettes Use: past-30 days use<br>Details on EC devices: Not reported<br>Electronic cigarettes availability: Not applicable
Outcomes	Methods: “Longitudinal study using four waves of data were collected in 6-month intervals between 2018 and 2020. Unconditional growth models were estimated to address the study aims of determining the nature of the trajectories and between individual differences in growth factors. Conditional growth models were estimated to address the aims of testing the associations among growth trajectories and determining the demographic predictors of growth factors.”<br>Type of combustible tobacco use: cigarette<br>Combustible tobacco use: past-30 days use<br>Associations among trajectories of e-cigarette use and cigarette smoking.
Study funding	“The National Cancer Institute (US) (R01CA202277, R01CA228905).”
Author declarations	“None.”

Primack BA, Soneji S, Stoolmiller M, Fine MJ, Sargent JD . Progression to Traditional Cigarette Smoking After Electronic Cigarette Use Among US Adolescents and Young Adults. JAMA Pediatrics 2015;169(11):1018-1023.

Study characteristics	
Methods	Design: Longitudinal cohort (Individual level study)<br>Recruitment: via Dartmouth Media, Advertising, and Health Study (waves 2 and 3)- random digit dialling using landline (66.7%) and cellular telephone numbers (33.3%).<br>Setting: Community, telephone interviews

	Study start date/end date: 2012-2013/2013-2014<br>Number of datapoints: 2<br>Primary dataset: Dartmouth Media, Advertising, and Health Study<br>Country: USA
Participants	Total N: 694<br>Age: mean (SD) 19.5 (2.0) years EC use at baseline, 20.0 (2.4) years non-EC user<br>EC use at baseline: 16 (2.3 %)<br>Gender/sex: Female EC user at baseline 5 (31.3%), non-EC user 369 (54.4%)<br>Ethnicity/race:<br>Non-Hispanic white EC user 12 (75.0%), non-EC user 519 (76.5%)<br>Non-Hispanic black EC user 1 (6.3%), non-EC user 46 (6.8%)<br>Hispanic EC user 1 (6.3%), non-EC user 52 (7.7%)<br>Other EC user 2 (12.5%), non-EC user 61 (9.0%)<br>Measures of socioeconomic status: Maternal educational level mean (SD) for EC user: 7.5 (1.8); for non-EC user: 6.9 (2.5) [Scores ranged from 1 to 10, with higher scores representing more advanced<br>education.]
Exposures	Electronic cigarettes Use: ever use<br>Details on EC devices: Not reported<br>Electronic cigarettes availability: Not applicable
Outcomes	Methods: "In this longitudinal cohort study, a national US sample of 694 participants aged 16 to 26 years who were never cigarette smokers and were attitudinally nonsusceptible to smoking cigarettes completed baseline surveys from October 1, 2012, to May 1, 2014, regarding smoking in 2012-2013. They were reassessed 1 year later. Analysis was conducted from July 1, 2014, to March 1, 2015. Multinomial logistic regression was used to assess the independent association between baseline e-cigarette use and cigarette smoking, controlling for sex, age, race/ethnicity, maternal educational level, sensation-seeking tendency, parental cigarette smoking, and cigarette smoking among friends. Sensitivity analyses were performed, with varying approaches to missing data and recanting."<br>Type of combustible tobacco use: cigarette<br>Combustible tobacco use: ever use<br>Odds of using e-cigarettes at baseline and progressing to cigarette smoking.
Study funding	"This study was supported by grant R01-CA077026 for the survey from the National Cancer Institute (Dr Sargent), grants R01-CA140150 and R21-CA185767 from the National Cancer Institute (Dr Primack), and grant KL2-TR001088 from the National Center for Advancing Translational Sciences (Dr Soneji)."
Author declarations	"None reported."

Primack BA, Shensa A, Sidani JE, Hoffman BL, Soneji S, Sargent JD, et al . Initiation of Traditional Cigarette Smoking after Electronic Cigarette Use Among Tobacco-Naïve US Young Adults. Am J Med 2018;131(4):443.e1-443.e9.

Study characteristics

Methods	Design: Prospective cohort (individual level study)<br>Recruitment: this panel was populated using a combination of random digit dialling and address-based sampling, resulting in a sampling frame of an estimated 97% of US households. English-speaking adults aged 18 to 30 years were randomly selected to complete a baseline survey about tobacco use.<br>Setting: Nationally representative sample of never-smoking young adults ages 18–30.<br>Study start date/end date: baseline March 2013/follow-up October 2014<br>Number of datapoints: 2<br>Primary dataset: a nationally representative probability-based online nonvolunteer access panel recruited and maintained by Growth from Knowledge.<br>Country: USA
Participants	Total N: 915 (60.8% of 1506 at baseline) who completed follow-up.<br>Age: 18–20 21.8% , 21–23 32.7%, 24–26 24.2%, 27–30 21.4%<br>EC use at baseline: 16/915, 1.8%<br>Gender/sex: Female 61.6%, Male 38.4%<br>Ethnicity/race: White, non-Hispanic 64.8%, Black, non-Hispanic 10.9%, Hispanic 14.2%, Other 10.1%<br>Measures of socioeconomic status:<br>Yearly Household Income<br>Low (under $30,000) 25.0%<br>Medium ($30,000–74,999) 38.1%<br>High ($75,000 or more) 36.8%<br>Education Level<br>High school or less 28.0%<br>Some college 39.6%<br>Bachelor’s degree or higher 32.5%
Exposures	Electronic cigarettes Use: ever use<br>Details on EC devices: not reported<br>Electronic cigarettes availability: not applicable
Outcomes	Methods: "We conducted a prospective cohort study with assessments at baseline (March 2013) and follow-up (October 2014). We used sampling frames representing 97% of the U.S. population to recruit a nationally-representative sample of never-smoking young adults ages 18–30. The independent variable was baseline ever use of e-cigarettes. The main outcome measure was initiation of traditional cigarette smoking between baseline and 18-month follow-up. We compared the independent variable and all covariates among individuals who did and did not initiate smoking by 18-month follow-up. We calculated the statistical significance of these differences using Pearson’s $\chi 2$ tests. We then used bivariable and multivariable logistic regression to assess associations between baseline e-cigarette use and initiation of cigarette smoking. Primary multivariable analyses adjusted for all 10 measured covariates."<br>Type of combustible tobacco use: cigarette

	Combustible tobacco use: ever use
Study funding	"National Cancer Institute (R01-CA140150). Dr. Primack is supported by two grants from the National Cancer Institute (R01-CA140150 and R21-CA185767). Dr. Sargent is supported by the National Cancer Institute (R01-CA077026). Dr. Soneji is supported by the National Cancer Institute (R21-CA197912). The funding organizations had no role in the design and conduct of the study; collection, management, analysis, and interpretation of the data; or preparation, review, or approval of the manuscript."
Author declarations	"The authors have no conflicts of interest to report."

Romm KF, Childers MG, Douglas AE, Bray BC, Dino G, Blank MD. Transitions in tobacco use profiles among adolescents: Results from the Population Assessment of Tobacco and Health (PATH) study waves 3 and 4. Drug and Alcohol Dependence 2022;232:109272.

Study characteristics

Methods	Design: Longitudinal cohort (Individual level study)<br>Recruitment: via Population Assessment of Tobacco and Health Study (PATH)<br>Setting: Nationally representative sample<br>Study start date/end date: 2015-2016/2016-2018 (wave 3 and wave 4)<br>Number of datapoints: 2<br>Primary dataset: PATH<br>Country: USA
Participants	Total N: 1072<br>Age: 13.71 (SD 1.71) years<br>EC use at baseline: wave 3 36.6 %<br>Gender/sex: Female 46.4%, Male 53.6%<br>Ethnicity/race:<br>White 56.6%<br>Black 9.7%<br>Hispanic 23.2%<br>Other 10.5%<br>Measures of socioeconomic status: Parent Education mean 2.58 (SD 1.04) [Parent education is measured on a scale from 1 (less than high school) to 4 (college graduate)].
Exposures	Electronic cigarettes Use: ever use, past-30 days use<br>Details on EC devices: Not reported<br>Electronic cigarettes availability: Not applicable

Outcomes	Methods: "Data derived from the Population Assessment of Tobacco and Health (PATH) study. Participants used at least one tobacco product (cigarettes, electronic cigarettes [ECIGs], traditional cigars, cigarillos, filtered cigars, snus, smokeless tobacco [SLT], hookah) at Wave 3 (W3; 2015-2016) or 4 (W4; 2016-2018) and had Wave 1 (W1) data (N = 1072; M_{age}= 13.71, SD =1.71, 46.4% female; 56.6% White, 23.2% Hispanic). Latent transition analysis (LTA) examined probabilities of transitioning between classes across waves and sociodemographic correlates of transitions."<br>Type of combustible tobacco use: cigarette, cigarillos, cigars<br>Combustible tobacco use: past-30 days use<br>Associations among trajectories of e-cigarette use and cigarette smoking.
Study funding	"This publication was supported by the National Institute on Drug Abuse of the National Institutes of Health (NIH) and the Center for Tobacco Products of the U.S. Food and Drug Administration (FDA) (R21DA051628; PI: Blank). The content is solely the responsibility of the authors and does not necessarily represent the views of the NIH or FDA."
Author declarations	"The authors declare no conflicts of interests."

Saller FS, Agaku IT, Filippidis FT. Association between e-cigarette use initiated after cigarette smoking and smoking abstinence: a cross-sectional study among adolescent established smokers in the USA. Tobacco Control 2022;31(3):416-423.

Study characteristics

Methods	Design: Longitudinal cohort (Individual level study)<br>Recruitment: via National Youth Tobacco Survey (NYTS)<br>Setting: middle and high schools<br>Study start date/end date: 2015/2018<br>Number of datapoints: 4<br>Primary dataset: NYTS<br>Country: USA
Participants	Total N: 1697<br>Age: weighted % (95%CI)<br>9–13 years n=67 4.2% (2.9 to 6.0)<br>14–15 years n=269 17.0% (14.6 to 19.8)<br>16–17 years n=894 52.7% (49.4 to 55.9)<br>≥18 n=467 26.1% (23.4 to 29.0)<br>EC use at baseline: weighted % (95%CI)<br>Experimental use n=416 25.0% (22.4 to 27.9)<br>Prior established use n=260 15.8% (13.4 to 18.5)<br>Current established use n=756 44.6% (40.7 to 48.5)<br>Gender/sex: weighted % (95%CI)

	Male n=1018 57.7% (54.0 to 61.4)<br>Female n=666 42.3% (38.6 to 46.0)<br>Ethnicity/race: weighted % (95%CI)<br>Non-Hispanic white n=1024 68.2% (64.3 to 71.8)<br>Non-Hispanic black n=87 4.6% (2.9 to 7.3)<br>Hispanic n=352 17.6% (14.9 to 20.6)<br>Other/multiple races n=188 9.7% (7.7 to 12.1)<br>Measures of socioeconomic status: not reported
Exposures	Electronic cigarettes Use: ever use, past-30 days use<br>Details on EC devices: Not reported<br>Electronic cigarettes availability: Not applicable
Outcomes	Methods: "The data were drawn from the 2015– 2018 National Youth Tobacco Survey—a nationally representative survey of US middle and high school students. Multivariable logistic regression was used to assess the association between ever e-cigarette use and past 30-day abstinence from cigarette smoking. The analytical sample comprised ever established cigarette smokers with or without a history of e-cigarette use after smoking initiation."<br>Adjustment of confounders: "After adjusting for a key set of potential confounders (age, sex, race/ethnicity and survey year), models adjusting for additional potential confounders were explored using Akaike information criterion and Bayesian information criterion."<br>Type of combustible tobacco use: cigarette, cigarillos, cigars<br>Combustible tobacco use: past-30 days use<br>Odds of ever electronic use and smoking abstinence.
Study funding	"The authors have not declared a specific grant for this research from any funding agency in the public, commercial or not- for- profit sectors."
Author declarations	"None declared."

Schneller LM, Kasza KA, Hammond D, Bansal-Travers M, O'Connor R, Hyland A. E-cigarette and tobacco product use among NYS youth before and after a state-wide vaping flavour restriction policy, 2020-2021. Tob Control 2022;31(Suppl 3):s161-s166.

Study characteristics

Methods	Design: Repeat cross-sectional surveys, natural experiment (population level study)<br>Recruitment: via NYS<br>Setting: Cross-sectional surveys of NYS youth (16-19 years)<br>Study start date/end date: Wave 3.5 (February 2020)/Wave 5 (August 2021)<br>Number of datapoints: 4<br>Primary dataset: Cross-sectional New York State (NYS) data from the US International Tobacco Control Policy Evaluation Project Youth Tobacco and E-cigarette.

	Country: USA
Participants	Total N: Survey wave 3.5 n=955, 4 n=946, 4.5 n=1030 and 5 n=753. Total 3684<br>Age: 16-19 years<br>EC use at baseline: Use of e-cigarettes in the past 30 days at wave 3.5 (February 2020) was 20.4%<br>Gender/sex: Male 50.8%, Female 49.2%<br>Ethnicity/race: Non-Hispanic White 67.7%<br>Measures of socioeconomic status:<br>Not meeting basic expenses wave 3.5: 5.3%, wave 4: 2.4%, wave 4.5: 2.5%, wave 5: 2.6%<br>Just meeting basic expenses wave 3.5: 21.7%, wave 4: 22.4%, wave 4.5: 21.8%, wave 5: 21.8%<br>Meeting needs with a little left over wave 3.5: 32.5%, wave 4: 34.7%, wave 4.5: 29.6%, wave 5: 31.9%<br>Living comfortably wave 3.5: 36.7%, wave 4: 34.2%,wave 4.5: 39.6%, wave 5: 38.9%<br>Don't Know/Refused wave 3.5: 3.8%, wave 4: 6.3%, wave 4.5: 6.5%, wave 5: 4.9%
Exposures	Electronic cigarettes Use: past 30 days at each time point. E-cigarette flavour(s) used most often in the past 30 days was assessed at each time point. Daily vapers were those who vaped in 30 days of the past 30 days. Frequent vapers were those who vaped 20+ days and infrequent users were those who vaped fewer than 20 days.<br>Details on EC devices: not reported<br>Electronic cigarettes availability: state- wide restriction on flavoured e-cigarettes in New York State
Outcomes	Methods: "Reducing youth e-cigarette use is a New York State (NYS) public health priority. In May 2020, a state- wide restriction on flavoured e-cigarettes, except tobacco flavour, was passed. This study examines changes in nicotine product use behaviour among youth around the time of the state-wide vaping flavour restriction. NYS data from the US International Tobacco Control Policy Evaluation Project Youth Tobacco and E-cigarette Tobacco and Vaping Survey were analysed cross-sectionally from February 2020 (n=955), August 2020 (n=946), February 2021 (n=1030) and August 2021 (n=753). Online surveys were conducted among youth 16–19 years. Weighted descriptive statistics and regression models were used to describe changes in nicotine product use behaviour. Models were adjusted for age, sex, race/ethnicity and perceived family socioeconomic status."<br>Type of combustible tobacco use: cigarettes<br>Combustible tobacco use: past 30 days
Study funding	"This work was supported by the Center for Research on Flavored Tobacco (CRoFT), a Tobacco Center of Regulatory Science (TCORS), funded by the US FDA and National Cancer Institute (U54CA238110), as well as the ITC Youth Tobacco and Vaping Survey (P01CA200512). The content is solely the responsibility of the authors and does not necessarily represent the official views of the National Institutes of Health or the US FDA."
Author declarations	"The authors declare that they do not have competing<br>financial interests or personal relationships that could have influenced the work reported in this presentation. DH has served as an expert witness on behalf of governments in litigation involving the tobacco industry."

Selya AS, Dierker L, Rose JS, Hedeker D, Mermelstein RJ. The Role of Nicotine Dependence in E-Cigarettes' Potential for Smoking Reduction. Nicotine & Tobacco Research 2018;20(10):1272-1277.

Study characteristics

Methods	Design: Longitudinal cohort (Individual level study)<br>Recruitment: via Social and Emotional Contexts of Adolescent Smoking Patterns (SECASP) Study<br>Setting: high schools<br>Study start date/end date: 8th year of the cohort, 9th year of the cohort (unclear)<br>Number of datapoints: 4<br>Primary dataset: Social and Emotional Contexts of Adolescent Smoking Patterns (SECASP) Study<br>Country: USA
Participants	Total N: 586<br>Age: 24.3 (SD 0.8) years (at 8 years follow up)<br>EC use at baseline: Lifetime e-cigarette use 217 (37.0%); Past-month e-cigarette frequency ever users 0.7 (2.0 %)<br>Gender/sex: Female 340 (58.0%); Male 243 (42.0%)<br>Ethnicity/race: White 445 (75.9%); Nonwhite 141 (24.1%)<br>Measures of socioeconomic status: Not reported
Exposures	Electronic cigarettes Use: ever use, past-30 days use<br>Details on EC devices: Not reported<br>Electronic cigarettes availability: Not applicable
Outcomes	Methods: "The authors examined whether e-cigarette use is associated with cigarette smoking and whether this association differs across tobacco users with varying levels of nicotine dependence. The authors used VCM's to examine the nicotine-dependence-varying relationship between (1) lifetime and (2) recent e-cigarette use and past-month smoking frequency. A nicotine dependence varying intercept was also included to account for differences in mean smoking frequency across the range of nicotine dependence. All time points were pooled in this analysis both to increase sample size and because we did not anticipate large differences in the effect from year to year. Multilevel modeling was used to account for repeated observations."<br>Adjustment for confounders: "Control variables included age, sex, white race/ethnicity, and past week smoking quantity."<br>Type of combustible tobacco use: cigarette<br>Combustible tobacco use: past 30 days use; past week use<br>Association between e-cigarette use and frequency of cigarette smoking.
Study funding	"This research was funded by Project Grant P01 CA098262 from the National Cancer Institute and L40 DA042431 from the National Institute on Drug Abuse. The content is solely the responsibility of the authors and does not necessarily represent the official views of the NIH, NCI, or NIDA."
Author declarations	"None declared."

Selya AS, Rose JS, Dierker L, Hedeker D, Mermelstein RJ. Evaluating the mutual pathways among electronic cigarette use, conventional smoking and nicotine dependence. Addiction 2018;113(2):325-333.

Study characteristics

Methods	Design: Longitudinal cohort (Individual level study)<br>Recruitment: via Social and Emotional Contexts of Adolescent Smoking Patterns (SECASP) Study<br>Setting: high schools<br>Study start date/end date: 8th year of the cohort, 9th year of the cohort (unclear)<br>Number of datapoints: 4<br>Primary dataset: Social and Emotional Contexts of Adolescent Smoking Patterns (SECASP) Study<br>Country: USA
Participants	Total N: 1007<br>Age: 23.6 (SD 0.6) years (at 8 years follow up)<br>EC use at baseline: Any e-cig use in past month 63 (6.8%)<br>Gender/sex: Female 621 (61.7%); Male 386 (38.3%)<br>Ethnicity/race: White 744 (73.9%); Non-white 263 (26.1%)<br>Measures of socioeconomic status: Not reported
Exposures	Electronic cigarettes Use: past-30 days use<br>Details on EC devices: Not reported<br>Electronic cigarettes availability: Not applicable
Outcomes	Methods: “Data from four annual waves of a prospective cohort study were analyzed. Path analysis modeled the bidirectional, longitudinal relationships between past-month smoking frequency, past-month e-cigarette frequency and nicotine dependence.”<br>Adjustment for confounders: Not reported<br>Type of combustible tobacco use: cigarette<br>Combustible tobacco use: past 30 days use<br>Association between e-cigarette use and later cigarette smoking.
Study funding	“This research was funded by Project Grant P01 CA098262 from the National Cancer Institute, Project L40 DA042431 from the National Institutes on Drug Abuse, and by Center Grant P50 DA038938 awarded to Penn State University. The content is solely the responsibility of the authors and does not necessarily represent the official views of the NIH, NIDA or NCI.”
Author declarations	"None."

Shahab L, Beard E, Brown J . Association of initial e-cigarette and other tobacco product use with subsequent cigarette smoking in adolescents: a cross-sectional, matched control study. Tobacco Control 2021;30:212-220.

Study characteristics

Methods	Design: cross-sectional, matched control study (population level study)<br>Recruitment: via NYTS<br>Setting: middle and high school surveys<br>Study start date/end date: 2014-2015/2017<br>Number of datapoints: 4<br>Primary dataset: National Youth Tobacco Survey (NYTS)<br>Country: USA
Participants	Total N: 78,625 in 2014-2017, 39,718 in 2014-2015, 38,630 in 2014-2015 with complete information on first product used.<br>Age: 2014 (N=22 007) %(95%CI)<br>≤12years 18.7 (16.4 to 21.2)<br>13 years 15.3 (12.9 to 18.0)<br>14 years 14.7 (13.5 to 16.0)<br>15 years 14.9 (13.4 to 16.5)<br>16 years 14.1 (12.6 to 15.8)<br>17+ years 22.3 (19.9 to 25.0)<br>EC use at baseline: 2014 (N=22 007) %(95%CI)<br>ever use e-cigarettes 19.9 (18.1 to 21.8), past 30 day use e-cigarettes 9.3 (8.0 to 10.8), first tried 5.6 (4.9 to 6.4)<br>Gender/sex: 2014 (N=22 007) %(95%CI)<br>Female 49.8 (48.0 to 51.5), Male 50.2 (48.5 to 52.0)<br>Ethnicity/race: 2014 (N=22 007) %(95%CI)<br>Non-Hispanic white 58.3 (52.5 to 63.8)<br>Non-Hispanic black 15.3 (12.0 to 19.4)<br>Hispanic 21.9 (18.4 to 25.8)<br>Non-Hispanic other 4.5 (3.5 to 5.8)<br>Measures of socioeconomic status: not reported
Exposures	Electronic cigarettes Use: ever use, past 30 days, first tried<br>Details on EC devices: not reported<br>Electronic cigarettes availability: not applicable
Outcomes	Methods: "This study assessed whether initiating e-cigarette use increases the uptake of cigarette smoking in US adolescents compared with behavioural and synthetic controls. Data come from 78 265 adolescents in the National Youth Tobacco Survey (2014–2017) of whom 38 630 provided information about the first tobacco product they had used in 2014/15. Ever, past 30 day and established (30 day use and 100+ lifetime cigarettes) cigarette smoking was compared in adolescents who first used an e-cigarette (exposure group), a non-cigarette combustible (CT) or other non-combustible tobacco (NT) product (behavioural controls), and propensity score matched adolescents without initial e-cigarette use (synthetic controls)."<br>Adjustment for confounders: The following potential confounding variables were assessed across all four waves: Age, Sex, Ethnicity, Grade, School type, Future smoking susceptibility, Environmental exposure to tobacco, Perceived health effects of smoking.<br>Type of combustible tobacco use: cigarettes

	Combustible tobacco use: ever use, past 30 days, established use
Study funding	"This project is funded by Cancer Research UK (C1417/A22962). All authors are members of the UK Centre for Tobacco and Alcohol Studies (UKCTAS), funded under the auspices of the UK Clinical Research Collaboration (MR/K023195/1)."
Author declarations	"LS has received a research grant and honoraria for a talk<br>and travel expenses from manufacturers of smoking cessation medications (Pfizer and Johnson & Johnson). JB has received unrestricted research funding from Pfizer to study smoking cessation."

Snow E, Johnson T, Ossip DJ, Williams GC, Ververs D, Rahman I, et al. Does E-cigarette Use at Baseline Influence Smoking Cessation Rates among 2-Year College Students? Journal of Smoking Cessation 2018;13(2):110-120.

Study characteristics

Methods	Design: Longitudinal cohort (Individual level study)<br>Recruitment: via three-phase RCT (parent study) comparing the effectiveness of enhanced web-assisted tobacco intervention (E-WATI) to basic web-assisted tobacco intervention (B-WATI) as tools for smoking cessation in the 2-year college student population<br>Setting: college<br>Study start date/end date: unclear, 12 months after baseline<br>Number of datapoints: 4<br>Primary dataset: secondary analysis of data from ongoing three-phase RCT.<br>Country: USA
Participants	Total N: 1400<br>Age: 18-24 years: e-Cigarette users 332 (54%); e-Cigarettes non users 355 (45%); 25 years or more: e-Cigarette users 285 (46%); e-Cigarette non users 428 (54%)<br>EC use at baseline: e-Cigarette users (n= 617); e-Cigarette non-users (n= 783)<br>Gender/sex: e-Cigarette users Male 218 (35%), Female 386 (63%); e-Cigarette non-users Male 254 (31%); Female 517 (66%)<br>Ethnicity/race: e-Cigarette users White 494 (80%), Non-White 114 (18%); Non-Hispanic 556 (90%), Hispanic 53 (9%); e-Cigarette non-users White 598 (76%), Non-White 172 (22%); Non-Hispanic 687 (88%), Hispanic 53 (9%) 86 (11%)<br>Measures of socioeconomic status: Not reported
Exposures	Electronic cigarettes Use: Past-30 days use<br>Details on EC devices: Not reported<br>Electronic cigarettes availability: Not applicable
Outcomes	Methods: "Participants were 1,400 students from over 60 2-year colleges across 25 states who were current smokers enrolled in a web-assisted tobacco intervention (WATI) trial. Survey data at baseline, 1-, and 6-months, were evaluated. A series of multivariate logistic regression analyses were conducted entering variables identified in bivariate analyses and screened for multicollinearity."

	Adjustment for confounders: “A priori variables were identified prior to bivariate analysis and were included in the final logistic regression model regardless of significance. These variables included age, gender, race/ethnicity and intervention arm.”<br>Type of combustible tobacco use: cigarette, cigars, cigarillos<br>Combustible tobacco use: past 30 days use<br>Odds ratio of smoking cessation after e-cigarette use at baseline.
Study funding	“This work was supported by the National Cancer Institute (R01CA152093-01). Its contents are solely the responsibility of the authors and do not necessarily represent the official views of the National Cancer Institute or the National Institutes of Health.”
Author declarations	“The authors have no conflicts of interest and have nothing to declare.”

Spindle TR, Hiler MM, Cooke ME, Eissenberg T, Kendler KS, Dick DM . Electronic cigarette use and uptake of cigarette smoking: A longitudinal examination of U.S. college students. Addictive Behaviors 2017;67:66-72.

Study characteristics

Methods	Design: Longitudinal cohort (Individual level study)<br>Recruitment: via the Spit for Science (S4S) project, a university-wide longitudinal study aimed at assessing genetic and environmental influences on substance use and emotional health in college students<br>Setting: universities<br>Study start date/end date: 2014/2015<br>Number of datapoints: 2<br>Primary dataset: subset of Spit for Science (S4S) project<br>Country: USA
Participants	Total N: 3757<br>Age: 18.5 (SD 0.43) years<br>EC use at baseline: 153/3657<br>Gender/sex: Female 62%<br>Ethnicity/race: White: 47%; Black: 19%; Asian: 17%; Hispanic/Latino: 6%, mixed race/ethnicity: 7%. 4% of participants reported being either: American Indian/Alaskan Native, Native Hawaiian/Other Pacific Islander, unknown race or ethnicity, or chose not to answer the question.<br>Measures of socioeconomic status: Not reported
Exposures	Electronic cigarettes Use: ever use, past-30 days use<br>Details on EC devices: Not reported<br>Electronic cigarettes availability: Not applicable
Outcomes	Methods: “3757 participants from a Mid-Atlantic university were surveyed in 2014 and again in 2015. The first set of these cross classification analyses compared time 1 and time 2 e-cigarette/cigarette ever-use groups (i.e., never users, ever users of e-cigarettes only, ever users of cigarettes only, and ever

	users of e-cigarettes and cigarettes). The second set of analyses compared time 1 e-cigarette/cigarette ever-use groups with time 2 e-cigarette/cigarette current use groups (i.e., current exclusive e-cigarette users, current exclusive cigarette smokers, and current dual users of e-cigarettes and cigarettes). In the second cross classification analysis, the time 2 ever use categories were also included, as this provided more accurate percentages of individuals who transitioned into the various e-cigarette/cigarette current use groups. For all logistic regression analyses, ever use and past 30-day use items were changed from the original formatting to a dichotomous."<br>Adjustment for confounders: "Demographic characteristics, the use of other nicotine-containing products, and other variables previously shown to independently predict the uptake of cigarette smoking (e.g., impulsivity) were included as covariates."<br>Type of combustible tobacco use: cigarette<br>Combustible tobacco use: ever use, past 30 days use<br>Odds ratio of Time 1 e-cigarette use predicting time 2 cigarette use status
Study funding	"Spit for Science: The VCU Student Survey has been supported by Virginia Commonwealth University, P20AA107828, R37AA011408, K02AA018755, and P50 AA022537 from the National Institute on Alcohol Abuse and Alcoholism (NIAAA), and UL1RR031990 from the National Center for Research Resources (NCRR) and National Institutes ofHealth Roadmap for Medical Research. Research reported in this publication was also supported by the National Institute on Drug Abuse (NIDA) of the National Institutes of Health under Award Numbers P50DA036105 and F31DA040319 and the Center for Tobacco Products ofthe U.S. Food and Drug Administration (FDA). The content is solely the responsibility of the authors and does not necessarily represent the official views of the National Institutes ofHealth or the Food and Drug Administration."
Author declarations	"The authors have no conflicts of interest to declare."

Staff J, Kelly BC, Maggs JL, Vuolo M. Adolescent electronic cigarette use and tobacco smoking in the Millennium Cohort Study. Addiction 2022;117(2):484-494.

Study characteristics

Methods	Design: Prospective cohort (individual level study)<br>Recruitment: via MCS<br>Setting: Nationally representative sample of infants born September 2000 to January, 2002 in the UK<br>Study start date/end date: modal age 11 years (January 2012 to February 2013)/modal age 17 years (January 2018–March 2019)<br>Number of datapoints: 3, modal ages 11, 14 and 17 years.<br>Primary dataset: Millennium Cohort Study (MCS)<br>Country: UK
Participants	Total N: 10 625 youth who completed the 2018–19 interview (modal age 17)<br>Age: not reported at baseline<br>EC use at baseline: Ever used by age 14 17%<br>Gender/sex: Male 51%<br>Ethnicity/race:

	White British 87%<br>Other British 4%<br>Asian British 6%<br>Black British 3%<br>Measures of socioeconomic status:<br>Parent(s) educational attainment 3.11 (SEM 0.04)<br>Parents(s) occupational attainment 42%
Exposures	Electronic cigarettes Use: Ever use, Frequent use (i.e., 7 or more times weekly)<br>Details on EC devices: Not reported<br>Electronic cigarettes availability: Not applicable
Outcomes	Methods: " examined associations between e-cigarette<br>use and tobacco cigarette smoking at modal ages 14 and 17 years, controlling for adolescent and infancy risk factors. Intergenerational, prospective cohort data from the Millennium Cohort Study (MCS). Nationally representative sample of infants born September 2000 to January, 2002 in the United Kingdom. Parent and child<br>data from 10 625 youth assessed in infancy and modal ages 11, 14 and 17 years. Used logistic regression and regressions following coarsened exact matching."<br>Adjustment for confounders: "potential confounders were age 11 risk factors (e.g. alcohol use, externalizing behaviors, parental tobacco use, permissiveness), infancy risk factors (e.g. maternal smoking during pregnancy, smoke exposure in infancy) and demographic characteristics."<br>Type of combustible tobacco use: cigarettes<br>Combustible tobacco use: ever use, frequent<br>use (i.e., 7 or more cigarettes weekly)
Study funding	"This research is based on analyses of data from the UK Millennium Cohort Study (MCS), which receives core funding from the Economic and Social Research Council UK (ESRC) and a consortium of UK government departments. The study sponsors played no role in the study design; the collection, analysis and interpretation of data; the writing of the report; or the decision to submit the manuscript for publication."
Author declarations	"The authors have no conflicts of interest relevant to this<br>article to disclose."

Stanton CA, Bansal-Travers M, Johnson AL, Sharma E, Katz L, Ambrose BK, et al . Longitudinal e-Cigarette and Cigarette Use Among US Youth in the PATH Study (2013-2015). J Natl Cancer Inst 2019;111(10):1088-1096.

Study characteristics

Methods	Design: Longitudinal cohort (individual level study)<br>Recruitment: via PATH<br>Setting: Nationally representative sample<br>Study start date/end date: wave 1 2013/2014, wave 2 2014/2015<br>Number of datapoints: 2

	Primary dataset: PATH<br>Country: USA
Participants	Total N: 11 996 youth with data available at both waves.<br>Age: 12-17 years<br>EC use at baseline: ever-ENDS use wave 1 10.7% (n=1451, 95% CI 10.0 to 11.4), Past-30-day ENDS use wave 1 3.1% (n=418, 3.1%, 95% CI 2.8 to 3.5)<br>Gender/sex: measured but not reported<br>Ethnicity/race: measured but not reported<br>Measures of socioeconomic status: allowance, parental education, measured but not reported
Exposures	Electronic cigarettes Use: ever use, past 30 days<br>Details on EC devices: not reported<br>Electronic cigarettes availability: not applicable
Outcomes	Methods: "This analysis examines the bidirectional patterns of ENDS and cigarette use among US youth over one year and uses propensity score matching (PSM) to examine frequency of ENDS use on changes in cigarette smoking. Our analysis included 11 996 participants who had two waves of available data (Wave 1 [W1] 2013–2014; Wave 2 [W2] 2014–2015) drawn from the longitudinal Population Assessment of Tobacco and Health Study. Cross-sectional weighted prevalence estimates are reported for cigarettes and ENDS. We used PSM to estimate the likelihood of ENDS use at W1 and to draw matched analytic samples, then used regression (logistic or linear) models to examine the effect of W1 ENDS use on W2 cigarette smoking."<br>Adjustment for confounders: "The following variables were included in the construction of the propensity scores for the primary models: sex, race, ethnicity, education, allowance, live with a smoker, parental education, house tobacco use rules, house access to tobacco products, family structure."<br>Type of combustible tobacco use: cigarettes<br>Combustible tobacco use: ever use, past 30 days
Study funding	"This work was supported by federal funds from the National Institute on Drug Abuse, National Institutes of Health; and the Center for Tobacco Products, Food and Drug Administration, Department of Health and Human Services, under a contract to Westat (contract no. HHSN271201100027C)."
Author declarations	"K. Michael Cummings has received grant funding from the Pfizer, Inc. to study the impact of a hospital-based tobacco cessation intervention. Dr Cummings also receives funding as an expert witness in litigation filed against the tobacco industry. Maciej Goniewicz receives fees for serving on an advisory board from Johnson & Johnson and grant support from Pfizer. Wilson Compton reports holding stock in General Electric, 3M Companies, and Pfizer. No financial disclosures were reported by the other authors of this paper."

Stanton CA, Sharma E, Seaman EL, Kasza KA, Edwards KC, Halenar MJ, et al. Initiation of any tobacco and five tobacco products across 3 years among youth, young adults and adults in the USA: findings from the PATH Study Waves 1-3 (2013-2016). Tob Control 2020;29(Suppl 3):s178-s190

Study characteristics

Methods	Design: Longitudinal cohort (individual level study)<br>Recruitment: via PATH<br>Setting: nationally representative sample

	Study start date/end date: 2013/2016<br>Number of datapoints: 3<br>Primary dataset: PATH<br>Country: USA
Participants	Total N: wave 1 youth 11046, young adults 6478; wave 2 youth 9332, young adults 8052; wave 3 youth 7595, young adults 8052.<br>Age: 12-17 (youth), 18-24 (young adult)<br>EC use at baseline: youth 10.7% (89.3% never ENDS wave 1), young adult 32.1% (67.9% never ENDS wave 1)<br>Gender/sex: not reported<br>Ethnicity/race: not reported<br>Measures of socioeconomic status: not reported
Exposures	Electronic cigarettes Use: past 30 days, past 12 months<br>Details on EC devices: e-cigarettes, e- cigars, e- pipes and e-hookah<br>Electronic cigarettes availability: not applicable
Outcomes	Methods: "This study reports weighted cross-sectional prevalence of never use of tobacco, and longitudinal past 12-month (P12M), past 30- day (P30D) and frequent P30D any tobacco or specific tobacco product initiation across three 1-year waves. Longitudinal three-wave pathways are examined to outline pathways of exclusive and polytobacco initiation, as well as pathways of new initiators of electronic nicotine delivery systems (ENDS) or cigarettes. Data were drawn from the first three waves (2013–2016) of the Population Assessment of Tobacco and Health Study."<br>Adjustment for confounders: not reported<br>Type of combustible tobacco use: cigarettes, cigars, cigarillos, filtered cigars, pipe tobacco<br>Combustible tobacco use: past 30 days, past 12 months
Study funding	"This manuscript is supported with Federal funds from the National Institute on Drug Abuse, National Institutes of Health, and the Center for Tobacco Products, Food and Drug Administration, Department of Health and Human Services, under a contract to Westat (Contract No. HHSN271201100027C)."
Author declarations	"WMC reports long- term stock holdings in General Electric Company, 3M Company, and Pfizer Incorporated, unrelated to this manuscript. No financial disclosures were reported by the other authors of this paper."

Stanton CA, Tang Z, Sharma E, Seaman E, Gardner LD, Silveira ML, et al . Predictors of E-cigarette and Cigarette Use Trajectory Classes from Early Adolescence to Emerging Adulthood Across Four Years (2013-2017) of the PATH Study. Nicotine Tob Res 2023;25(3):421-429.

Study characteristics

Methods	Design: Longitudinal cohort (individual level study)<br>Recruitment: via PATH<br>Setting: Nationally representative sample<br>Study start date/end date: 2013/2017<br>Number of datapoints: 4<br>Primary dataset: PATH<br>Country: USA
Participants	Total N: 10086<br>Age: 12-17 years<br>EC use at baseline: unclear<br>Gender/sex: measured but not reported<br>Ethnicity/race: measured but not reported<br>Measures of socioeconomic status: parental education measured but not reported
Exposures	Electronic cigarettes Use: (1) never use, (2) ever use (excluding past 12-month (P12M) use), (3) P12M use (excluding P30D use), (4) P30D low frequency use (1–5 days), and (5) P30D high frequency use (6+ days).<br>Details on EC devices: e- cigarettes, e- cigars, e- pipes and e- hookah<br>Electronic cigarettes availability: not applicable
Outcomes	Methods: "This study examines predictors of trajectories of cigarette and e-cigarette use among a cohort of US adolescents transitioning into young adulthood. Comparing trajectories of each tobacco product is important to determine if different intervention targets are needed to prevent progression to daily use. Latent trajectory class analyses identified cigarette and e-cigarette use (never, ever excluding past 12-month, past 12-month (excluding past 30-day (P30D)), P30D 1–5 days, P30D 6+ days) trajectory classes, separately, among US youth (12–17; N = 10,086) using the first 4 waves (2013–2017) of data from the nationally representative PATH Study. Weighted descriptive analyses described the class characteristics. Weighted multinomial logistic regression analyses examined demographic, psychosocial, and behavioral predictors of class membership."<br>Adjustment for confounders: not reported<br>Type of combustible tobacco use: cigarettes<br>Combustible tobacco use: (1) never use, (2) ever use (excluding past 12-month (P12M) use), (3) P12M use (excluding P30D use), (4) P30D low frequency use (1–5 days), and (5) P30D high frequency use (6+ days).
Study funding	"This work was supported by Federal funds from the National Institute on Drug Abuse, National Institutes of Health; and the Center for Tobacco Products, Food and Drug Administration, Department of Health and Human Services, under a contract to Westat (Contract No. HHSN271201100027C). The findings

	and conclusions in this report are those of the authors and do not necessarily represent the official position of the U.S. Department of Health and Human Services or any of its affiliated institutions or agencies."
Author declarations	"K. Michael Cummings has received grant funding from the Pfizer, Inc., to study the impact of a hospital-based tobacco cessation intervention. Dr. Cummings also receives funding as an expert witness in litigation filed against the tobacco industry. Maciej Goniewicz has received a research grant from Pfizer and served as a member of a scientific advisory board to Johnson & Johnson, a pharmaceutical company that manufactures smoking cessation medications. Wilson Compton reports holding stock in General Electric, 3M Companies and Pfizer. Jennifer Pearson is an expert witness for the Plaintiffs in a multi-district litigation invoking American Spirit Cigarettes.<br>No financial disclosures were reported by the other authors of this paper."

Stokes AC, Wilson AE, Lundberg DJ, Xie W, Berry KM, Fetterman JL, et al. Racial/Ethnic Differences in Associations of Non-cigarette Tobacco Product Use With Subsequent Initiation of Cigarettes in US Youths. Nicotine & Tobacco Research 2021;23(6):900-908.

Study characteristics

Methods	Design: Longitudinal cohort (Individual level study)<br>Recruitment: via the Population Assessment of Tobacco and Health Study (PATH) - waves 1-4<br>Setting: Nationally representative sample<br>Study start date/end date: 2013/2018<br>Number of datapoints: 4<br>Primary dataset: PATH<br>Country: USA
Participants	Total N: 29 788 person-intervals constituted 13 934 unique person-level observations.<br>Age: 14.3 years<br>EC use at baseline: Ever use 6.3%; past-30 days use 1.2%<br>Gender/sex: Female 48.7%<br>Ethnicity/race: non-Hispanic white 53.3%, non-Hispanic black 13.9%, Hispanic 23.1%<br>Measures of socioeconomic status: Parental education - Parent completed college or higher 40.1%
Exposures	Electronic cigarettes Use: Ever use, Past-30 days use<br>Details on EC devices: Not reported<br>Electronic cigarettes availability: Not applicable

Outcomes	Methods: “The authors used nationally representative, longitudinal data from the Population Assessment of Tobacco and Health Study waves 1–4. The sample was a dynamic cohort of cigarette-naïve youth aged 12–17 years. Mixed-effects models were used to assess non-cigarette product (e-cigarette, cigar product, or other product) use with cigarette use over 1-year intervals.”<br>Adjustment for confounders: “Regression models were adjusted for sex, age, race/ethnicity, parental education level (bachelors or higher), ever alcohol use, ever marijuana use, ever prescription drug abuse, interval, and cigarette susceptibility.”<br>Type of combustible tobacco use: cigarette<br>Combustible tobacco use: ever use, past 30 days use<br>Odds ratio of non-cigarette tobacco product ever use with subsequent cigarette use.
Study funding	“Research reported in this publication was supported by the National Heart, Lung, and Blood Institute of the National Institutes of Health and the US Food and Drug Administration’s Center for Tobacco Products (grants P50HL120163, U54HL120163, and 1K01HL154130-01). The content is solely the responsibility of the authors and does not necessarily represent the official views of the study sponsors.”
Author declarations	“Dr Stokes reported receiving grants from Ethicon, a subsidiary of Johnson and Johnson, outside the submitted work. The remaining co-authors report no conflict of interest.”

Sumbe A, Clendennen SL, Opara SC, Jackson CD, Chen B, Wilkinson AV, et al . ENDS Device Type and Initiation of Combustible Tobacco Products Among Adolescents. Nicotine Tob Res 23;3:479-486.

Study characteristics

Methods	Design: Longitudinal cohort (individual level study)<br>Recruitment: via the Texas Adolescent Tobacco and Marketing Surveillance System<br>Setting: a web-based survey for students<br>Study start date/end date: 2014/2018<br>Number of datapoints: 8<br>Primary dataset: Texas Adolescent Tobacco and Marketing Surveillance System (TATAMS). Eight waves of a web-based survey on ENDS and other tobacco use behaviors were collected every 6 months.<br>Country: USA
Participants	Total N: Ever ENDS users n = 1324 of N = 151 784 (TATAMS Waves 1–8 (2014–2018))<br>Age: grade used as proxy for age.<br>Sixth grade 13.4%<br>Eighth grade 33.82%<br>Tenth grade 52.78%<br>EC use at baseline: 100%; all Ever ENDS users<br>Gender/sex: Male 50.66%, Female 49.34%<br>Ethnicity/race:

	Hispanic 55.44%<br>Non-Hispanic White 19.41%<br>Non-Hispanic Black 14.48%<br>Other 10.67%<br>Measures of socioeconomic status:<br>High 18.38%<br>Middle 59.53%<br>Low 21.88%
Exposures	Electronic cigarettes Use: Ever use<br>Details on EC devices:<br>Cartridges 8.25%<br>Disposables 7.92%<br>Refillables 34.89%<br>Don't remember 11.70%<br>Not reported/missing 37.24%<br>Electronic cigarettes availability: not applicable
Outcomes	Methods: "The aim of this study was to determine whether Electronic Nicotine Delivery Systems (ENDS) device type (disposable devices, replaceable cartridges, and refillables) at initial or first ENDS use predicts subsequent initiation of combustible tobacco products (cigarettes, hookah, cigars) among adolescents and/or differentiates between those who initiate use of both ENDS and combustible tobacco products at the same time. The study examined data from the Texas Adolescent Tobacco and Marketing Surveillance System (TATAMS), a longitudinal population-based cohort of students in major metropolitan areas of Texas (n = 3907; N = 461 069). Data were collected every 6 months, from 2014 to 2018; 33.9%<br>(n = 1324; N = 151 784) of the sample-initiated ENDS use across this period. Unadjusted and adjusted logistic regression models were used to assess the odds of initiating combustible tobacco<br>products at a subsequent or similar wave as ENDS initiation, given initial ENDS device type."<br>Adjustment for confounders: Controlled for sociodemographic variables.<br>Type of combustible tobacco use: cigarettes<br>Combustible tobacco use: ever use
Study funding	"This work was supported by grant number 1 P50 CA180906 from the National Cancer Institute and the Food and Drug Administration Center for Tobacco Products (FDA CTP). Funding was also provided by the National Cancer Institute through the grant R01-CA239097. The content is solely the responsibility of the authors and does not necessarily represent the official views of the National Institutes of Health or the Food and Drug Administration."
Author declarations	"M.B.H. is a consultant in litigation involving the vaping industry. Other authors have no conflicts of interest to disclose."

Sun R, Mendez D, Warner KE. Is Adolescent E-Cigarette Use Associated With Subsequent Smoking? A New Look. Nicotine Tob Res 2022;24(5):710-718.

Study characteristics

Methods	Design: Longitudinal cohort<br>Recruitment: via PATH<br>Setting: Nationally representative sample<br>Study start date/end date: 2013/2019<br>Number of datapoints: 5<br>Primary dataset: PATH<br>Country: USA
Participants	Total N: 11 560<br>Age: 12–14 50.7%, 15–17 49.3%<br>EC use at baseline: Ever vaped Yes 11.3%, No 88.7%<br>Gender/sex: Male 51.8%, Female 48.2%<br>Ethnicity/race:<br>Non-Hispanic white 46.2%<br>Non-Hispanic black 13.4%<br>Hispanic 30.6%<br>Non-Hispanic other 9.8%<br>Measures of socioeconomic status:<br>Highest parental education<br>High school/GED or less 29.4%<br>Some college 29.7%<br>College or higher 40.9%<br>Household income<br><50k 44.0%<br>50k to 100k 25.7%<br>>100k 30.3%
Exposures	Electronic cigarettes Use: ever use<br>Details on EC devices: not reported<br>Electronic cigarettes availability: not applicable
Outcomes	Methods: "Using longitudinal data from the Population Assessment of Tobacco and Health (PATH) Study, we employed multivariable logistic regressions to assess the adolescent vaping-to-smoking relationship, with four regressions (Models 1–4) sequentially adding more risk factors. Our sample included all waves (waves 1–5) of the PATH Study."<br>Adjustment for confounders: "Control variables in each model:<br>Model 1: Sociodemographic variables.<br>Model 2: Model 1 + exposure to tobacco users (family tobacco use, second-hand smoke, friends' tobacco use).

	Model 3: Model 2 + cigarette susceptibility.<br>Model 4: Model 3 + behavioural risk factors (ever use of other tobacco products, past 12-month use of alcohol and marijuana)"<br>Type of combustible tobacco use: cigarettes<br>Combustible tobacco use: past 30 days
Study funding	"The authors received no specific funding for this work."
Author declarations	"None declared."

Sun R, Mendez D, Warner KE. Association of Electronic Cigarette Use by US Adolescents With Subsequent Persistent Cigarette Smoking. JAMA Network Open 2023;6(3):e234885.

Study characteristics

Methods	Design: Longitudinal cohort (individual level study)<br>Recruitment: via PATH<br>Setting: nationally representative sample of U.S youth<br>Study start date/end date: start Wave 3 (October 2015-October 2016), end Wave 5 (December 2018-November 2019)<br>Number of datapoints: 3<br>Primary dataset: PATH<br>Country: USA
Participants	Total N: 8671<br>Age: 12-17 years<br>12-14 years n=4823 weighted % (95%CI) 55.4 (54.8-56.1)<br>15-17 years n= 3848 weighted % (95%CI) 44.6 (43.9-45.3)<br>EC use at baseline: n=138 current e-cigarette users (and 842 ever e-cig users, out of total 8671 never cigarette users)<br>Gender/sex: Male n=4454 weighted % (95%CI) 51.1 (50.5-51.8)<br>Female n=4195 weighted % (95%CI) 48.9 (48.3-49.5)<br>Ethnicity/race:<br>Hispanic n=2606 weighted % (95%CI) 4.5 (23.9-25.2)<br>Non-Hispanic<br>Black n=1199 weighted % (95%CI) 14.2 (13.6-14.7)<br>White n=3763 weighted % (95%CI) 51.0 (50.3-51.8)<br>Other n=792 weighted % (95%CI) 0.3 (9.8-10.8)<br>Measures of socioeconomic status:

	Highest parental education<br>High school or general educational development or less n=3057 weighted % (95%CI) 31.7 (30.0-33.5), Some college n=2700 weighted % (95%CI) 31.2 (29.6-32.9), College or higher n=2733 weighted % (95%CI) 37.0 (34.9-39.3)<br>Annual household income<br><50 000$ n= 3956 weighted % (95%CI) 43.7 (41.8-45.5) 57 1.5 (1.2-2.1)<br>50 000-100 000$ n=2082 weighted % (95%CI) 26.7 (25.2-28.1)<br>>100 000$ n=2060 weighted % (95%CI) 29.7 (27.5-32.0)<br>School grades<br>Less than mostly Bs n=2383 weighted % (95%CI) 5.7 (24.5-27.0)<br>Mostly Bs and higher n=6228 weighted % (95%CI) 74.3 (73.0-75.5)
Exposures	Electronic cigarettes Use: Ever and current (past 30-day)<br>Details on EC devices: Not reported<br>Electronic cigarettes availability: Not applicable
Outcomes	Methods: "This sample consisted of youth who participated in waves 3, 4, and 5 of the PATH study (wave 3 was from October 2015 to October 2016, wave 4 was from December 2016 to January 2018, and wave 5 was from December 2018 to November 2019) and had never used cigarettes (cigarette-naive) by wave 3. The current analysis used multivariable logistic regressions in August 2022 to assess the association between e-cigarette use among cigarette-naive adolescents aged 12 to 17 years in 2015 and 2016 and subsequent continued cigarette smoking."<br>Adjustment for confounders: models adjusted for all study covariates "Sociodemographic variables included age (12-14 vs 15-17 years), sex (male vs female), race and ethnicity (Hispanic, non-Hispanic Black, non-Hispanic White, and non-Hispanic other, highest parental education (high school or general educational development or less, some college, and college or higher), annual household income (<$50 000, $50 000-$100 000, and >$100 000), and school grades (less than mostly Bs vs mostly Bs and higher)."<br>Type of combustible tobacco use: cigarettes<br>Combustible tobacco use: any use in last 12 months, any use in past 30 days
Study funding	"Drs Méndez and Warner received support from the National Cancer Institute of the National Institutes of Health (NIH) and the Food and Drug Administration's (FDA) Center for Tobacco Products (award number U54CA229974)."
Author declarations	"None reported"

Sutfin EL, Reboussin BA, Debinski B, Wagoner KG, Spangler J, Wolfson M. The Impact of Trying Electronic Cigarettes on Cigarette Smoking by College Students: A Prospective Analysis. American Journal of Public Health 2015;105(8):e83-9.

Study characteristics

Methods	Design: Longitudinal cohort (Individual level study)<br>Recruitment: via the Smokeless Tobacco Use in College Students study

	Setting: colleges<br>Study start date/end date: autumn 2010/ autumn 2013<br>Number of datapoints: 6<br>Primary dataset: Smokeless Tobacco Use in College Students study<br>Country: USA
Participants	Total N: 271<br>Age: 18 and over<br>EC use at baseline: none<br>Gender/sex: Female 51.7%<br>Ethnicity/race: white 243 (89.7%), non-white 28 (10.3%), Hispanic 5.9%, non-Hispanic 94.1%<br>Measures of socioeconomic status: Mother's education - mother with a college degree or higher 59.4%
Exposures	Electronic cigarettes Use: Ever use<br>Details on EC devices: Not reported<br>Electronic cigarettes availability: Not applicable
Outcomes	Methods: "In this longitudinal study, first-semester college students at 7 colleges in North Carolina and 4 in Virginia completed a baseline survey and 5 follow-up surveys between fall 2010 and fall 2013. Current cigarette smoking at wave 6 was the primary outcome. Participants (n = 271) reported current cigarette smoking at baseline and no history of e-cigarette use. We measured trying e-cigarettes at each wave, defined as use in the past 6 months."<br>Adjustment for confounders: "adjustment for potential confounding variables."<br>Type of combustible tobacco use: cigarette<br>Combustible tobacco use: ever use<br>Odds ratio of current cigarette smoking at wave 6 after trying e-cigarettes on previous waves.
Study funding	"This research was supported by the National Cancer Institute, National Institutes of Health (award R01CA141643)"
Author declarations	Not reported.

Treur JL, Rozema AD, Mathijssen JJP, van Oers H, Vink JM. E-cigarette and waterpipe use in two adolescent cohorts: cross-sectional and longitudinal associations with conventional cigarette smoking. Eur J Epidemiol 2018;33(3):323-334.

Study characteristics

Methods	Design: Longitudinal cohort (cohort I) and cross-sectional analyses (individual level study)<br>Recruitment: via original cohorts<br>Setting: Educational institutions in the Netherlands

	Study start date/end date: 2014/2015<br>Number of datapoints: 2 (Cohort I)<br>Primary dataset: Two Dutch Cohorts:<br>Cohort I consists of 6819 adolescents aged 11–17 years [mean age = 13.8 (SD = 1.1), 48.2% female] who were enrolled in a study that investigated the impact of school smoking policy on changes in adolescents' smoking behaviour. Data were collected in 2014–2015 from 19 secondary schools randomly selected across the Netherlands. Longitudinal data at baseline and 6 months in 2100.<br>Cohort II consists of 2758 adolescent participants of the<br>Trends study (Traditional and Novel Substance use among<br>Adolescents) aged 14 to 21 years [mean age = 17.3 (SD = 1.8), 61.3% female]. Trends aims to assess addictive behaviour in a representative group of Dutch adolescents and young adults, with a particular focus on 'novel' types of addictive behaviour, including the use of alternative tobacco products. Data were collected in 2016–2017 from 14 educational institutions located mostly in the West of the Netherlands.<br>Country: The Netherlands
Participants	Total N: Cohort I N=6819 (n=2100 for longitudinal analyses), Cohort II N=2758<br>Age: Mean age Cohort I 13.8 (SD 1.1), Cohort II 17.3 (SD 1.8)<br>EC use at baseline: Ever use prevalence was 13.7% for e-cigarettes with nicotine, 29.4% for e-cigarettes without nicotine in Cohort I and 12.3% and 27.6% respectively in Cohort II.<br>Gender/sex: Female % Cohort I 48.2%, Cohort II 61.3%<br>Ethnicity/race:<br>Cohort I: Netherlands (n = 5328), Surinam/Aruba/Netherlands Antilles (n = 124), Morocco (n = 201), Turkey (n = 137) Other (n = 689)<br>Cohort II: Netherlands (n = 2246), Surinam/Aruba/Netherlands Antilles (n = 52), Morocco (n = 56), Turkey (n = 57) Other (n = 114)<br>Measures of socioeconomic status:<br>Educational level<br>Cohort I: Low (n = 2280), Average (n = 2132), Middle (n = 1174), High (n = 1105)<br>Cohort II: Low/average (n = 942), Middle (n = 754) High (n = 1012)
Exposures	Electronic cigarettes Use: ever use, past 30 days<br>Details on EC devices: e-cigarettes with and without nicotine<br>Electronic cigarettes availability: not applicable
Outcomes	Methods: "Alternative tobacco products are increasing in popularity. An important question is whether their use is associated with or even leads to conventional smoking, but large-scale (European) studies are scarce. In two cohorts of Dutch adolescents (Cohort I n = 6819, mean age = 13.8 SD = 1.1, 48.2% female; Cohort II n = 2758, mean age = 17.3 SD = 1.8, 61.3% female), we investigated use of electronic (e)-cigarettes with nicotine, e-cigarettes without nicotine and waterpipe. Generalized estimating equation modelling was conducted with ever conventional smoking as the dependent variable (0 = no,1 = yes) and ever alternative tobacco use as the independent variable, correcting for clustering within schools, age, sex and education in both cohorts. In a subsample (n = 2100), the association between alternative tobacco use at baseline and conventional smoking 6 months later was tested, taking into account smoking propensity (based on personality, susceptibility to peer pressure and smoking intentions)."<br>Type of combustible tobacco use: cigarettes<br>Combustible tobacco use: ever use

Study funding	"This work was supported by the European Research Council (ERC; 284167), Netherlands Organization for Health Research and Development (ZonMw; 200100003) and the National Institute for Public Health and the Environment (RIVM)."
Author declarations	Not reported.

Unger JB, Soto DW, Leventhal A. E-cigarette use and subsequent cigarette and marijuana use among Hispanic young adults. Drug and Alcohol Dependence 2016;163:261-4.

Study characteristics

Methods	Design: Longitudinal cohort (Individual level study)<br>Recruitment: via Project RED<br>Setting: colleges<br>Study start date/end date: 2014/ 2015<br>Number of datapoints: 2<br>Primary dataset: Project RED<br>Country: USA
Participants	Total N: 1332<br>Age: 22.7 (SD 0.39) years<br>EC use at baseline: Past-month use 9%<br>Gender/sex: Female 59%<br>Ethnicity/race: Not reported<br>Measures of socioeconomic status: Not reported
Exposures	Electronic cigarettes Use: Past-30 days use<br>Details on EC devices: Not reported<br>Electronic cigarettes availability: Not applicable
Outcomes	Methods: “Survey data were collected from 1332 Hispanic young adults (59% female, mean age = 22.7 years, SD = 0.39 years) in 2014 and 2015. Logistic regression analyses examined the association between e-cigarette use in 2014 and cigarette/marijuana use in 2015, controlling for age, sex, and other substance use.”<br>Type of combustible tobacco use: cigarette<br>Combustible tobacco use: past-30 days use<br>Odds ratio of past-month cigarette smoking in 2015 following e-cigarette use.
Study funding	“This research was supported by the National Institutes of Health (grant 5R01DA016310).”

Author declarations	"The authors report no conflicts of interest."

Wang MP, Li WH, Wu Y, Lam TH, Chan SS. Electronic cigarette use is not associated with quitting of conventional cigarettes in youth smokers. Pediatric Research 2017;82(1):14-18.

Study characteristics

Methods	Design: Longitudinal cohort (Individual level study)<br>Recruitment: via Youth Quitline in Hong Kong<br>Setting: smoking cessation service<br>Study start date/end date: 2014-2015/ 6 months after baseline<br>Number of datapoints: 2<br>Primary dataset: longitudinal data from the Youth Quitline in Hong Kong<br>Country: Hong Kong
Participants	Total N: 189<br>Age: 18.1 (SD 2.7) years<br>EC use at baseline: ever use 112(59.3%)<br>Gender/sex: Male 82%<br>Ethnicity/race: Not reported<br>Measures of socioeconomic status:<br>Education: Highest education - Junior second or below 24.3%; Senior second or diploma 57.2%; Sub-degree/higher diploma or above 18.5%<br>Occupation: Work/study status - Full-time student 74.6%; Employed or self-employed 16.6%; Unemployed 1.7%; Others 7.2%
Exposures	Electronic cigarettes Use: ever use<br>Details on EC devices: Not reported<br>Electronic cigarettes availability: Not applicable
Outcomes	Methods: "This longitudinal study collected data on youth smokers' (*N*=189) use and perception of e-cigs, conventional cigarette smoking behaviour, and sociodemographic characteristics at baseline. Self-reported past 7-day point prevalence of abstinence (PPA) was assessed in the 6-month telephone follow-up. Linear and logistic regressions were used to estimate the association of e-cig use with quitting cigarette smoking and other cessation-related outcomes."<br>Adjustment for confounders: "adjusting for model 1 variables (adjusting for baseline respective level) and sex, age, smoking friends, and smoking family members."<br>Type of combustible tobacco use: cigarette<br>Combustible tobacco use: past week use<br>Odds ratio of quitting cigarette smoking at the 6-month follow up following e-cigarette use.
Study funding	"The Youth Quitline is funded by Tobacco Control Office, Department of Health, Government of Hong Kong SAR."

Author declarations	“The authors declare no conflict of interest.”

Wang G, Wu L. Healthy People 2020: Social Determinants of Cigarette Smoking and Electronic Cigarette Smoking among Youth in the United States 2010-2018. International Journal of Environmental Research and Public Health 2020;17(20):7503.

Study characteristics

Methods	Design: Repeated cross-sectional surveys (Individual level study)<br>Recruitment: via National Health Interview Survey (NHIS) (2018, 2014, 2010)<br>Setting: household surveys<br>Study start date/end date: 2010/2018<br>Number of datapoints: 3<br>Primary dataset: NHIS<br>Country: USA
Participants	Total N: 3281 in 2010, 3981 in 2014, 2195 in 2018.<br>Age: 18-25 years<br>EC use at baseline: Ever use e-cigs 2014: 18.2-26.1% across age in years; 2018 23.3-33.6% across age in years<br>Gender/sex: 2010;2014;2018<br>Male 1532 46.70%; 1955 49.10%; 1103 50.30%<br>Female 1749 53.30%; 2026 50.90%; 1092 49.70%<br>Ethnicity/race: 2010;2014;2018<br>Non-Hispanic White 1619 49.30%; 2241 56.30%; 1251 57.00%<br>Non-Hispanic Black 576 17.60%; 573 14.40%; 274 12.50%<br>Hispanic 844 25.70%; 840 21.10%; 453 20.60%<br>Other race group 242 7.40%; 327 8.20%; 217 9.90%<br>Measures of socioeconomic status: 2010;2014;2018<br>Income-to-Poverty ratio<br>Less than 100% 1057 34.90%; 1309 34.20%; 488 22.90%<br>100–199% 725 23.90%; 923 24.10%; 480 22.60%<br>200% and greater 1249 41.20%; 1599 41.70%; 1160 54.50%<br>Education<br>Less than high school 535 16.40%; 478 12.00%; 239 10.90%<br>High school/GED 905 27.70%; 1085 27.30%; 584 26.60%<br>Some college 1335 40.80%; 1751 44.00%; 896 40.90%<br>Bachelor and above 497 15.20%; 664 16.70%; 474 21.60%
Exposures	Electronic cigarettes Use: ever use

	Details on EC devices: Not reported<br>Electronic cigarettes availability: Not applicable
Outcomes	Methods: "Using secondary data from National Health Interview Surveys (NHIS) across the 2010, 2014, and 2018 survey years, this study analyzed the prevalence rates of cigarette smoking and ever using e-cigarettes between 2010 and 2018, demographic and socioeconomic disparities in smoking, and the relationship between previous e-cigarette use and current smoking. Logistic regressions were conducted to test the relationship between sociodemographic factors, being a previous electronic cigarette smoker, and being a current smoker. Odds ratios and 95% confidence intervals were reported."<br>Type of combustible tobacco use: cigarette<br>Combustible tobacco use: "smoking every day or some days"<br>Odds ratio of current cigarette smoking and previous e-cigarette use.
Study funding	"We gratefully acknowledge the support of the Research Project of Young Scholars, "Innovations of Data Protection Regulations", in Humanities and Social Sciences of Wuhan University."
Author declarations	"The authors declare no conflict of interest."

Watkins SL, Glantz SA, Chaffee BW. Association of Noncigarette Tobacco Product Use With Future Cigarette Smoking Among Youth in the Population Assessment of Tobacco and Health (PATH) Study, 2013-2015. JAMA Pediatr 2018;172(2):181-187.

Study characteristics

Methods	Design: Longitudinal cohort (individual level study)<br>Recruitment: via PATH<br>Setting: Nationally representative sample<br>Study start date/end date: wave 1 (2013/2014)/ wave 2 (2014/2015)<br>Number of datapoints: 2<br>Primary dataset: PATH<br>Country: USA
Participants	Total N: the analysis was based on the 10 384 PATH youth respondents who reported never having smoked a cigarette in wave 1 and whose cigarette ever or past 30-day use was reported in wave 2.<br>Age: mean 14.3 (SD 1.7) years<br>EC use at baseline: ever use 4.2%, past 30 days use 0.9%, ever only use 2.6%<br>Gender/sex: Female 49.1%<br>Ethnicity/race:<br>White 52.5%<br>African American 13.9%<br>Latino 22.3%<br>Other 11.3%

	Measures of socioeconomic status:<br>Parent's educational level (bachelor's degree or higher) 44.8%<br>Urban residence 80.7%
Exposures	Electronic cigarettes Use: ever use, past 30 days, ever only use<br>Details on EC devices: not reported<br>Electronic cigarettes availability: not applicable
Outcomes	Methods: "To estimate the longitudinal association between noncigarette tobacco use and subsequent cigarette smoking initiation among US youth. In this prospective cohort study of the Population Assessment of Tobacco and Health (PATH) waves 1 (September 12, 2013, to December 14, 2014) and 2 (October 23, 2014, to October 30, 2015), a nationally representative sample of youths who never smoked a conventional cigarette at baseline and completed wave 2 follow-up (N = 10 384) was studied. PATH retention at follow-up was 87.9%."<br>Adjustment for confounders: "the following wave 1<br>covariates: female, age, race/ethnicity, parental educational level, urban residence, sensation seeking, alcohol ever use, living with tobacco user, notice of cigarette warning labels, tobacco advertising receptivity, and summer season."<br>Type of combustible tobacco use: cigarettes<br>Combustible tobacco use: ever use, past 30 days
Study funding	"This work was supported in part by grant P50 CA180890 from the US National Cancer Institute and Food and Drug Administration Center for Tobacco Products (Drs Watkins, Glantz, and Chaffee), grant R01DA043950 from the National Institute on Drug Abuse (Dr Glantz), grant KL2TR000143 from the US National Center for Advancing Translational Sciences<br>(Dr. Chaffee), and grant T32CA113710-11 from the National<br>Cancer Institute (Dr Watkins)."
Author declarations	"None reported."

Westling E, Rusby JC, Crowley R, Light JM. Electronic Cigarette Use by Youth: Prevalence, Correlates, and Use Trajectories From Middle to High School. Journal of Adolescent Health 2017;60(6):660-666.

Study characteristics

Methods	Design: Longitudinal cohort (Individual level study)<br>Recruitment: via Web-based computer surveys at 11 middle schools in seven school districts in Oregon<br>Setting: middle schools<br>Study start date/end date: 2014/2016<br>Number of datapoints: 4<br>Primary dataset: original dataset<br>Country: USA

Participants	Total N: 1,091<br>Age: 14.4 (SD 0.5) years<br>EC use at baseline: 27.7% lifetime use; 16.6% current use (past 30 days)<br>Gender/sex: Male 47%<br>Ethnicity/race: non-Hispanic white 53%, Hispanic 37%, Native American 4%, African-American 2%, Asian or Pacific Islander 1%, more than one race 12%, remainder unknown race/ethnicity.<br>Measures of socioeconomic status: not reported
Exposures	Electronic cigarettes Use: ever use, past 30 days use<br>Details on EC devices: Not reported<br>Electronic cigarettes availability: Not applicable
Outcomes	Methods: "Participants (N = 1,091) from seven school districts in Oregon, United States, completed four self-report surveys on substance use, from the spring of eighth grade through the spring of ninth grade. Growth mixture modeling (GMM) was used to explore trajectories of current usage of e-cigarettes; GMM is a person-centered technique that can identify differences in longitudinal change among unobserved groups (i.e., classes)."<br>Type of combustible tobacco use: cigarette<br>Combustible tobacco use: ever use, past-30 days use<br>Correlates of lifetime e-cigarette use by eight grade and trajectories of current use across ninth grade.
Study funding	"This research was supported by the National Institute on Drug Abuse at the National Institutes of Health (Grant # R01-DA034062). The funder had no role in the design and conduct of the study; collection, analysis, or interpretation of the data; or preparation, review, or approval of the manuscript."
Author declarations	Not reported.

Wills TA, Gibbons FX, Sargent JD, Schweitzer RJ. How is the effect of adolescent e-cigarette use on smoking onset mediated: A longitudinal analysis. Psychology of Addictive Behaviors 2016;30(8):876-886.

Study characteristics

Methods	Design: Longitudinal cohort (Individual level study)<br>Recruitment: Data were obtained through a school-based study conducted with six high school students in Oahu, Hawaii. Parents and students provided consent.<br>Setting: high schools<br>Study start date/end date: 2013/2014<br>Number of datapoints: 2<br>Primary dataset: original dataset<br>Country: USA

Participants	Total N: T1=2338, T2=2239<br>Age: 14.7 (0.7) years<br>EC use at baseline: ever use 31%<br>Gender/sex: Female 53%<br>Ethnicity/race: Asian-American background (Chinese, Japanese, or Korean) 24%, Caucasian 19%, Filipino-American 27%, Native Hawaiian or other Pacific Islander 20%r and other race/ethnicity 10%.<br>Measures of socioeconomic status: “Father’s education on a 1–6 scale with anchor points grade school and post-college was 4.2 (SD = 1.2).”
Exposures	Electronic cigarettes Use: ever use<br>Details on EC devices: not reported<br>Electronic cigarettes availability: Not applicable
Outcomes	Methods: “The authors assessed e-cigarette use, cigarette smoking, demographic covariates, and four hypothesized mediators: smoking-related expectancies, prototypes, and peer affiliations as well as marijuana use. The primary structural modeling analysis, based on initial never-smokers, used an autoregressive model (entering T2 mediator values adjusted for T1 values) to test for mediational pathways in the relation between e-cigarette use at T1 and cigarette smoking status at T2.”<br>Adjustment for confounders: “The covariates were gender (dichotomous), ethnicity (four binary indices, with Asian-American as the reference group), family structure (three binary indices, with intact family as the reference group), and father’s education (6-point scale).”<br>Type of combustible tobacco use: cigarette<br>Combustible tobacco use: ever use<br>Association of e-cigarette use with cigarette smoking.
Study funding	“This work was supported by grants R01 CA153154 and P30 CA071789-16S2 from the National Cancer Institute.”
Author declarations	Not reported
Notes	Same samples as Wills 2017 and same dataset as Wills 2017b

Wills TA, Knight R, Sargent JD, Gibbons FX, Pagano I, Williams RJ. Longitudinal study of e-cigarette use and onset of cigarette smoking among high school students in Hawaii. Tobacco Control 2017;26(1):34-49.

Study characteristics

Methods	Design: Longitudinal cohort (Individual level study)<br>Recruitment: Data were obtained through a school-based study conducted with six high school students in Oahu, Hawaii. Parents and students provided consent.<br>Setting: schools<br>Study start date/end date: 2013/2014<br>Number of datapoints: 2

	Primary dataset: original dataset<br>Country: USA
Participants	Total N: T1=2338, T2=2239<br>Age: 14.7 (0.7) years<br>EC use at baseline: ever use 31%<br>Gender/sex: Female 53%<br>Ethnicity/race: Asian-American background (Chinese, Japanese, or Korean) 24%, Caucasian 19%, Filipino-American 27%, Native Hawaiian or other Pacific Islander 20% and other race/ethnicity 10%.<br>Measures of socioeconomic status: “Father’s education on a 1–6 scale with anchor points grade school and post-college was 4.2 (SD = 1.2).”
Exposures	Electronic cigarettes Use: ever use<br>Details on EC devices: not reported<br>Electronic cigarettes availability: Not applicable
Outcomes	Methods: “Longitudinal school-based survey with a baseline sample of 2338 students (9th and 10th graders, mean age 14.7 years) in Hawaii surveyed in 2013 (time 1, T1) and followed up 1 year later (time 2, T2). We assessed e-cigarette use, tobacco cigarette use, and psychosocial covariates (demographics, parental support and monitoring, and sensation seeking and rebelliousness). Regression analyses including the covariates tested whether e-cigarette use was related to the onset of smoking among youth who had never smoked cigarettes, and to change in smoking frequency among youth who had previously smoked cigarettes.”<br>Adjustment for confounders: “All multivariable analyses controlled for demographics, parenting and personality variables, and adjusted for clustering within schools. “<br>Type of combustible tobacco use: cigarette<br>Combustible tobacco use: ever use<br>Odds ratio of ever smoking at T2 as a function of T1 e-cigarette use among T1 never smokers.
Study funding	“This research was supported by grants R01 CA153154 and P30071789-16S2 from the National Cancer Institute.”
Author declarations	"None declared."
Notes	Same sample as Wills 2016

Wills TA, Sargent JD, Gibbons FX, Pagano I, Schweitzer R. E-cigarette use is differentially related to smoking onset among lower risk adolescents. Tobacco Control 2017;26(5):534-539.

Study characteristics

Methods	Design: Longitudinal cohort (Individual level study)

	Recruitment: Data were obtained through a school-based study conducted with six high school students in Oahu, Hawaii. Parents and students provided consent.<br>Setting: high schools<br>Study start date/end date: 2013/2014<br>Number of datapoints: 2<br>Primary dataset: original dataset<br>Country: USA
Participants	Total N: 1136<br>Age: 14.8 (0.7) years<br>EC use at baseline: ever use 18%<br>Gender/sex: Female 57%<br>Ethnicity/race: Asian-American background (Chinese, Japanese, or Korean) 34%, Caucasian 17%, Filipino-American 25%, Native Hawaiian or other Pacific Islander 17% and other race/ethnicity 7%.<br>Measures of socioeconomic status: Fathers education<br>Grade school <1%<br>Some high school 4%<br>High school graduate 25%<br>1-2 years college 16%<br>College graduate 42%<br>Post-college 13%
Exposures	Electronic cigarettes Use: ever use<br>Details on EC devices: not reported<br>Electronic cigarettes availability: Not applicable
Outcomes	Methods: "School-based survey with a longitudinal sample of 1136 students (9th–11th graders, mean age 14.7 years) in Hawaii, initially surveyed in 2013 (T1) and followed up 1 year later (T2). We assessed e-cigarette use, propensity to smoke based on 3 psychosocial factors known to predict smoking (rebelliousness, parental support and willingness to smoke), and cigarette smoking status. Analyses based on T1 never-smokers tested the relation of T1 e-cigarette use to T2 smoking status for participants lower versus higher on T1 propensity to smoke."<br>Adjustment for confounders: "Level 1 variables were the covariates (gender, ethnicity and parental education), T1 e-cigarette use, T1 smoking propensity and their cross-product; school was a level 2 variable. Gender was coded as a binary variable, ethnicity was coded with four binary variables (Caucasian, Filipino, Native Hawaii and other ethnicity with Asian-American as the reference group) and father's education was a six-point scale. "<br>Type of combustible tobacco use: cigarette<br>Combustible tobacco use: ever use, other<br>Odds ratio of smoking onset by T2 and T1 e-cigarette use.
Study funding	"This research was supported by grants R01 CA153154 and P30071789-16S2 from the National Cancer Institute."

Author declarations	"None declared."
Notes	Same dataset as Wills 2016 and Wills 2017

Wu DC, Essue BM, Jha P. Impact of vaping introduction on cigarette smoking in six jurisdictions with varied regulatory approaches to vaping: an interrupted time series analysis. BMJ Open 2022;12: e058324.

Study characteristics

Methods	Design: Interrupted time series analysis (population level study)<br>Recruitment: Not applicable<br>Setting: Not applicable<br>Study start date/end date: 2012 to 2019<br>Number of datapoints: Not applicable<br>Primary dataset: Obtained prevalence of past 30 days' e-cigarette use, by province, from the Canadian Tobacco, Alcohol and Drugs Survey. In the UK, used prevalence of current e-cigarette use reported by Action on Smoking and Health based on annual surveys. For Australia, used data from the National Drug Strategy Household Survey (NDSHS). Estimated cigarette consumption for each country using sales data.<br>Country: four Canadian provinces (Alberta, Ontario, Quebec, BC), UK and Australia
Participants	Total N: Entire population of smokers in each country<br>Age: Not reported<br>EC use at baseline: Across all study settings, the prevalence of current e-cigarette use was variable over time, but low overall (<7%).<br>Gender/sex: Not reported but measured<br>Ethnicity/race: Not reported<br>Measures of socioeconomic status: Not reported
Exposures	Electronic cigarettes Use: not applicable<br>Details on EC devices: not applicable<br>Electronic cigarettes availability: the year that vaping was widely introduced in each country.
Outcomes	Methods: "We sought to quantify the impact of vaping introduction on cigarette smoking across settings with varied regulatory approaches to vaping. Design was an interrupted time series analysis, adjusted for cigarette tax levels. Setting was four Canadian provinces, UK and Australia. Participants included the entire population of smokers in each country. The intervention was the year that vaping was widely introduced in each country. The primary outcome is cigarette consumption per adult, and the secondary outcome is smoking prevalence among young adults."<br>Adjustment for confounders: "Our ITS model adjusted for tobacco tax or cigarette price as a potential confounder." Plain packaging and gender also controlled for.<br>Type of combustible tobacco use: cigarettes<br>Combustible tobacco use: prevalence and cigarette consumption

Study funding "This work was supported by the Canadian Institutes of Health Research Foundation scheme (grant number FDN 154277)."

Author declarations "None declared"

Xu S, Coffman DL, Liu B, Xu Y, He J, Niaura RS. Relationships Between E-cigarette Use and Subsequent Cigarette Initiation Among Adolescents in the PATH Study: an Entropy Balancing Propensity Score Analysis. Prev Sci 2022;23(4):608-617.

Study characteristics

Methods
Design: Longitudinal cohort (individual level study)
Recruitment: via PATH
Setting: Nationally representative sample
Study start date/end date: 2013/2014, 2015/2016
Number of datapoints: 3 (waves 1-3)
Primary dataset: PATH
Country: USA

Participants
Total N: 6309
Age: 12 and 15 years at Wave 1
EC use at baseline: tobacco-naïve wave 1, e-cigarette initiators wave 2 (n=414 exclusive e-cigarette users)
Gender/sex: n (%)
Male 3243 (51.4)
Female 3066 (48.6)
Ethnicity/race: n (%)
Hispanic 1862 (29.5)
Not Hispanic 4447 (70.5)
White 4354 (69.0)
Black 1006 (15.9)
Other 949 (15.0)
Measures of socioeconomic status: n (%)
Education level (parent) Less than high school 1247 (19.8)
High school graduate or equivalent 1095 (17.4)
Some college (no degree) or associates degree 1923 (30.5)
Bachelor's degree 1319 (20.9)
Advanced degree 690 (10.9)

Exposures
Electronic cigarettes Use: ever use
Details on EC devices: not reported

	Electronic cigarettes availability: not applicable
Outcomes	Methods: "This study aimed to examine the relationship between electronic cigarette use and subsequent combustible cigarette use, controlling for confounding by using a propensity score method approach. Data from the first three annual waves of the Population Assessment of Tobacco and Health study were analyzed (n = 6309). Participants were tobacco-naïve at Wave 1;used e-cigarettes exclusively (n = 414), used combustible cigarettes exclusively (n = 46), or not used any tobacco products (n = 5849) at Wave 2. We conducted entropy balancing propensity score analysis to examine the association between exclusive e-cigarette or cigarette initiation and subsequent cigarette use at Wave 3, adjusting for non-response bias, sampling bias, and confounding."<br>Type of combustible tobacco use: cigarettes<br>Combustible tobacco use: ever use, past 30 days
Study funding	"This work was supported by a grant from the New York University (NYU) Research Challenge Fund Program. Research reported in this publication was also supported by the National Cancer Institute of the National Institutes of Health (NIH) and FDA Center for Tobacco Products (CTP) under Award Number U54CA229974. The content is solely the responsibility of the authors and does not necessarily represent the official views of the NIH or the Food and Drug Administration."
Author declarations	"DC, BL, YX, and JH report no financial or other relationship relevant to the subject of this article. SX receives the research grants from NYU Research Challenge Fund and NIH/NCI supplement award through Grant U54CA229974. RN receives funding from the Food and Drug Administration Center for Tobacco Products via contractual mechanisms with Westat and the National Institutes of Health. The work presented here is independent of this funding, and does not represent the views or opinions of any government institutes or agencies."

Yang Z, Berhane K, Leventhal AM, Liu M, Barrington-Trimis JL, Thomas DC. Modeling the longitudinal transitions of electronic cigarettes and conventional cigarettes with time-dependent covariates among adolescents. Preventive Medicine 2022;164:107294.

Study characteristics

Methods	Design: Longitudinal cohort (Individual level study)<br>Recruitment: via Happiness and Health Study (H&H)<br>Setting: high schools<br>Study start date/end date: Autumn 2013/ Autumn 2015<br>Number of datapoints: 5<br>Primary dataset: H&H<br>Country: USA
Participants	Total N: 1977<br>Age: mean 14.06 (SD 0.4) years<br>EC use at baseline: e-cigarette only users 170 (8.6%), dual users 32 (1.6%).

	Gender/sex: 56.4% female<br>Ethnicity/race: Non hispanic 54.3%, Hispanic 45.7%<br>Measures of socioeconomic status: not reported
Exposures	Electronic cigarettes Use: past 6 months use<br>Details on EC devices: not reported<br>Electronic cigarettes availability: Not applicable
Outcomes	Methods: “The authors used data from a prospective cohort study of youth in Southern California followed twice annually from Fall 2013 (9th grade) to Fall 2015 (11th grade) (N = 1977). A polytomous logistic regression model was used to simultaneously estimate transition rates for initiation of and abstention from e-cigarettes and cigarettes”<br>Adjustment for confounders: Adjusted for the covariates family history of cigarette use, age, gender, ethnicity, delinquency, friend use and susceptibility to cigarettes use.<br>Type of combustible tobacco use: cigarettes<br>Combustible tobacco use: past 6 months use<br>Odds of cigarette initiation after e-cigarettes use.
Study funding	“Research reported in this publication was supported by grant number U54CA180905 from the National Cancer Institute at the National Institutes of Health and the Food and Drug Administration (FDA) Center for Tobacco Products (CTP). The funder had no role in the design and conduct of the study; collection, management, analysis, or interpretation of the data; or preparation, review, or approval of the manuscript.”
Author declarations	“The authors declare that they have no known competing financial interests or personal relationships that could have appeared to influence the work reported in this paper.”
