# Supplementary material for: Electronic cigarettes and subsequent cigarette smoking in young people: A systematic review
Source: Addiction. 2025 Jan 30;120(6):1090–111. doi: 10.1111/add.16773 (PMC12046492; doi:10.1111/add.16773)
Supplement: Supplementary file 4 — Data S4. Supporting Information. [file ADD-120-1090-s001.pdf]

Supplementary file 4 – Characteristics of excluded studies

List with examples of excluded studies and reasons for exclusion.

Study	Reason for exclusion
Adeosun S. Accessibility to vaping products, vaping, and use of cigarettes among adolescents. Dissertation Abstracts International: Section B: The Sciences and Engineering 2023;84(4-B):No-specified.	Wrong study design
AlDelaimy WK, Myers MG, Leas EC, Strong DR, Hofstetter CR. E-cigarette use in the past and quitting behavior in the future: a population-based study. Am J Public Health 2015;105(6):1213-9.	Wrong population
Aljandaleh H, Bolze C, El-Khoury Lesueur F, Melchior M, Mary-Krause M. Factors Associated with Electronic Cigarette Use among Young Adults: The French "Trajectoires EpideMiologiques en POpulation" (TEMPO) Cohort Study. Subst Use Misuse 2020;55(6):964-972	Wrong population
Amato MS, Boyle RG, Levy D. E-cigarette use 1 year later in a population-based prospective cohort. Tob Control 2017;26(e2):e92-e96.	Wrong population
Ambrose BK, Day HR, Rostron B, Conway KP, Borek N, Hyland A, et al. Flavored tobacco product use among US youth aged 12-17 Years, 2013-2014. JAMA 2015;314(17):1871-1873.	Wrong study design
Anic GM, Holder-Hayes E, Ambrose BK, Rostron BL, Coleman B, Jamal A, et al. E-cigarette and Smokeless Tobacco Use and Switching Among Smokers: Findings From the National Adult Tobacco Survey. Am J Prev Med 2018;54(4):539-551.	Wrong Population
Aonso-Diego G, Secades-Villa R, García-Pérez Á, Weidberg S, Fernández-Hermida JR. Association between e-cigarette and conventional cigarette use among Spanish adolescents. Adicciones 2023;0(0):1797.	Wrong study design
Audrain-McGovern J, Rodriguez D, Pianin S, Testa S. Conjoint Developmental Trajectories of Adolescent E-cigarette and Combustible Cigarette Use. Pediatrics 2021;148(5):e2021051828.	Wrong study design
Auf R, Trepka MJ, Selim M, Ben Taleb Z, De La Rosa M, Bastida E, et al. E-cigarette use is associated with other tobacco use among US adolescents. Int J Public Health 2019;64(1):125-134.	Wrong study design
Balfour DJK, Benowitz NL, Colby SM, Hatsukami DK, Lando HA, Leischow SJ, et al. Balancing Consideration of the Risks and Benefits of E-Cigarettes. Am J Public Health 2021;111(9):1661-1672.	Wrong study design
Benmarhnia T, Pierce JP, Leas E, White MM, Strong DR, Noble ML, et al. Can E-Cigarettes and Pharmaceutical Aids Increase Smoking Cessation and Reduce Cigarette Consumption? Findings From a Nationally Representative Cohort of American Smokers. Am J Epidemiol 2018;187(11):2397-2404.	Wrong population
Brady BR, Crane TE, O'Connor PA, Nair US, Yuan NP. Electronic cigarette use and tobacco cessation in a state-based quitline. Journal of Smoking Cessation 2019;14(3):176 - 185.	Wrong population
Buu A, Hu YH, Piper ME, Lin HC. The association between e-cigarette use characteristics and combustible cigarette consumption and dependence symptoms: Results from a national longitudinal study. Addict Behav 2018; 84:69-74.	No age subgroup analysis
Cahn Z, Haardorfer R, Lewis M, Wang Y, Berg CJ. Examining e-cigarette purchases and cessation in a consumer panel of smokers. Journal of Smoking Cessation 2019;14(1):32-41	Wrong population
Camenga DR, Kong G, Cavallo DA, Krishnan-Sarin S. Current and Former Smokers' Use of Electronic Cigarettes for Quitting Smoking: An Exploratory Study of Adolescents and Young Adults. Nicotine Tob Res 2019;21(3):395.	Wrong study design

Cardenas VM, Evans VL, Balamurugan A, Faramawi MF, Delongchamp RR, Wheeler JG. Use of electronic nicotine delivery systems and recent initiation of smoking among US youth. Int J Public Health 2016;61(2):237-41.	Wrong study design
Chang JT, Mayer M, Jackson RA, Rostron BL, Coleman B, Lee T, et al. Characteristics and Patterns of Cigarette Smoking and Vaping By Past-Year Smokers Who Reported Using Electronic Nicotine Delivery System to Help Quit Smoking in the Past Year: Findings From the 2018–2019 Tobacco Use Supplement to the Current Population Survey. Nicotine & Tobacco Research 2023;25(3):596–601.	No age subgroup analysis
Chen R, Pierce JP, Leas EC, White MM, Kealey S, Strong DR, et al. Use of Electronic Cigarettes to Aid Long-Term Smoking Cessation in the United States: Prospective Evidence From the PATH Cohort Study. Am J Epidemiol 2020;189(12):1529-1537.	No age subgroup analysis
Curran KA, Burk T, Pitt PD, Middleman AB. Trends and Substance Use Associations With E-Cigarette Use in US Adolescents. Clin Pediatr (Phila) 2018;57(10):1191-1198.	Wrong study design
Dai H, Leventhal AM. Association of electronic cigarette vaping and subsequent smoking relapse among former smokers. Drug Alcohol Depend 2019; 199:10-17.	No age subgroup analysis
Edwards KC, Kasza KA, Tang Z, Stanton CA, Sharma E, Halenar MJ, et al. Correlates of tobacco product reuptake and relapse among youth and adults in the USA: findings from the PATH Study Waves 1-3 (2013-2016). Tob Control. 2020;29(Suppl 3):s216-s226.	No age group subgroup analysis
Everard, Colm D; Silveira, Marushka L; Kimmel, Heather L; et al. Association of Electronic Nicotine Delivery System Use With Cigarette Smoking Relapse Among Former Smokers in the United States. JAMA Network Open 2020;3(6):e204813	No age subgroup analysis
Gallus S, Stival C, McKee M, Carreras G, Gorini G, Odone A, et al. Impact of electronic cigarette and heated tobacco product on conventional smoking: an Italian prospective cohort study conducted during the COVID-19 pandemic. Tob Control 2022tobaccocontrol-2022-057368.	No age subgroup analysis
Glasser AM, Vojjala M, Cantrell J, Levy DT, Giovenco DP, Abrams D, et al. Patterns of E-cigarette Use and Subsequent Cigarette Smoking Cessation Over 2 Years (2013/2014-2015/2016) in the Population Assessment of Tobacco and Health Study. Nicotine Tob Res 2021;23(4):669-677.	No age subgroup analysis
Kong G, Chaffee BW, Wu R, Krishnan-Sarin S, Liu F, Leventhal AM, et al. E-cigarette device type and combustible tobacco use: Results from a pooled analysis of 10,482 youth. Drug Alcohol Depend 2022; 232:109279.	Wrong study design
Mok Y, Jeon J, Levy DT, Meza R. Associations Between E-cigarette Use and E-cigarette Flavors With Cigarette Smoking Quit Attempts and Quit Success: Evidence From a U.S. Large, Nationally Representative 2018-2019 Survey. Nicotine Tob Res 2023;25(3):541-552.	No age subgroup analysis
Prakash S, Xu Y, Goldenson NI, Wissmann R, Gougelet R, Shiffman S. Transitions in Smoking among Adults Newly Purchasing the JUUL System. Am J Health Behav 2021;45(3):546-562.	No age subgroup analysis
Simon P, Buta E, Gueorguieva R, Kong G, Morean ME, Camenga DR, et al. Transitions across tobacco use profiles among adolescents: results from the Population Assessment of Tobacco and Health (PATH) study waves 1 and 2. Addiction 2020;115(4):740-747.	Wrong study design
Wei L, Muhammad-Kah RS, Hannel T, Pithawalla YB, Gogova M, Chow S, et al. The impact of cigarette and e-cigarette use history on transition patterns: a longitudinal analysis of the population assessment of tobacco and health (PATH) study, 2013-2015. Harm Reduct J 2020;17(1):45.	No age subgroup analysis
Young-Wolff KC, Klebaner D, Folck B, Tan ASL, Fogelberg R, Sarovar V, et al. Documentation of e-cigarette use and associations with smoking from 2012 to 2015 in an integrated healthcare delivery system. Prev Med 2018;109:113-118.	No age subgroup analysis
